# 6-Aryl-4-cycloamino-1,3,5-triazine-2-amines: synthesis, antileukemic activity, and 3D-QSAR modelling†

**DOI:** 10.1039/d3ra08091a

**Published:** 2024-03-11

**Authors:** Muhammad Syafiq Bin Shahari, Ahmad Junaid, Edward R. T. Tiekink, Anton V. Dolzhenko

**Affiliations:** a Center for Drug Design, College of Pharmacy, University of Minnesota Nils Hasselmo Hall, 312 Church Street SE, Mail Code 1191 Minneapolis Minnesota 55455 USA; b Inimmune Corp. 1121 E Broadway St, Ste 106 Missoula Montana 59802 USA; c Department of Chemistry, Universitat de les Illes Balears Crta de Valldemossa km 7.5 07122 Palma de Mallorca Spain; d School of Pharmacy, Monash University Malaysia Jalan Lagoon Selatan Bandar Sunway Selangor Darul Ehsan 47500 Malaysia anton.dolzhenko@monash.edu; e Curtin Medical School, Curtin Health Innovation Research Institute, Faculty of Health Sciences, Curtin University GPO Box U1987 Perth Western Australia 6845 Australia

## Abstract

Despite significant progress in immunotherapy and chimeric antigen receptor T cell therapy of leukemia, chemotherapy is the major treatment option for the disease. Therefore, the development of potent and safe drugs for standard and targeted chemotherapy of leukemia remains an important task for medicinal chemists. A library of 94 diverse 6-aryl-4-cycloamino-1,3,5-triazine-2-amines was prepared using a one-pot microwave-assisted protocol, which involves a three-component reaction of cyanoguanidine, aromatic aldehydes and cyclic amines, and subsequent dehydrogenative aromatization of the dihydrotriazine intermediates in the presence of alkali. The cytotoxic properties of prepared compounds were evaluated against the leukemic Jurkat T cell line and the selectivity of the 24 most active compounds was also assessed using a normal fibroblast MRC-5 cell line, indicating selective antiproliferative activity against leukemic cells. The structure–activity relationship was analysed, and the prepared 3D-QSAR model was found to predict the antileukemic activity of the compounds with reasonable accuracy. In the cell morphology study, both apoptosis and necrosis features were observed in Jurkat T cells after treatment with the most active compound.

## Abstract 2

New potent and selective antileukemic agents were identified in the screening of 94 compounds prepared using a convenient one-pot three-component approach.

## Graphical abstract

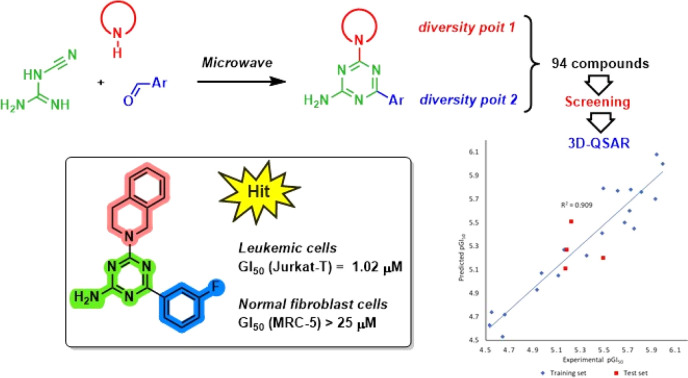

## Introduction

**Fig. 1:**
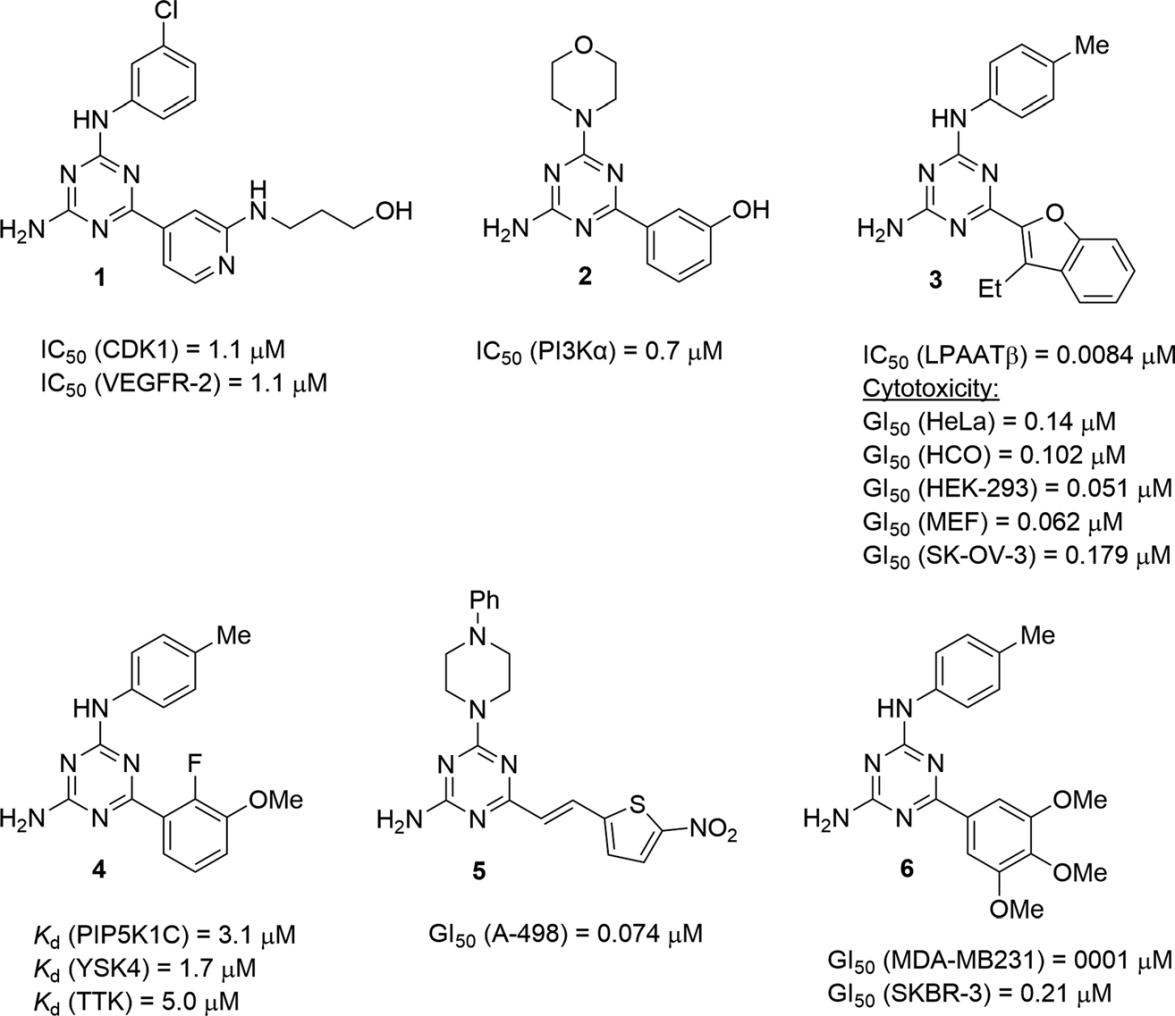
Selected anticancer 1,3,5-triazine-2,4-diamines.

Useful biological properties of 2,4-diamino-1,3,5-triazines substituted at position 6 and one of the amino groups have been well recognised.^1^ Compounds constructed using this scaffold were found to target various bioorganic molecules of therapeutic importance. Structural variations at just two points of diversity allowed modifications of pharmacological profiles from CNS-active molecules to anticancer and antiviral agents. Particularly promising results were obtained in the search for new anticancer agents. For example, developed by Johnson & Johnson, compound 1 (Fig. 1) exhibited the dual inhibition of cancer-related proteins *viz.* cyclin-dependent kinase 1 and vascular endothelial growth factor receptor 2.^2^ The morpholino-substituted triazine 2 was found to inhibit an α isoform of phosphatidylinositol-3 kinase.^3^ A potent inhibitor of lysophosphatidic acid acyltransferase β, compound 3, was effective against several cancer cell lines.^4^ Often, the antiproliferative effect of anticancer triazines is associated with the inhibition of numerous biological targets as was demonstrated by 4 and related compounds inhibiting several important kinases.^5^ Therefore, a strategy of antiproliferative screening prior to the identification of crucial biomolecular targets remains effective in the search for new anticancer agents and has resulted in the development of potent agents, *e.g.*5, which was active against kidney carcinoma A-498 cells.^6^ The broad screening of 6,*N*^2^-diaryl-1,3,5-triazine-2,4-diamines allowed the building of a 3D-QSAR model,^7^ which served for the further design of active compounds, including compound 6 exhibiting a highly potent and selective antiproliferative effect against triple-negative breast cancer MDA-MD231 cells.^8^

The synthesis of 1,3,5-triazines often benefits by performing reactions under microwave irradiation.^9^ Recently, we successfully modified the microwave-assisted three-component synthesis of 6,*N*^2^-diaryl-1,3,5-triazine-2,4-diamines^10^ to prepare 6-aryl-4-cycloamino-1,3,5-triazine-2-amines.^11^ In continuation of our search for new effective antileukemic agents,^12^ we expand herein our method^11^ to synthesise a library of 94 diverse 6-aryl-4-cycloamino-1,3,5-triazine-2-amines. These compounds were also screened for antiproliferative properties against the Jurkat T cell line (acute T cell leukemia) and a 3D-QSAR model was developed to facilitate further antileukemic drug discovery. The selectivity of the most active compounds towards leukemic *vs.* non-cancerous cells was evaluated. Additionally, fluorescence staining was used to explore the effects of the hit compound on cell morphology.

## Results and discussion

### Synthesis

**Scheme 1:**
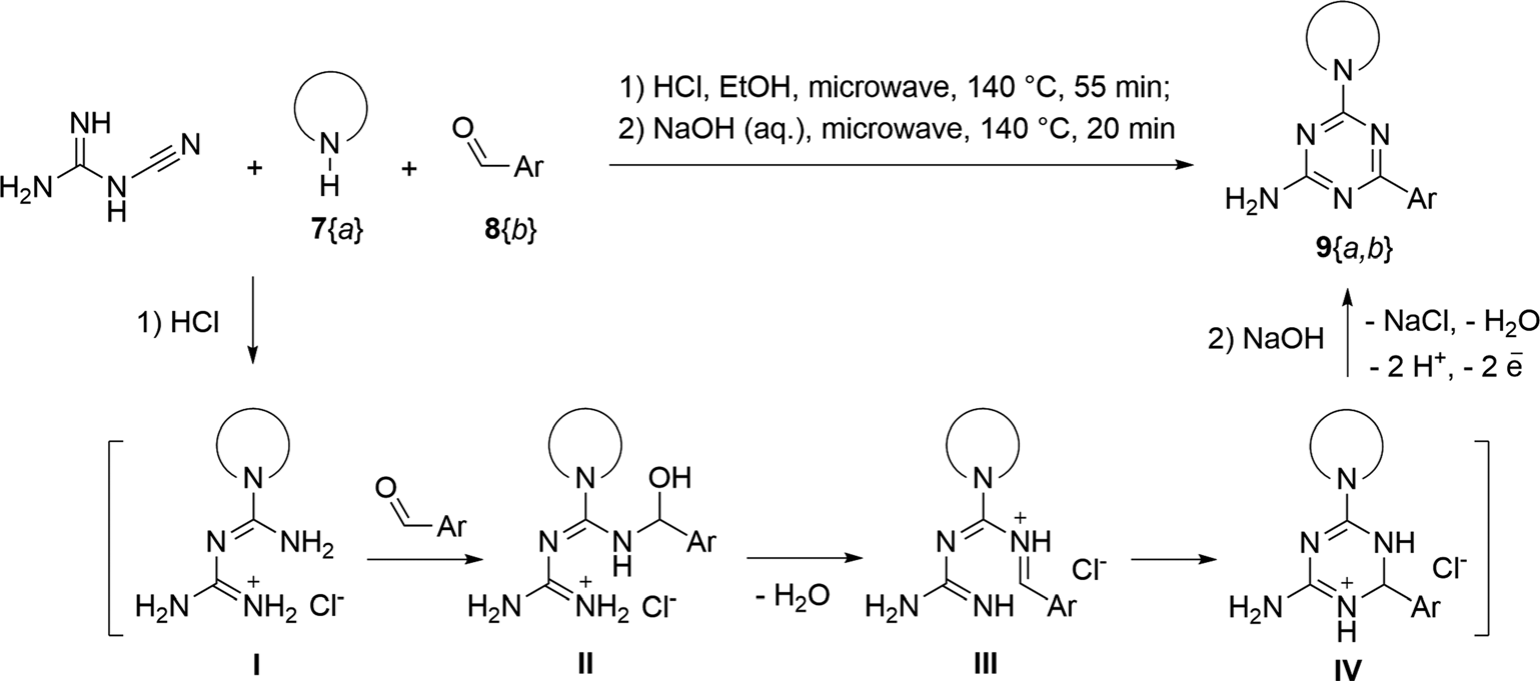
Synthesis of 6-aryl-4-cycloamino-1,3,5-triazine-2-amines 9{*a*,*b*}.

**Fig. 2:**
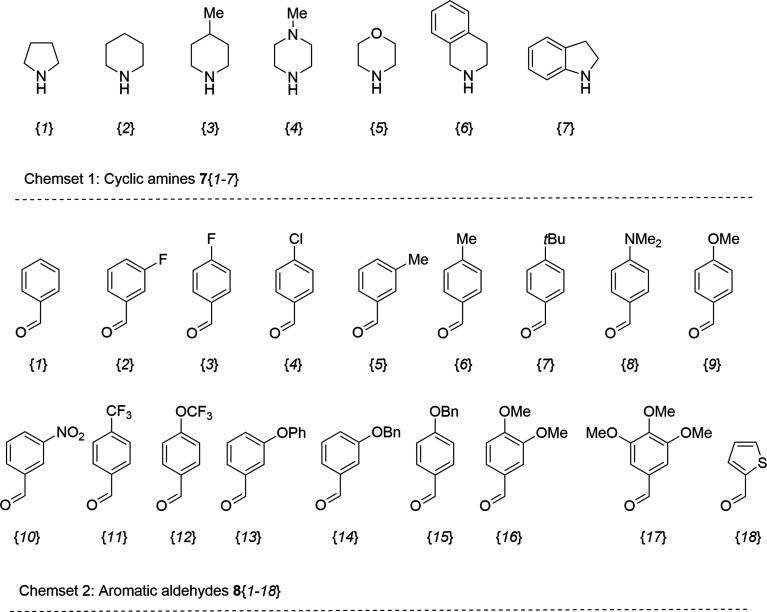
Building blocks for the library of 6-aryl-4-cycloamino-1,3,5-triazine-2-amines 9{*a*,*b*}.

The synthesis of 6-aryl-4-cycloamino-1,3,5-triazine-2-amines 9{*a*,*b*} was carried out applying a recently developed one-pot two-step three-component method.^9^ The initial reaction of cyanoguanidine, cyclic secondary amines 7{*a*}, and aromatic aldehydes 8{*b*} in the presence of hydrochloric acid results in the formation of dihydrotriazine salts IV as key intermediates (Scheme 1). The possible mechanism leading to IV involves the acid-catalysed addition of amines 7{*a*} to the cyanoguanidine nitrile group affording biguanides I, which undergo the subsequent nucleophilic addition to the aldehyde 8{*b*} carbonyl group. The dehydration of adduct II results in the formation of intermediates III, which undergo the dihydrotriazine ring closure. In the second step, the treatment of dihydrortiazine IV with aqueous alkali and further heating of the reaction mixture under microwave irradiation resulted in dehydrogenative aromatization thus affording 6-aryl-4-cycloamino-1,3,5-triazine-2-amines 9{*a*,*b*}. Both steps were effectively performed under microwave irradiation in a one-pot manner producing a library of 94 compounds with two points of diversity. The prepared library of 6-aryl-4-cycloamino-1,3,5-triazine-2-amines 9{*a*,*b*} covers a broad chemical space using combinations of various secondary cyclic amines 7{1–7} (Chemset 1) and aromatic aldehydes 8{1–18} (Chemset 2) as building blocks (Fig. 2). The product yields varied substantially and depended more on the type of cyclic amines 7. In general, higher yields (up to 78%) were obtained in reactions of tetrahydroisoquinoline (7{6}) and indoline (7{7}).

The structures of the prepared compounds were confirmed by ^1^H and ^13^C NMR spectroscopic data. In ^13^C NMR spectra of 9{*a*,*b*}, the characteristic downfield-shifted signals of triazine ring carbon atoms appear at 163.0–169.8 ppm. The common structural features of 9{*a*,*b*} include a primary amino group (position 2 of the 1,3,5-triazine ring) with a lone pair of electrons delocalised over the triazine ring. The chemical shift of the amino group signal in ^1^H NMR depends on the substituents in positions 4 and 6 of the triazine. For compounds 9{1–6,*b*}, it varies from 6.61 ppm to 7.11 ppm with the downfield shift for compounds with electron-withdrawing substituents in the 6-aryl group. Moreover, the strong electron-withdrawing effect of the 3-nitro group in 9{*a*,10} results in increased planarity of the amino group and restricted rotation around the C–N bond that can be observed as a splitting the amino group signal into two singlets, *e.g.* 7.07 ppm and 7.17 ppm for 9{4,10}. The 4-indolino-substituted triazines 9{7,*b*} have more downfield-shifted 2-amino group signals (7.12–7.34 ppm) than compounds with cyclic aliphatic amino groups in position 4.

The cycloamino nitrogen electron pair was also delocalised over the triazine ring. That was particularly prominent for the 4-pyrrolidino-substituted 9{1,*b*} series demonstrating restricted rotation around the C–N bond connecting two rings. In ^1^H NMR spectra, this restricted rotation manifested in two broad triplets of magnetically non-equivalent N-bound methylenic groups at 4.45–3.49 ppm and 3.55–3.64 ppm. The same groups gave two separate signals in the 45.6–45.9 ppm range in ^13^C NMR spectra. Moreover, the other two methylenic groups of the pyrrolidine ring can also be distinguished in ^13^C NMR spectra as two independent signals about 24.6 ppm and 24.7 ppm (in ^1^H NMR spectra signals of these groups overlap).

### X-ray crystallography of 9{2,6}

**Fig. 3:**
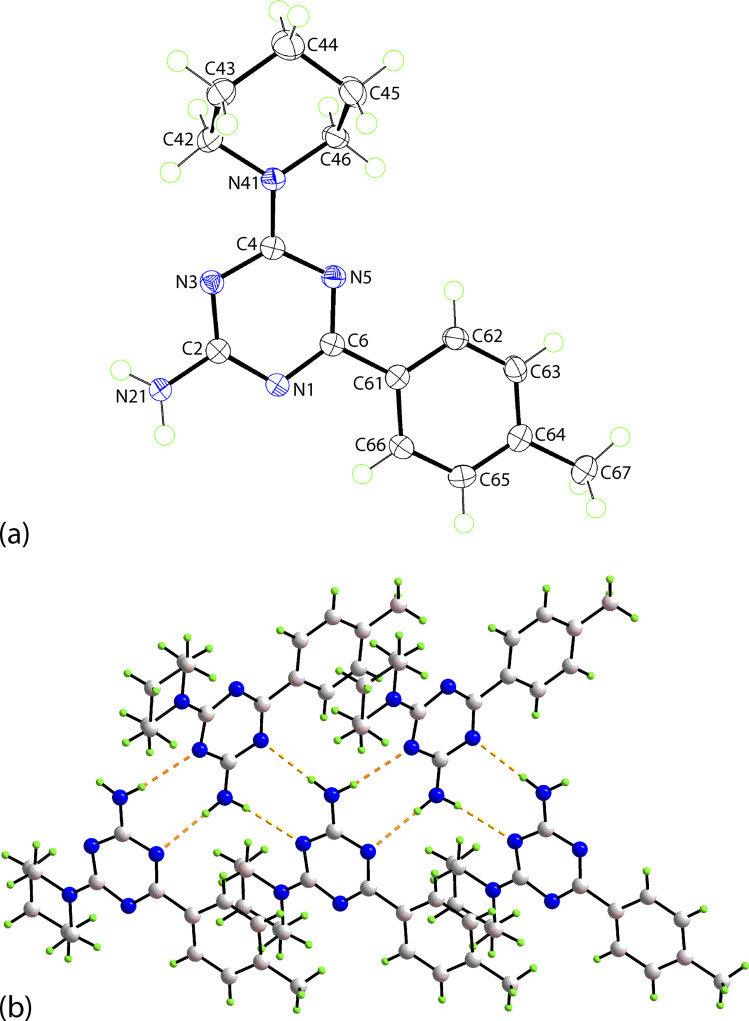
Crystallographic images for 9{2,6}: (a) the molecular structure showing atom-labelling scheme and displacement ellipsoids at the 70% probability level and (b) a view of the supramolecular tape formed in the crystal and featuring amine-N–H⋯N(triazine) hydrogen-bonding highlighted as orange dashed lines.

The structure of the newly prepared 6-aryl-4-cycloamino-1,3,5-triazine-2-amines 9{*a*,*b*} derivatives was also confirmed by an X-ray crystallographic analysis on a representative compound, namely 9{2,6}. The molecular structure is illustrated in Fig. 3a and comprises a central triazine ring connected at the C2-, C4- and C6-positions by amine, piperidino and 4-methylphenyl substituents, respectively. Non-systematic variations are noted in the C–N bond lengths within the triazine ring. For example, the greatest differential in C–N bonds is noted for the N5 atom, *i.e.* C6–N5 [1.3289(15) Å] and C4–N5 [1.3560(15) Å], suggesting the C2–N1 bond [1.3543(15) Å] should be shorter than the C6–N1 bond length [1.3396(15) Å] but the inverse is true; similarly, the C4–N3 bond [1.3513(15) Å] is longer than the C2–N3 bond [1.3424(15) Å]. These differences relate to the greater electron-withdrawing effect of the 4-methylphenyl [C6–C61 = 1.4887(16) Å] compared to the electron-donating amine [C2–N21 = 1.3443(15) Å] and piperidino [C4–N41 = 1.3490(15) Å] substituents. Substantiating this is the observation of the co-planar relationship between the triazine [r.m.s.d. = 0.031 Å with the maximum deviation of 0.035(1) Å for the C4 atom] and 4-methylphenyl rings as evident in the dihedral angle between them of 2.48(5)°. It is concluded there is significant delocalisation of π-electron density over the triazine ring as noted above from the NMR analyses. The piperidino ring has the expected chair conformation with the appended C4 atom occupying an equatorial position with respect to the N41 atom.

In the crystal, amine-N–H⋯N(triazine) hydrogen-bonding leads to the formation of non-symmetric, eight-membered {···NCNH}_2_ synthons which are connected into a supramolecular tape, Fig. 3b. Being propagated along a crystallographic 2-fold screw symmetry along the *b*-axis, the tape has a helical topology with the dihedral angle between adjacent triazine rings being 56.46(3)°. The connections between translationally related tapes are off-set π⋯π stacking interactions between 4-methylphenyl rings, as illustrated in ESI Fig. S1(a).† The supramolecular layers have a zigzag topology with off-set layers inter-digitating along the *a*-axis in an ···ABA··· fashion without directional interactions between them. A view of the unit-cell contents for 9{2,6} is given in ESI Fig. S1(b)† with the caption including geometric parameters characterising the specified interactions.

### Cytotoxicity evaluation

The prepared compounds 9{*a*,*b*} were screened for their antileukemic activity against Jurkat T cells at 10 μM using an MTS assay with the calculation of the percentage of cell viability performed 72 h after treatment (Table 1). The Jurkat T cell line was chosen for its reliability as a model for chemotherapeutic studies, particularly in T-cell leukemia.^13^

**Table 1:** Antiproliferative screening of 6-aryl-4-cycloamino-1,3,5-triazine-2-amines 9{*a*,*b*} at 10 μM against Jurkat T cells

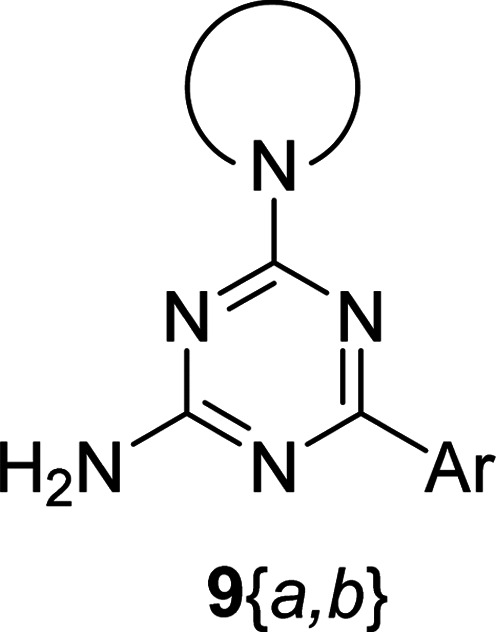			
Compound	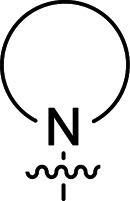	Ar	Cell viability (%)^a^
9{1,1}	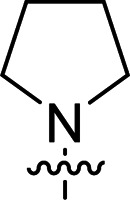	Ph	100
9{1,2}		3-FC_6_H_4_	100
9{1,3}		4-FC_6_H_4_	97
9{1,5}		3-MeC_6_H_4_	82
9{1,6}		4-MeC_6_H_4_	100
9{1,7}		4-tBuC_6_H_4_	88
9{1,9}		4-MeOC_6_H_4_	100
9{1,10}		3-NO_2_C_6_H_4_	94
9{1,11}		4-CF_3_C_6_H_4_	78
9{1,12}		4-CF_3_OC_6_H_4_	96
9{1,13}		3-PhOC_6_H_4_	87
9{1,15}		4-BnOC_6_H_4_	94
9{1,16}		3,4-MeOC_6_H_3_	95
9{1,17}		3,4,5-MeOC_6_H_2_	98
9{1,18}		2-Thienyl	100
9{2,1}	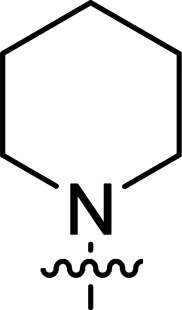	Ph	100
9{2,2}		3-FC_6_H_4_	62
9{2,3}		4-FC_6_H_4_	98
9{2,4}		4-ClC_6_H_4_	83
9{2,6}		4-MeC_6_H_4_	100
9{2,9}		4-MeOC_6_H_4_	100
9{2,10}		3-NO_2_C_6_H_4_	100
9{2,12}		4-CF_3_OC_6_H_4_	12
9{2,13}		3-PhOC_6_H_4_	10
9{2,14}		3-BnOC_6_H_4_	8
9{3,1}	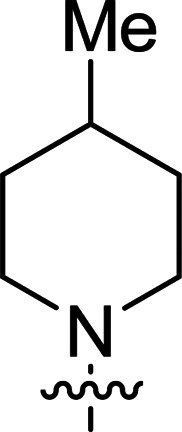	Ph	100
9{3,2}		3-FC_6_H_4_	61
9{3,3}		4-FC_6_H_4_	86
9{3,4}		4-ClC_6_H_4_	79
9{3,6}		4-MeC_6_H_4_	100
9{3,8}		4-Me_2_NC_6_H_4_	84
9{3,9}		4-MeOC_6_H_4_	100
9{3,10}		3-NO_2_C_6_H_4_	84
9{3,11}		4-CF_3_C_6_H_4_	11
9{3,12}		4-CF_3_OC_6_H_4_	5
9{3,17}		3,4,5-MeOC_6_H_2_	95
9{3,18}		2-Thienyl	35
9{4,1}	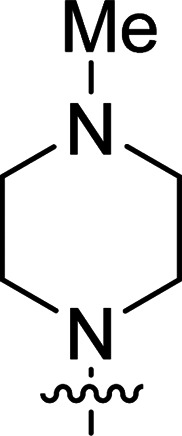	Ph	100
9{4,2}		3-FC_6_H_4_	95
9{4,3}		4-FC_6_H_4_	100
9{4,4}		4-ClC_6_H_4_	100
9{4,5}		3-MeC_6_H_4_	95
9{4,6}		4-MeC_6_H_4_	100
9{4,7}		4-tBuC_6_H_4_	16
9{4,8}		4-Me_2_NC_6_H_4_	99
9{4,9}		4-MeOC_6_H_4_	100
9{4,10}		3-NO_2_C_6_H_4_	100
9{4,11}		4-CF_3_C_6_H_4_	100
9{4,12}		4-CF_3_OC_6_H_4_	100
9{4,14}		3-BnOC_6_H_4_	73
9{4,15}		4-BnOC_6_H_4_	74
9{4,16}		3,4-MeOC_6_H_3_	100
9{4,17}		3,4,5-MeOC_6_H_2_	100
9{5,1}	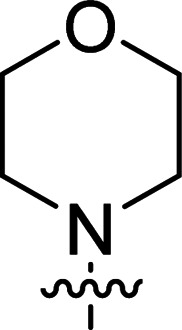	Ph	98
9{5,2}		3-FC_6_H_4_	100
9{5,3}		4-FC_6_H_4_	100
9{5,4}		4-ClC_6_H_4_	100
9{5,5}		3-MeC_6_H_4_	100
9{5,6}		4-MeC_6_H_4_	100
9{5,7}		4-tBuC_6_H_4_	60
9{5,8}		4-Me_2_NC_6_H_4_	100
9{5,9}		4-MeOC_6_H_4_	100
9{5,12}		4-CF_3_OC_6_H_4_	100
9{5,13}		3-PhOC_6_H_4_	78
9{5,14}		3-BnOC_6_H_4_	63
9{5,15}		4-BnOC_6_H_4_	94
9{5,16}		3,4-MeOC_6_H_3_	100
9{5,17}		3,4,5-MeOC_6_H_2_	100
9{5,18}		2-Thienyl	77
9{6,1}	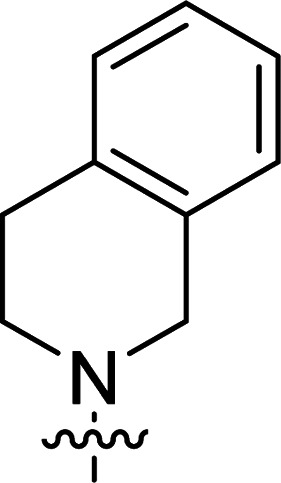	Ph	12
9{6,2}		3-FC_6_H_4_	1
9{6,4}		4-ClC_6_H_4_	2
9{6,5}		3-MeC_6_H_4_	2
9{6,6}		4-MeC_6_H_4_	4
9{6,7}		4-tBuC_6_H_4_	1
9{6,8}		4-Me_2_NC_6_H_4_	100
9{6,9}		4-MeOC_6_H_4_	14
9{6,11}		4-CF_3_C_6_H_4_	3
9{6,12}		4-CF_3_OC_6_H_4_	3
9{6,13}		3-PhOC_6_H_4_	4
9{6,14}		3-BnOC_6_H_4_	23
9{6,15}		4-BnOC_6_H_4_	59
9{6,16}		3,4-MeOC_6_H_3_	23
9{6,17}		3,4,5-MeOC_6_H_2_	96
9{6,18}		2-Thienyl	78
9{7,3}	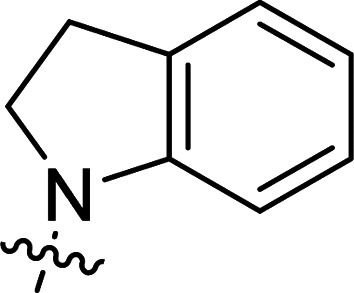	4-FC_6_H_4_	35
9{7,8}		4-Me_2_NC_6_H_4_	30
9{7,9}		4-MeOC_6_H_4_	85
9{7,11}		4-CF_3_C_6_H_4_	53
9{7,12}		4-CF_3_OC_6_H_4_	73
9{7,15}		4-BnOC_6_H_4_	32
9{7,16}		3,4-MeOC_6_H_3_	67
9{7,17}		3,4,5-MeOC_6_H_2_	10
9{7,18}		2-Thienyl	20

a Mean value of three independent experiments.

The primary single-point antiproliferative screening of the prepared 6-aryl-4-cycloamino-1,3,5-triazine-2-amines 9{*a*,*b*} suggested that the effectiveness of a compound strongly depends on the nature of the cycloamino substituents and is generally less affected by 6-aryl substituents introduced by aldehydes.

No substantial activity was observed at the tested concentration for 6-aryl-4-pyrrolidino-1,3,5-triazine-2-amines 9{1,1–18}. However, their analogues with piperidino, 4-methylpiperidino and 4-methylpiperazino groups in position 4 of the triazine ring were more active, particularly those possessing bulkier lipophilic substituents at the 6-phenyl ring of 9{2–4,*b*}. Thus, compounds with *tert*-butyl (9{4,7}), trifluoromethyl (9{3,11}), trifluoromethoxy (9{2,12} and 9{3,12}), phenoxy (9{2,13}) or benzoxy (9{2,14}) groups effectively inhibited Jurkat T cell growth. Some studies have shown the importance of morpholine as a substituent in various anticancer triazines.^14,15^ However, in our library, 6-aryl-4-morpholino-1,3,5-triazine-2-amines 9{5,1–18} did not exhibit appreciable antileukemic activity. In contrast, most of the triazines 9{6,*b*} prepared using 1,2,3,4-tetrahydroisoquinoline (7{6}) as a cyclic amine demonstrated prominent antiproliferative properties against Jurkat T cells. Applied at the screening concentration, compounds 9{6,2–7} and 9{6,11–13} nearly completely halted the Jurkat T cell proliferation. Several potent antiproliferative compounds were also identified among 6-aryl-4-indolino-1,3,5-triazine-2-amines 9{7,1–18}.

Overall, 24 out of 94 prepared 6-aryl-4-cycloamino-1,3,5-triazine-2-amines 9{*a*,*b*} were found to inhibit the Jurkat T cell growth by 50% or more at the screening concentration. These compounds were selected for further testing to estimate their 50% growth inhibition (GI_50_) value. Standard antileukemic drugs mercaptopurine, methotrexate and cytarabine were chosen as positive controls. Compound 9{6,2} was identified as the most active compound inhibiting Jurkat T cell proliferation with the GI_50_ value of 1.02 ± 0.30 μM. This compound was substantially more potent than mercaptopurine and demonstrated activity comparable to that of methotrexate and cytarabine (Table 2).

**Table 2:** Inhibition of Jurkat T cell growth by most active compounds selected from the initial screening

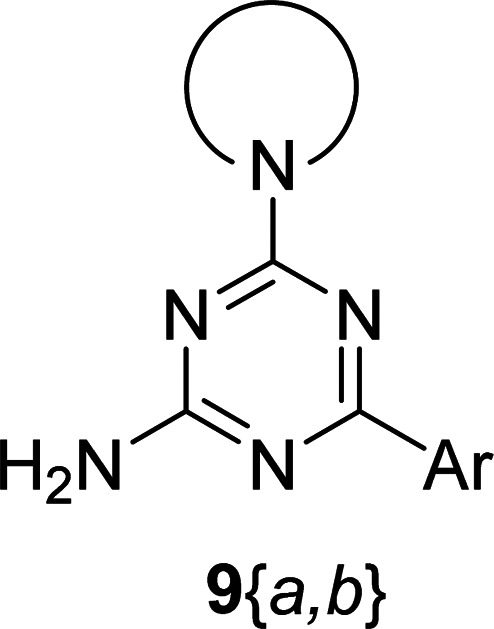			
Compound	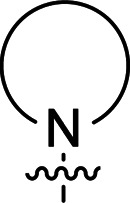	Ar	GI_50_^a^ ± SEM^b^ (μM)
9{2,12}	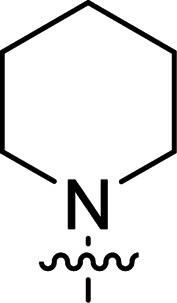	4-CF_3_OC_6_H_4_	10.81 ± 0.13
9{2,13}		3-PhOC_6_H_4_	6.88 ± 1.22
9{2,14}		3-BnOC_6_H_4_	7.68 ± 0.11
9{3,11}	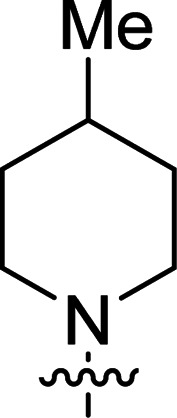	4-CF_3_C_6_H_4_	4.51 ± 0.15
9{3,12}		4-CF_3_OC_6_H_4_	3.23 ± 0.88
9{3,18}		2-Thienyl	28.17 ± 1.03
9{4,7}	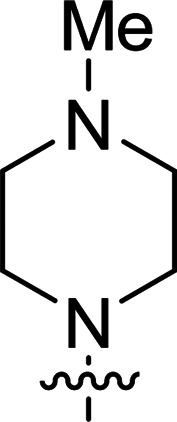	4-tBuC_6_H_4_	21.83 ± 1.10
9{6,1}	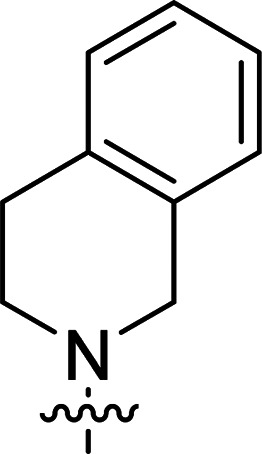	Ph	1.95 ± 0.25
9{6,2}		3-FC_6_H_4_	1.02 ± 0.30
9{6,4}		4-ClC_6_H_4_	3.30 ± 0.65
9{6,5}		3-MeC_6_H_4_	1.14 ± 0.13
9{6,6}		4-MeC_6_H_4_	1.79 ± 0.58
9{6,7}		4-tBuC_6_H_4_	2.43 ± 0.57
9{6,9}		4-MeOC_6_H_4_	29.59 ± 2.20
9{6,11}		4-CF_3_C_6_H_4_	3.23 ± 0.16
9{6,12}		4-CF_3_OC_6_H_4_	1.18 ± 0.22
9{6,13}		3-PhOC_6_H_4_	1.54 ± 0.66
9{6,14}		3-BnOC_6_H_4_	6.54 ± 0.34
9{6,16}		3,4-MeOC_6_H_3_	11.64 ± 2.12
9{7,3}	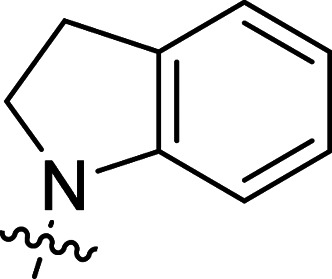	4-FC_6_H_4_	1.89 ± 0.48
9{7,8}		4-Me_2_NC_6_H_4_	2.12 ± 0.97
9{7,15}		4-BnOC_6_H_4_	23.07 ± 0.08
9{7,17}		3,4,5-MeOC_6_H_2_	6.73 ± 0.43
9{7,18}		2-Thienyl	6.03 ± 0.64
Mercaptopurine^c^	—	—	11.12 ± 4.89
Methotrexate^c^	—	—	0.37 ± 0.03
Cytarabine^c^	—	—	0.29 ± 0.01

a Concentration (μM) required to inhibit Jurkat T cell growth by 50%, values are the mean of three independent experiments.

b Standard error of the mean.

c Positive controls.

The majority of tetrahydroisoquinolino-substituted compounds 9{6,*b*} demonstrated substantial inhibition of the leukemic cell growth. More prominent activity in this sub-library was observed for compounds with lipophilic substituents in the phenyl ring in position 6, while hydrophilic groups, like 4-dimethylamino in 9{6,8} or 3,4,5-trimethoxy in 9{6,17}, were detrimental for the activity. The replacement of the phenyl ring in position 6 by a thienyl substituent in compound 9{6,18} also made this compound inactive against Jurkat T cells. Importantly, this trend was not observed in indolino-substituted sub-library 9{7,*b*}, where 9{7,8}, 9{7,17}, and 9{7,18} were among the most active in the series. Therefore, we attempted to comprehend the structural requirements for the activity using a 3D-QSAR model. The GI_50_ values for active compounds were used to build a 3D-QSAR model describing structural requirements for antileukemic activity of 6-aryl-4-cycloamino-1,3,5-triazine-2-amines 9{*a*,*b*}.

All compounds selected in the primary screening were also tested for their effects on the viability of normal fibroblast MRC-5 cells. None of the compounds applied at a concentration of 25 μM showed significant inhibition of the MRC-5 cell growth. These results suggested a beneficial safety profile, indicating that the identified active compounds selectively inhibited Jurkat T cell growth without visible toxic effects on normal cells.

### Cell morphology analysis

**Fig. 4:**
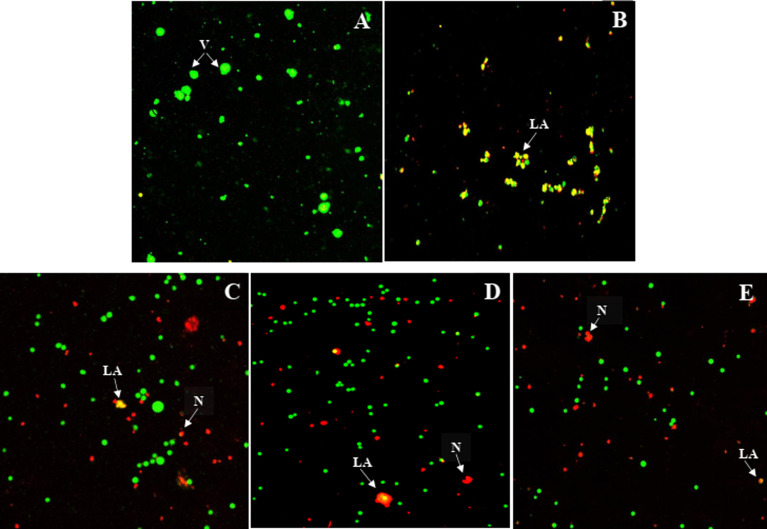
Compound 9{6,2} triggered the aponecrosis in the Jurkat T cells assessed by the AO/PI staining. Jurkat T cells were treated with compound 9{6,2} (GI_50_ conc. = 1.02 ± 0.30 μM) with 24 h (C), 48 h (D), and 72 h (E) exposure. The untreated cells (A) were used as a negative control and methotrexate (GI_50_ conc. = 0.37 ± 0.03 μM, 72 h) as a positive control (B). Images represent one of the three independent experiments. Label meaning: LA – late apoptosis, N– necrosis, and V – viable cells.

The effects of the most active compound 9{6,2} on Jurkat T cell morphology were explored using cell imaging with the acridine orange and propidium iodide (AO/PI) fluorescence staining. The AO/PI staining was employed to assess the Jurkat T cells morphological changes 24 h, 48 h and 72 h after the treatment with the most potent compound 9{6,2}. The GI_50_ value for this compound was selected as the treatment concentration. Bright-green and round-shaped cells were observed in the untreated cells, indicating normal morphology of healthy alive cells (Fig. 4A). The positive control, methotrexate, induced the late apoptotic morphological changes indicated by the reddish-orange color due to AO staining the denatured DNA of the treated Jurkat T cells (Fig. 4B). It was observed that 24 h after the treatment with compound 9{6,2}, some red-stained cells became visible, thus indicating the necrotic type of cell death.^16^ Moreover, some Jurkat T cells underwent late stages of apoptosis as shown by the reddish-orange stain (Fig. 4C). Similar events also occurred 48 h and 72 h after the treatment but with a lower number of viable cells (Fig. 4D and E). The changes observed in the fluorescent staining experiments with 9{6,2} on Jurkat T cells suggested that cell death occurred within the first 24 h and probably involved aponecrosis, which might result from the incomplete biochemical process of apoptosis.^17^

### 3D-QSAR modelling

To better understand structural requirements for antileukemic activity against Jurkat T within the 6-aryl-4-cycloamino-1,3,5-triazine-2-amine chemical space, a 3D-QSAR model was built applying the 3D-QSAR protocol of Discovery Studio version 18^18^ to the experimentally obtained leukemic cell growth inhibition (GI_50_) data. The model data set comprised 24 compounds with GI_50_ values against Jurkat T cells ranging from 1.02 to 29.59 μM. The GI_50_ values were converted to the negative logarithmic scale (pGI_50_). After aligning compounds to the minimum energy, they were split randomly into a training set (20 compounds) and a test set (4 compounds).

**Fig. 5:**
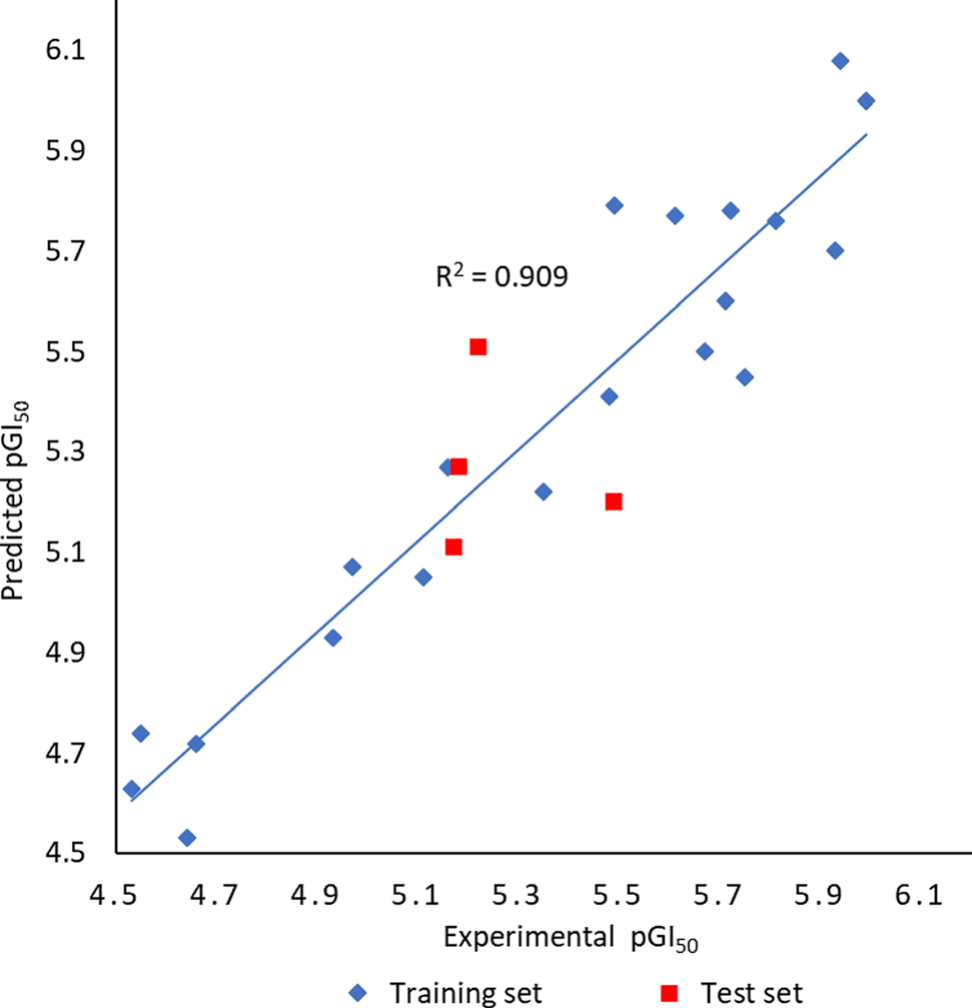
Plot of experimental *versus* predicted pGI_50_ for the developed 3D-QSAR model.

The CHARMm force field was used to build the QSAR model, and the model quality was assessed through two parameters: correlation coefficient (*r*^2^) and root-mean-square error (RMSE). The experimental and predicted pGI_50_ values were found to correlate well in the partial least squares (PLS) model resulting in the *r*^2^ value of 0.91 (Fig. 5). The capability of the 3D-QSAR model to predict the GI_50_ values was also confirmed by the acceptable RMSE value of 0.21. The experimental pGI_50_ values are compared with predicted ones and the values together with the residual errors are presented in Table 3.

**Table 3:** Experimental and 3D-QSAR predicted inhibitory activities of compounds 9{a,*b*}

Compound^a^	Experimental pGI_50_	Predicted pGI_50_	Residual error
9{2,12}	4.97	5.07	−0.10
9{2,13}	5.16	5.27	−0.11
9{2,14}	5.11	5.05	0.06
1{3,11}	5.35	5.22	0.13
1**{3,12}**	5.49	5.20	0.29
9{3,18}	4.55	4.74	−0.19
9{4,7}	4.66	4.72	−0.06
9{6,1}	5.71	5.60	0.11
9{6,2}	5.99	6.00	−0.01
9{6,4}	5.48	5.41	0.07
9{6,5}	5.94	6.08	−0.14
9{6,6}	5.75	5.45	0.30
9{6,7}	5.61	5.77	−0.16
9{6,9}	4.53	4.63	−0.10
9{6,11}	5.49	5.79	−0.29
9{6,12}	5.93	5.70	0.23
9{6,13}	5.81	5.76	0.05
9**{6,14}**	5.18	5.27	−0.09
9{6,16}	4.93	4.93	0.01
9{7,3}	5.72	5.78	−0.06
9{7,8}	5.67	5.50	0.17
9{7,15}	4.64	4.53	0.10
9**{7,17}**	5.17	5.11	0.06
9**{7,18}**	5.22	5.51	−0.29

a Bold compounds were randomly selected for the test set.

**Fig. 6:**
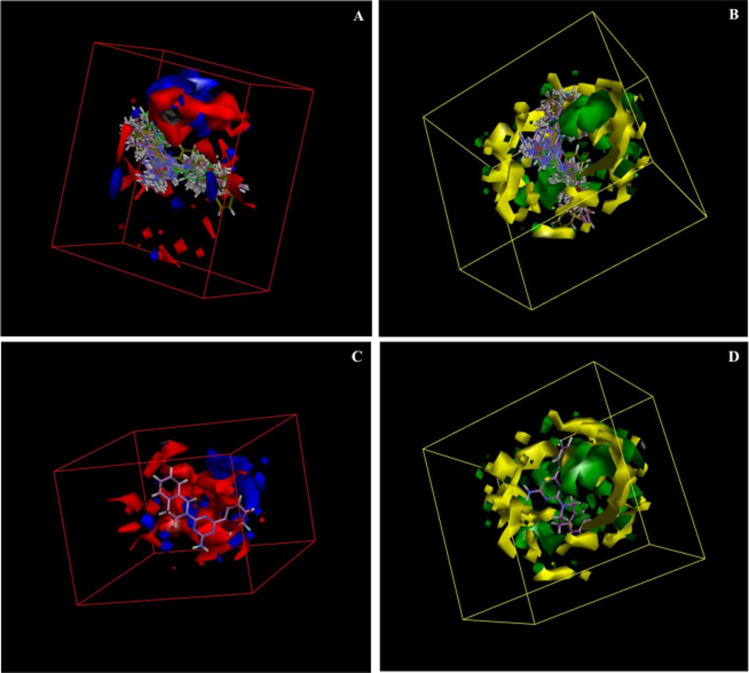
3D-QSAR model coefficients on electrostatic grid (A) and van der Waals grid (B); 3D-QSAR model coefficients of compound 9{6,2} on the electrostatic grid (C) and van der Waals grid (D).

The electrostatic potential grid and van der Waals grid in Fig. 6 illustrate the alignment of active molecules 9{*a*,*b*} to the iso-surface of the developed 3D-QSAR model. Red contours in the electrostatic potential grid (Fig. 6A) indicate regions where an increase in electron density is beneficial for activity, while the opposite effect occurs in the blue contour regions. The presence of bulky groups at the green contour region of the steric map (Fig. 6B) suggests higher antileukemic activity, while the yellow contour shows areas where an increase in the steric bulk results in a lower activity. Based on the 3D-QSAR model, the bulkier groups are preferred at the cycloamino part of 9{*a*,*b*}. These findings fit a more general trend observed for 6-aryl-4-cycloamino-1,3,5-triazine-2-amines 9{*a*,*b*}: an increase of antiproliferative activity from the inactive 4-pyrrolidino substituted 9{1,*b*} to more active compound series 9{2,*b*} and 9{3,*b*} with 4-piperidino and 4-(4-methylpiperidino) groups and the most active 4-tetrahydroisoquinolino and 4-indolino substituted triazine sub-libraries 9{6,*b*} and 9{7,*b*}.

A minimal residual error (−0.01) was observed for the most active triazine 9{6,2} (GI_50_ = 1.02 μM). This compound is well-aligned with the developed 3D-QSAR map in electrostatic and steric requirements as illustrated by its positioning in the electrostatic potential and van der Waals grids in Fig. 6C and D.

## Conclusions

Using a three-component microwave-assisted method, we prepared a library of 6-aryl-4-cycloamino-1,3,5-triazine-2-amines for evaluation of antileukemic activity. The method allowed the preparation of 94 diverse compounds from commercially available starting materials in a one-pot process with two simple synthetic steps with chromatography-free product isolation. The majority of the compounds were prepared for the first time. The screening of synthesised compounds against the Jurkat T cells identified 24 compounds with substantial antiproliferative properties comparable with those of standard antileukemic drugs. No effect on the proliferation of normal fibroblast MRC-5 cells was observed even at higher concentrations of the active antileukemic compounds thus indicating their selectivity. The fluorescence staining experiments suggested the aponecrosis pathway of the Jurkat T cell death. To understand structure–activity relationships and facilitate the further search for antileukemic agents among 6-aryl-4-cycloamino-1,3,5-triazine-2-amines, a 3D-QSAR model was successfully developed. The hit compounds identified in the screening and the developed 3D-QSAR model can be used for further lead design and optimisation together with understanding molecular targets involved in the biological response.

## Experimental

### General

Melting points (uncorrected) were determined on a Stuart™ SMP40 automatic melting point apparatus. The ^1^H and ^13^C NMR spectra were recorded on a Bruker Fourier NMR spectrometer (300 MHz) using DMSO-d_6_ as a solvent and TMS as an internal reference. Microwave-assisted reactions were carried out using the closed-vessel focused single-mode settings in a Monowave 400 microwave synthesizer (Anton Paar, Austria) controlling reaction temperature *via* the equipped IR sensor.

### General procedure for the synthesis of 6-aryl-4-cycloamino-1,3,5-triazine-2-amines (9{*a*,*b*})

To the mixture of cyanoguanidine (0.21 g, 2.5 mmol), (het)arylaldehyde (2.5 mmol), and cyclic amine (2.5 mmol) in EtOH (2 mL) in a 10 mL seamless pressure vial, concentrated HCl (0.21 mL, 2.5 mmol) was added. The reaction mixture was irradiated in the Monowave 400 (Anton Paar, Austria) microwave reactor operating at a maximal microwave power output of up to 850 W at 140 °C for 55 min. After cooling to room temperature, an aqueous solution of NaOH (5 N, 1 mL) was added to the reaction mixture followed by microwave irradiation for another 20 min at 140 °C. After cooling, the precipitated product was filtered, washed with water, and recrystallised from an appropriate solvent affording desired products 9{*a*,*b*}. The synthesis and characterization of compounds 9{1–4,1}, 9{5,1–9}, 9{5,12–18}, and 9{6,1} was described previously.^11^

### 6-(3-Fluorophenyl)-4-pyrrolidino-1,3,5-triazine-2-amine (9{1,2})

White solid; yield 102 mg (16%); mp 182–183 °C (EtOH). ^1^H NMR (300 MHz, DMSO-d_6_): *δ* 1.89–1.93 (4H, m), 3.45 (2H, br t, *J* = 6.5 Hz), 3.61 (2H, br t, *J* = 6.6 Hz), 6.89 (2H, br s), 7.36 (1H, dddd, *J* = 0.9, 2.6, 8.5, 8.5 Hz), 7.52 (1H, td, *J* = 8.0, 6.0 Hz), 7.99 (1H, ddd, *J* = 1.4, 2.7, 10.6 Hz), 8.13 (1H, ddd, *J* = 1.2, 1.2, 7.7 Hz). ^13^C NMR (75 MHz, DMSO-d_6_): *δ* 24.6, 24.7, 45.7, 45.8, 113.9 (d, *J* = 22.8 Hz), 117.8 (d, *J* = 21.1 Hz), 123.6 (d, *J* = 2.2 Hz), 130.1 (d, *J* = 7.4 Hz), 139.8 (d, *J* = 7.5 Hz), 162.1 (d, *J* = 242.4 Hz), 163.4, 166.8, 167.9. Anal. Calcd for C_13_H_14_FN_5_: C, 60.22; H, 5.44; N, 27.01. Found: C, 60.12; H, 5.53; N, 26.88.

### 6-(4-Fluorophenyl)-4-pyrrolidino-1,3,5-triazine-2-amine (9{1,3})

Brownish solid; yield 73 mg (11%); mp 202–203 °C (EtOH). ^1^H NMR (300 MHz, DMSO-d_6_): *δ* 1.88–1.90 (4H, m), 3.47 (2H, br t, *J* = 6.5 Hz), 3.60 (2H, br t, *J* = 6.6 Hz), 6.84 (2H, br s), 7.29 (2H, dd, *J* = 8.9, 8.9 Hz), 8.34 (2H, dd, *J* = 5.8, 8.9 Hz). ^13^C NMR (75 MHz, DMSO-d_6_): *δ* 24.65, 24.73, 45.7, 45.8, 114.9 (2C, d, *J* = 22.4 Hz), 130.0 (2C, d, *J* = 8.9 Hz), 133.5, 163.4, 164.0 (d, *J* = 248.7 Hz), 166.8, 168.1. Anal. Calcd for C_13_H_14_FN_5_: C, 60.22; H, 5.44; N, 27.01. Found: C, 60.17; H, 5.49; N, 26.95.

### 6-(3-Methylphenyl)-4-pyrrolidino-1,3,5-triazine-2-amine (9{1,5})

Brownish solid; yield 104 mg (16%); mp 185–186 °C (EtOH). ^1^H NMR (300 MHz, DMSO-d_6_): *δ* 1.88–1.93 (4H, m), 2.37 (3H, s), 3.46 (2H, br t, *J* = 6.5 Hz), 3.61 (2H, br t, *J* = 6.5 Hz), 6.80 (2H, br s), 7.32–7.35 (2H, m), 8.08–8.12 (2H, m). ^13^C NMR (75 MHz, DMSO-d_6_): *δ* 21.5, 24.6, 24.7, 45.7, 45.8, 124.9, 127.9, 128.2 (2C), 131.6, 137.1, 163.5, 166.9, 169.2. Anal. Calcd for C_14_H_17_N_5_: C, 65.86; H, 6.71; N, 27.43. Found: C, 65.71; H, 6.79; N, 27.34.

### 6-(4-Methylphenyl)-4-pyrrolidino-1,3,5-triazine-2-amine (9{1,6})

Brownish solid; yield 111 mg (17%); mp 229–230 °C (EtOH). ^1^H NMR (300 MHz, DMSO-d_6_): *δ* 1.88–1.92 (4H, m), 2.36 (3H, s), 3.46 (2H, br t, *J* = 6.5 Hz), 3.60 (2H, br t, *J* = 6.5 Hz), 6.77 (2H, br s), 7.26 (2H, d, *J* = 8.1 Hz), 8.20 (2H, d, *J* = 8.2 Hz). ^13^C NMR (75 MHz, DMSO-d_6_): *δ* 21.0, 24.65, 24.73, 45.6, 45.8, 127.7 (2C), 128.6 (2C), 134.4, 140.7, 163.5, 166.9, 169.1. Anal. Calcd for C_14_H_17_N_5_: C, 65.86; H, 6.71; N, 27.43. Found: C, 65.78; H, 6.77; N, 27.38.

### 6-[4-(*tert*-Butyl)phenyl]-4-pyrrolidino-1,3,5-triazine-2-amine (9{1,7})

White solid; yield 144 mg (19%); mp 196–197 °C (EtOH). ^1^H NMR (300 MHz, DMSO-d_6_): *δ* 1.31 (9H, s), 1.88–193 (4H, m), 3.46 (2H, br t, *J* = 6.6 Hz), 3.60 (2H, br t, *J* = 6.5 Hz), 6.77 (2H, br s), 7.48 (2H, d, *J* = 8.6 Hz), 8.22 (2H, d, *J* = 8.6 Hz). ^13^C NMR (75 MHz, DMSO-d_6_): *δ* 24.66, 24.74, 30.9 (3C), 34.5, 45.6, 45.8, 124.8 (2C), 127.5 (2C), 134.4, 153.7, 163.5, 166.9, 169.1. Anal. Calcd for C_17_H_23_N_5_: C, 68.66; H, 7.80; N, 23.55. Found: C, 68.49; H, 7.86; N, 23.37.

### 6-(4-Methoxyphenyl)-4-pyrrolidino-1,3,5-triazine-2-amine (9{1,9})

White solid; yield 50 mg (7%); mp 208–209 °C (EtOH). ^1^H NMR (300 MHz, DMSO-d_6_): *δ* 1.88–1.92 (4H, m), 3.46 (2H, br t, *J* = 6.6 Hz), 3.60 (2H, br t, *J* = 6.5 Hz), 3.82 (3H, s), 6.72 (2H, br s), 7.00 (2H, d, *J* = 9.0 Hz), 8.25 (2H, d, *J* = 8.9 Hz). ^13^C NMR (75 MHz, DMSO-d_6_): *δ* 24.66, 24.74, 45.6, 45.8, 55.2, 113.3 (2C), 129.36 (2C), 129.42, 161.6, 163.4, 166.8, 168.8. Anal. Calcd for C_14_H_17_N_5_O: C, 61.98; H, 6.32; N, 25.81. Found: C, 61.92; H, 6.38; N, 25.73.

### 6-(3-Nitrophenyl)-4-pyrrolidino-1,3,5-triazine-2-amine (9{1,10})

Brownish solid; yield 110 mg (15%); mp 207–208 °C (EtOH). ^1^H NMR (300 MHz, DMSO-d_6_): *δ* 1.90–1.95 (4H, m), 3.49 (2H, br t, *J* = 6.6 Hz), 3.64 (2H, br t, *J* = 6.5 Hz), 7.03 (2H, br s), 7.79 (1H, ddd, *J* = 0.4, 7.8, 8.2 Hz), 8.38 (1H, ddd, *J* = 1.1, 2.5, 8.2 Hz), 8.70 (1H, ddd, *J* = 1.1, 1.5, 7.7 Hz), 9.06 (1H, ddd, *J* = 0.4, 1.6, 2.5 Hz). ^13^C NMR (75 MHz, DMSO-d_6_): *δ* 24.6, 24.7, 45.8, 45.9, 122.0, 125.6, 129.9, 133.7, 138.8, 147.9, 163.3, 166.8, 167.1. Anal. Calcd for C_13_H_14_N_6_O_2_: C, 54.54; H, 4.93; N, 29.36. Found: C, 54.38; H, 5.17; N, 29.12.

### 6-[4-(Trifluoromethyl)phenyl]-4-pyrrolidino-1,3,5-triazine-2-amine (9{1,11})

White solid; yield 125 mg (16%); mp 186–187 °C (EtOH). ^1^H NMR (300 MHz, DMSO-d_6_): *δ* 1.89–1.94 (4H, m), 3.48 (2H, br t, *J* = 6.5 Hz), 3.62 (2H, br t, *J* = 6.6 Hz), 6.95 (2H, br s), 7.85 (2H, d, *J* = 8.2 Hz), 8.48 (2H, d, *J* = 8.0 Hz). ^13^C NMR (75 MHz, DMSO-d_6_): *δ* 24.6, 24.7, 45.7, 45.9, 124.1 (q, *J* = 272.1 Hz), 125.1 (2C, q, *J* = 3.5 Hz), 128.3 (2C), 130.8 (q, *J* = 31.5 Hz), 141.0, 163.4, 166.9, 167.9. Anal. Calcd for C_14_H_14_F_3_N_5_: C, 54.37; H, 4.56; N, 22.64. Found: C, 54.24; H, 4.62; N, 22.51.

### 6-[4-(Trifluoromethoxy)phenyl]-4-pyrrolidino-1,3,5-triazine-2-amine (9{1,12})

White solid; yield 130 mg (16%); mp 149–150 °C (EtOH). ^1^H NMR (300 MHz, DMSO-d_6_): *δ* 1.89–1.93 (4H, m), 3.47 (2H, br t, *J* = 6.5 Hz), 3.61 (2H, br t, *J* = 6.5 Hz), 6.89 (2H, br s), 7.46 (2H, dd, *J* = 1.0, 9.0 Hz), 8.39 (2H, d, *J* = 8.9 Hz). ^13^C NMR (75 MHz, DMSO-d_6_): *δ* 24.6, 24.7, 45.7, 45.8, 119.9 (q, *J* = 256.8 Hz), 120.4 (2C), 129.7 (2C), 136.2, 150.3 (q, *J* = 1.9 Hz), 163.4, 166.8, 167.9. Anal. Calcd for C_14_H_14_F_3_N_5_O: C, 51.69; H, 4.34; N, 21.53. Found: C, 51.55; H, 4.45; N, 21.38.

### 6-(3-Phenoxyphenyl)-4-pyrrolidino-1,3,5-triazine-2-amine (9{1,13})

White solid; yield 202 mg (24%); mp 170–171 °C (EtOH). ^1^H NMR (300 MHz, DMSO-d_6_): *δ* 1.87–1.91 (4H, m), 3.45 (2H, br t, *J* = 6.3 Hz), 3.55 (2H, br t, *J* = 6.2 Hz), 6.84 (2H, br s), 7.04 (2H, dd, *J* = 1.1, 8.7), 7.13–7.20 (2H, m), 7.38–7.44 (2H, m), 7.49 (1H, t, *J* = 7.9 Hz), 7.91 (1H, dd, *J* = 1.4, 2.5 Hz), 8.09 (1H, ddd, *J* = 1.3, 1.3, 7.9 Hz). ^13^C NMR (75 MHz, DMSO-d_6_): *δ* 24.6, 24.7, 45.65, 45.74, 117.6, 118.4 (2C), 121.4, 122.9, 123.4, 129.7, 130.0 (2C), 139.3, 156.5, 156.7, 163.3, 166.8, 168.4. Anal. Calcd for C_19_H_19_N_5_O: C, 68.45; H, 5.74; N, 21.01. Found: C, 68.36; H, 5.87; N, 20.78.

### 6-[4-(Benzyloxy)phenyl]-4-pyrrolidino-1,3,5-triazine-2-amine (9{1,15})

Brownish solid; yield 120 mg (14%); mp 201–202 °C (EtOH). ^1^H NMR (300 MHz, DMSO-d_6_): *δ* 1.87–1.92 (4H, m), 3.46 (2H, br t, *J* = 6.6 Hz), 3.60 (2H, br t, *J* = 6.7 Hz), 5.17 (2H, s), 6.72 (2H, br s), 7.09 (2H, d, *J* = 8.9 Hz), 7.32–7.43 (3H, m), 7.48 (2H, dd, *J* = 1.5, 8.1 Hz), 8.25 (2H, d, *J* = 8.9 Hz). ^13^C NMR (75 MHz, DMSO-d_6_): *δ* 24.6, 24.7, 45.6, 45.7, 69.2, 114.1 (2C), 127.7, 127.8 (2C), 128.3, 129.3 (2C), 129.6 (2C), 136.7, 160.7, 163.4, 166.8, 168.7. Anal. Calcd for C_20_H_21_N_5_O: C, 69.14; H, 6.09; N, 20.16. Found: C, 68.98; H, 6.20; N, 19.92.

### 6-(3,4-Dimethoxyphenyl)-4-pyrrolidino-1,3,5-triazine-2-amine (9{1,16})

Brownish solid; yield 103 mg (14%); mp 217–218 °C (EtOH). ^1^H NMR (300 MHz, DMSO-d_6_): *δ* 1.88–1.92 (4H, m), 3.46 (2H, br t, *J* = 6.5 Hz), 3.61 (2H, br t, *J* = 6.4 Hz), 3.81 (3H, s), 3.82 (3H, s), 6.74 (2H, br s), 7.03 (1H, d, *J* = 8.5 Hz), 7.88 (1H, d, *J* = 1.9 Hz), 7.93 (1H, dd, *J* = 2.0, 8.5 Hz). ^13^C NMR (75 MHz, DMSO-d_6_): *δ* 24.65, 24.73, 45.6, 45.7, 55.3, 55.4, 110.7, 110.8, 121.1, 129.5, 148.1, 151.3, 163.4, 166.8, 168.8. Anal. Calcd for C_15_H_19_N_5_O_2_: C, 59.79; H, 6.36; N, 23.24. Found: C, 59.72; H, 6.47; N, 23.15.

### 6-(3,4,5-Trimethoxyphenyl)-4-pyrrolidino-1,3,5-triazine-2-amine (9{1,17})

Yellowish solid; yield 123 mg (15%); mp 213–214 °C (EtOH). ^1^H NMR (300 MHz, DMSO-d_6_): *δ* 1.88–1.93 (4H, m), 3.46 (2H, br t, *J* = 6.5 Hz), 3.62 (2H, br t, *J* = 6.5 Hz), 3.72 (3H, s), 3.83 (6H, s), 6.81 (2H, br s), 7.65 (2H, s). ^13^C NMR (75 MHz, DMSO-d_6_): *δ* 24.6, 24.7, 45.6, 45.7, 55.7 (2C), 60.0, 104.9 (2C), 132.4, 140.1, 152.4 (2C), 163.3, 166.8, 168.6. Anal. Calcd for C_16_H_21_N_5_O_3_: C, 57.99; H, 6.39; N, 21.13. Found: C, 57.92; H, 6.46; N, 21.03.

### 4-Pyrrolidino-6-(2-thienyl)-1,3,5-triazine-2-amine (9{1,18})

Brownish solid; yield 51 mg (8%); mp 210–211 °C (EtOH). ^1^H NMR (300 MHz, DMSO-d_6_): *δ* 1.87–1.91 (4H, m), 3.45 (2H, br t, *J* = 6.6 Hz), 3.55 (2H, br t, *J* = 6.6 Hz), 6.81 (2H, br s), 7.15 (1H, dd, *J* = 3.7, 5.0 Hz), 7.69 (1H, dd, *J* = 1.3, 5.0 Hz), 7.85 (1H, dd, *J* = 1.3, 3.7 Hz). ^13^C NMR (75 MHz, DMSO-d_6_): *δ* 24.6, 24.7, 45.6, 45.7, 127.8, 128.7, 130.3, 143.0, 163.1, 165.7, 166.5. Anal. Calcd for C_11_H_13_N_5_S: C, 53.42; H, 5.30; N, 28.32. Found: C, 53.29; H, 5.45; N, 28.18.

### 6-(3-Fluorophenyl)-4-piperidino-1,3,5-triazine-2-amine (9{2,2})

White solid; yield 52 mg (8%); mp 138–139 °C (EtOH). ^1^H NMR (300 MHz, DMSO-d_6_): *δ* 1.49–1.69 (6H, m), 3.80 (4H, br s), 6.95 (2H, br s), 7.37 (1H, dddd, *J* = 0.9, 2.8, 8.5, 8.5 Hz), 7.53 (1H, td, *J* = 8.0, 6.0 Hz), 7.98 (1H, ddd, *J* = 1.4, 2.6, 10.6 Hz), 8.13 (1H, ddd, *J* = 1.1, 1.1, 7.8 Hz). ^13^C NMR (75 MHz, DMSO-d_6_): *δ* 24.2, 25.3 (2C), 43.5 (2C), 114.0 (d, *J* = 23.1 Hz), 117.9 (d, *J* = 20.9 Hz), 123.7 (d, *J* = 2.2 Hz), 130.2 (d, *J* = 8.2 Hz), 139.6 (d, *J* = 7.4 Hz), 162.1 (d, *J* = 243.1 Hz), 164.3, 166.8, 168.2. Anal. Calcd for C_14_H_16_FN_5_: C, 61.52; H, 5.90; N, 25.62. Found: C, 61.40; H, 6.07; N, 25.43.

### 6-(4-Fluorophenyl)-4-piperidino-1,3,5-triazine-2-amine (9{2,3})

Yellowish solid; yield 57 mg (8%); mp 156–157 °C (EtOH). ^1^H NMR (300 MHz, DMSO-d_6_): *δ* 1.48–1.68 (6H, m), 3.80 (4H, br s), 6.85 (2H, br s), 7.28 (2H, dd, *J* = 8.9, 8.9 Hz), 8.33 (2H, dd, *J* = 5.8, 9.0 Hz). ^13^C NMR (75 MHz, DMSO-d_6_): *δ* 24.2, 25.3 (2C), 43.4 (2C), 115.0 (d, *J* = 21.6 Hz), 130.0 (d, *J* = 8.9 Hz), 133.5 (d, *J* = 3.0 Hz), 164.0 (d, *J* = 248.1 Hz), 164.4, 167.1, 168.5. Anal. Calcd for C_14_H_16_FN_5_: C, 61.52; H, 5.90; N, 25.62. Found: C, 61.43; H, 6.02; N, 25.51.

### 6-(4-Chlorophenyl)-4-piperidino-1,3,5-triazine-2-amine (9{2,4})

Yellowish solid; yield 75 mg (10%); mp 177–178 °C (EtOH). ^1^H NMR (300 MHz, DMSO-d_6_): *δ* 1.47–1.68 (6H, m), 3.80 (4H, br s), 6.89 (2H, br s), 7.54 (2H, d, *J* = 8.7 Hz), 8.28 (2H, d, *J* = 8.7 Hz). ^13^C NMR (75 MHz, DMSO-d_6_): *δ* 24.2, 25.3 (2C), 43.3 (2C), 128.2 (2C), 129.4 (2C), 135.8, 135.9, 164.3, 167.0, 168.5. Anal. Calcd for C_14_H_16_ClN_5_: C, 58.03; H, 5.57; N, 24.17. Found: C, 57.89; H, 5.71; N, 24.03.

### 6-(4-Methylphenyl)-4-piperidino-1,3,5-triazine-2-amine (9{2,6})

Yellowish solid; yield 79 mg (12%); mp 183–184 °C (EtOH). ^1^H NMR (300 MHz, DMSO-d_6_): *δ* 1.47–1.68 (6H, m), 2.36 (3H, s), 3.79 (4H, br s), 6.79 (2H, br s), 7.27 (2H, d, *J* = 8.0 Hz), 8.18 (2H, d, *J* = 8.2 Hz). ^13^C NMR (75 MHz, DMSO-d_6_): *δ* 20.9, 24.2, 25.3 (2C), 43.3 (2C), 127.7 (2C), 128.6 (2C), 134.3, 140.8, 164.4, 167.1, 169.5. Anal. Calcd for C_14_H_16_N_5_: C, 66.89; H, 7.11; N, 26.00. Found: C, 66.81; H, 7.18; N, 25.92.

### 6-(4-Methoxyphenyl)-4-piperidino-1,3,5-triazine-2-amine (9{2,9})

White solid; yield 72 mg (10%); mp 178–179 °C (EtOH). ^1^H NMR (300 MHz, DMSO-d_6_): *δ* 1.46–1.69 (6H, m), 3.80 (4H, br s), 3.82 (3H, s), 6.76 (2H, br s), 7.00 (2H, d, *J* = 9.0 Hz), 8.24 (2H, d, *J* = 9.0 Hz). ^13^C NMR (75 MHz, DMSO-d_6_): *δ* 24.2, 25.3 (2C), 43.3 (2C), 55.2, 113.3 (2C), 129.3, 129.4 (2C), 161.7, 164.4, 166.9, 169.1. Anal. Calcd for C_15_H_19_N_5_O: C, 63.14; H, 6.71; N, 24.54. Found: C, 63.03; H, 6.82; N, 24.40.

### 6-(3-Nitrophenyl)-4-piperidino-1,3,5-triazine-2-amine (9{2,10})

Brownish solid; yield 91 mg (12%); mp 158–159 °C (EtOH). ^1^H NMR (300 MHz, DMSO-d_6_): *δ* 1.48–1.70 (6H, m), 3.84 (4H, br s), 7.05 (2H, br s), 7.79 (1H, t, *J* = 8.0 Hz), 8.38 (1H, ddd, *J* = 1.0, 2.4, 8.2 Hz), 8.69 (1H, ddd, *J* = 1.3, 1.3, 7.8 Hz), 9.04 (1H, dd, *J* = 1.7, 2.2 Hz). ^13^C NMR (75 MHz, DMSO-d_6_): *δ* 24.2, 25.3 (2C), 43.4 (2C), 122.0, 125.6, 130.0, 133.8, 138.8, 147.9, 164.3, 167.1, 167.6. Anal. Calcd for C_14_H_16_N_6_O_2_: C, 55.99; H, 5.37; N, 27.98. Found: C, 55.74; H, 5.54; N, 27.77.

### 6-[4-(Trifluoromethoxy)phenyl]-4-piperidino-1,3,5-triazine-2-amine (9{2,12})

White solid; yield 43 mg (5%); mp 136–137 °C (EtOH). ^1^H NMR (300 MHz, DMSO-d_6_): *δ* 1.47–1.70 (6H, m), 3.81 (4H, br s), 6.92 (2H, br s), 7.46 (2H, dd, *J* = 0.9, 8.9 Hz), 8.39 (2H, d, *J* = 8.9 Hz). ^13^C NMR (75 MHz, DMSO-d_6_): *δ* 24.2, 25.3 (2C), 43.4 (2C), 119.9 (q, *J* = 257.0 Hz), 120.4 (2C), 129.7 (2C), 136.2, 150.3 (q, *J* = 1.5 Hz), 164.4, 167.1, 168.3. Anal. Calcd for C_15_H_16_F_3_N_5_O: C, 53.10; H, 4.75; N, 20.64. Found: C, 52.97; H, 4.84; N, 20.50.

### 4-Piperidino-6-(3-phenoxyphenyl)-1,3,5-triazine-2-amine (9{2,13})

Yellowish solid; yield 162 mg (19%); mp 125–126 °C (EtOH). ^1^H NMR (300 MHz, DMSO-d_6_): *δ* 1.45–1.67 (6H, m), 3.77 (4H, s), 6.87 (2H, br s), 7.05 (2H, dd, *J* = 1.1, 8.7), 7.13–7.21 (2H, m), 7.38–7.45 (2H, m), 7.50 (1H, t, *J* = 7.9 Hz), 7.91 (1H, dd, *J* = 1.5, 2.5 Hz), 8.09 (1H, ddd, *J* = 1.3, 1.3, 7.9 Hz). ^13^C NMR (75 MHz, DMSO-d_6_): *δ* 24.2, 25.3 (2C), 43.3 (2C), 117.6, 118.5 (2C), 121.4, 122.8, 123.4, 129.8, 130.0 (2C), 139.3, 156.5, 156.6, 164.3, 167.1, 168.9. Anal. Calcd for C_20_H_21_N_5_O: C, 69.14; H, 6.09; N, 20.16. Found: C, 68.96; H, 6.30; N, 19.98.

### 6-[3-(Benzyloxy)phenyl]-4-piperidino-1,3,5-triazine-2-amine (9{2,14})

White solid; yield 196 mg (22%); mp 157–158 °C (EtOH). ^1^H NMR (300 MHz, DMSO-d_6_): *δ* 1.46–1.69 (6H, m), 3.79 (4H, br s), 5.16 (2H, s), 6.83 (2H, br s), 7.17 (1H, ddd, *J* = 1.1, 8.3, 2.5 Hz), 7.30–7.44 (4H, m), 7.48 (2H, d, *J* = 7.6 Hz), 7.85–7.90 (2H, m). ^13^C NMR (75 MHz, DMSO-d_6_): *δ* 24.2, 25.3 (2C), 43.3 (2C), 69.2, 113.8, 117.5, 120.2, 127.6 (2C), 127.7, 128.4 (2C), 129.1, 137.0, 138.6, 158.2, 164.4, 167.1, 169.3. Anal. Calcd for C_21_H_23_N_5_O: C, 69.78; H, 6.41; N, 19.38. Found: C, 69.64; H, 6.55; N, 19.21.

### 6-(3-Fluorophenyl)-4-(4-methylpiperidino)-1,3,5-triazine-2-amine (9{3,2})

White solid; yield 58 mg (9%); mp 125–126 °C (EtOH). ^1^H NMR (300 MHz, DMSO-d_6_): *δ* 0.93 (3H, d, *J* = 6.2 Hz), 1.01–1.13 (2H, m), 1.56–1.75 (3H, m), 2.86 (2H, t, *J* = 11.7 Hz), 4.69 (1H, br s), 4.81 (1H, br s), 6.93 (2H, br s), 7.37 (1H, dddd, *J* = 0.9, 2.7, 8.5, 8.5 Hz), 7.52 (1H, td, *J* = 8.0, 6.0 Hz), 7.98 (1H, ddd, *J* = 1.4, 2.7, 10.6 Hz), 8.12 (1H, ddd, *J* = 1.2, 1.2, 7.8 Hz). ^13^C NMR (75 MHz, DMSO-d_6_): *δ* 21.7, 30.5 (2C), 33.5, 42.7 (2C), 113.9 (d, *J* = 22.1 Hz), 117.8 (d, *J* = 20.9 Hz), 123.7 (d, *J* = 2.2 Hz), 130.2 (d, *J* = 7.5 Hz), 139.8 (d, *J* = 7.5 Hz), 162.1 (d, *J* = 242.5 Hz), 164.4, 167.1, 168.4. Anal. Calcd for C_15_H_18_FN_5_: C, 62.70; H, 6.31; N, 24.37. Found: C, 62.59; H, 6.43; N, 24.22.

### 6-(4-Fluorophenyl)-4-(4-methylpiperidino)-1,3,5-triazine-2-amine (9{3,3})

White solid; yield 13 mg (2%); mp > 300 °C (EtOH). ^1^H NMR (300 MHz, DMSO-d_6_): *δ* 0.93 (3H, d, *J* = 6.2 Hz), 0.96–1.12 (2H, m), 1.56–1.76 (3H, m), 2.85 (2H, t, *J* = 12.4 Hz), 4.72 (1H, br s), 4.80 (1H, br s), 6.86 (2H, br s), 7.29 (2H, dd, *J* = 8.9, 8.9 Hz), 8.33 (2H, dd, *J* = 5.8, 9.0 Hz). ^13^C NMR (75 MHz, DMSO-d_6_): *δ* 21.7, 30.5 (2C), 33.5, 42.7 (2C), 115.0 (2C, d, *J* = 21.6 Hz), 130.1 (2C, d, *J* = 8.8 Hz), 133.5 (d, *J* = 2.2 Hz), 164.0 (d, *J* = 247.4 Hz), 164.4, 167.1, 168.6. Anal. Calcd for C_15_H_18_FN_5_: C, 62.70; H, 6.31; N, 24.37. Found: C, 62.63; H, 6.38; N, 24.29.

### 6-(4-Chlorophenyl)-4-(4-methylpiperidino)-1,3,5-triazine-2-amine (9{3,4})

Yellowish solid; yield 104 mg (14%); mp 168–169 °C (EtOH). ^1^H NMR (300 MHz, DMSO-d_6_): *δ* 0.92 (3H, d, *J* = 6.2 Hz), 1.00–1.12 (2H, m), 1.57–1.74 (3H, m), 2.86 (2H, t, *J* = 12.3 Hz), 4.73 (2H, br s), 6.89 (2H, br s), 7.53 (2H, d, *J* = 8.8 Hz), 8.28 (2H, d, *J* = 8.8 Hz). ^13^C NMR (75 MHz, DMSO-d_6_): *δ* 21.7, 30.6 (2C), 33.5, 42.7 (2C), 128.2 (2C), 129.4 (2C), 135.8, 135.9, 164.4, 167.1, 168.6. Anal. Calcd for C_15_H_18_ClN_5_: C, 59.30; H, 5.97; N, 23.05. Found: C, 59.19; H, 6.06; N, 22.98.

### 6-(4-Methylphenyl)-4-(4-methylpiperidino)-1,3,5-triazine-2-amine (9{3,6})

White solid; yield 102 mg (14%); mp > 300 °C (EtOH). ^1^H NMR (300 MHz, DMSO-d_6_): *δ* 0.93 (3H, d, *J* = 6.5 Hz), 1.00–1.11 (2H, m), 1.57–1.73 (3H, m), 2.36 (3H, s), 2.84 (2H, t, *J* = 12.2 Hz), 4.77 (2H, br s), 6.80 (2H, br s), 7.27 (2H, d, *J* = 8.0 Hz), 8.18 (2H, d, *J* = 8.2 Hz). ^13^C NMR (75 MHz, DMSO-d_6_): *δ* 21.0, 21.7, 30.6 (2C), 33.5, 42.7 (2C), 127.7 (2C), 128.6 (2C), 134.3, 140.8, 164.5, 167.1, 169.5. Anal. Calcd for C_16_H_21_N_5_: C, 67.82; H, 7.47; N, 24.71. Found: C, 67.69; H, 7.63; N, 24.58.

### 6-[4-(*N*,*N*-Dimethylamino)phenyl]-4-(4-methylpiperidino)-1,3,5-triazine-2-amine (9{3,8})

White solid; yield 23 mg (3%); mp 212–213 °C (EtOH). ^1^H NMR (300 MHz, DMSO-d_6_): *δ* 0.92 (3H, d, *J* = 6.2 Hz), 1.00–1.10 (2H, m), 1.55–1.73 (3H, m), 2.82 (2H, t, *J* = 12.1 Hz), 2.98 (6H, s), 4.74 (2H, br s), 6.61 (2H, br s), 6.72 (2H, d, *J* = 9.1 Hz), 8.13 (2H, d, *J* = 9.0 Hz). ^13^C NMR (75 MHz, DMSO-d_6_): *δ* 21.7, 30.6 (2C), 33.6, 40.4 (2C), 42.6 (2C), 110.8 (2C), 124.0, 129.1 (2C), 152.2, 164.4, 167.0, 169.6. Anal. Calcd for C_17_H_24_N_6_: C, 65.36; H, 7.74; N, 26.90. Found: C, 65.18; H, 7.96; N, 26.68.

### 6-(4-Methoxyphenyl)-4-(4-methylpiperidino)-1,3,5-triazine-2-amine (9{3,9})

White solid; yield 62 mg (8%); mp 172–173 °C (EtOH). ^1^H NMR (300 MHz, DMSO-d_6_): *δ* 0.92 (3H, d, *J* = 6.1 Hz), 1.00–1.11 (2H, m), 1.57–1.74 (3H, m), 2.84 (2H, t, *J* = 12.4 Hz), 3.82 (3H, s), 4.75 (2H, br s), 6.74 (2H, br s), 7.00 (2H, d, *J* = 9.0 Hz), 8.24 (2H, d, *J* = 8.8 Hz). ^13^C NMR (75 MHz, DMSO-d_6_): *δ* 21.7, 30.6 (2C), 33.6, 42.7 (2C), 55.2, 113.4 (2C), 129.4 (3C), 161.7, 164.4, 167.1, 169.2. Anal. Calcd for C_16_H_21_N_5_O: C, 64.19; H, 7.07; N, 23.39. Found: C, 64.11; H, 7.15; N, 23.30.

### 4-(4-Methylpiperidino)-6-(3-nitrophenyl)-1,3,5-triazine-2-amine (9{3,10})

Brownish solid; yield 89 mg (11%); mp 153–154 °C (EtOH). ^1^H NMR (300 MHz, DMSO-d_6_): *δ* 0.93 (3H, d, *J* = 6.2 Hz), 0.98–1.14 (2H, m), 1.58–1.78 (3H, m), 2.89 (2H, br s), 4.70 (1H, br s), 4.83 (1H, br s), 7.04 (1H, br s), 7.10 (1H, br s), 7.79 (1H, t, *J* = 8.0 Hz), 8.38 (1H, ddd, *J* = 1.1, 2.5, 8.2 Hz), 8.69 (1H, ddd, *J* = 1.3, 1.3, 7.9 Hz), 9.04 (1H, dd, *J* = 1.6, 2.1 Hz). ^13^C NMR (75 MHz, DMSO-d_6_): *δ* 21.6, 30.5 (2C), 33.5, 42.8 (2C), 122.0, 125.6, 129.9, 133.8, 138.8, 147.9, 164.4, 167.1, 167.6. Anal. Calcd for C_15_H_18_N_6_O_2_: C, 57.31; H, 5.77; N, 26.74. Found: C, 57.16; H, 5.94; N, 26.54.

### 6-[4-(Trifluoromethyl)phenyl]-4-(4-methylpiperidino)-1,3,5-triazine-2-amine (9{3,11})

White solid; yield 15 mg (2%); mp 170–171 °C (EtOH). ^1^H NMR (300 MHz, DMSO-d_6_): *δ* 0.93 (3H, d, *J* = 6.2 Hz), 0.97–1.14 (2H, m), 1.58–1.76 (3H, m), 2.88 (2H, t, *J* = 11.8 Hz), 4.71 (1H, br s), 4.82 (1H, br s), 6.97 (2H, br s), 7.85 (2H, d, *J* = 8.6 Hz), 8.46 (2H, d, *J* = 7.8 Hz). ^13^C NMR (75 MHz, DMSO-d_6_): *δ* 21.6, 30.5 (2C), 33.5, 42.8 (2C), 124.1 (q, *J* = 272.2 Hz), 125.1 (2C, q, *J* = 4.0 Hz), 128.3 (2C), 130.8 (q, *J* = 31.8 Hz), 141.0 (q, *J* = 1.5 Hz), 164.4, 167.1, 168.3. Anal. Calcd for C_16_H_18_F_3_N_5_: C, 56.97; H, 5.38; N, 20.76. Found: C, 56.84; H, 5.54; N, 20.63.

### 6-[4-(Trifluoromethoxy)phenyl]-4-(4-methylpiperidino)-1,3,5-triazine-2-amine (9{3,12})

White solid; yield 108 mg (12%); mp 152–153 °C (EtOH). ^1^H NMR (300 MHz, DMSO-d_6_): *δ* 0.93 (3H, d, *J* = 6.1 Hz), 0.96–1.13 (2H, m), 1.56–1.75 (3H, m), 2.86 (2H, t, *J* = 11.7 Hz), 4.70 (1H, br s), 4.82 (1H, br s), 6.93 (2H, br s), 7.46 (2H, dd, *J* = 0.9, 8.9 Hz), 8.39 (2H, d, *J* = 8.9 Hz). ^13^C NMR (75 MHz, DMSO-d_6_): *δ* 21.7, 30.6 (2C), 33.5, 42.7 (2C), 120.0 (q, *J* = 256.8 Hz), 120.4 (2C), 129.7 (2C), 136.2, 150.3 (q, *J* = 1.7 Hz), 164.4, 167.1, 168.4. Anal. Calcd for C_16_H_18_F_3_N_5_O: C, 54.39; H, 5.13; N, 19.82. Found: C, 54.30; H, 5.26; N, 19.71.

### 6-(3,4,5-Trimethoxyphenyl)-4-(4-methylpiperidino)-1,3,5-triazine-2-amine (9{3,17})

White solid; yield 118 mg (13%); mp 153–154 °C (EtOH). ^1^H NMR (300 MHz, DMSO-d_6_): *δ* 0.93 (3H, d, *J* = 6.2 Hz), 0.96–1.13 (2H, m), 1.56–1.75 (3H, m), 2.86 (2H, t, *J* = 11.9 Hz), 3.73 (3H, s), 3.84 (6H, s), 4.76 (2H, br s), 6.86 (2H, br s), 7.62 (2H, s). ^13^C NMR (75 MHz, DMSO-d_6_): *δ* 21.7, 30.6 (2C), 33.6, 42.8 (2C), 55.8 (2C), 60.0, 105.0 (2C), 132.4, 140.2, 152.5 (2C), 164.3, 166.9, 169.0. Anal. Calcd for C_18_H_25_N_5_O: C, 60.15; H, 7.01; N, 19.49. Found: C, 60.06; H, 7.09; N, 19.42.

### 4-(4-Methylpiperidino)-6-(2-thienyl)-1,3,5-triazine-2-amine (9{3,18})

Brownish solid; yield 90 mg (13%); mp 155–156 °C (EtOH). ^1^H NMR (300 MHz, DMSO-d_6_): *δ* 0.92 (3H, d, *J* = 6.0 Hz), 0.98–1.10 (2H, m), 1.56–1.73 (3H, m), 2.83 (2H, t, *J* = 12.4 Hz), 4.69 (2H, br s), 6.84 (2H, br s), 7.15 (1H, dd, *J* = 3.7, 5.0 Hz), 7.70 (1H, dd, *J* = 1.3, 5.0 Hz), 7.85 (1H, dd, *J* = 1.3, 3.7 Hz). ^13^C NMR (75 MHz, DMSO-d_6_): *δ* 21.6, 30.5 (2C), 33.5, 42.6 (2C), 127.8, 128.8, 130.3, 143.0, 164.0, 166.1, 166.8. Anal. Calcd for C_13_H_17_N_5_S: C, 56.70; H, 6.22; N, 25.43. Found: C, 56.58; H, 6.34; N, 25.29.

### 6-(3-Fluorophenyl)-4-(4-methylpiperazino)-1,3,5-triazine-2-amine (9{4,2})

White solid, yield 120 mg (17%); mp 187–188 °C (EtOH). ^1^H NMR (300 MHz, DMSO-d_6_): *δ* 2.22 (3H, s), 2.32–2.39 (4H, m), 3.81 (4H, br s), 6.99 (2H, br s), 7.37 (1H, dddd, *J* = 0.8, 2.8, 8.5, 8.5 Hz), 7.53 (1H, td, *J* = 8.0, 6.0 Hz), 7.99 (1H, ddd, *J* = 1.3, 2.6, 10.5 Hz), 8.14 (1H, ddd, *J* = 1.1, 1.1, 7.8 Hz). ^13^C NMR (75 MHz, DMSO-d_6_): *δ* 42.5, 45.7 (2C), 54.3 (2C), 114.0 (d, *J* = 22.9 Hz), 117.9 (d, *J* = 20.9 Hz), 123.7 (d, *J* = 2.2 Hz), 130.2 (d, *J* = 8.2 Hz), 139.6 (d, *J* = 7.5 Hz), 162.1 (d, *J* = 242.9 Hz), 164.6, 167.1, 168.4. Anal. Calcd for C_14_H_17_FN_6_: C, 58.32; H, 5.94; N, 29.15. Found: C, 58.19; H, 6.11; N, 28.92.

### 6-(4-Fluorophenyl)-4-(4-methylpiperazino)-1,3,5-triazine-2-amine (9{4,3})

White solid; yield 211 mg (29%); mp 192–193 °C (EtOH) (Lit.^19^ mp 198–200 °C). ^1^H NMR (300 MHz, DMSO-d_6_): *δ* 2.21 (3H, s), 2.32–2.38 (4H, m), 3.80 (4H, br s), 6.92 (2H, br s), 7.29 (2H, dd, *J* = 8.9, 8.9 Hz), 8.34 (2H, dd, *J* = 5.8, 9.0 Hz). ^13^C NMR (75 MHz, DMSO-d_6_): *δ* 42.4, 45.7 (2C), 54.3 (2C), 115.0 (2C, d, *J* = 21.6 Hz), 130.1 (2C, d, *J* = 8.9 Hz), 133.4 (d, *J* = 2.8 Hz), 164.1 (d, *J* = 248.3 Hz), 164.6, 167.1, 168.6. Anal. Calcd for C_14_H_17_FN_6_: C, 58.32; H, 5.94; N, 29.15. Found: C, 58.23; H, 6.07; N, 29.03.

### 6-(4-Chlorophenyl)-4-(4-methylpiperazino)-1,3,5-triazine-2-amine (9{4,4})

White solid, yield 284 mg (37%); mp 222–223 °C (EtOH) (Lit.^19^ mp 229–231 °C). ^1^H NMR (300 MHz, DMSO-d_6_): *δ* 2.2 (3H, s), 2.31–2.38 (4H, m), 3.80 (4H, br s), 6.95 (2H, br s), 7.53 (2H, d, *J* = 8.6 Hz), 8.29 (2H, d, *J* = 8.7 Hz). ^13^C NMR (75 MHz, DMSO-d_6_): *δ* 42.4, 45.7 (2C), 54.3 (2C), 128.2 (2C), 129.4 (2C), 135.8, 135.9, 164.6, 167.0, 168.6. Anal. Calcd for C_14_H_17_ClN_6_: C, 55.17; H, 5.62; N, 27.57. Found: C, 55.06; H, 5.75; N, 27.44.

### 6-(3-Methylphenyl)-4-(4-methylpiperazino)-1,3,5-triazine-2-amine (9{4,5})

White solid, yield 67 mg (9%); mp 194–195 °C (EtOH) (Lit.^19^ mp 187–190 °C). ^1^H NMR (300 MHz, DMSO-d_6_): *δ* 2.22 (3H, s), 2.33–2.40 (7H, m), 3.81 (4H, br s), 6.90 (2H, br s), 7.31–7.38 (2H, m), 8.07–8.13 (2H, m). ^13^C NMR (75 MHz, DMSO-d_6_): *δ* 21.0, 42.4, 45.7 (2C), 54.3 (2C), 125.0, 128.0, 128.2, 131.7, 136.9, 137.1, 164.7, 167.1, 169.7. Anal. Calcd for C_15_H_20_N_6_: C, 63.36; H, 7.09; N, 29.55. Found: C, 63.22; H, 7.20; N, 29.44.

### 6-(4-Methylphenyl)-4-(4-methylpiperazino)-1,3,5-triazine-2-amine (9{4,6})

White solid; yield 235 mg (33%); mp 220–221 °C (EtOH) (Lit.^19^ mp 216–219 °C). ^1^H NMR (300 MHz, DMSO-d_6_): *δ* 2.21 (3H, s), 2.31–2.38 (7H, m), 3.80 (4H, br s), 6.84 (2H, br s), 7.27 (2H, d, *J* = 8.0 Hz), 8.18 (2H, d, *J* = 8.1 Hz). ^13^C NMR (75 MHz, DMSO-d_6_): *δ* 21.0, 42.4, 45.7 (2C), 54.3 (2C), 127.7 (2C), 128.7 (2C), 134.2, 140.9, 164.7, 167.1, 169.6. Anal. Calcd for C_15_H_20_N_6_: C, 63.36; H, 7.09; N, 29.55. Found: C, 63.28; H, 7.14; N, 29.47.

### 6-[4-(*tert*-Butyl)phenyl]-4-(4-methylpiperazino)-1,3,5-triazine-2-amine (9{4,7})

White solid, yield 65 mg (8%); mp 228–229 °C (EtOH). ^1^H NMR (300 MHz, DMSO-d_6_): *δ* 1.31 (9H, s), 2.21 (3H, s), 2.31–2.39 (4H, m), 3.80 (4H, br s), 6.88 (2H, br s), 7.48 (2H, d, *J* = 8.6 Hz), 8.21 (2H, d, *J* = 8.6 Hz). ^13^C NMR (75 MHz, DMSO-d_6_): *δ* 30.9 (3C), 34.5, 42.4, 45.7 (2C), 54.3 (2C), 124.8 (2C), 127.6 (2C), 134.2, 153.9, 164.7, 167.1, 169.6. Anal. Calcd for C_15_H_20_N_6_: C, 66.23; H, 8.03; N, 25.74. Found: C, 66.07; H, 8.20; N, 25.56.

### 6-[4-(*N*,*N*-Dimethylamino)phenyl]-4-(4-methylpiperazino)-1,3,5-triazine-2-amine (9{4,8})

White solid, yield 84 mg (11%); mp 244–245 °C (EtOH) (Lit.^19^ mp 253–256 °C). ^1^H NMR (300 MHz, DMSO-d_6_): *δ* 2.21 (3H, s), 2.30–2.37 (4H, m), 2.98 (6H, s), 3.78 (4H, br s), 6.66 (2H, br s), 6.72 (2H, d, *J* = 9.1 Hz), 8.13 (2H, d, *J* = 8.9 Hz). ^13^C NMR (75 MHz, DMSO-d_6_): *δ* 39.7, 42.3 (2C), 45.7 (2C), 54.4 (2C), 110.8 (2C), 123.8, 129.1 (2C), 152.3, 164.7, 167.0, 169.6. Anal. Calcd for C_16_H_23_N_7_: C, 61.32; H, 7.40; N, 31.28. Found: C, 61.13; H, 7.52; N, 31.18.

### 6-(4-Methoxyphenyl)-4-(4-methylpiperazino)-1,3,5-triazine-2-amine (9{4,9})

White solid, yield 32 mg (4%); mp 196–197 °C (EtOH). ^1^H NMR (300 MHz, DMSO-d_6_): *δ* 2.21 (3H, s), 2.30–2.38 (4H, m), 3.80 (4H, br s), 3.82 (3H, s), 6.82 (2H, br s), 7.00 (2H, d, *J* = 8.9 Hz), 8.25 (2H, d, *J* = 8.9 Hz). ^13^C NMR (75 MHz, DMSO-d_6_): *δ* 42.4, 45.7 (2C), 54.3, 55.2 (2C), 113.3 (2C), 129.2, 129.4 (2C), 161.7, 164.6, 167.0, 169.2. Anal. Calcd for C_15_H_20_N_6_O: C, 59.98; H, 6.71; N, 27.98. Found: C, 59.93; H, 6.76; N, 27.92.

### 4-(4-Methylpiperazino)-6-(3-nitrophenyl)-1,3,5-triazine-2-amine (9{4,10})

Yellowish solid, yield 89 mg (11%); mp 226–227 °C (EtOH). ^1^H NMR (300 MHz, DMSO-d_6_): *δ* 2.22 (3H, s), 2.34–2.41 (4H, m), 3.81 (2H, br s), 3.85 (2H, br s) 7.07 (1H, br s), 7.17 (1H, br s), 7.79 (1H, t, *J* = 8.0 Hz), 8.39 (1H, ddd, *J* = 1.1, 2.5, 8.2 Hz), 8.70 (1H, ddd, *J* = 1.3, 1.3, 7.8 Hz), 9.04 (1H, dd, *J* = 1.7, 2.1 Hz). ^13^C NMR (75 MHz, DMSO-d_6_): *δ* 42.5, 45.7 (2C), 54.3 (2C), 122.0, 125.7, 129.9, 133.8, 138.6, 147.9, 164.6, 167.0, 167.6. Anal. Calcd for C_14_H_17_N_7_O_2_: C, 53.33; H, 5.43; N, 31.09. Found: C, 53.11; H, 5.59; N, 30.92.

### 6-[4-(Trifluoromethyl)phenyl]-4-(4-methylpiperazino)-1,3,5-triazine-2-amine (9{4,11})

White solid, yield 238 mg (28%); mp 194–195 °C (EtOH) (Lit.^19^ mp 204–205 °C). ^1^H NMR (300 MHz, DMSO-d_6_): *δ* 2.22 (3H, s), 2.33–2.40 (4H, m), 3.83 (4H, br s), 7.07 (2H, br s), 7.85 (2H, d, *J* = 8.3 Hz), 8.48 (2H, d, *J* = 8.3 Hz). ^13^C NMR (75 MHz, DMSO-d_6_): *δ* 42.5, 45.7 (2C), 54.3 (2C), 124.1 (q, *J* = 272.4 Hz), 125.1 (2C, q, *J* = 3.5 Hz), 128.4 (2C), 131.0 (q, *J* = 31.7 Hz), 140.9 (q, *J* = 1.5 Hz), 164.7, 167.2, 168.4. Anal. Calcd for C_15_H_17_F_3_N_6_: C, 53.25; H, 5.06; N, 24.84. Found: C, 53.07; H, 5.19; N, 24.68.

### 6-[4-(Trifluoromethoxy)phenyl]-4-(4-methylpiperazino)-1,3,5-triazine-2-amine (9{4,12})

White solid, yield 88 mg (10%); mp 194–195 °C (EtOH). ^1^H NMR (300 MHz, DMSO-d_6_): *δ* 2.22 (3H, s), 2.31–2.40 (4H, m), 3.81 (4H, br s), 7.00 (2H, br s), 7.46 (2H, dd, *J* = 0.8, 8.9 Hz), 8.40 (2H, d, *J* = 8.9 Hz). ^13^C NMR (75 MHz, DMSO-d_6_): *δ* 42.5, 45.7 (2C), 54.3 (2C), 119.9 (q, *J* = 257.1 Hz), 120.4 (2C), 129.8 (2C), 136.0, 150.4 (q, *J* = 2.0 Hz), 164.6, 167.1, 168.4. Anal. Calcd for C_15_H_17_F_3_N_6_O: C, 50.85; H, 4.84; N, 23.72. Found: C, 50.76; H, 4.93; N, 23.58.

### 6-[3-(Benzyloxy)phenyl]-4-(4-methylpiperazino)-1,3,5-triazine-2-amine (9{4,14})

White solid; yield 112 mg (12%); mp 117–118 °C (EtOH). ^1^H NMR (300 MHz, DMSO-d_6_): *δ* 2.22 (3H, s), 2.31–2.38 (4H, m), 3.78 (4H, br s), 5.17 (2H, s), 6.90 (2H, br s), 7.17 (1H, ddd, *J* = 1.2, 2.0, 8.1 Hz), 7.30–7.44 (4H, m), 7.48 (2H, d, *J* = 7.5 Hz), 7.86–7.91 (2H, m). ^13^C NMR (75 MHz, DMSO-d_6_): *δ* 42.4, 45.7 (2C), 54.3 (2C), 69.2, 113.9, 117.6, 120.3, 127.6, 127.7 (2C), 128.4 (2C), 129.2, 137.0, 138.4, 158.2, 164.6, 167.1, 169.3. Anal. Calcd for C_21_H_24_N_6_O: C, 67.00; H, 6.43; N, 22.32. Found: C, 66.87; H, 6.57; N, 22.19.

### 6-[4-(Benzyloxy)phenyl]-4-(4-methylpiperazino)-1,3,5-triazine-2-amine (9{4,15})

White solid; yield 385 mg (41%); mp 191–192 °C (EtOH). ^1^H NMR (300 MHz, DMSO-d_6_): *δ* 2.21 (3H, s), 2.31–2.37 (4H, m), 3.79 (4H, br s), 5.17 (2H, s), 6.81 (2H, br s), 7.09 (2H, d, *J* = 9.0 Hz), 7.32–7.44 (3H, m), 7.47 (2H, d, *J* = 7.6 Hz), 8.24 (2H, d, *J* = 8.9 Hz). ^13^C NMR (75 MHz, DMSO-d_6_): *δ* 42.4, 45.7 (2C), 54.3 (2C), 69.2, 114.2 (2C), 127.7 (2C), 127.8 (3C), 128.3, 129.4 (2C), 136.7, 160.8, 164.6, 167.0, 169.2. Anal. Calcd for C_21_H_24_N_6_O: C, 67.00; H, 6.43; N, 22.32. Found: C, 66.89; H, 6.58; N, 22.20.

### 6-(3,4-Dimethoxyphenyl)-4-(4-methylpiperazino)-1,3,5-triazine-2-amine (9{4,16})

White solid; yield 319 mg (39%); mp 223–224 °C (EtOH). ^1^H NMR (300 MHz, DMSO-d_6_): *δ* 2.21 (3H, s), 2.31–2.38 (4H, m), 3.79 (4H, br s), 3.81 (3H, s), 3.82 (3H, s), 6.83 (2H, br s), 7.03 (1H, d, *J* = 8.6 Hz), 7.84 (1H, d, *J* = 1.9 Hz), 7.92 (1H, dd, *J* = 2.0, 8.5 Hz). ^13^C NMR (75 MHz, DMSO-d_6_): *δ* 42.4, 45.7 (2C), 54.3 (2C), 55.3, 55.4, 110.7, 110.9, 121.2, 129.4, 148.1, 151.5, 164.6, 167.0, 169.3. Anal. Calcd for C_16_H_22_N_6_O_2_: C, 58.17; H, 6.71; N, 25.44. Found: C, 58.04; H, 6.85; N, 25.30.

### 6-(3,4,5-Trimethoxyphenyl)-4-(4-methylpiperazino)-1,3,5-triazine-2-amine (9{4,17})

White solid, yield 318 mg (35%); mp 234–235 °C (EtOH). ^1^H NMR (300 MHz, DMSO-d_6_): *δ* 2.23 (3H, s), 2.34–2.41 (4H, m), 3.73 (3H, s) 3.82 (4H, br s), 3.84 (6H, s), 6.89 (2H, br s), 7.62 (2H, s). ^13^C NMR (75 MHz, DMSO-d_6_): *δ* 42.4, 45.6 (2C), 54.3 (2C), 55.8 (2C), 60.0, 105.0 (2C), 132.3, 140.2, 152.5 (2C), 164.6, 167.0, 169.2. Anal. Calcd for C_17_H_24_N_6_O_3_: C, 56.65; H, 6.71; N, 23.32. Found: C, 56.57; H, 6.78; N, 23.24.

### 6-(3-Fluorophenyl)-4-(3,4-dihydroisoquinolin-2(1*H*)-yl)-1,3,5-triazine-2-amine (9{6,2})

White solid; yield 174 mg (22%); mp 148–149 °C (EtOH). ^1^H NMR (300 MHz, DMSO-d_6_): *δ* 2.89 (2H, br t, *J* = 5.5 Hz), 4.00 (1H, br s), 4.11 (1H, br s), 4.89 (1H, br s), 5.02 (1H, br s), 7.04 (2H, br s), 7.15–7.33 (4H, m), 7.38 (1H, dddd, *J* = 0.9, 2.7, 8.5, 8.5 Hz), 7.55 (1H, td, *J* = 8.0, 6.0 Hz), 8.06 (1H, d, *J* = 10.0 Hz), 8.19 (1H, d, *J* = 7.8 Hz). ^13^C NMR (75 MHz, DMSO-d_6_): *δ* 28.1, 40.4, 44.9, 114.1 (d, *J* = 22.9 Hz), 118.0 (d, *J* = 21.2 Hz), 123.8 (d, *J* = 2.2 Hz), 126.0, 126.2, 126.3, 128.5, 130.2 (d, *J* = 8.2 Hz), 133.5*, 133.8*, 134.7, 139.7 (d, *J* = 7.6 Hz), 162.1 (d, *J* = 242.8 Hz), 164.7, 167.1, 168.5 (d, *J* = 3.0 Hz); * – signals of two conformers. Anal. Calcd for C_18_H_16_FN_5_: C, 67.28; H, 5.02; N, 21.79. Found: C, 67.14; H, 5.15; N, 21.63.

### 6-(4-Chlorophenyl)-4-(3,4-dihydroisoquinolin-2(1*H*)-yl)-1,3,5-triazine-2-amine (9{6,4})

Brownish solid; yield 317 mg (38%); mp 183–184 °C (EtOH). ^1^H NMR (300 MHz, DMSO-d_6_): *δ* 2.88 (2H, br t, *J* = 5.3 Hz), 4.00 (1H, br s), 4.11 (1H, br s), 4.88 (1H, br s), 5.01 (1H, br s), 7.03 (2H, br s), 7.15–7.32 (4H, m), 7.56 (2H, d, *J* = 8.6 Hz), 8.34 (2H, d, *J* = 8.6 Hz). ^13^C NMR (75 MHz, DMSO-d_6_): *δ* 28.0, 40.4, 44.9, 126.0, 126.2, 126.3, 128.2 (2C), 128.5, 129.5 (2C), 133.6*, 133.7*, 134.7, 135.8, 135.9, 164.7, 167.0, 168.6; * – signals of two conformers. Anal. Calcd for C_18_H_16_ClN_5_: C, 64.00; H, 4.77; N, 20.73. Found: C, 63.87; H, 4.91; N, 20.59.

### 6-(3-Methylphenyl)-4-(3,4-dihydroisoquinolin-2(1*H*)-yl)-1,3,5-triazine-2-amine (9{6,5})

Yellowish solid; yield 302 mg (38%); mp 145–146 °C (EtOH). ^1^H NMR (300 MHz, DMSO-d_6_): *δ* 2.39 (3H, s), 2.88 (2H, br t, *J* = 5.5 Hz), 4.02 (1H, br s), 4.09 (1H, br s), 4.89 (1H, br s), 5.01 (1H, br s), 6.94 (2H, br s), 7.16–7.31 (4H, m), 7.31–7.41 (2H, m), 8.12–8.19 (2H, m). ^13^C NMR (75 MHz, DMSO-d_6_): *δ* 21.0, 28.1, 40.3, 44.9, 125.0, 126.0, 126.2, 126.3, 128.0, 128.2, 128.5, 131.7, 133.7 (br s), 134.7, 136.9, 137.1, 164.8, 167.1, 169.7. Anal. Calcd for C_19_H_19_N_5_: C, 71.90; H, 6.03; N, 22.07. Found: C, 71.75; H, 6.16; N, 21.89.

### 6-(4-Methylphenyl)-4-(3,4-dihydroisoquinolin-2(1*H*)-yl)-1,3,5-triazine-2-amine (9{6,6})

Brownish solid; yield 433 mg (54%); mp 166–167 °C (EtOH). ^1^H NMR (300 MHz, DMSO-d_6_): *δ* 2.38 (3H, s), 2.87 (2H, br t, *J* = 5.7 Hz), 4.02 (1H, br s), 4.09 (1H, br s), 4.90 (1H, br s), 5.00 (1H, br s), 6.92 (2H, br s), 7.16–7.24 (4H, m), 7.29 (2H, d, *J* = 8.0 Hz), 8.26 (2H, d, *J* = 8.1 Hz). ^13^C NMR (75 MHz, DMSO-d_6_): *δ* 21.0, 28.1, 40.4, 44.9, 126.0, 126.2, 126.3, 127.8 (2C), 128.5, 128.7 (2C), 133.8 (br s), 134.3, 134.8, 141.0, 164.8, 167.1, 169.6. Anal. Calcd for C_19_H_19_N_5_: C, 71.90; H, 6.03; N, 22.07. Found: C, 71.79; H, 6.10; N, 21.93.

### 6-[4-(*tert*-Butyl)phenyl]-4-(3,4-dihydroisoquinolin-2(1*H*)-yl)-1,3,5-triazine-2-amine (9{6,7})

White solid; yield 499 mg (49%); mp 162–164 °C (EtOH). ^1^H NMR (300 MHz, DMSO-d_6_): *δ* 1.32 (9H, s), 2.88 (2H, br t, *J* = 5.4 Hz), 4.00 (1H, br s), 4.10 (1H, br s), 4.88 (1H, br s), 5.00 (1H, br s), 6.93 (2H, br s), 7.16–7.30 (4H, m), 7.50 (2H, d, *J* = 8.7 Hz), 8.25 (2H, d, *J* = 8.5 Hz). ^13^C NMR (75 MHz, DMSO-d_6_): *δ* 28.0, 31.0 (3C), 34.5, 40.3, 44.8, 124.9 (2C), 126.0, 126.19, 126.24, 127.6 (2C), 128.5, 133.8 (br s), 134.2, 134.7, 153.9, 164.7, 167.1, 169.6. Anal. Calcd for C_22_H_25_N_5_: C, 73.51; H, 7.01; N, 19.48. Found: C, 73.39; H, 7.24; N, 19.21.

### 6-[4-(*N*,*N*-Dimethylamino)phenyl]-4-(3,4-dihydroisoquinolin-2(1*H*)-yl)-1,3,5-triazine-2-amine (9{6,8})

White solid; yield 72 mg (8%); mp 201–202 °C (EtOH). ^1^H NMR (300 MHz, DMSO-d_6_): *δ* 2.86 (2H, br t, *J* = 5.6 Hz), 2.99 (6H, s), 4.03 (2H, br s), 4.91 (2H, br s), 6.73 (2H, br s), 6.75 (2H, d, *J* = 8.9 Hz), 7.16–7.28 (4H, m), 8.19 (2H, d, *J* = 9.0 Hz). ^13^C NMR (75 MHz, DMSO-d_6_): *δ* 28.0, 39.7 (2C), 40.2, 44.8, 110.8 (2C), 123.8, 125.9, 126.1, 126.2, 128.5, 129.2, 133.9 (br s), 134.8, 152.3, 164.7, 166.9, 169.6. Anal. Calcd for C_20_H_22_N_6_: C, 69.34; H, 6.40; N, 24.26. Found: C, 69.22; H, 6.54; N, 24.15.

### 6-(4-Methoxyphenyl)-4-(3,4-dihydroisoquinolin-2(1*H*)-yl)-1,3,5-triazine-2-amine (9{6,9})

White solid; yield 285 mg (35%); mp 165–166 °C (EtOH). ^1^H NMR (300 MHz, DMSO-d_6_): *δ* 2.87 (2H, br t, *J* = 5.7 Hz), 3.83 (3H, s), 4.01 (1H, br s), 4.07 (1H, br s), 4.89 (1H, br s), 4.98 (1H, br s), 6.86 (2H, br s), 7.02 (2H, d, *J* = 9.0 Hz), 7.16–7.29 (4H, m), 8.30 (2H, d, *J* = 8.9 Hz). ^13^C NMR (75 MHz, DMSO-d_6_): *δ* 28.0, 40.3, 44.8, 55.1, 113.4 (2C), 125.9, 126.1, 126.2, 128.5, 129.2, 129.5 (2C), 133.8 (br s), 134.7, 161.7, 164.7, 167.0, 169.2. Anal. Calcd for C_19_H_19_N_5_O: C, 68.45; H, 5.74; N, 21.01. Found: C, 68.33; H, 5.85; N, 20.93.

### 6-[4-(Trifluoromethyl)phenyl]-4-(3,4-dihydroisoquinolin-2(1*H*)-yl)-1,3,5-triazine-2-amine (9{6,11})

White solid; yield 245 mg (26%); mp 181–182 °C (EtOH). ^1^H NMR (300 MHz, DMSO-d_6_): *δ* 2.89 (2H, br t, *J* = 5.2 Hz), 4.00 (1H, br s), 4.13 (1H, br s), 4.89 (1H, br s), 5.03 (1H, br s), 7.11 (2H, br s), 7.18–7.32 (4H, m), 7.87 (2H, d, *J* = 8.2 Hz), 8.52 (2H, d, *J* = 8.1 Hz). ^13^C NMR (75 MHz, DMSO-d_6_): *δ* 28.0, 40.4, 44.9, 124.1 (q, *J* = 272.5 Hz), 125.1 (2C, q, *J* = 3.5 Hz), 126.0, 126.25 126.34, 128.4, 128.5, 131.0 (q, *J* = 31.8 Hz), 133.5*, 133.7*, 134.7 (2C), 140.9, 164.7, 167.1, 168.4; * – signals of two conformers. Anal. Calcd for C_19_H_16_F_3_N_5_: C, 61.45; H, 4.34; N, 18.86. Found: C, 61.31; H, 4.56; N, 18.67.

### 6-[4-(Trifluoromethoxy)phenyl]-4-(3,4-dihydroisoquinolin-2(1*H*)-yl)-1,3,5-triazine-2-amine (9{6,12})

White solid; yield 282 mg (29%); mp 119–120 °C (EtOH). ^1^H NMR (300 MHz, DMSO-d_6_): *δ* 2.89 (2H, br t, *J* = 5.6 Hz), 4.00 (1H, br s), 4.11 (1H, br s), 4.89 (1H, br s), 5.02 (1H, br s), 7.03 (2H, br s), 7.16–7.32 (4H, m), 7.48 (2H, dd, *J* = 0.9, 8.9 Hz), 8.45 (2H, d, *J* = 8.7 Hz). ^13^C NMR (75 MHz, DMSO-d_6_): *δ* 28.0, 40.4, 44.9, 119.9 (q, *J* = 257.0 Hz), 120.4 (2C), 126.0, 126.2 (2C), 128.5, 129.8 (2C), 133.6 (br s), 134.7, 136.1, 150.4 (q, *J* = 1.7 Hz), 164.7, 167.1, 168.4. Anal. Calcd for C_19_H_16_F_3_N_5_O: C, 58.91; H, 4.16; N, 18.08. Found: C, 58.78; H, 4.35; N, 17.92.

### 4-(3,4-Dihydroisoquinolin-2(1*H*)-yl)-6-(3-phenoxyphenyl)-1,3,5-triazine-2-amine (9{6,13})

Brownish solid; yield 492 mg (50%); mp 128–129 °C (EtOH). ^1^H NMR (300 MHz, DMSO-d_6_): *δ* 2.86 (2H, br t, *J* = 5.8 Hz), 4.00 (1H, br s), 4.03 (1H, br s), 4.89 (1H, br s), 4.92 (1H, br s), 6.97 (2H, br s), 7.07 (2H, d, *J* = 7.7 Hz), 7.16–7.24 (6H, m), 7.43 (2H, dd, *J* = 7.7, 8.3 Hz), 7.52 (1H, t, *J* = 7.9 Hz), 7.94 (1H, dd, *J* = 1.4, 2.2 Hz), 8.13 (1H, ddd, *J* = 1.3, 1.3, 7.9 Hz). ^13^C NMR (75 MHz, DMSO-d_6_): *δ* 28.0, 40.4, 44.9, 117.5, 118.5, 118.7, 121.5, 122.9, 123.5, 126.0, 126.2 (2C), 128.5, 129.8, 129.9, 130.0, 133.7 (br s), 134.7, 139.1, 156.6, 156.7, 164.7, 167.1, 168.9. Anal. Calcd for C_24_H_21_N_5_O: C, 72.89; H, 5.35; N, 17.71. Found: C, 72.76; H, 5.48; N, 17.57.

### 6-(3-(Benzyloxy)phenyl)-4-(3,4-dihydroisoquinolin-2(1*H*)-yl)-1,3,5-triazine-2-amine (9{6,14})

White solid; yield 650 mg (64%); mp 143–144 °C (EtOH). ^1^H NMR (300 MHz, DMSO-d_6_): *δ* 2.88 (2H, br t, *J* = 5.3 Hz), 4.00 (1H, br s), 4.08 (1H, br s), 4.87 (1H, br s), 4.99 (1H, br s), 5.19 (2H, s), 6.95 (2H, br s), 7.16–7.25 (5H, m), 7.31–7.45 (4H, m), 7.50 (2H, d, *J* = 7.6 Hz), 7.91–7.96 (2H, m). ^13^C NMR (75 MHz, DMSO-d_6_): *δ* 28.0 (br s), 40.4, 44.9, 69.2, 113.9, 117.6, 120.4, 126.0, 126.2, 126.3, 127.6 (2C), 127.7, 128.4 (2C), 128.5, 129.2, 133.7*, 133.8*, 134.7, 137.0, 138.5, 158.2, 164.7, 167.1, 169.3; * – signals of two conformers. Anal. Calcd for C_25_H_23_N_5_O: C, 73.33; H, 5.66; N, 17.10. Found: C, 73.19; H, 5.83; N, 16.92.

### 6-(4-(Benzyloxy)phenyl)-4-(3,4-dihydroisoquinolin-2(1*H*)-yl)-1,3,5-triazine-2-amine (9{6,15})

White solid; yield 662 mg (65%); mp 143–144 °C (EtOH). ^1^H NMR (300 MHz, DMSO-d_6_): *δ* 2.87 (2H, br t, *J* = 5.5 Hz), 4.00 (1H, br s), 4.09 (1H, br s), 4.88 (1H, br s), 5.00 (1H, br s), 5.18 (2H, s), 6.88 (2H, br s), 7.11 (2H, d, *J* = 8.9 Hz), 7.16–7.24 (4H, m), 7.32–7.44 (3H, m), 7.48 (2H, dd, *J* = 7.6 Hz), 8.29 (2H, d, *J* = 8.9 Hz). ^13^C NMR (75 MHz, DMSO-d_6_): *δ* 28.1 (br s), 40.4, 44.8, 69.3, 114.2 (2C), 126.0, 126.2, 126.3, 127.7 (2C), 127.8, 128.4 (2C), 128.5, 129.4, 129.5 (2C), 133.7*, 133.8*, 134.7, 136.7, 160.9, 164.7, 167.0, 169.1; * – signals of two conformers. Anal. Calcd for C_25_H_23_N_5_O: C, 73.33; H, 5.66; N, 17.10. Found: C, 73.27; H, 5.81; N, 16.96.

### 6-(3,4-Dimethoxyphenyl)-4-(3,4-dihydroisoquinolin-2(1*H*)-yl)-1,3,5-triazin-2-amine (9{6,16})

White solid; yield 350 mg (39%); mp 159–160 °C (EtOH). ^1^H NMR (300 MHz, DMSO-d_6_): *δ* 2.88 (2H, br t, *J* = 5.4 Hz), 3.83 (3H, s), 3.84 (3H, s), 4.00 (1H, br s), 4.07 (1H, br s), 4.89 (1H, br s), 4.99 (1H, br s), 6.89 (2H, br s), 7.06 (1H, d, *J* = 8.6 Hz), 7.17–7.31 (4H, m), 7.89 (1H, d, *J* = 2.0 Hz), 7.98 (1H, dd, *J* = 1.9, 8.4 Hz). ^13^C NMR (75 MHz, DMSO-d_6_): *δ* 28.0, 40.3, 44.8, 55.3, 55.5, 110.8, 110.9, 121.3, 126.0, 126.2, 126.3, 128.5, 129.4, 133.8 (br s), 134.7, 148.1, 151.5, 164.7, 167.0, 169.2. Anal. Calcd for C_20_H_21_N_5_O_2_: C, 66.10; H, 5.82; N, 19.27. Found: C, 65.98; H, 5.90; N, 19.19.

### 6-(3,4,5-Trimethoxyphenyl)-4-(3,4-dihydroisoquinolin-2(1*H*)-yl)-1,3,5-triazine-2-amine (9{6,17})

White solid; yield 372 mg (38%); mp 198–199 °C (EtOH). ^1^H NMR (300 MHz, DMSO-d_6_): *δ* 2.89 (2H, br t, *J* = 5.4 Hz), 3.74 (3H, s), 3.87 (6H, s), 3.98 (1H, br s), 4.11 (1H, br s), 4.89 (1H, br s), 5.00 (1H, br s), 6.96 (2H, br s), 7.14–7.33 (4H, m), 7.67 (2H, s). ^13^C NMR (75 MHz, DMSO-d_6_): *δ* 28.0, 40.4, 44.9, 55.8 (2C), 60.0, 105.1 (2C), 126.0, 126.2, 126.3, 128.5, 132.3, 133.8 (br s), 134.8, 140.3, 152.5 (2C), 164.7, 167.0, 169.1. Anal. Calcd for C_21_H_23_N_5_O_3_: C, 64.11; H, 5.89; N, 17.80. Found: C, 63.97; H, 6.03; N, 17.65.

### 4-(3,4-Dihydroisoquinolin-2(1*H*)-yl)-6-(2-thienyl)-1,3,5-triazine-2-amine (9{6,18})

Brownish solid; yield 350 mg (50%); mp 170–171 °C (EtOH). ^1^H NMR (300 MHz, DMSO-d_6_): *δ* 2.87 (2H, br t, *J* = 5.8 Hz), 4.02 (2H, br s), 4.88 (1H, br s), 4.94 (1H, br s), 6.96 (2H, br s), 7.16–7.26 (5H, m), 7.73 (1H, dd, *J* = 1.3, 5.0 Hz), 7.93 (1H, dd, *J* = 1.0, 3.7 Hz). ^13^C NMR (75 MHz, DMSO-d_6_): *δ* 28.0, 40.4, 44.8, 126.0, 126.2, 126.3, 127.9, 128.5, 129.0, 130.5, 133.7 (br s), 134.7, 142.8, 164.3, 166.1, 166.8. Anal. Calcd for C_16_H_15_N_5_S: C, 62.11; H, 4.89; N, 22.64. Found: C, 61.97; H, 5.00; N, 22.51.

### 6-(4-Fluorophenyl)-4-(indolin-1-yl)-1,3,5-triazine-2-amine (9{7,3})

Brownish solid; yield 201 mg (26%); mp 195–196 °C (EtOH). ^1^H NMR (300 MHz, DMSO-d_6_): *δ* 3.16 (2H, br t, *J* = 8.6 Hz), 4.24 (2H, br s), 6.96 (1H, dt, *J* = 0.7, 7.4 Hz), 7.17–7.29 (4H, m), 7.36 (2H, dd, *J* = 8.7, 8.7 Hz), 8.41 (2H, dd, *J* = 5.8, 8.9 Hz), 8.50 (1H, d, *J* = 8.0 Hz). ^13^C NMR (75 MHz, DMSO-d_6_): *δ* 26.4, 47.8, 115.2 (2C, d, *J* = 21.7 Hz), 116.2, 122.0, 124.7, 126.8, 130.2 (2C, d, *J* = 8.9 Hz), 132.6, 133.2 (d, *J* = 3.0 Hz), 142.7, 163.1, 164.2 (d, *J* = 248.6 Hz), 165.9, 166.8, 168.8. Anal. Calcd for C_17_H_14_FN_5_: C, 66.44; H, 4.59; N, 22.79. Found: C, 66.38; H, 4.72; N, 22.67.

### 6-[4-(*N*,*N*-Dimethylamino)phenyl]-4-(indolin-1-yl)-1,3,5-triazine-2-amine (9{7,8})

Brownish solid; yield 513 mg (62%); mp 226–227 °C (EtOH). ^1^H NMR (300 MHz, DMSO-d_6_): *δ* 3.01 (6H, s), 3.14 (2H, br t, *J* = 8.2 Hz), 4.21 (2H, br t, *J* = 8.6 Hz), 6.79 (2H, d, *J* = 8.9 Hz), 6.89–7.00 (3H, m), 7.16–7.27 (2H, m), 8.21 (2H, d, *J* = 9.1 Hz), 8.51 (1H, d, *J* = 7.9 Hz). ^13^C NMR (75 MHz, DMSO-d_6_): *δ* 26.4, 40.6 (2C), 47.7, 111.0 (2C), 121.6, 123.5, 124.6, 126.8, 129.2 (2C), 132.5 (2C), 143.0, 152.5, 163.1, 166.7, 169.8. Anal. Calcd for C_19_H_20_N_6_: C, 68.65; H, 6.06; N, 25.28. Found: C, 68.49; H, 6.22; N, 25.15.

### 6-(4-Methoxyphenyl)-4-(indolin-1-yl)-1,3,5-triazine-2-amine (9{7,9})

Brownish solid; yield 276 mg (35%); mp 219–220 °C (EtOH). ^1^H NMR (300 MHz, DMSO-d_6_): *δ* 3.15 (2H, br t, *J* = 8.5 Hz), 3.85 (3H, s), 4.23 (2H, br t, *J* = 8.5 Hz), 6.95 (1H, dt, *J* = 0.7, 7.4 Hz), 7.07 (2H, d, *J* = 8.7 Hz), 7.12 (2H, br s), 7.17–7.29 (2H, m), 8.32 (2H, d, *J* = 8.9 Hz), 8.51 (1H, d, *J* = 8.0 Hz). ^13^C NMR (75 MHz, DMSO-d_6_): *δ* 26.4, 47.7, 55.2, 113.6 (2C), 116.2, 121.8, 124.7, 126.8, 129.1, 129.6, 132.6 (2C), 142.8, 162.0, 163.1, 166.8, 169.4. Anal. Calcd for C_18_H_17_N_5_O: C, 67.70; H, 5.37; N, 21.93. Found: C, 67.63; H, 5.46; N, 21.88.

### 6-[4-(Trifluoromethyl)phenyl]-4-(indolin-1-yl)-1,3,5-triazine-2-amine (9{7,11})

White solid; yield 240 mg (27%); mp 227–228 °C (EtOH). ^1^H NMR (300 MHz, DMSO-d_6_): *δ* 3.17 (2H, br t, *J* = 8.4 Hz), 4.27 (2H, br s), 6.98 (1H, dt, *J* = 0.8, 7.4 Hz), 7.18–7.30 (2H, m), 7.34 (2H, br s), 7.92 (2H, d, *J* = 8.0 Hz), 8.47–8.57 (3H, m). Anal. Calcd for C_18_H_14_F_3_N_5_: C, 60.50; H, 3.95; N, 19.60. Found: C, 60.36; H, 4.08; N, 19.47.

### 6-[4-(Trifluoromethoxy)phenyl]-4-(indolin-1-yl)-1,3,5-triazine-2-amine (9{7,12})

White solid; yield 145 mg (16%); mp 195–196 °C (EtOH). ^1^H NMR (300 MHz, DMSO-d_6_): *δ* 3.17 (2H, br t, *J* = 8.5 Hz), 4.24 (2H, br s), 6.97 (1H, dt, *J* = 0.9, 7.4 Hz), 7.17–7.34 (4H, m), 7.53 (2H, d, *J* = 8.6 Hz), 8.43–8.52 (3H, m). Anal. Calcd for C_18_H_14_F_3_N_5_O: C, 57.91; H, 3.78; N, 18.76. Found: C, 57.85; H, 3.86; N, 18.67.

### 6-(4-(Benzyloxy)phenyl)-4-(indolin-1-yl)-1,3,5-triazine-2-amine (9{7,15})

Brownish solid; yield 222 mg (22%); mp 188–189 °C (EtOH). ^1^H NMR (300 MHz, DMSO-d_6_): *δ* 3.15 (2H, br t, *J* = 8.5 Hz), 4.23 (2H, br s), 5.20 (2H, s), 6.95 (1H, dt, *J* = 0.8, 7.4 Hz), 7.06–7.28 (6H, m), 7.30–7.44 (3H, m), 7.49 (2H, dd, *J* = 1.5, 8.1 Hz), 8.32 (2H, d, *J* = 8.9 Hz), 8.50 (1H, d, *J* = 7.9 Hz). ^13^C NMR (75 MHz, DMSO-d_6_): *δ* 26.4, 47.7, 69.3, 114.4 (2C), 116.2, 121.8, 124.7, 126.8, 127.7 (2C), 127.8, 128.4 (3C) 129.2, 129.5 (2C), 132.6, 136.6, 142.8, 161.1, 163.1, 166.7, 169.4. Anal. Calcd for C_24_H_21_N_5_O: C, 72.89; H, 5.35; N, 17.71. Found: C, 72.76; H, 5.53; N, 17.52.

### 6-(3,4-Dimethoxyphenyl)-4-(indolin-1-yl)-1,3,5-triazine-2-amine (9{7,16})

Brownish solid; yield 492 mg (56%); mp 183–184 °C (EtOH). ^1^H NMR (300 MHz, DMSO-d_6_): *δ* 3.16 (2H, br t, *J* = 8.6 Hz), 3.85 (3H, s), 3.86 (3H, s), 4.24 (2H, br s), 6.95 (1H, dt, *J* = 0.9, 7.4 Hz), 7.06–7.28 (5H, m), 7.94 (1H, d, *J* = 1.9 Hz), 8.00 (1H, dd, *J* = 1.9, 8.4 Hz), 8.52 (1H, d, *J* = 7.9 Hz). ^13^C NMR (75 MHz, DMSO-d_6_): *δ* 26.5, 47.7, 55.2, 55.5, 110.7, 111.0, 116.2, 121.3, 121.8, 124.7, 126.8, 129.2, 132.6, 142.9, 148.2, 151.7, 163.1, 166.8, 169.4. Anal. Calcd for C_19_H_19_N_5_O_2_: C, 65.32; H, 5.48; N, 20.04. Found: C, 65.23; H, 5.57; N, 19.95.

### 6-(3,4,5-Trimethoxyphenyl)-4-(indolin-1-yl)-1,3,5-triazine-2-amine (9{7,17})

White solid; yield 253 mg (27%); mp 199–200 °C (EtOH). ^1^H NMR (300 MHz, DMSO-d_6_): *δ* 3.16 (2H, br t, *J* = 8.5 Hz), 3.75 (3H, s), 3.88 (6H, s), 4.24 (2H, br s), 6.96 (1H, dt, *J* = 1.0, 7.4 Hz), 7.16–7.29 (4H, m), 7.71 (2H, s), 8.52 (1H, d, *J* = 8.0 Hz). ^13^C NMR (75 MHz, DMSO-d_6_): *δ* 26.5, 47.7, 55.7 (2C), 60.0, 105.0 (2C), 116.3, 121.9, 124.7, 126.8, 132.0, 132.6, 140.3, 142.8, 152.6 (2C), 163.0, 166.9, 169.2. Anal. Calcd for C_20_H_21_N_5_O_3_: C, 63.31; H, 5.58; N, 18.46. Found: C, 63.18; H, 5.74; N, 18.35.

### 4-(Indolin-1-yl)-6-(2-thienyl)-1,3,5-triazine-2-amine (9{7,18})

Brownish solid; yield 578 mg (78%); mp 163–164 °C (EtOH). ^1^H NMR (300 MHz, DMSO-d_6_): *δ* 3.15 (2H, br t, *J* = 8.6 Hz), 4.19 (2H, br t, *J* = 7.8 Hz), 6.96 (1H, dt, *J* = 0.9, 7.4 Hz), 7.16–7.29 (5H, m), 7.79 (1H, dd, *J* = 1.1, 5.0 Hz), 7.95 (1H, dd, *J* = 1.2, 3.6 Hz), 8.48 (1H, d, *J* = 7.9 Hz). ^13^C NMR (75 MHz, DMSO-d_6_): *δ* 26.4, 47.7, 116.3, 122.0, 124.7, 126.9, 128.1, 129.3, 131.0, 132.6, 142.5, 142.7, 162.7, 166.5 (2C). Anal. Calcd for C_15_H_13_N_5_S: C, 61.00; H, 4.44; N, 23.71. Found: C, 60.86; H, 4.52; N, 23.59.

### Cytotoxicity evaluation

An MTS (3-(4,5-dimethylthiazol-2-yl)-5-(3-carboxymethoxyphenyl)-2-(4-sulfophenyl)-2*H*-tetrazolium inner salt) assay^20^ was used to assess the leukemic cell viability. Jurkat T cells (human leukemic T cell, clone E6-1) from American Type Culture Collection (ATCC) were cultured in RPMI-1640 medium (Nacalai Tesque, Japan) supplemented with 10% v/v fetal bovine serum (FBS) (Biosera, France). The cells were maintained at 37 °C in a humidified 5% CO_2_ incubator (Thermo Fisher, USA). A total of 2 × 10^3^ cells per 100 μL cell culture media were seeded into each well of a 96-well plate and incubated for 24 h. Then, tested compounds or reference drugs [6-mercaptopurine (Merck Millipore, Germany), methotrexate (Merck Millipore, Germany) and cytarabine (Merck Millipore, Germany)] were added, followed by incubation for 72 h. After that, a mixture of MTS (Sigma Aldrich, USA) and phenazine methosulfate (PMS) (Nacalai Tesque, Japan) were added to each well, followed by another incubation for 1 h to 4 h at 37 °C. The absorbance in each well was measured at 490 nm using a microplate reader (Tecan NanoQuant Infinite M200 Pro, Austria). The percentage of cell viability was estimated by comparing absorbance in wells with the treated and untreated (vehicle control) cells using the following formula: OD_treated_/OD_untreated_ × 100%. All experiments were performed in triplicate and repeated in three independent experiments. The GI_50_ values were calculated using sigmoidal concentration-response curves generated by the GraphPad Prism 8 program.^21^

An MTT (3-(4,5-dimethylthiazol-2-yl)-2,5-diphenyltetrazolium bromide) assay^22^ was used to evaluate the cytotoxicity of most active compounds against the non-cancerous MRC-5 cells. A similar procedure was applied for the MTT assay by changing the MTS/PMS solution to the MTT solution and measuring the absorbance at 570 nm.

### Acridine orange/propidium iodide double fluorescence staining and cell morphology study

Fluorescence microscopy was applied to portray apoptotic and necrotic changes in Jurkat T cells using AO/PI fluorescence staining.^23^ The Jurkat T cells were cultured as described above. A total of 5 × 10^5^ cells (based on the doubling time for the cell line) per 1 mL cell culture media were seeded into each well of a 6-well plate (Biologix, USA) and incubated for 24 h. The cells were then treated with the most active compound, positive control and negative control, followed by incubation for 24 h, 48 h or 72 h. The fluorescence dye containing AO (10 μg mL^−1^, 16 μL) and PI (10 μg mL^−1^, 13 μL) was prepared immediately prior to use. The treated cells, positive and negative controls containing dyes were examined immediately under an inverted UV-fluorescent microscope (Nikon Ti2-E, Japan) to prevent fluorescence dyes from fading. The images of viable cells and non-viable cells after treatment were taken by Nikon Ti2-E software. The observations were repeated in three independent experiments.

### 3D-QSAR modelling

The QSAR model was built using the 3D-QSAR protocol in Discovery Studio version 18 (Biovia, D.S., USA).^18^ The model was created using data for the 24 most active compounds identified in the initial screening. Applying the ‘Generate training and test set’ protocol in Discover Studio, 20 compounds were selected randomly as a training set, while the remaining four compounds were used as a test set to assess the reliability of the developed model. The GI_50_ values of the compounds against Jurkat T cells were converted to the negative logarithmic scale (pGI_50_) using the ‘Prepare dependent values’ protocol of Discovery Studio followed by the alignment of compounds to the minimum energy with steric (50%) and electrostatic (50%) fields using the ‘Align small molecules” module. The CHARMm force field was used to create a model for the electrostatic potential and for the van der Waals potential. A +1*e* point charge was applied as the electrostatic potential probe. The solvation effect was mimicked using the distance-dependent dielectric constant. A carbon atom with a 1.5 Å radius was used as a probe in the van der Waals potential model. The truncation of 30 kcal mol^−1^ was set for both the steric and electrostatic energies and the standard Discovery Studio version 18 parameters were applied. A Partial Least Square (PLS) model was built using energy grids as descriptors in the ‘Create 3D-QSAR model’ protocol of Discovery Studio.

### X-ray crystallography

X-ray intensity data for a colourless prism (0.08 × 0.12 × 0.15 mm^3^) of 9{2,6} were measured at *T* = 100 K on a Rigaku/Oxford Diffraction XtaLAB Synergy diffractometer (Dualflex, AtlasS2) fitted with CuKα radiation (*λ* = 1.54178 Å) so that *θ*_max_ = 67.1° (corresponding to 100% completeness = 2524 independent reflections from the 19004 measured). Data reduction and gaussian absorption correction were accomplished with CrysAlisPro.^24^ The structure was solved with SHELXT-2018/2 (ref. 25) and refined (anisotropic displacement parameters and C-bound H atoms in the riding model approximation) on *F*^2^ with SHELXT-2018/2.^26^ The N-bound H atoms were refined with the N–H bond lengths constrained to 0.88 ± 0.01 Å, and with *U*_iso_(H) = 1.2*U*_eq_(N). A weighting scheme of the form *w* = 1/[*σ*^2^(*F*_o_^2^) + (*aP*)^2^ + *bP*] where *P* = (*F*_o_^2^ + 2*F*_c_^2^)/3) was introduced and at the conclusion of the refinement *a* = 0.059 and *b* = 0.572. One reflection, *i.e.* (−1 1 16), was omitted from the final cycles of refinement owing to poor agreement. The molecular structure diagram was generated with ORTEP for Windows^27^ with 70% displacement ellipsoids, and the packing diagrams were drawn with DIAMOND.^28^ Additional data analysis was made with PLATON.^29^

### Crystal data for 9{2,6}

C_15_H_19_N_5_, *M* = 269.35, monoclinic, *P*2_1_/*c*, *a* = 8.8670(1), *b* = 7.1430(1), *c* = 22.2918(3) Å, *β* = 90.537(1)°, *V* = 1411.83(3) Å^3^, *Z* = 4, *D*_x_ = 1.267 g cm^−3^, *F*(000) = 576, *μ* = 0.631 mm^−1^, no. unique reflns = 2524 (*R*_int_ = 0.024), no. reflns with *I* ≥ 2.0*σ*(*I*) = 2371, no. parameters = 188, *R*(obs. data) = 0.038, w*R*_2_(all data) = 0.105.

## Conflicts of interest

There are no conflicts to declare.

## Supplementary Material

RA-014-D3RA08091A-s001

RA-014-D3RA08091A-s002
