# Supplementary material for: 6-Aryl-4-cycloamino-1,3,5-triazine-2-amines: synthesis, antileukemic activity, and 3D-QSAR modelling
Source: RSC Adv. 2024 Mar 11;14(12):8264–82. doi: 10.1039/d3ra08091a (PMC10925993; doi:10.1039/d3ra08091a)

Electronic Supplementary Material (ESI) for RSC Advances.
This journal is © The Royal Society of Chemistry 2024

Electronic Supplementary Information

6-Aryl-4-cycloamino-1,3,5-triazine-2-amines: Synthesis, antileukemic activity, and 3D-QSAR modeling

Muhammad Syafiq Bin Shahari,[a] *Ahmad Junaid,*[b]

Edward R. T. Tiekink[c] *and Anton V. Dolzhenko**[de]

[a] Center for Drug Design, College of Pharmacy, University of Minnesota, Nils Hasselmo Hall, 312 Church Street SE, Mail Code 1191, Minneapolis, Minnesota 55455, USA

[b] Inimmune Corp., 1121 E Broadway St, Ste 106, Missoula, Montana 59802, USA

[c] Department of Chemistry, Universitat de les Illes Balears, Crta de Valldemossa km 7.5, 07122 Palma de Mallorca, Spain

[d] School of Pharmacy, Monash University Malaysia, Jalan Lagoon Selatan, Bandar Sunway, Selangor Darul Ehsan 47500, Malaysia

[e] Curtin Medical School, Curtin Health Innovation Research Institute, Faculty of Health Sciences, Curtin University, GPO Box U1987 Perth, Western Australia 6845, Australia

Contents

Figure S1. Molecular packing diagrams for **9**{*2,6*} (CCDC 2310051) S3

Copies of ^{1}H and ^{13}C NMR spectra of prepared compounds **9**{*a,b*} S4-S147

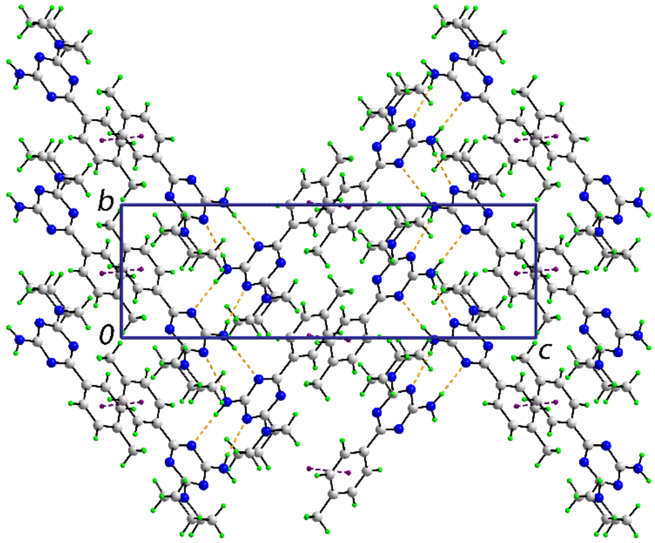

(a)

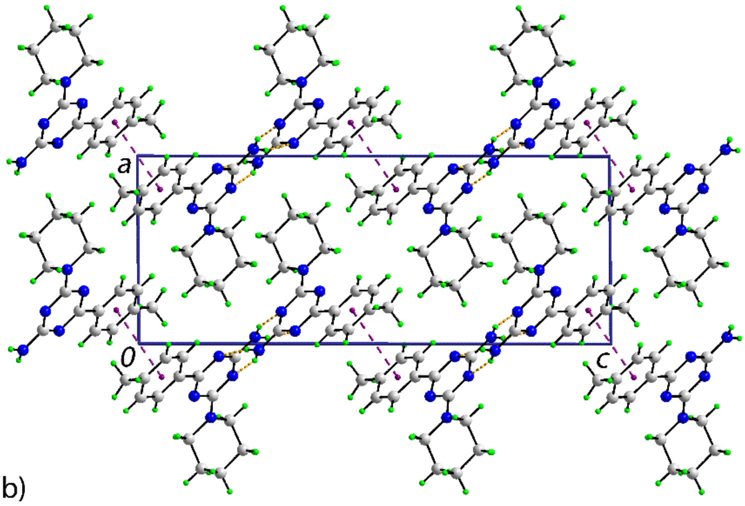

(b)

Figure S1. Molecular packing diagrams for **9**{*2,6*}: (a) a view of the supramolecular layer in the *bc*-plane and (b) a view of the unit-cell contents in projection down the *b*-axis. The amine-N–H···N(triazine) hydrogen-bonding and π-stacking interactions are highlighted as orange and purple dashed lines, respectively. Intermolecular contacts: N21–H1n···N3^{i}: H1n···N3^{i} = 2.327(12) Å, N21···N3^{i} = 3.1774(13) Å with angle at H1n = 161.7(12)°; N21–H2n···N1^{ii}: N21–H2n···N1^{ii} = 2.166(12) Å, N21···N1^{ii} = 3.0304(13) Å with angle at H2n = 167.3(13)°; ring centroid(C61-C66)···ring centroid(C61-C66)^{iii} = 3.8345(7) Å with an off-set = 1.642 Å for symmetry operations (i) –*x*, $^{1}/_{2}$+*y*, $^{1}/_{2}$–*z*; (ii) –*x*, –$^{1}/_{2}$+*y*, $^{1}/_{2}$–*z*; (iii) –*x*, 2–*y*, 1–*z*.

6-(3-Fluorophenyl)-4-pyrrolidino-1,3,5-triazine-2-amine (**9**{*1*,*2*})

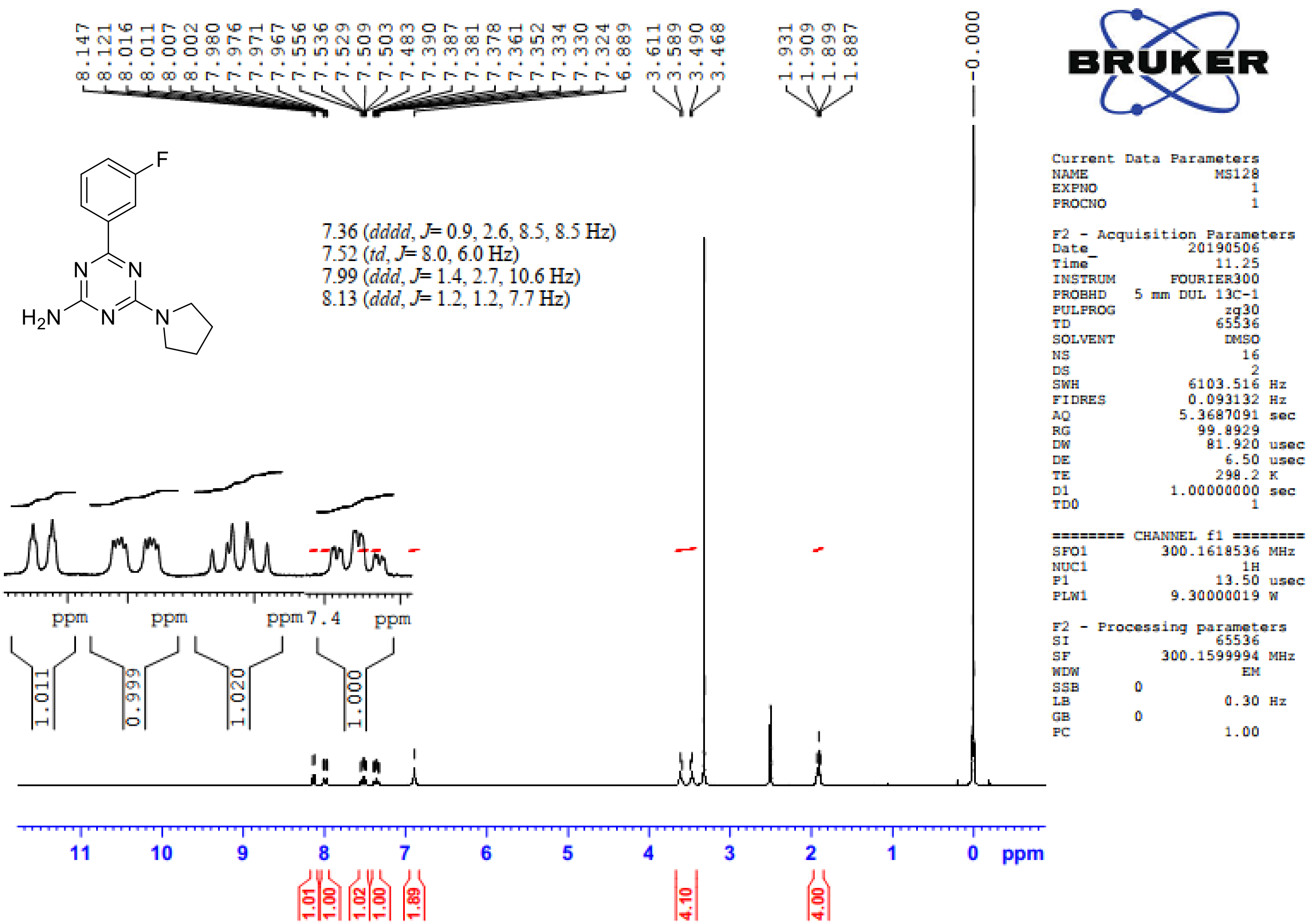

6-(3-Fluorophenyl)-4-pyrrolidino-1,3,5-triazine-2-amine (**9**{*1*,*2*})

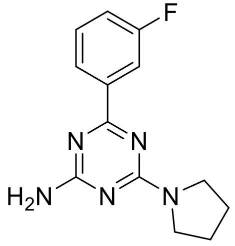

113.9 (*d*, *J*= 22.8 Hz)
117.8 (*d*, *J*= 21.1 Hz)
123.6 (*d*, *J*= 2.2 Hz)
130.1 (*d*, *J*= 7.4 Hz)
139.8 (*d*, *J*= 7.5 Hz)
162.1 (*d*, *J*= 242.4 Hz)

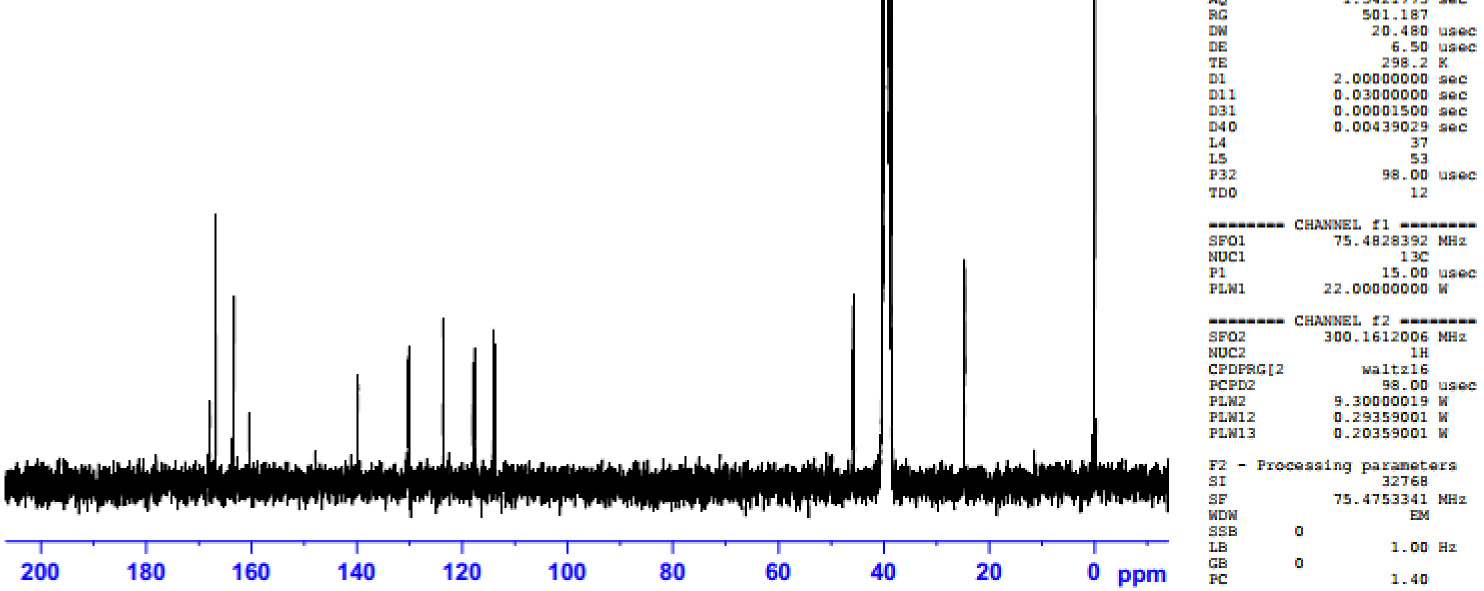

6-(4-Fluorophenyl)-4-pyrrolidino-1,3,5-triazine-2-amine (**9**{*1*,*3*})

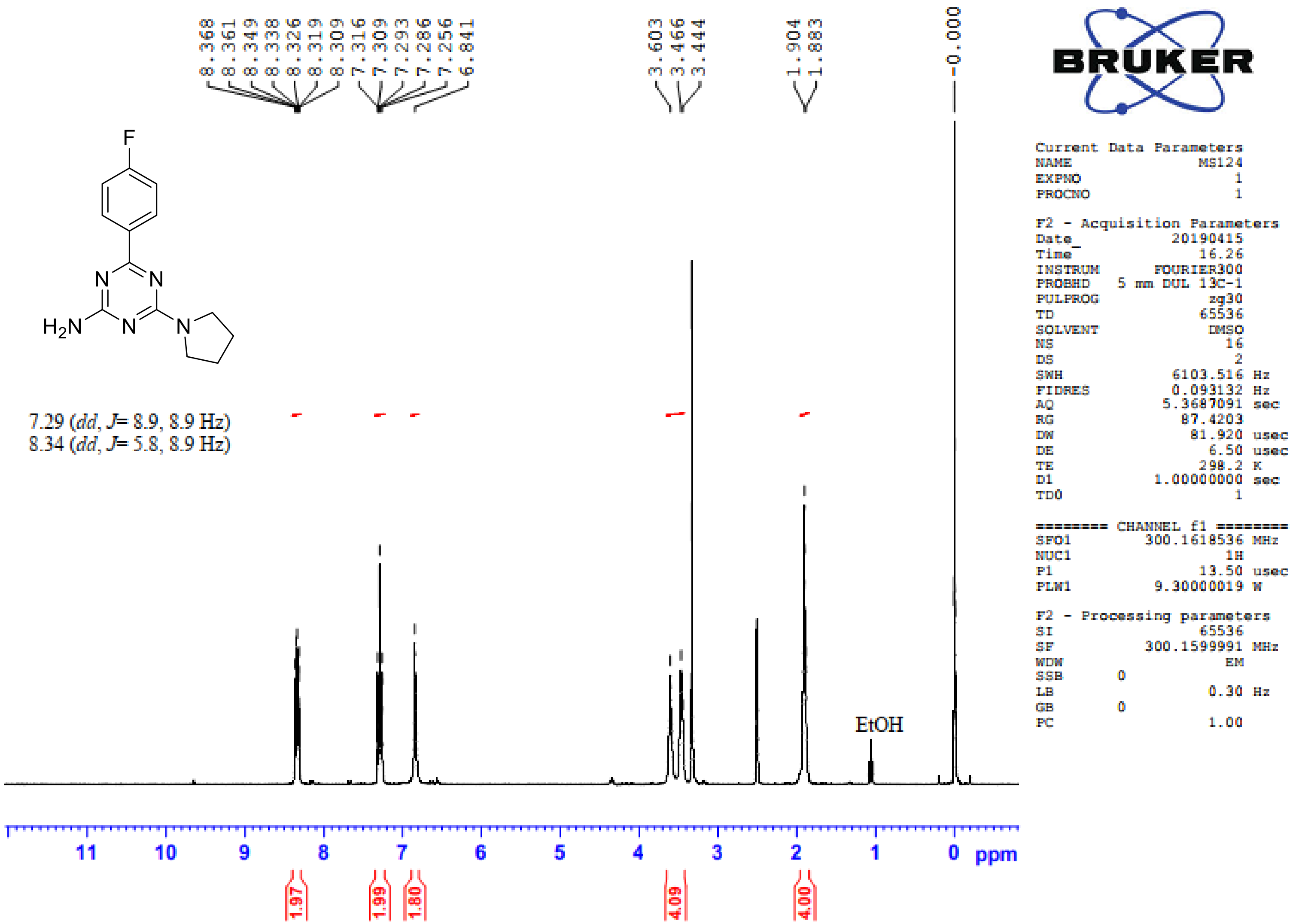

6-(4-Fluorophenyl)-4-pyrrolidino-1,3,5-triazine-2-amine (**9**{*1,3*})

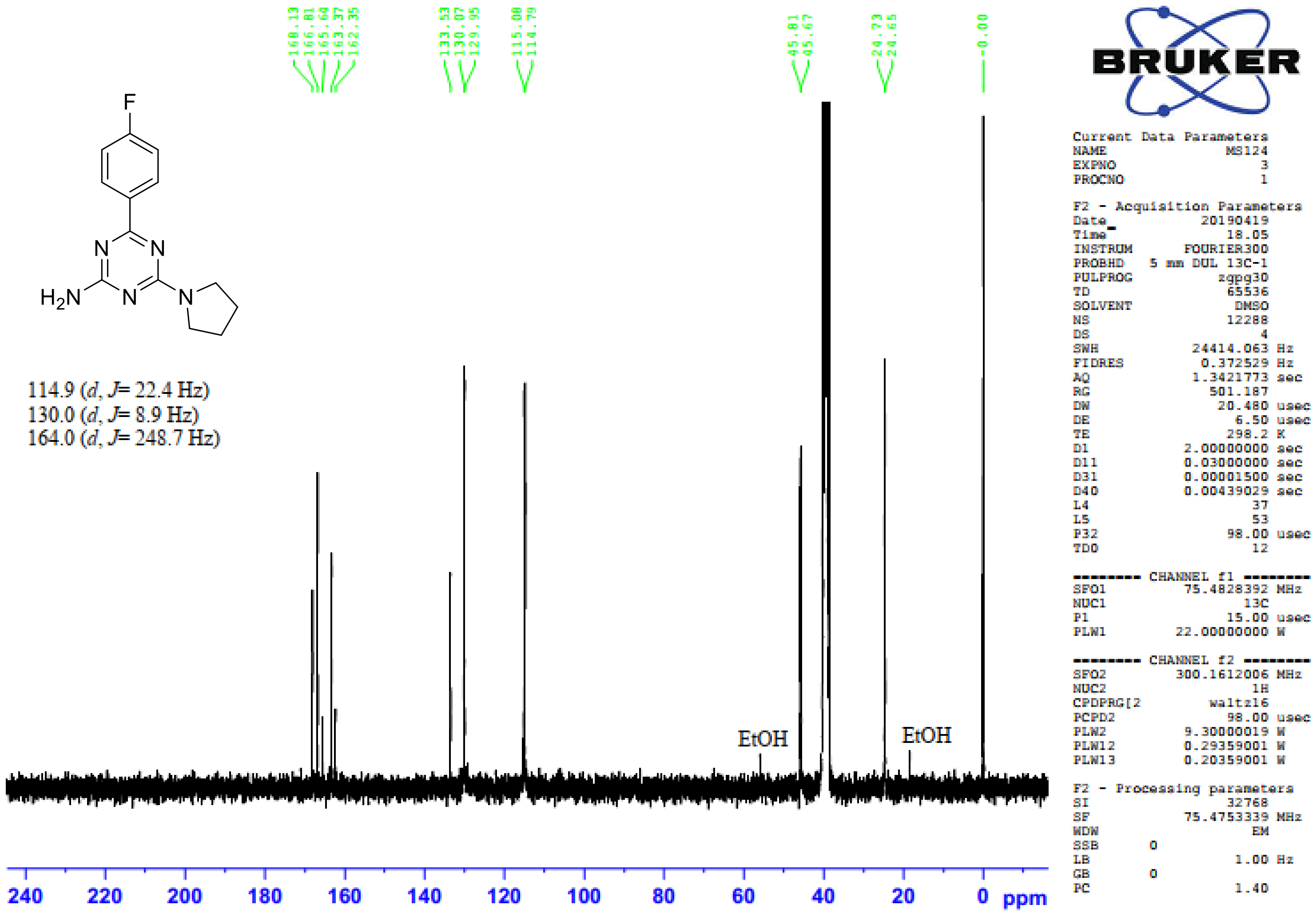

6-(3-Methylphenyl)-4-pyrrolidino-1,3,5-triazine-2-amine (**9**{*1*,*5*})

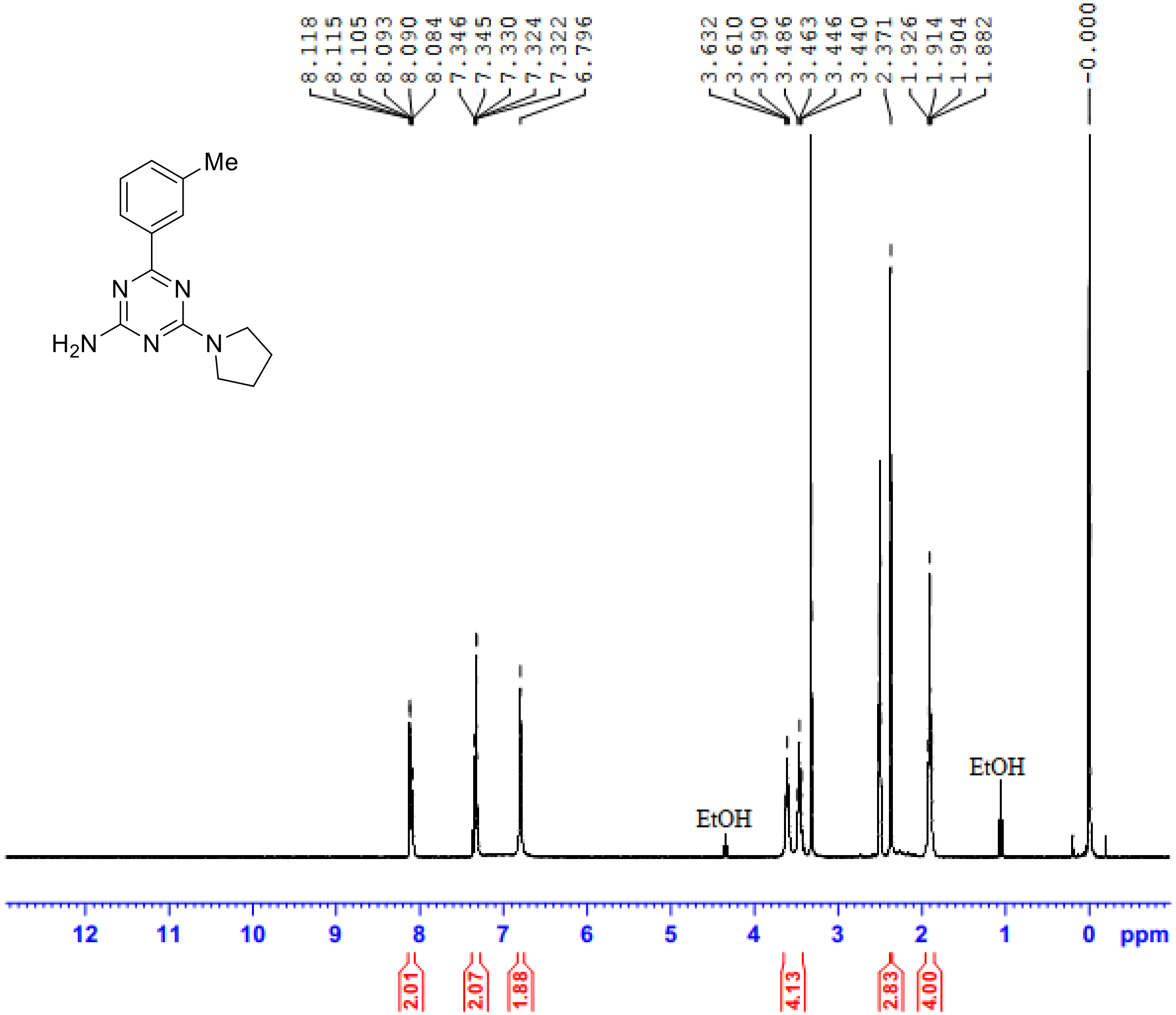

BRUKER

Current Data Parameters
NAME MS125
EXPNO 1
PROCNO 1

F2 - Acquisition Parameters
Date_ 20190415
Time 16.30
INSTRUM FOURIER300
PROBHD 5 mm DUL 13C-1
PULPROG zg30
TD 65536
SOLVENT DMSO
NS 16
DS 2
SWH 6103.516 Hz
FIDRES 0.093132 Hz
AQ 5.3687091 sec
RG 109.925
DW 81.920 usec
DE 6.50 usec
TE 298.2 K
D1 1.00000000 sec
TD0 1

======== CHANNEL f1 ========
SFO1 300.1618536 MHz
NUC1 1H
P1 13.50 usec
PLW1 9.30000019 W

F2 - Processing parameters
SI 65536
SF 300.1600003 MHz
WDW EM
SSB 0
LB 0.30 Hz
GB 0
PC 1.00

6-(3-Methylphenyl)-4-pyrrolidino-1,3,5-triazine-2-amine (**9**{*1*,*5*})

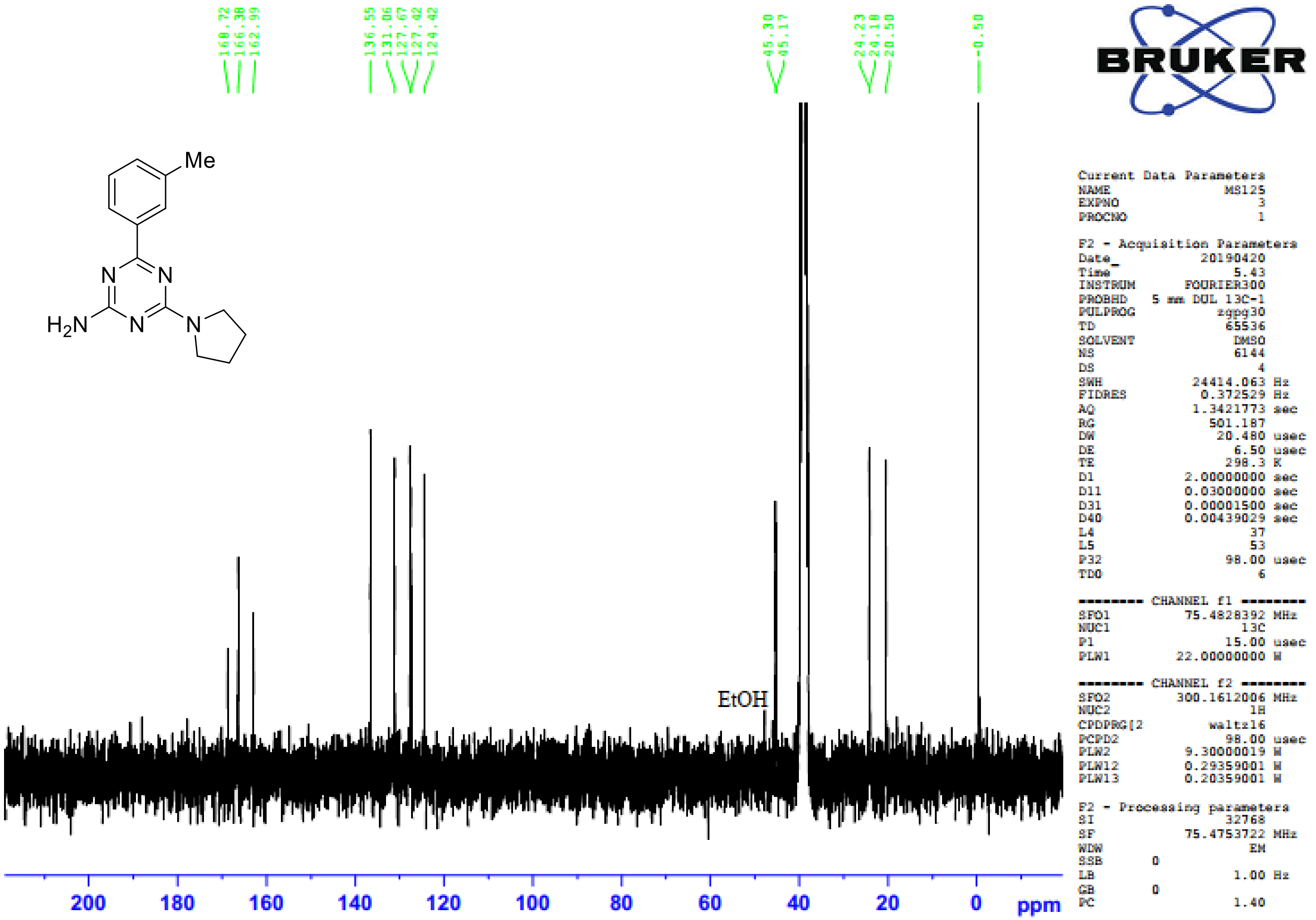

6-(4-Methylphenyl)-4-pyrrolidino-1,3,5-triazine-2-amine (**9**{*1*,*6*})

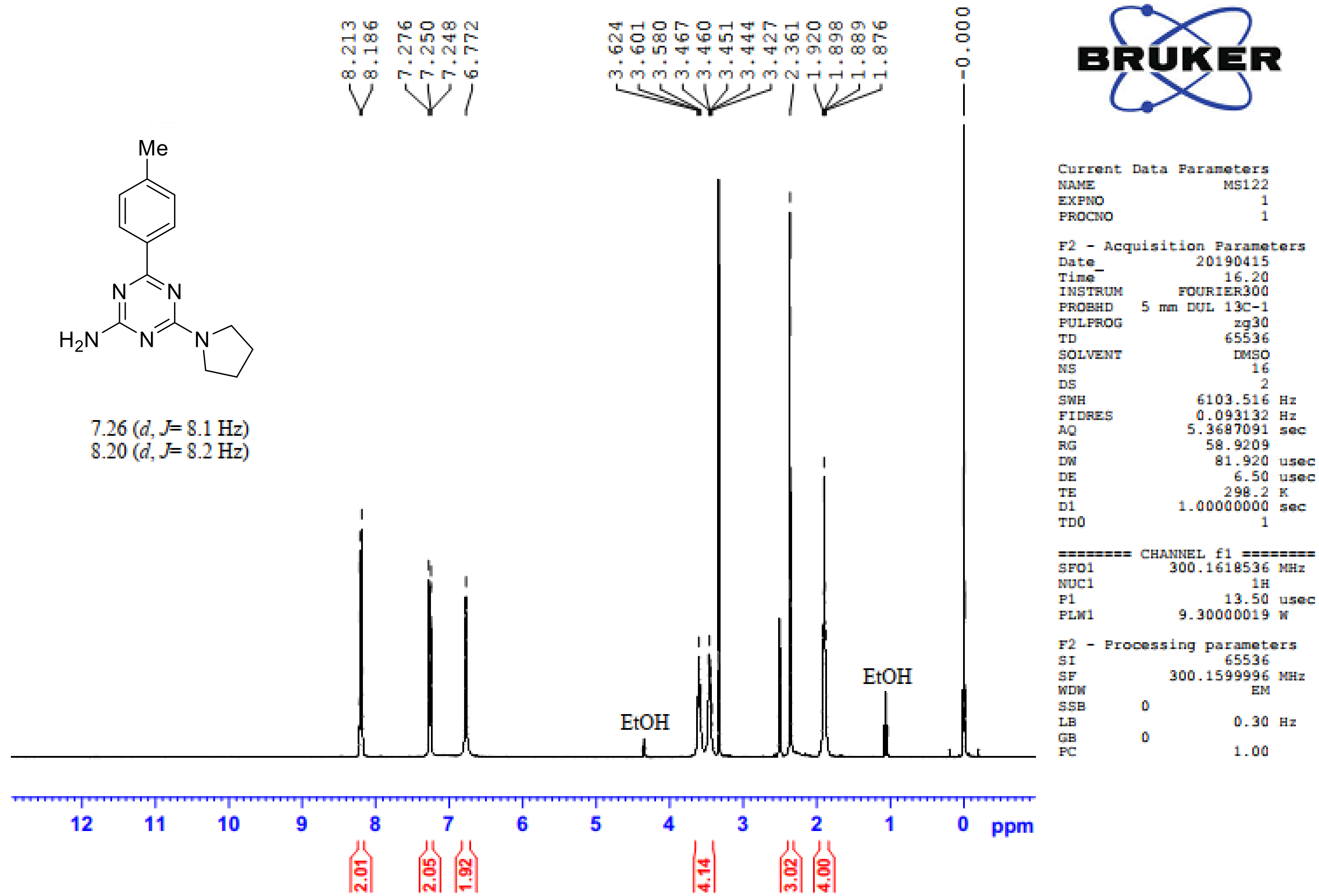

6-(4-Methylphenyl)-4-pyrrolidino-1,3,5-triazine-2-amine (**9**{*1*,*6*})

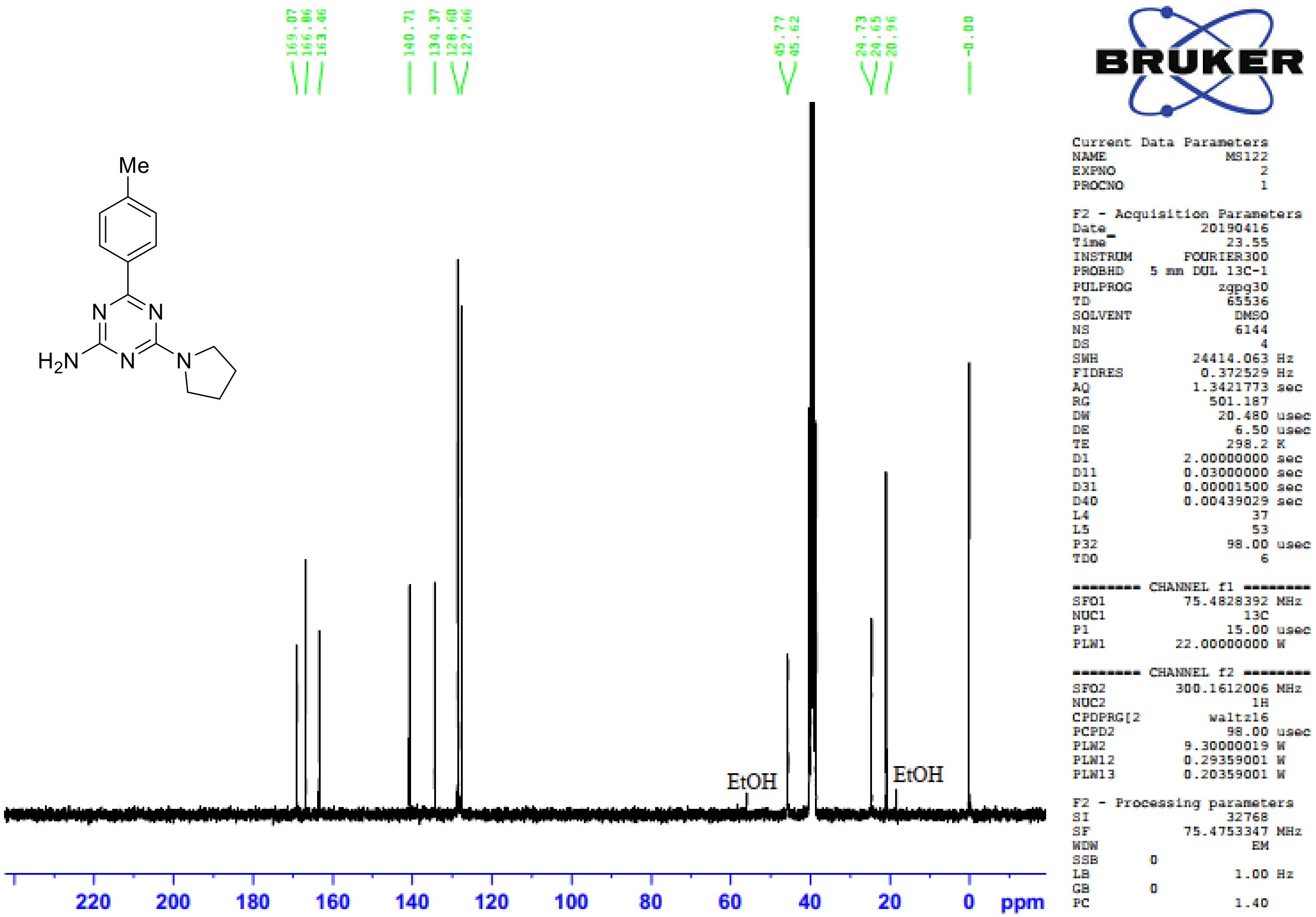

6-[4-(*tert*-Butyl)phenyl]-4-pyrrolidino-1,3,5-triazine-2-amine (**9**{*1,7*})

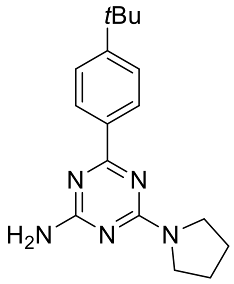

7.48 (*d*, *J*= 8.6 Hz)
8.22 (*d*, *J*= 8.6 Hz)

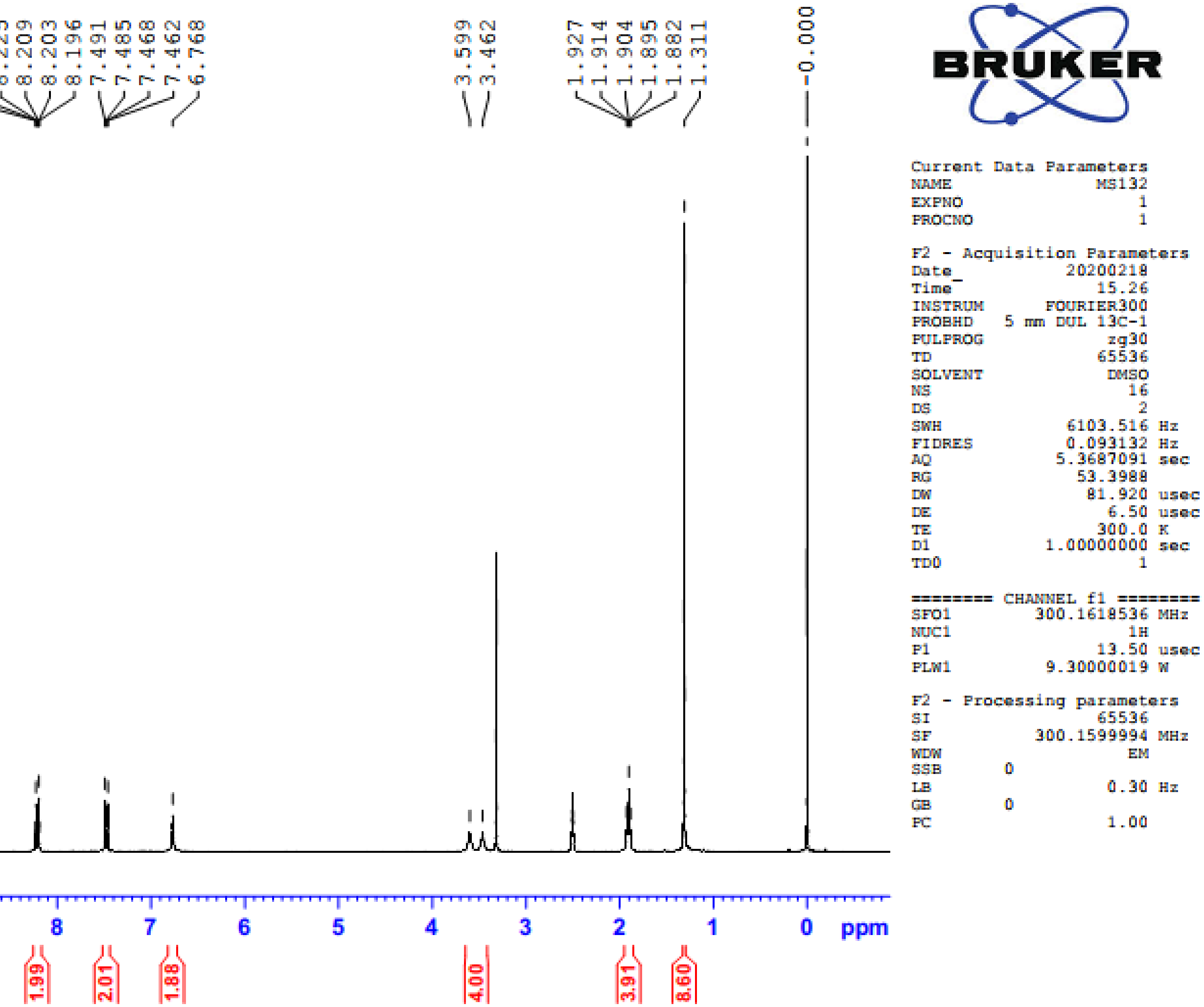

6-[4-(*tert*-Butyl)phenyl]-4-pyrrolidino-1,3,5-triazine-2-amine (**9**{*1,7*})

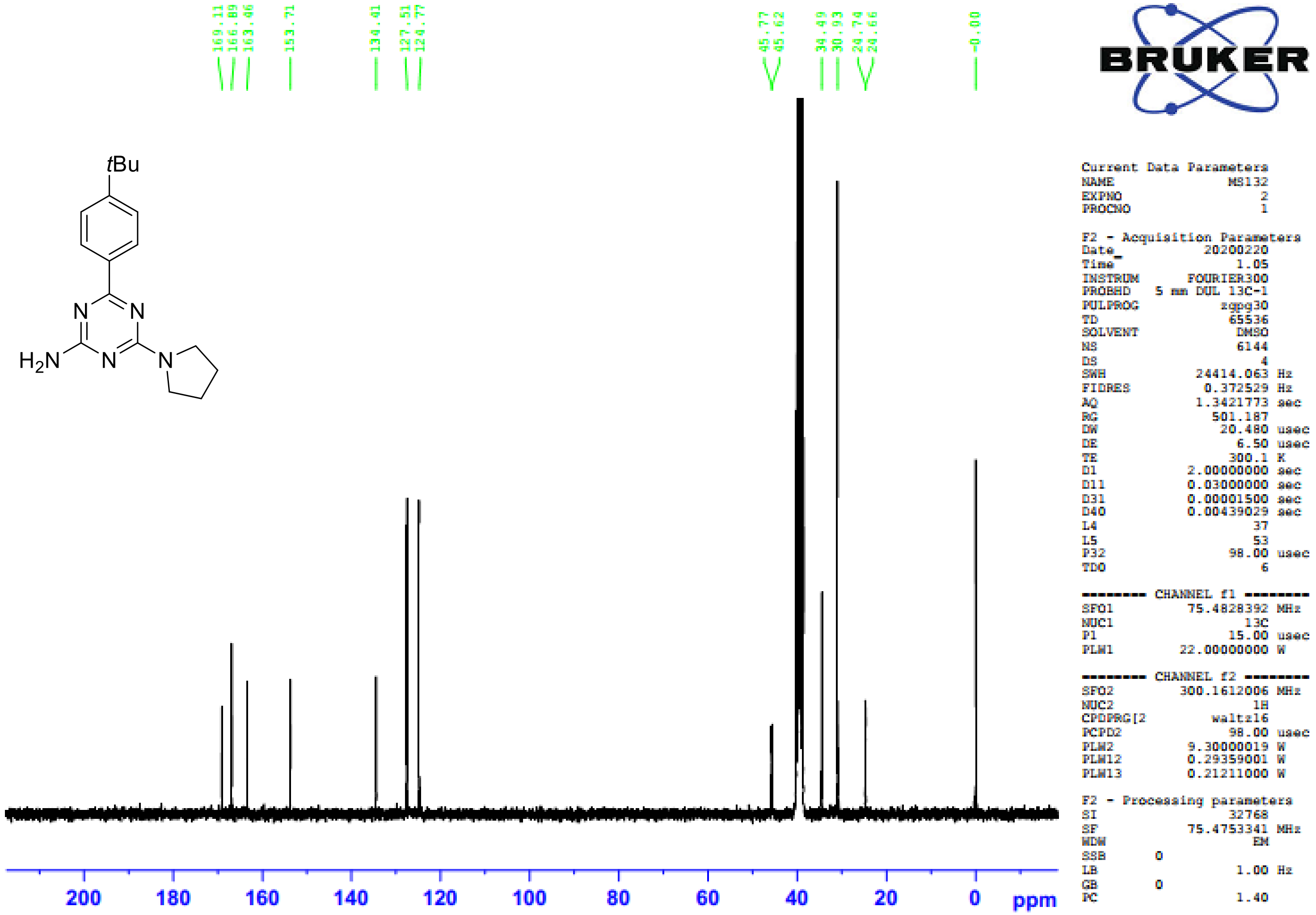

6-(4-Methoxyphenyl)-4-pyrrolidino-1,3,5-triazine-2-amine (**9**{*1*,*9*})

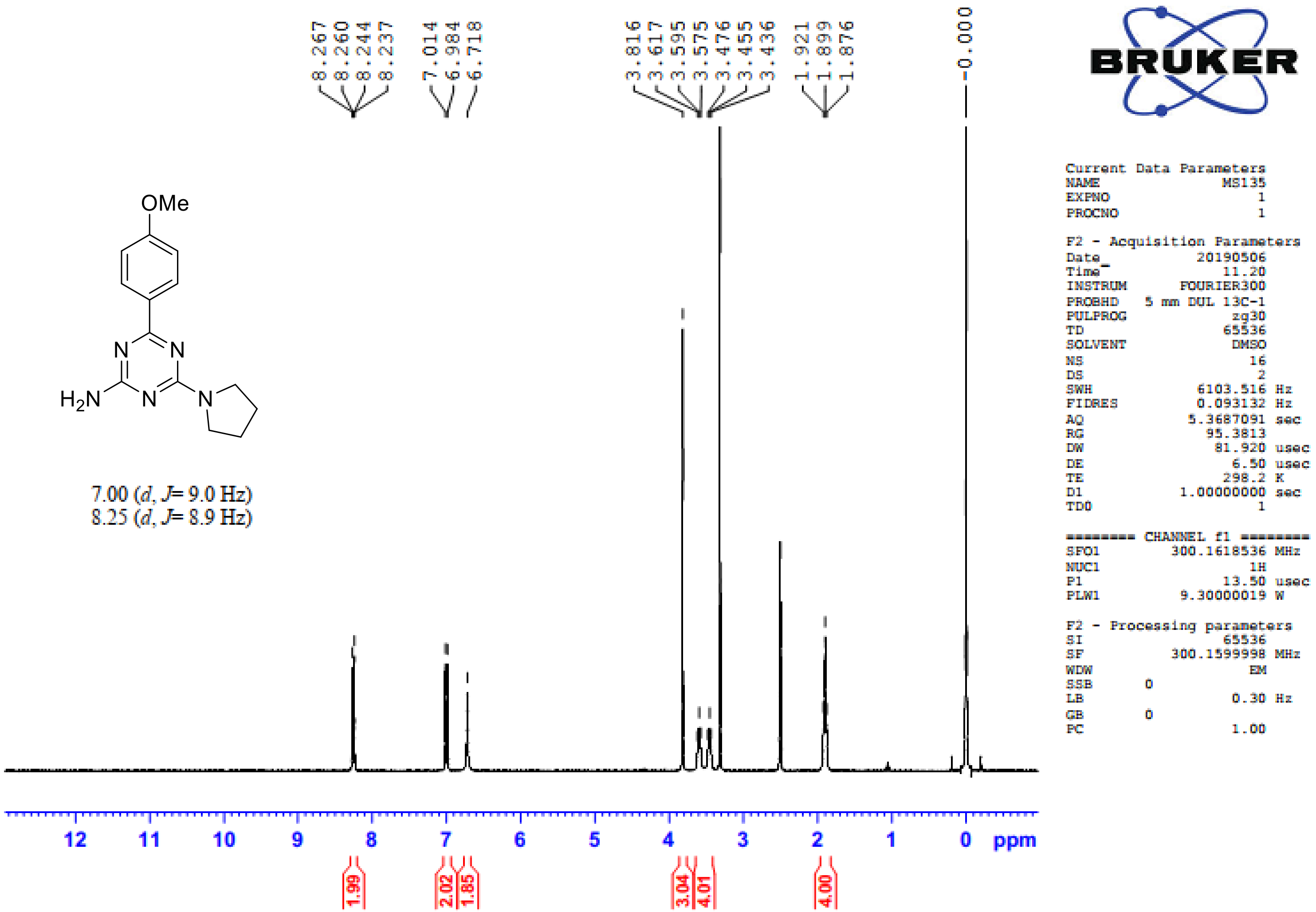

6-(4-Methoxyphenyl)-4-pyrrolidino-1,3,5-triazine-2-amine (**9**{*1,9*})

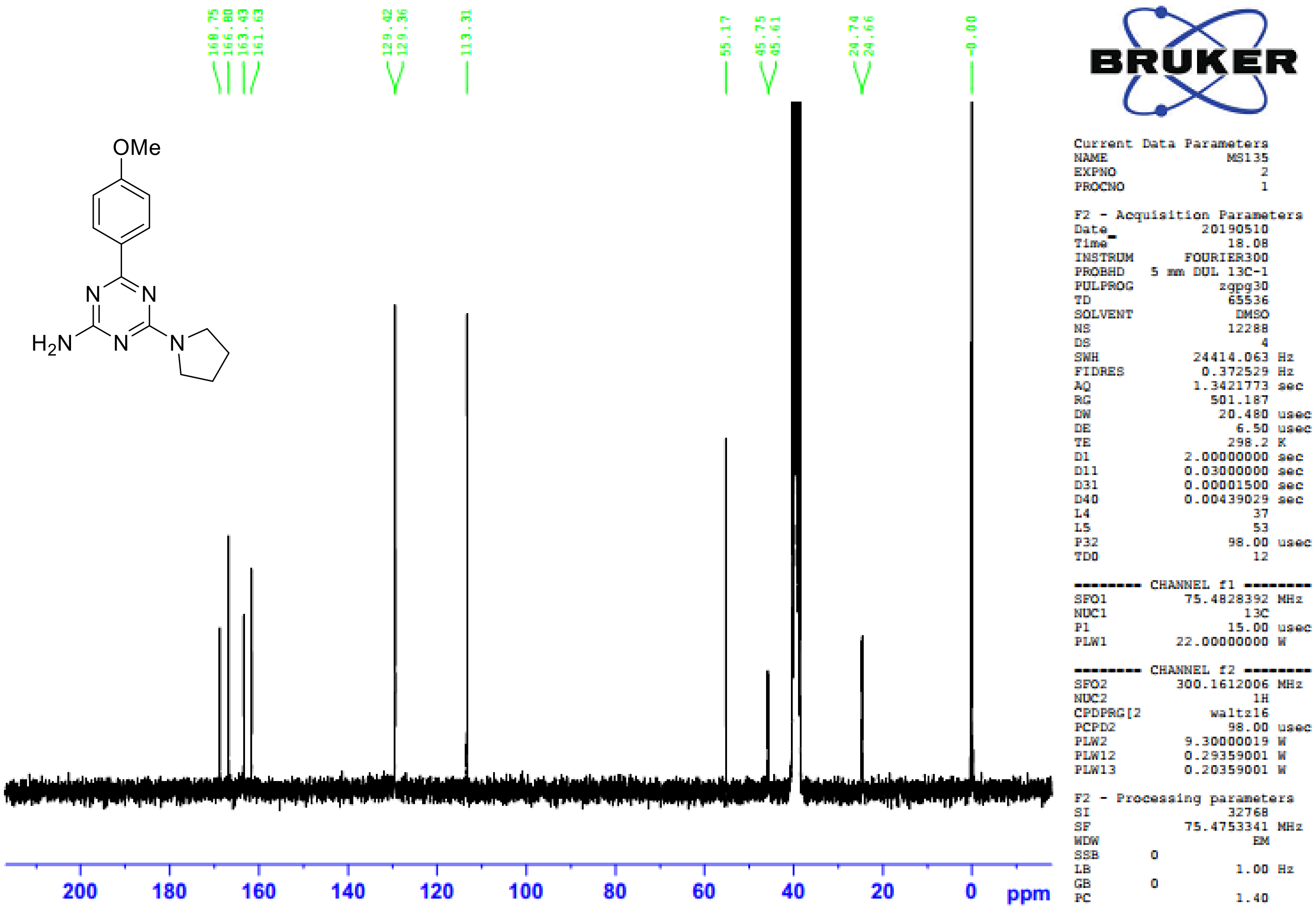

6-(3-Nitrophenyl)-4-pyrrolidino-1,3,5-triazine-2-amine (**9**{*1*,*10*})

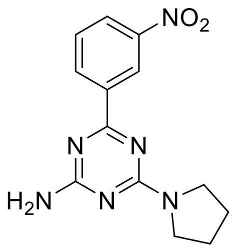

7.79 (*ddd*, *J*= 0.4, 7.8, 8.2 Hz)
8.38 (*ddd*, *J*= 1.1, 2.5, 8.2 Hz)
8.70 (*ddd*, *J*= 1.1, 1.5, 7.7 Hz)
9.06 (*ddd*, *J*= 0.4, 1.6, 2.5 Hz)

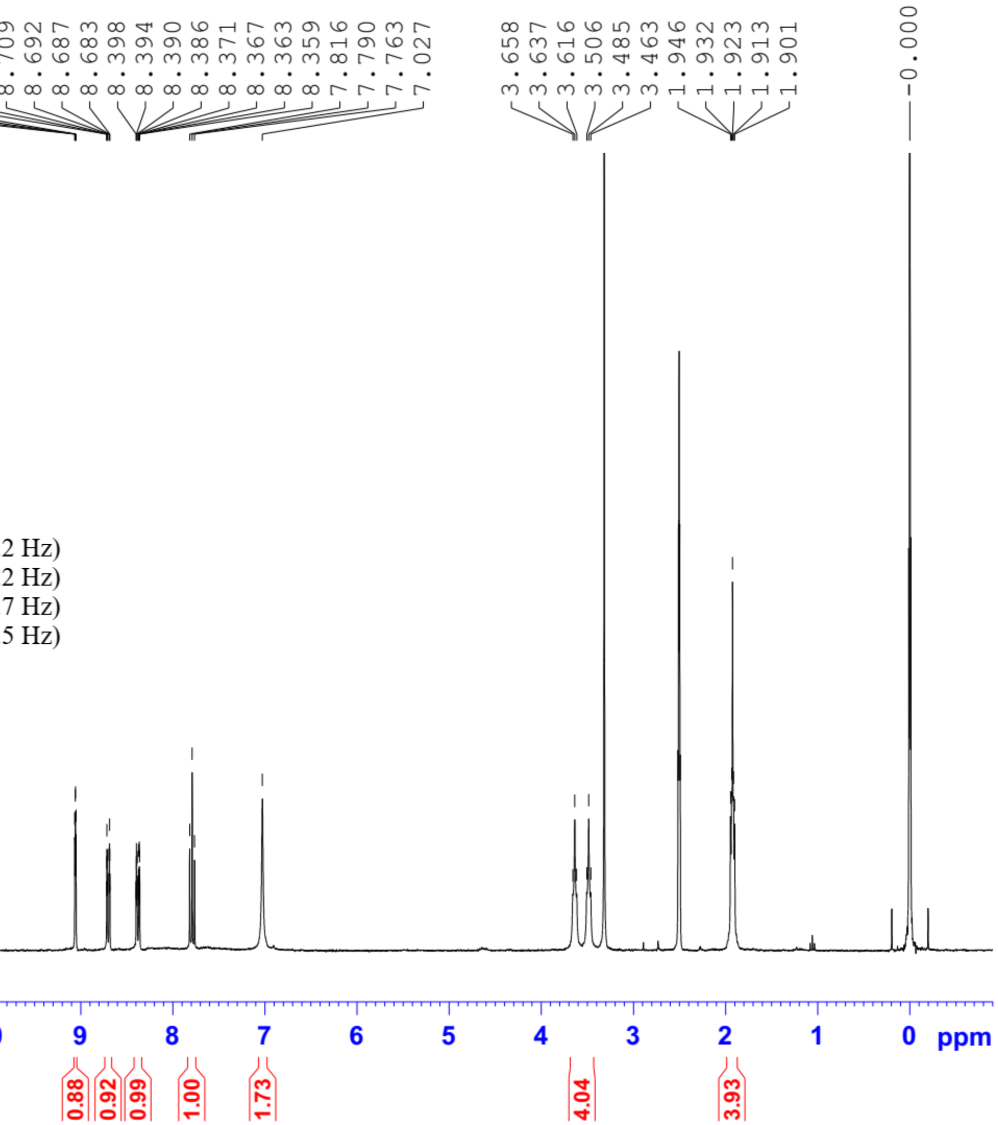


```
Current Data Parameters
NAME                MS130
EXPNO                   1
PROCNO                  1

F2 - Acquisition Parameters
Date_            20190506
Time                11.34
INSTRUM        FOURIER300
PROBHD   5 mm DUL 13C-1
PULPROG              zg30
TD                  65536
SOLVENT              DMSO
NS                     16
DS                      2
SWH              6103.516 Hz
FIDRES           0.093132 Hz
AQ              5.3687091 sec
RG                104.824
DW                 81.920 usec
DE                   6.50 usec
TE                  298.2 K
D1             1.00000000 sec
TD0                     1

======== CHANNEL f1 ========
SFO1          300.1618536 MHz
NUC1                   1H
P1                  13.50 usec
PLW1           9.30000019 W

F2 - Processing parameters
SI                  65536
SF            300.1599992 MHz
WDW                    EM
SSB      0
LB                   0.30 Hz
GB       0
PC                   1.00
```

6-(3-Nitrophenyl)-4-pyrrolidino-1,3,5-triazine-2-amine (**9**{*1*,*10*})

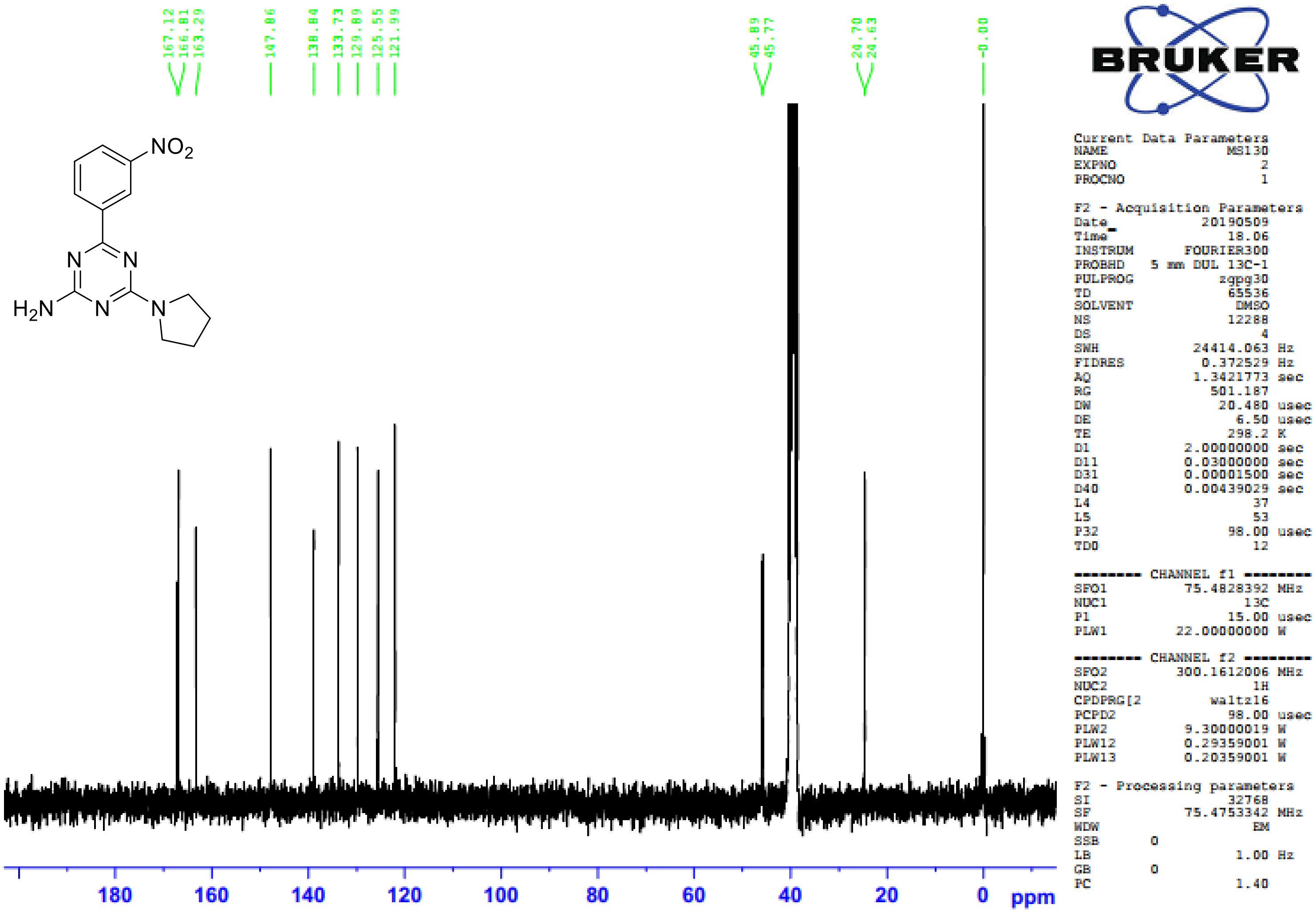

6-[4-(Trifluoromethyl)phenyl]-4-pyrrolidino-1,3,5-triazine-2-amine (**9**{*1*,*11*})

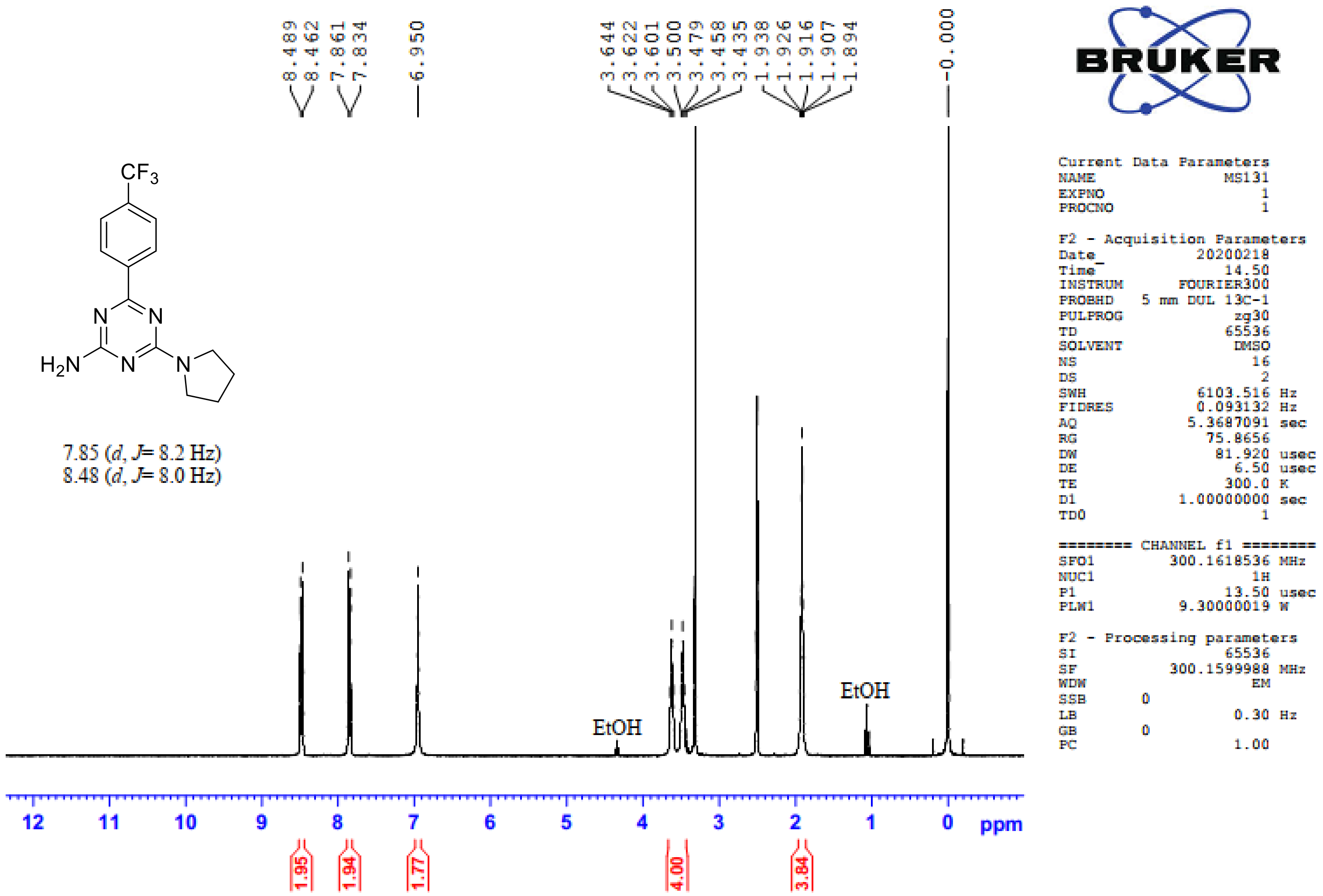

6-[4-(Trifluoromethyl)phenyl]-4-pyrrolidino-1,3,5-triazine-2-amine (**9**{*1,11*})

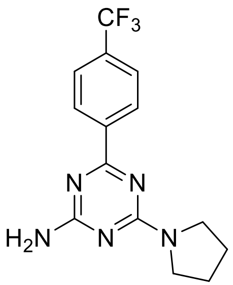

124.1 (*q*, *J*= 272.1 Hz)
125.1 (*q*, *J*= 3.5 Hz)
130.8 (*q*, *J*= 31.5 Hz)

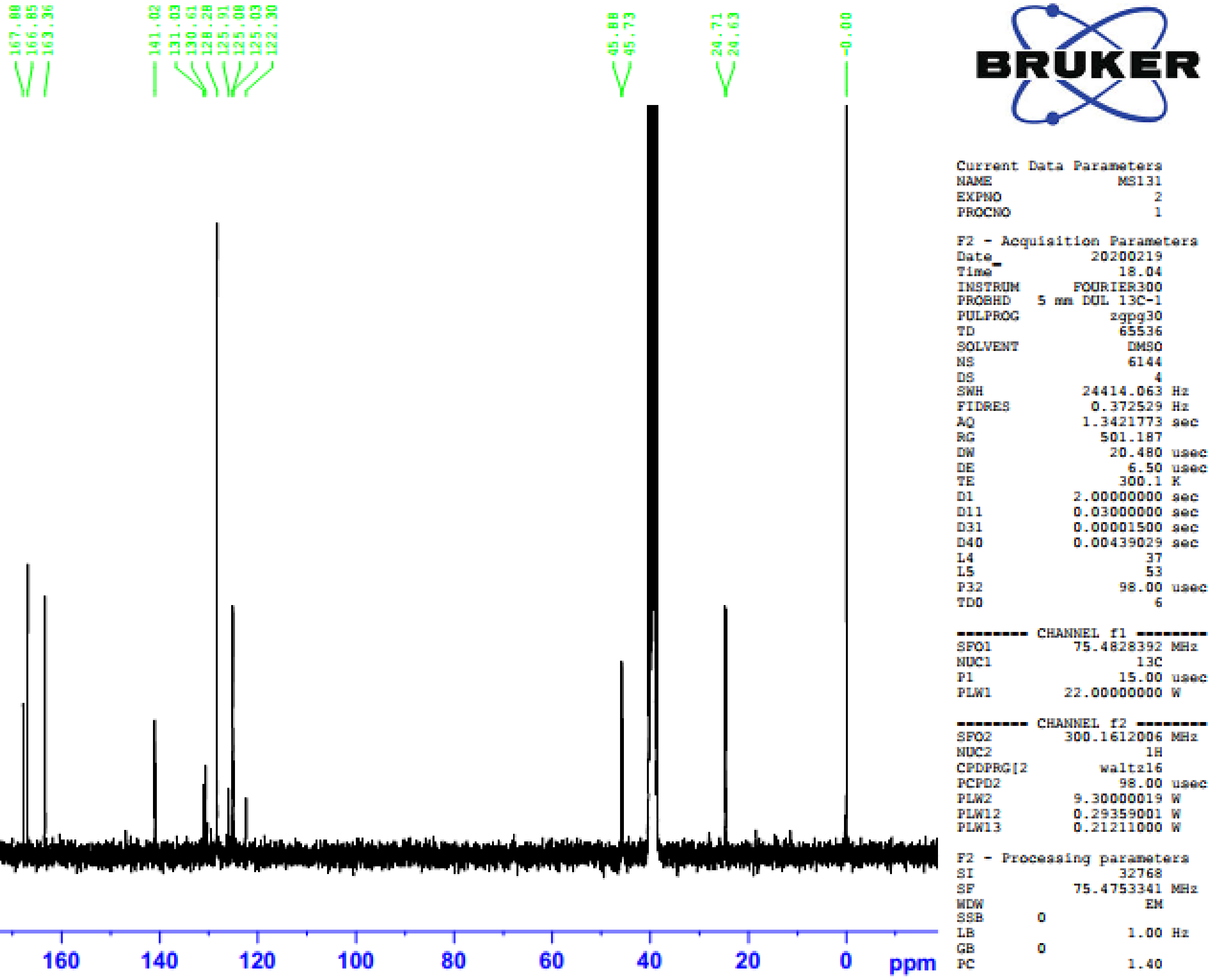

6-[4-(Trifluoromethoxy)phenyl]-4-pyrrolidino-1,3,5-triazine-2-amine (**9**{*1*,*12*})

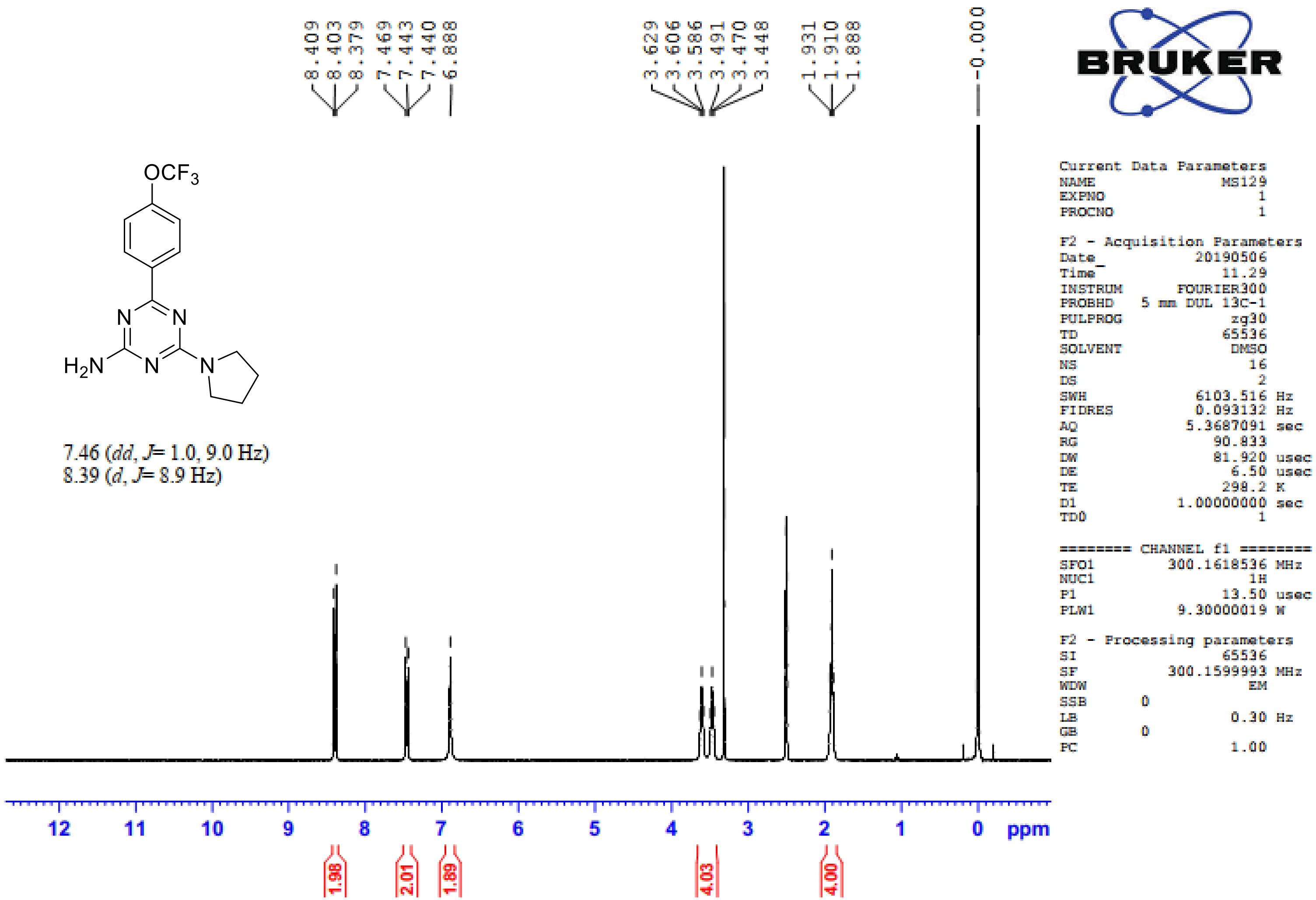

6-[4-(Trifluoromethoxy)phenyl]-4-pyrrolidino-1,3,5-triazine-2-amine (**9**{*1*,*12*})

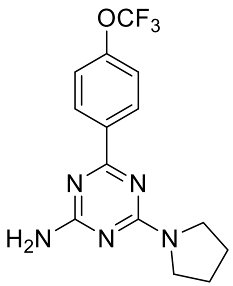

119.9 (*q*, *J*= 256.8 Hz)
150.3 (*q*, *J*= 1.9 Hz)

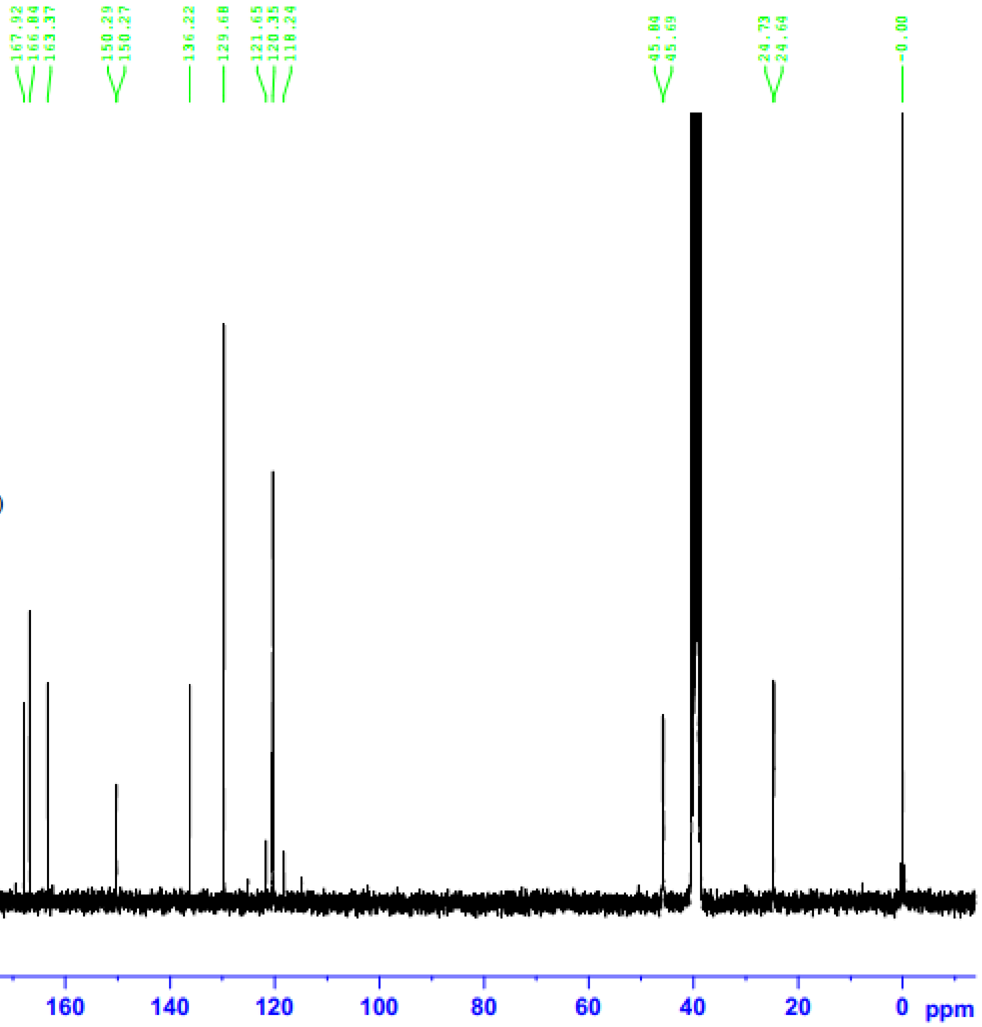


```
Current Data Parameters
NAME                MS129
EXPNO                   2
PROCNO                  1

F2 - Acquisition Parameters
Date_            20190508
Time                18.06
INSTRUM        FOURIER300
PROBHD   5 mm DUL 13C-1
PULPROG           zgpg30
TD                 65536
SOLVENT             DMSO
NS                 12288
DS                     4
SWH            24414.063 Hz
FIDRES          0.372529 Hz
AQ             1.3421773 sec
RG               501.187
DW                20.480 usec
DE                  6.50 usec
TE                 298.2 K
D1            2.00000000 sec
D11           0.03000000 sec
D31           0.00001500 sec
D40           0.00439029 sec
L4                    37
L5                    53
P32                98.00 usec
TD0                   12

======== CHANNEL f1 ========
SFO1         75.4828392 MHz
NUC1                13C
P1                15.00 usec
PLW1        22.00000000 W

======== CHANNEL f2 ========
SFO2        300.1612006 MHz
NUC2                 1H
CPDPRG[2        waltz16
PCPD2             98.00 usec
PLW2         9.30000019 W
PLW12        0.29359001 W
PLW13        0.20359001 W

F2 - Processing parameters
SI                 32768
SF           75.4753334 MHz
WDW                   EM
SSB      0
LB                  1.00 Hz
GB       0
PC                  1.40
```

6-(3-Phenoxyphenyl)-4-pyrrolidino-1,3,5-triazine-2-amine (**9**{*1*,*13*})

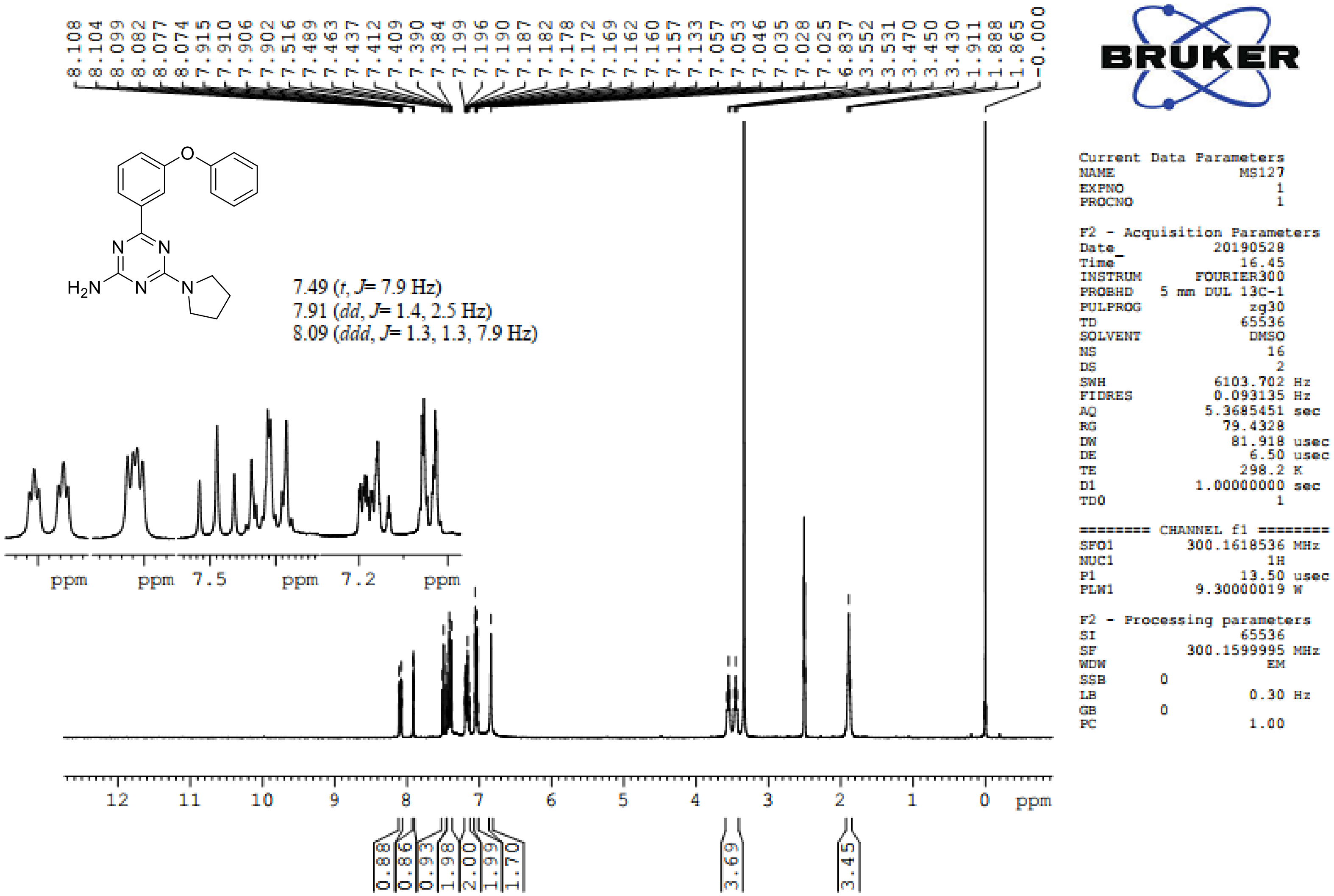

6-(3-Phenoxyphenyl)-4-pyrrolidino-1,3,5-triazine-2-amine (**9**{*1*,*13*})

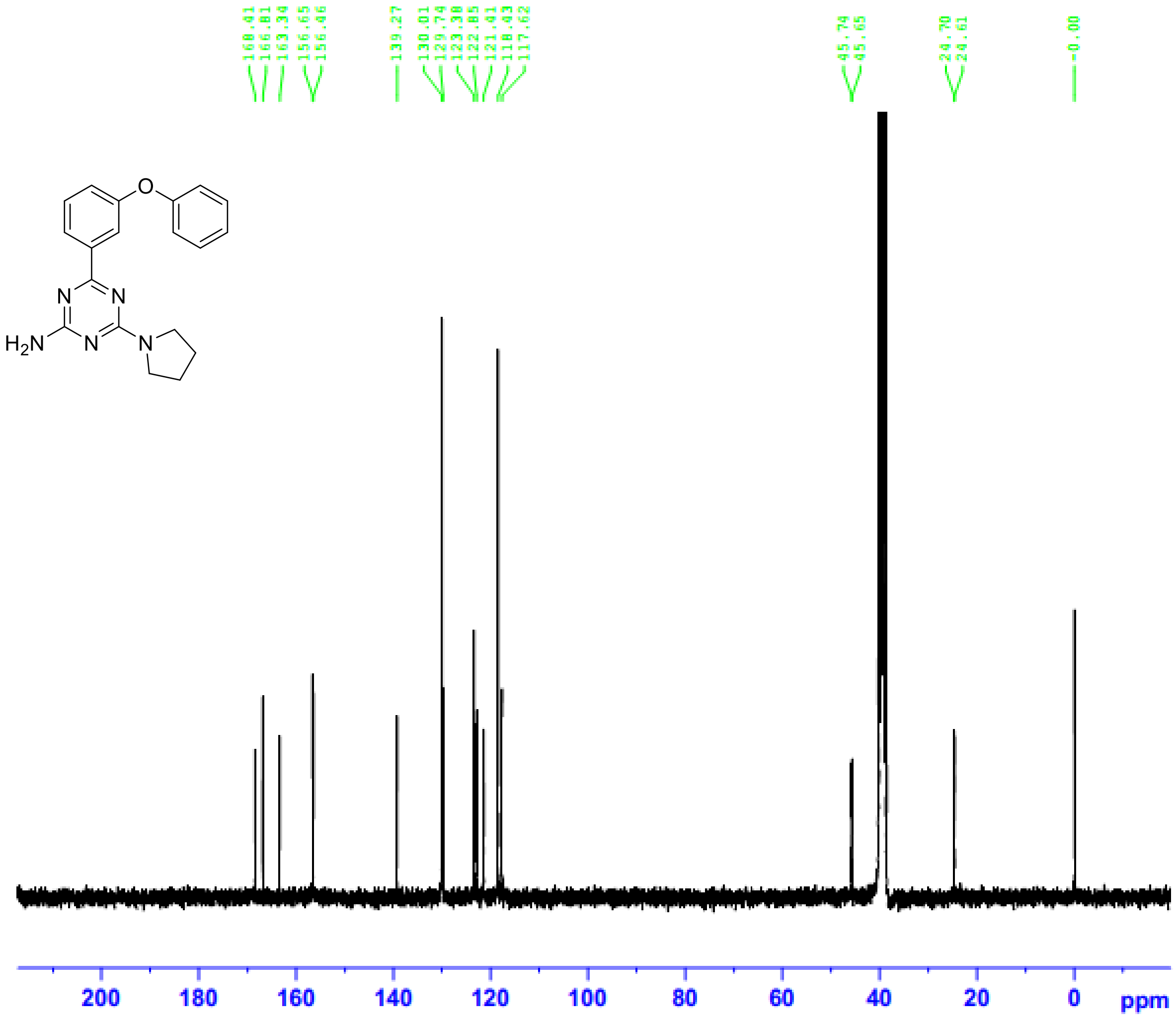


```
Current Data Parameters
NAME                 MS127
EXPNO                    2
PROCNO                   1

F2 - Acquisition Parameters
Date_             20190529
Time                 20.59
INSTRUM         FOURIER300
PROBHD     5 mm DUL 13C-1
PULPROG            zgpg30
TD                   65536
SOLVENT               DMSO
NS                   12288
DS                       4
SWH              24414.063 Hz
FIDRES            0.372529 Hz
AQ               1.3421773 sec
RG                 501.187
DW                  20.480 usec
DE                    6.50 usec
TE                   298.2 K
D1              2.00000000 sec
D11             0.03000000 sec
D31             0.00001500 sec
D40             0.00439029 sec
L4                      37
L5                      53
P32                  98.00 usec
TD0                     12

======== CHANNEL f1 ========
SFO1            75.4828392 MHz
NUC1                   13C
P1                   15.00 usec
PLW1           22.00000000 W

======== CHANNEL f2 ========
SFO2           300.1612006 MHz
NUC2                    1H
CPDPRG[2           waltz16
PCPD2                98.00 usec
PLW2            9.30000019 W
PLW12           0.29359001 W
PLW13           0.20359001 W

F2 - Processing parameters
SI                   32768
SF              75.4753350 MHz
WDW                     EM
SSB          0
LB                    1.00 Hz
GB           0
PC                    1.40
```

6-[4-(Benzyloxy)phenyl]-4-pyrrolidino-1,3,5-triazine-2-amine (**9**{*1*,*15*})

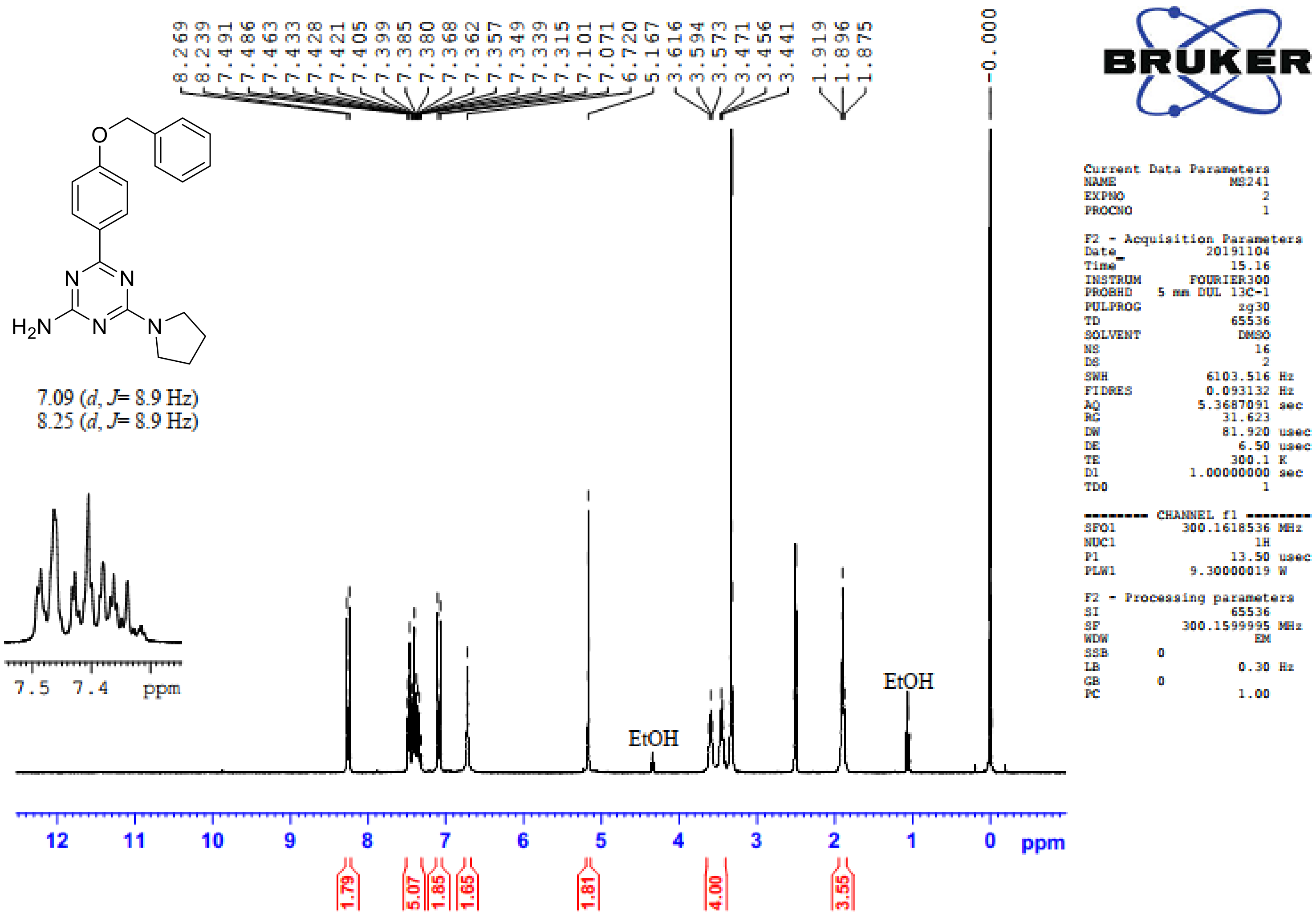

6-[4-(Benzyloxy)phenyl]-4-pyrrolidino-1,3,5-triazine-2-amine (**9**{*1*,*15*})

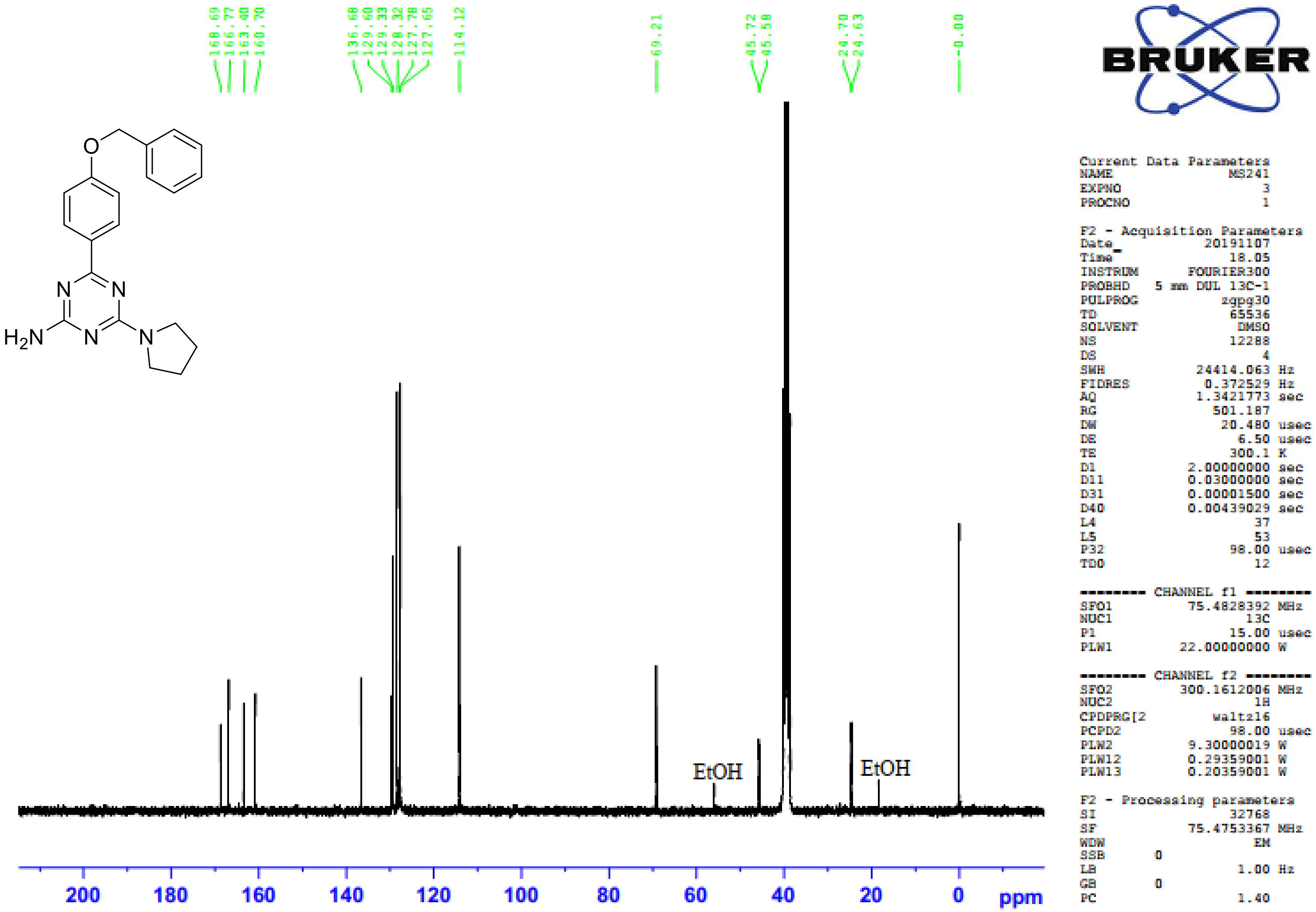

6-(3,4-Dimethoxyphenyl)-4-pyrrolidino-1,3,5-triazine-2-amine (**9**{*1,16*})

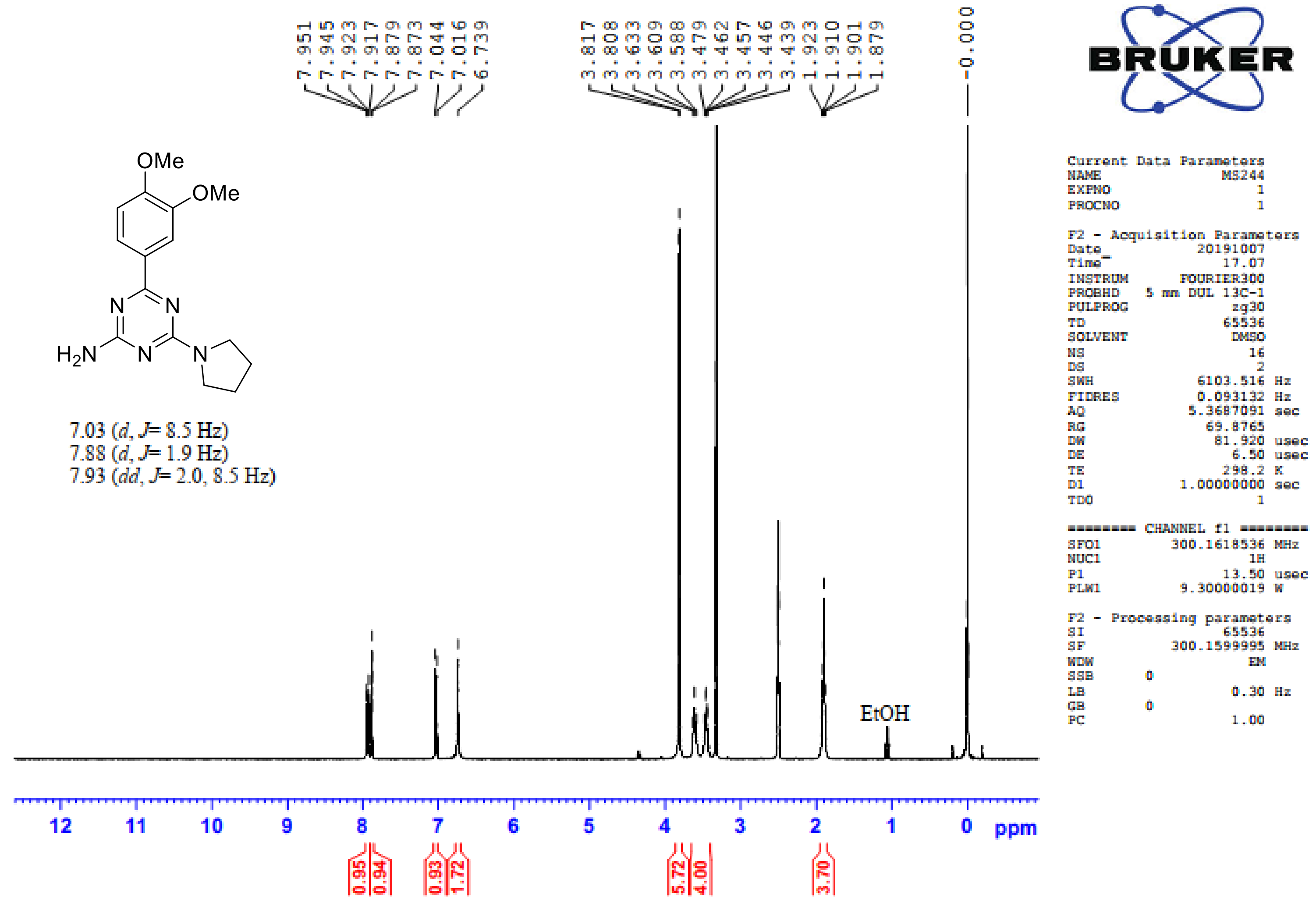

6-(3,4-Dimethoxyphenyl)-4-pyrrolidino-1,3,5-triazine-2-amine (**9**{*1,16*})

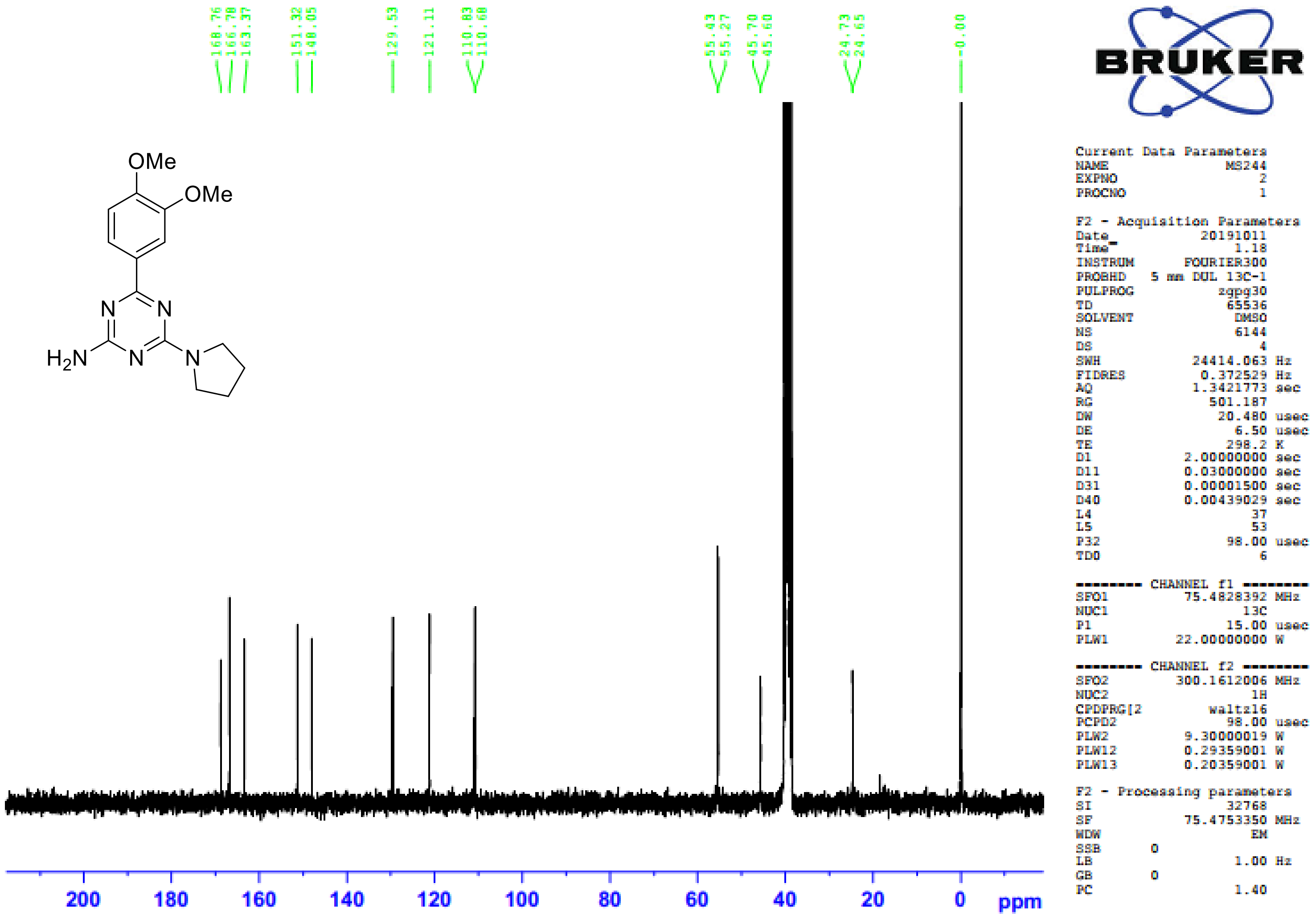

6-(3,4,5-Trimethoxyphenyl)-4-pyrrolidino-1,3,5-triazine-2-amine (**9**{*1,17*})

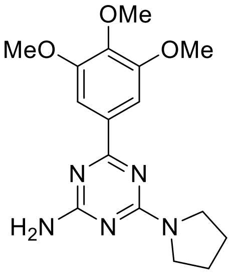

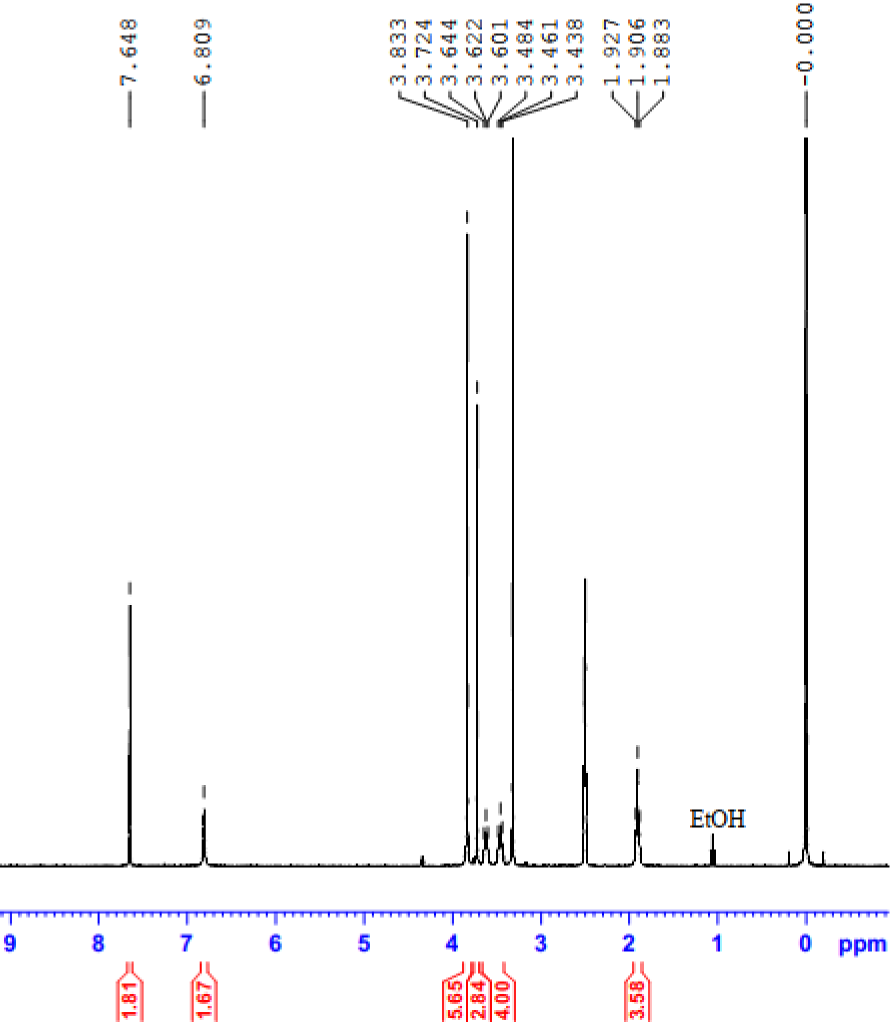


```
Current Data Parameters
NAME                 MS243
EXPNO                    1
PROCNO                   1

F2 - Acquisition Parameters
Date_             20191007
Time                 17.03
INSTRUM         FOURIER300
PROBHD     5 mm DUL 13C-1
PULPROG               zg30
TD                   65536
SOLVENT               DMSO
NS                      16
DS                       2
SWH               6103.516 Hz
FIDRES            0.093132 Hz
AQ               5.3687091 sec
RG                 88.2542
DW                  81.920 usec
DE                    6.50 usec
TE                   298.2 K
D1              1.00000000 sec
TD0                      1

======== CHANNEL f1 ========
SFO1           300.1618536 MHz
NUC1                    1H
P1                   13.50 usec
PLW1            9.30000019 W

F2 - Processing parameters
SI                   65536
SF             300.1599996 MHz
WDW                     EM
SSB      0
LB                    0.30 Hz
GB       0
PC                    1.00
```

6-(3,4,5-Trimethoxyphenyl)-4-pyrrolidino-1,3,5-triazine-2-amine (**9**{*1*,*17*})

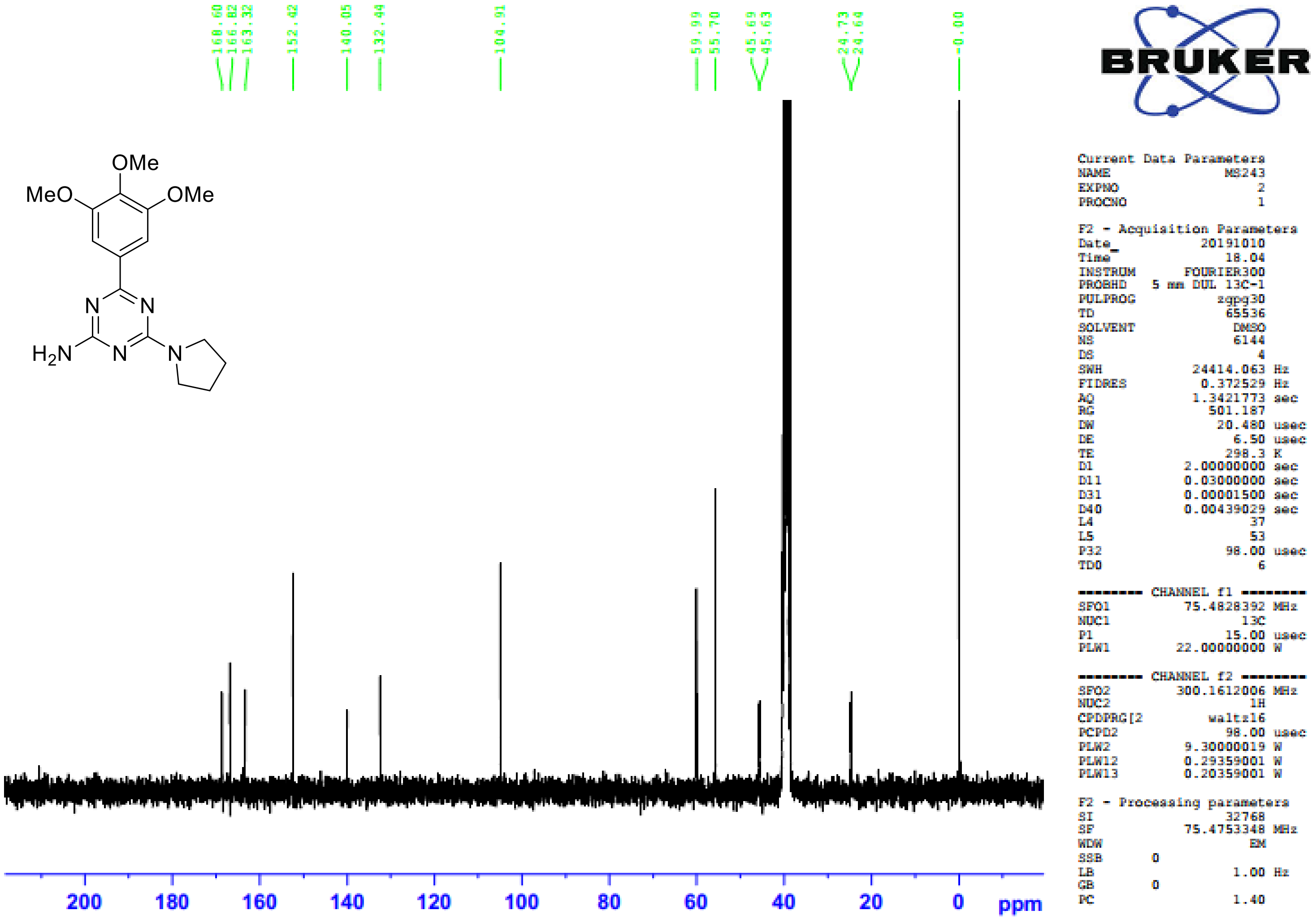

4-Pyrrolidino-6-(2-thienyl)-1,3,5-triazine-2-amine (**9**{*1,18*})

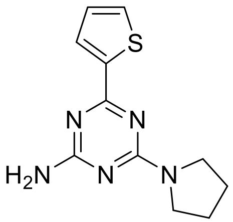

7.15 (*dd*, *J*= 3.7, 5.0 Hz)
7.69 (*dd*, *J*= 1.3, 5.0 Hz)
7.85 (*dd*, *J*= 1.3, 3.7 Hz)

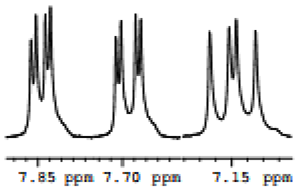

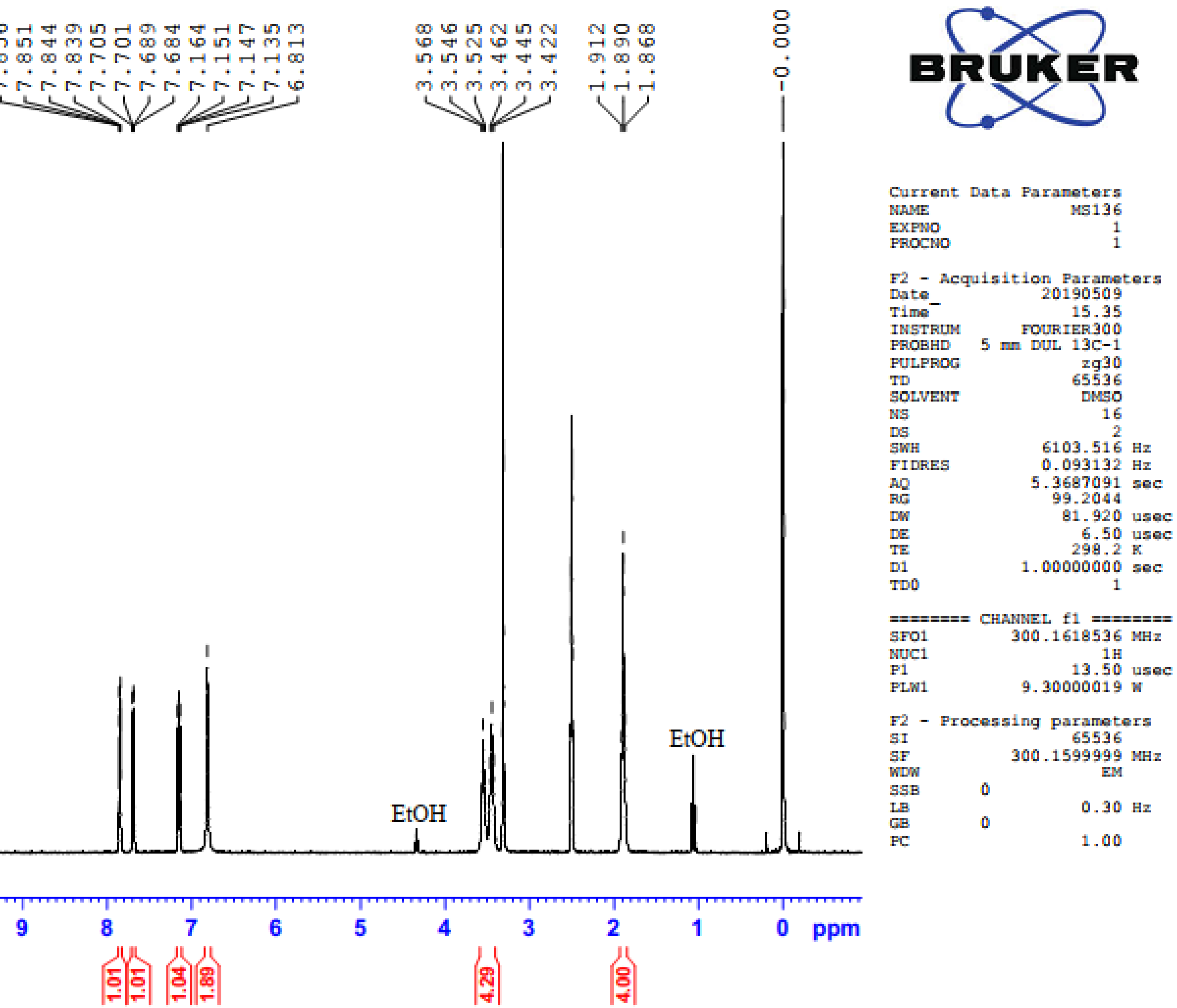

4-Pyrrolidino-6-(2-thienyl)-1,3,5-triazine-2-amine (**9**{*1,18*})

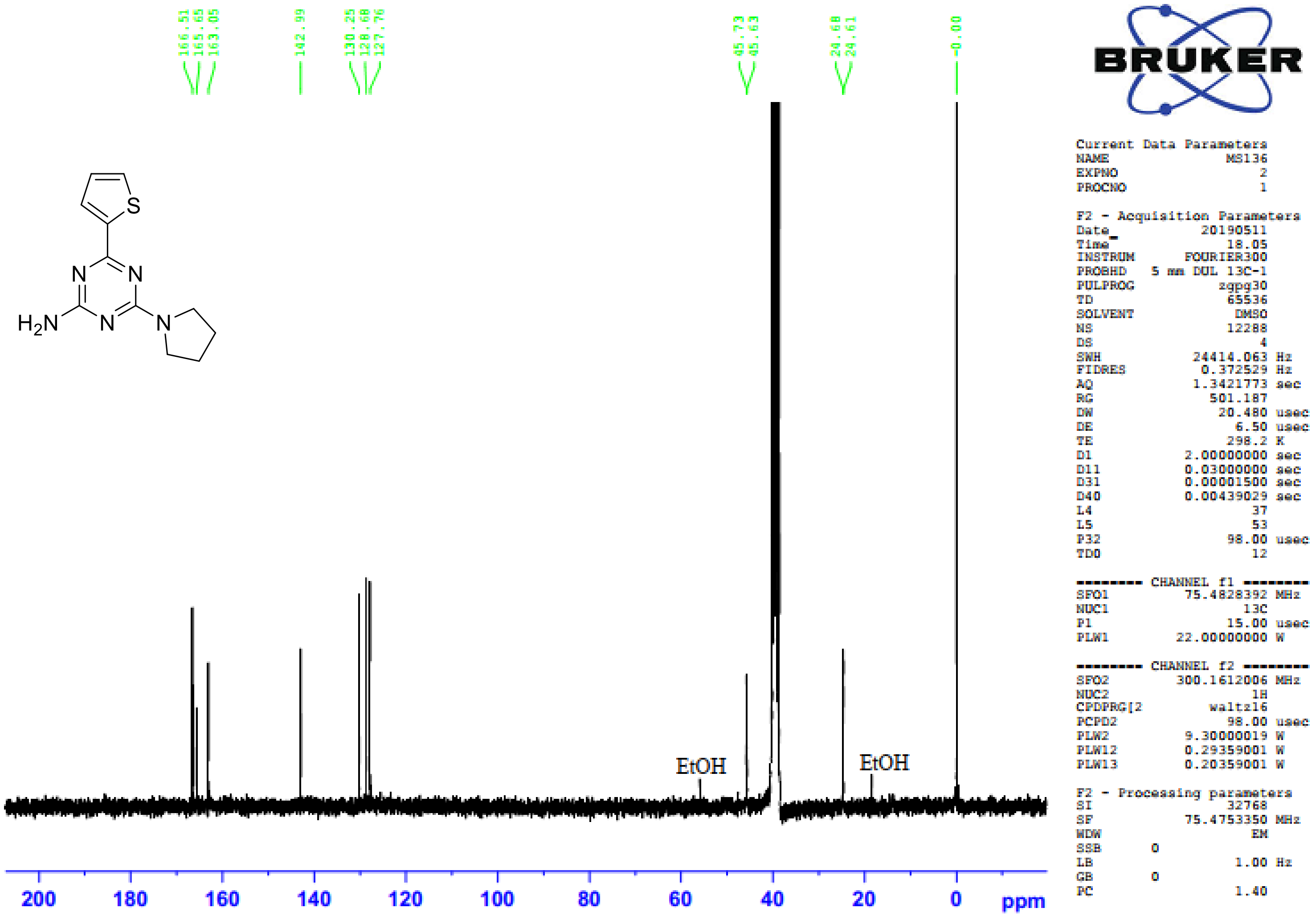

6-(3-Fluorophenyl)-4-piperidino-1,3,5-triazine-2-amine (**9**{*2*,*2*})

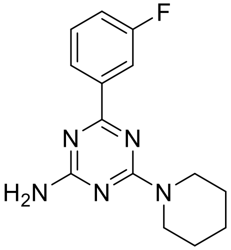

7.37 (*dddd*, *J* = 0.9, 2.8, 8.5, 8.5 Hz)
7.53 (*td*, *J* = 8.0, 6.0 Hz)
7.98 (*ddd*, *J* = 1.4, 2.6, 10.6 Hz)
8.13 (*ddd*, *J* = 1.1, 1.1, 7.8 Hz)

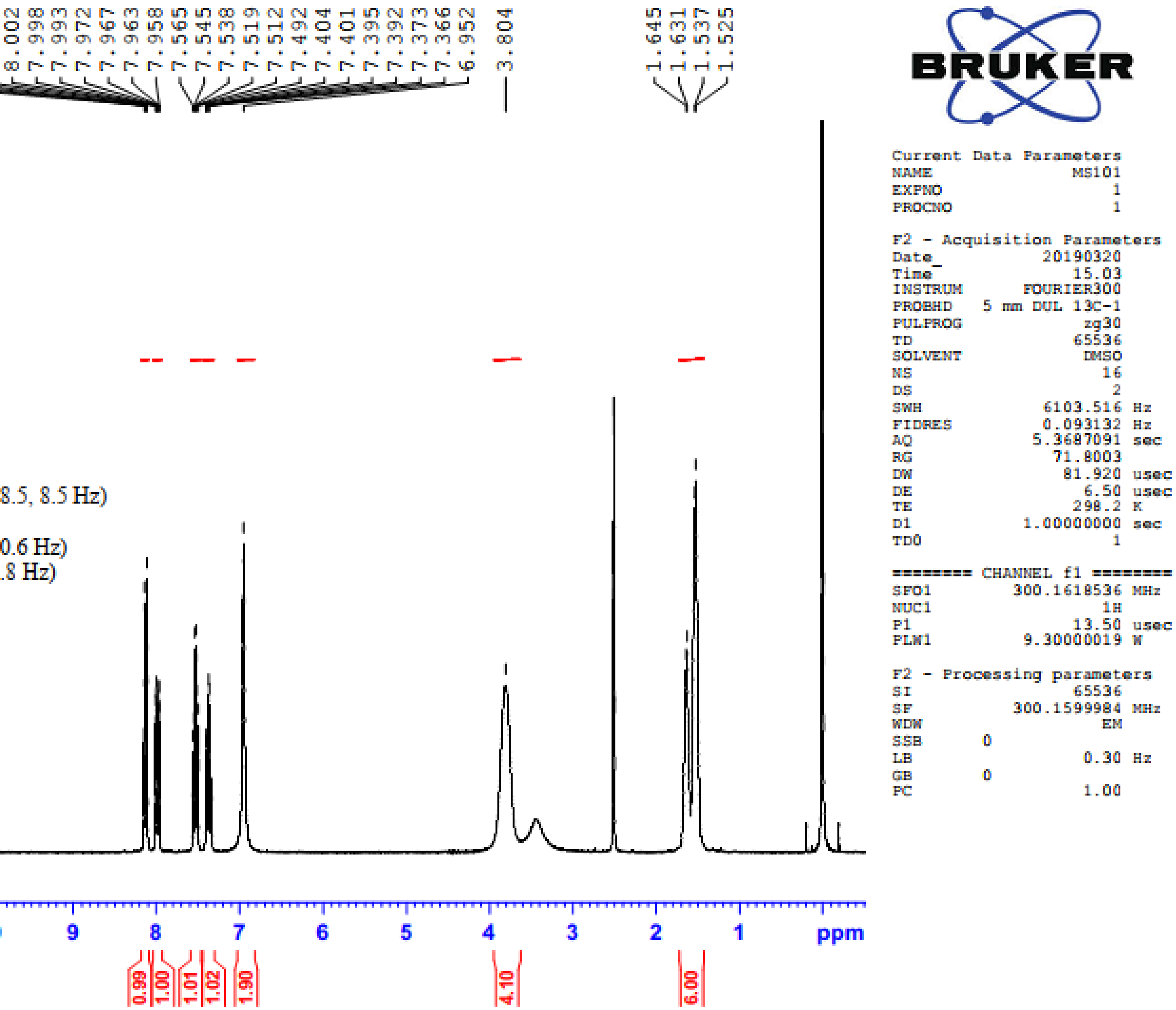

6-(3-Fluorophenyl)-4-piperidino-1,3,5-triazine-2-amine (**9**{*2*,*2*})

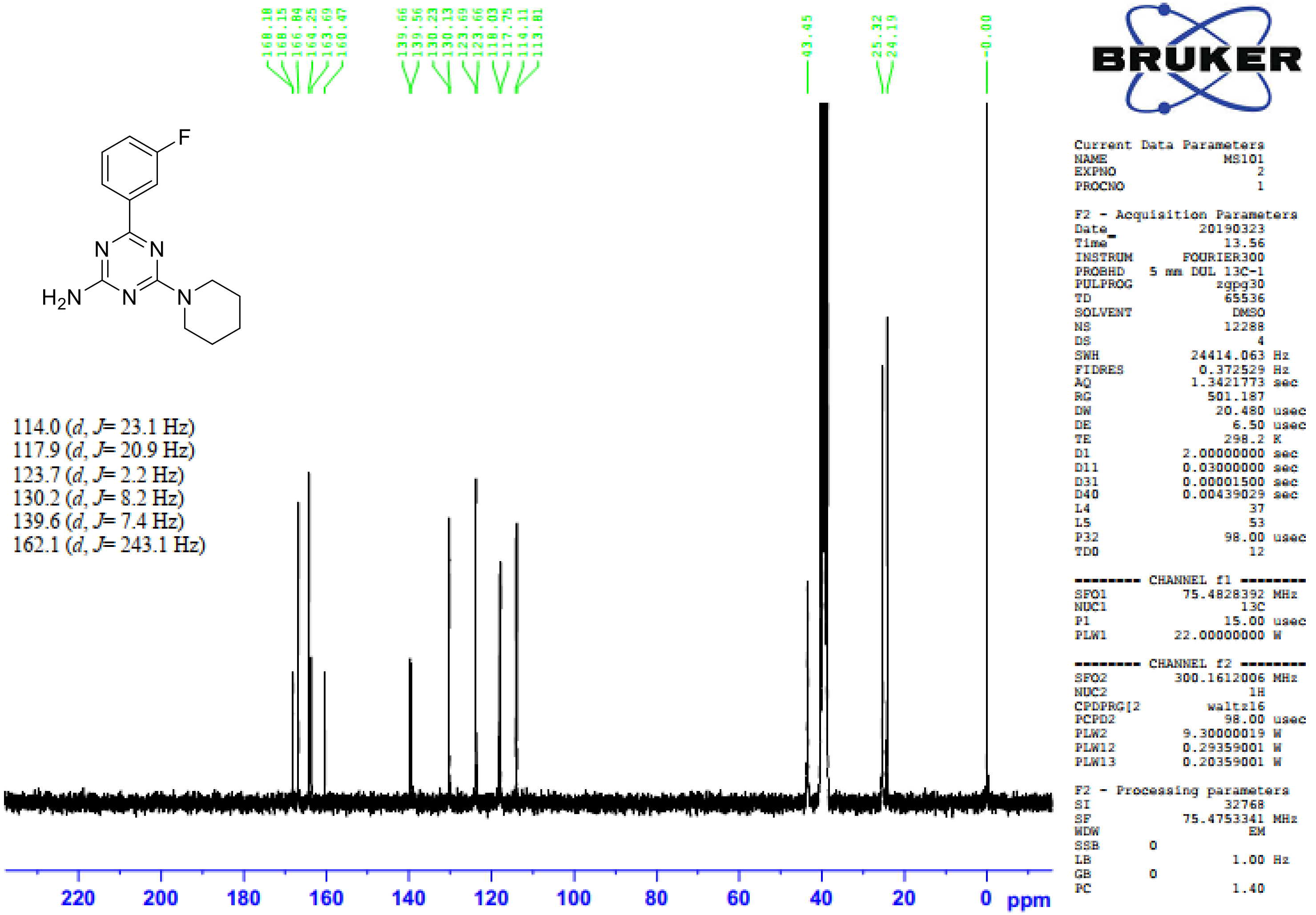

114.0 (*d*, *J*= 23.1 Hz)
117.9 (*d*, *J*= 20.9 Hz)
123.7 (*d*, *J*= 2.2 Hz)
130.2 (*d*, *J*= 8.2 Hz)
139.6 (*d*, *J*= 7.4 Hz)
162.1 (*d*, *J*= 243.1 Hz)

6-(4-Fluorophenyl)-4-piperidino-1,3,5-triazine-2-amine (**9**{*2*,*3*})

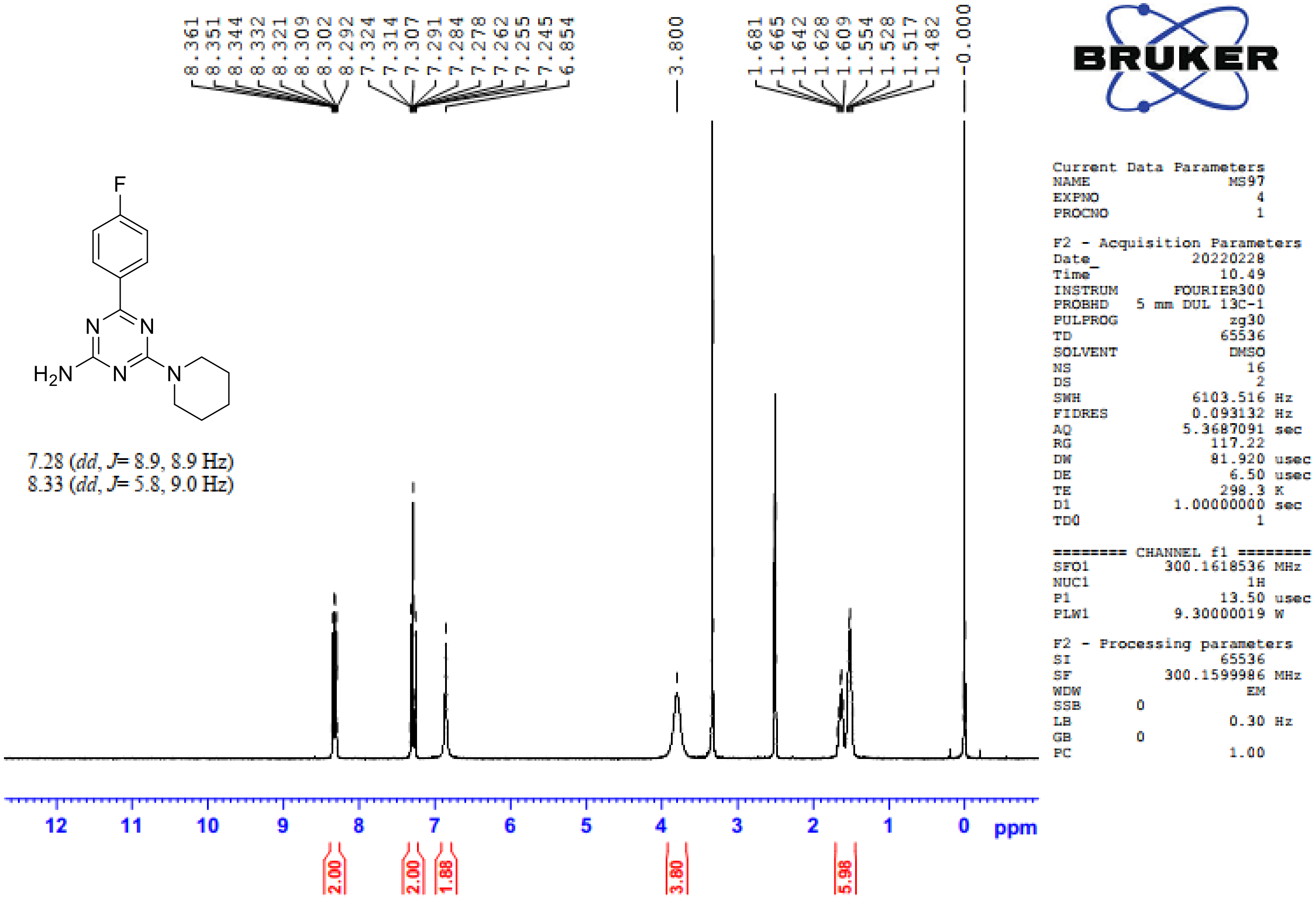

7.28 (*dd*, *J*= 8.9, 8.9 Hz)
8.33 (*dd*, *J*= 5.8, 9.0 Hz)

6-(4-Fluorophenyl)-4-piperidino-1,3,5-triazine-2-amine (**9**{*2*,*3*})

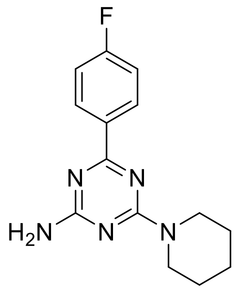

115.0 (*d*, *J*= 21.6 Hz)
130.0 (*d*, *J*= 8.9 Hz)
133.5 (*d*, *J*= 3.0 Hz)
164.0 (*d*, *J*= 248.1 Hz)

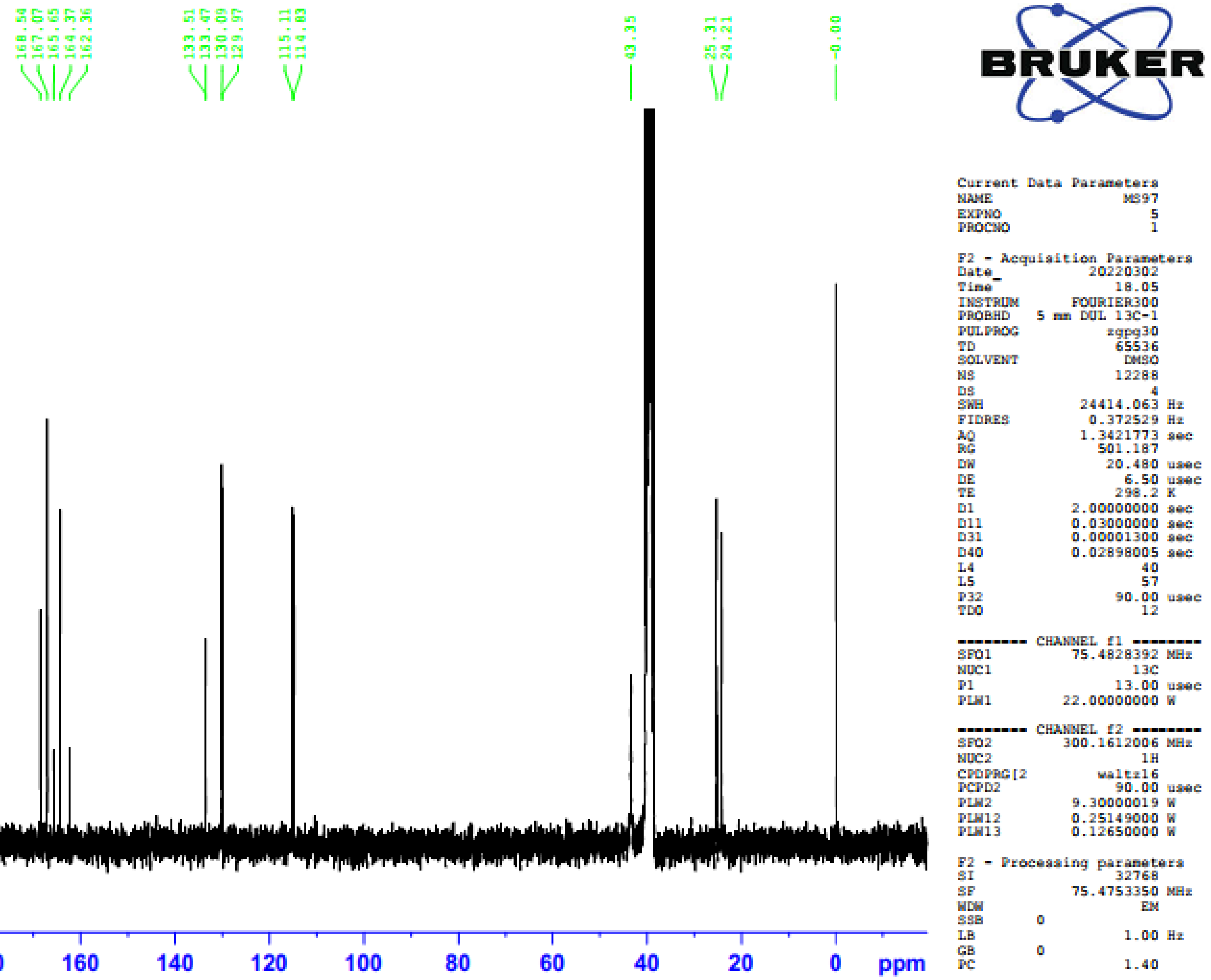

6-(4-Chlorophenyl)-4-piperidino-1,3,5-triazine-2-amine (**9**{*2*,*4*})

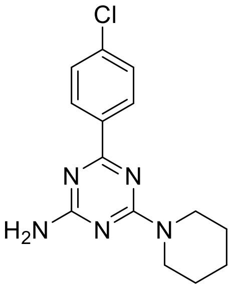

7.54 (*d*, *J*= 8.7 Hz)
8.28 (*d*, *J*= 8.7 Hz)

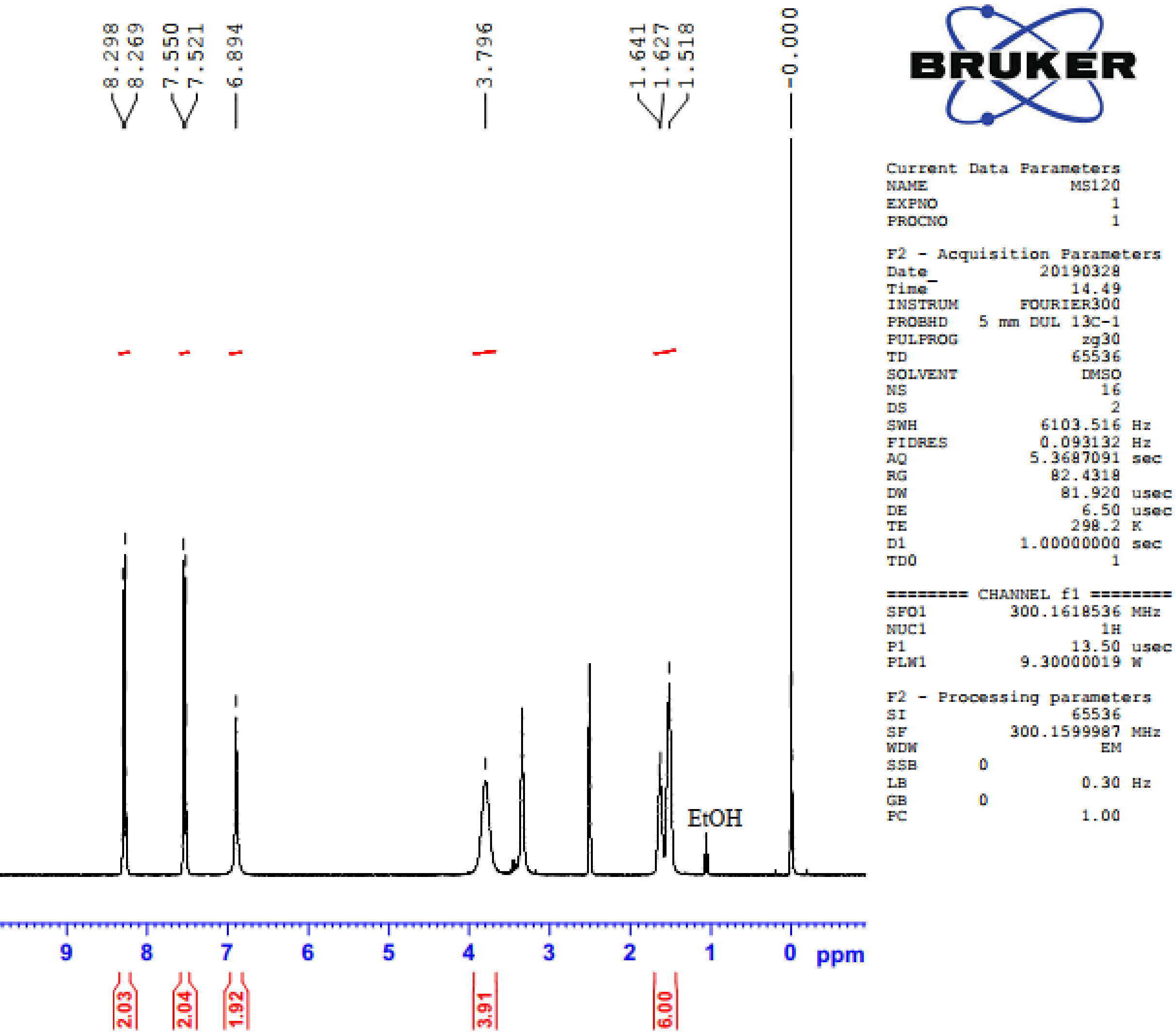

6-(4-Chlorophenyl)-4-piperidino-1,3,5-triazine-2-amine (**9**{*2*,*4*})

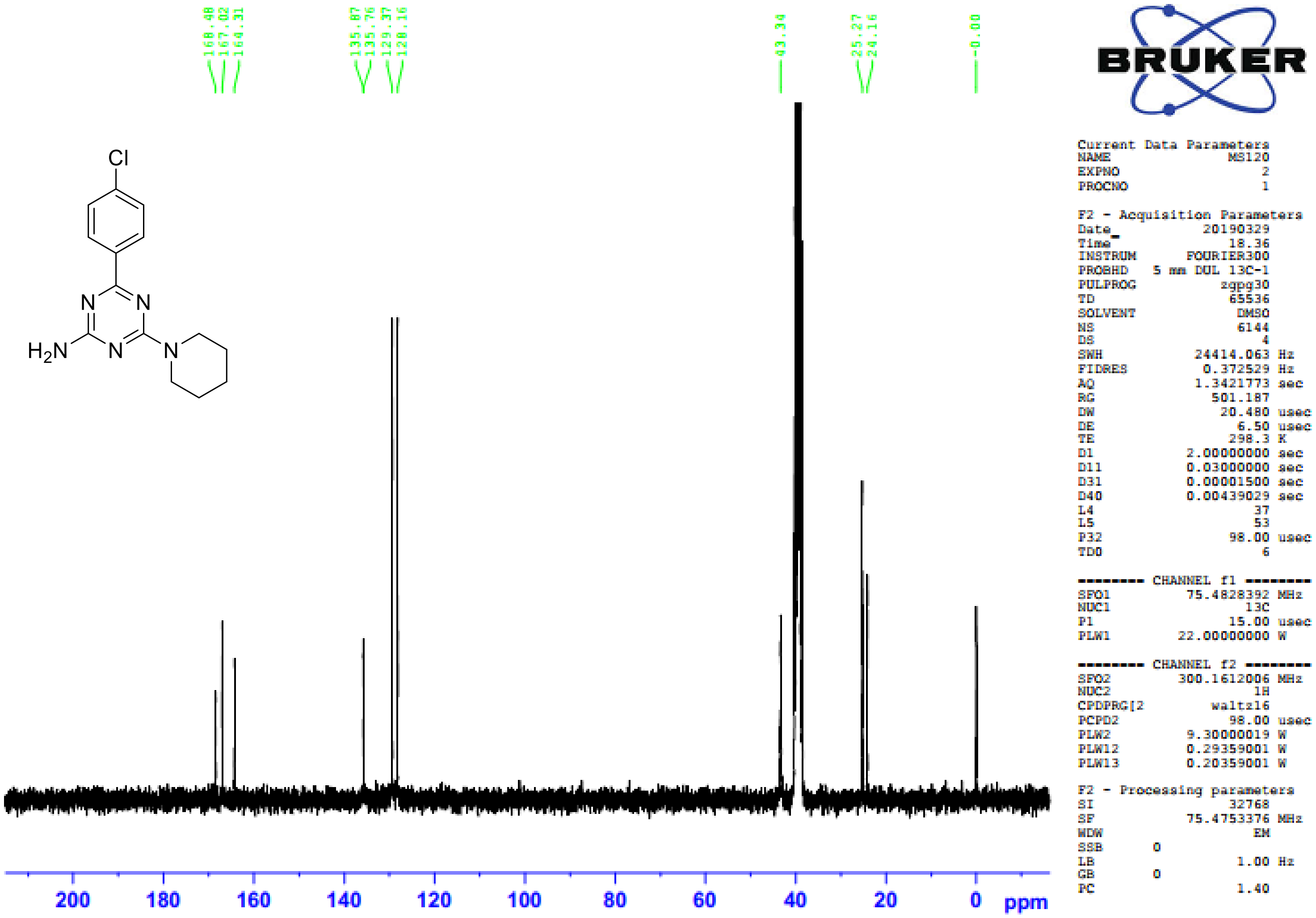

6-(4-Methylphenyl)-4-piperidino-1,3,5-triazine-2-amine (**9**{*2*,*6*})

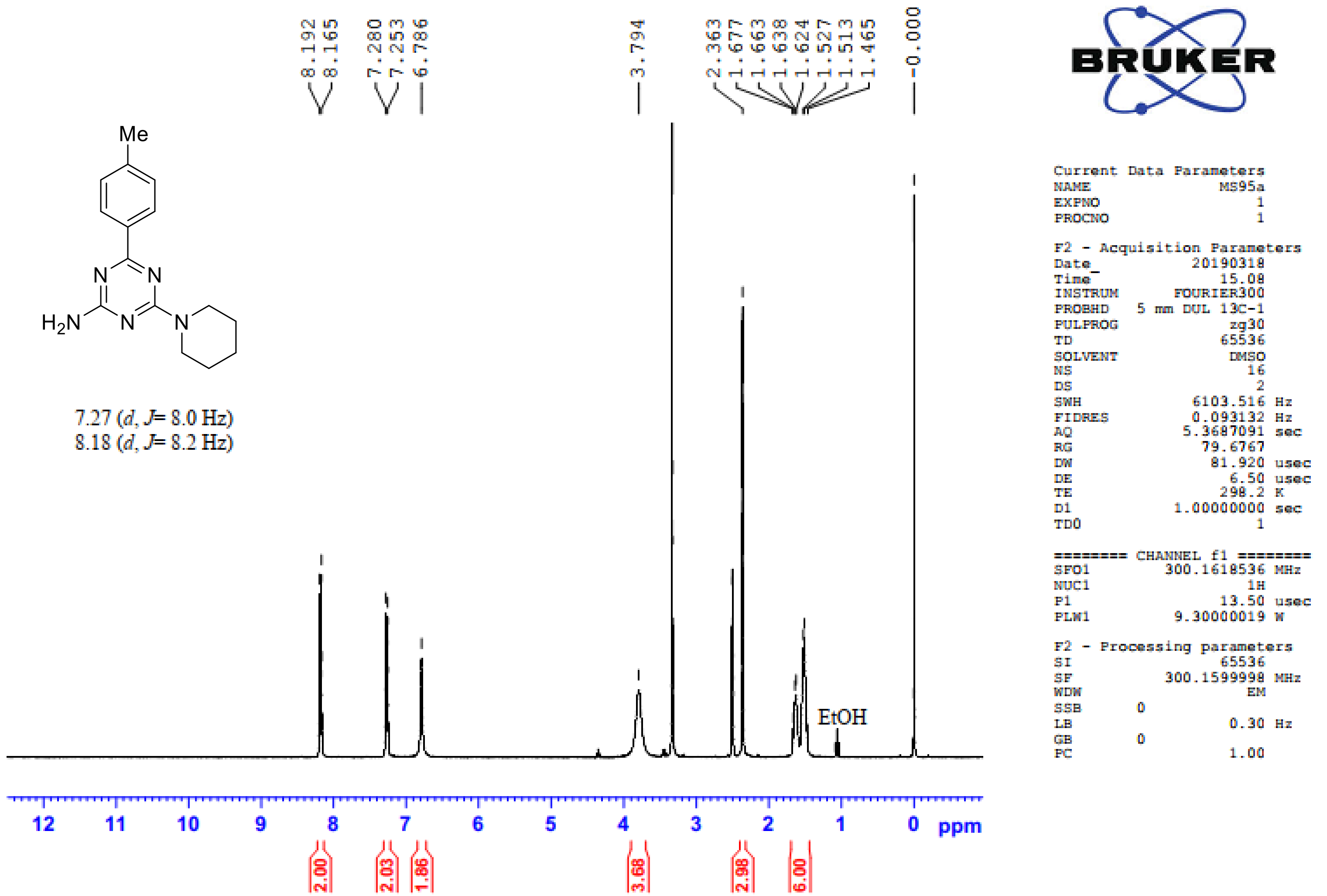

6-(4-Methylphenyl)-4-piperidino-1,3,5-triazine-2-amine (**9**{*2,6*})

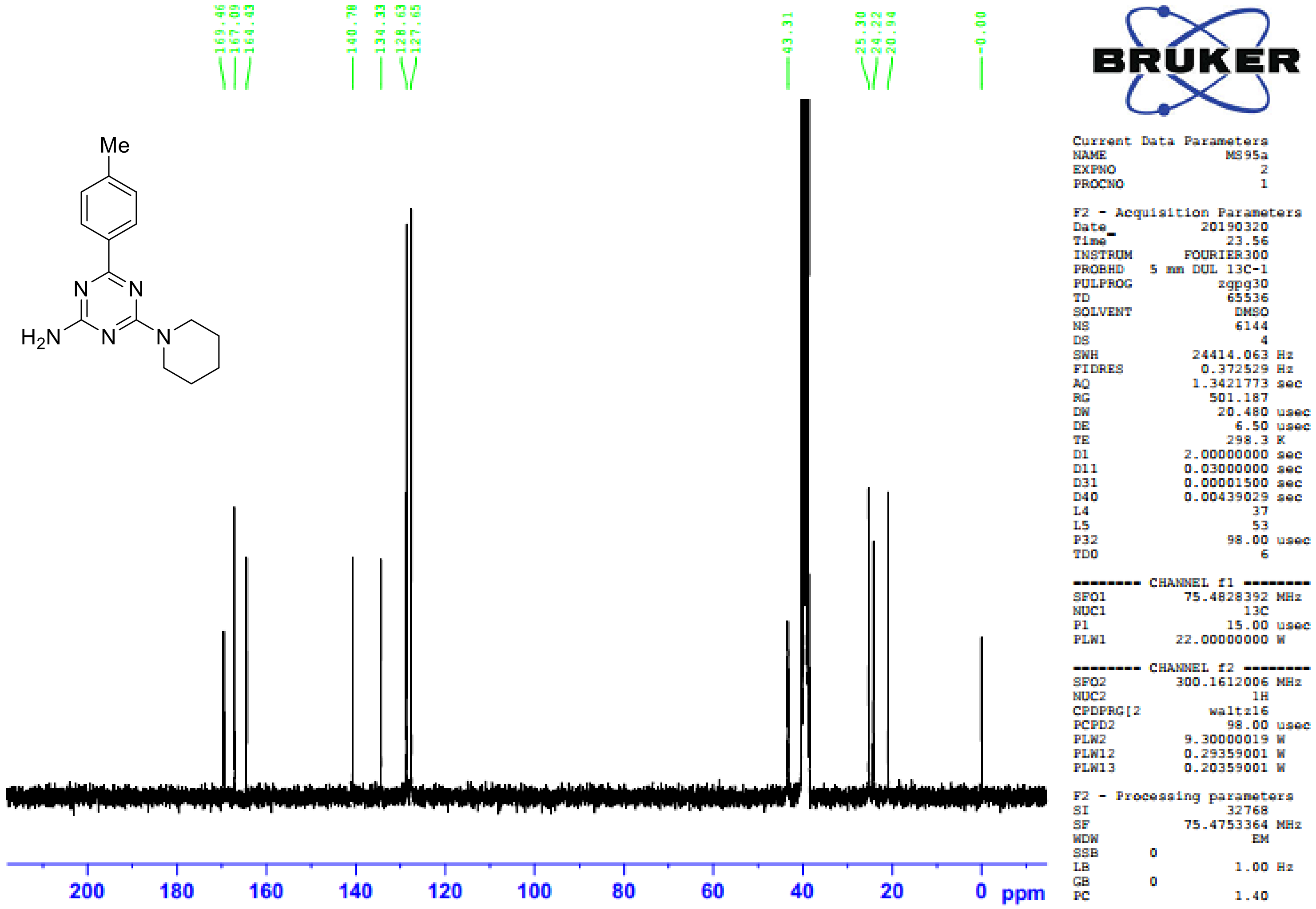

6-(4-Methoxyphenyl)-4-piperidino-1,3,5-triazine-2-amine (**9**{*2*,*9*})

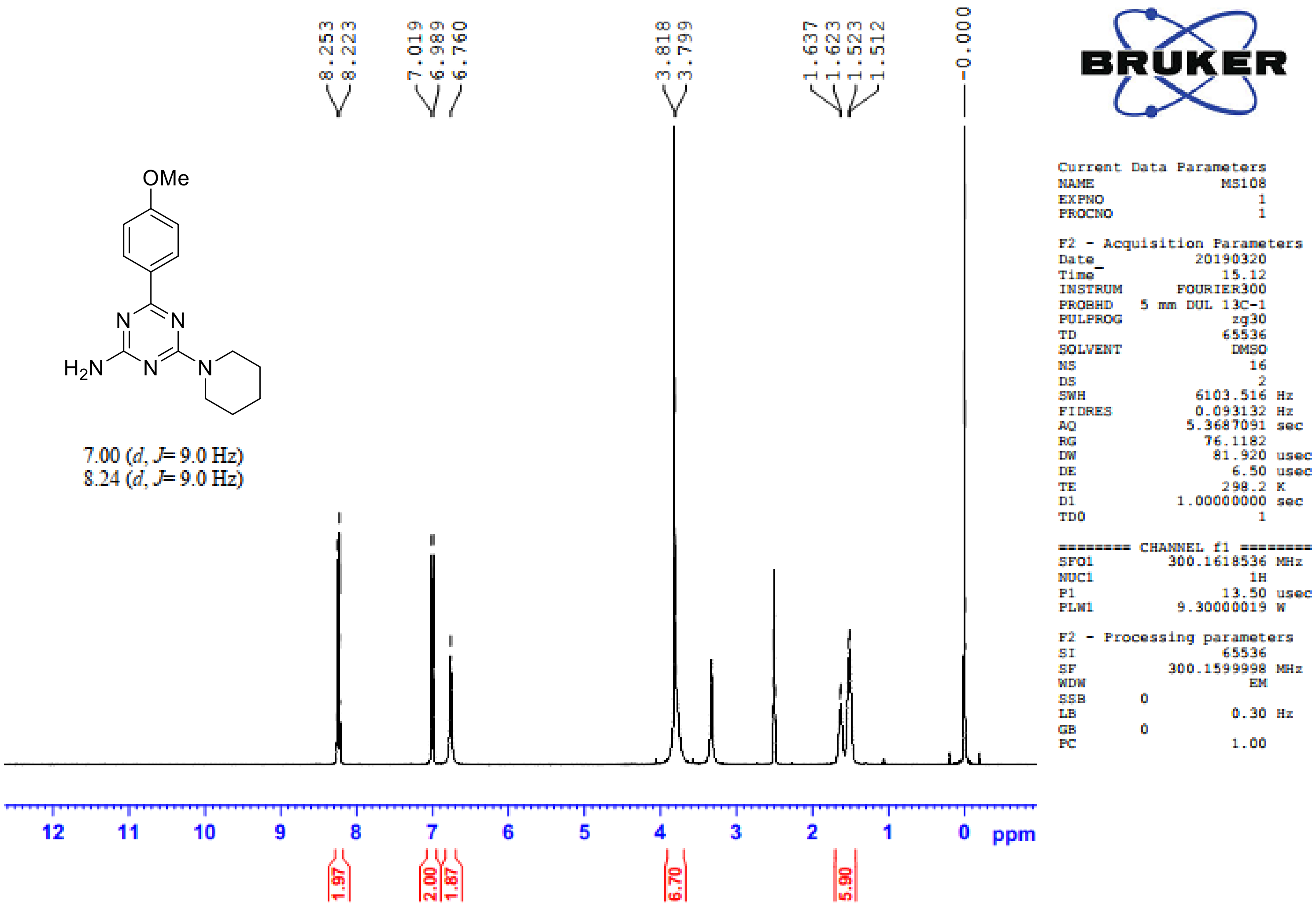

6-(4-Methoxyphenyl)-4-piperidino-1,3,5-triazine-2-amine (**9**{*2*,*9*})

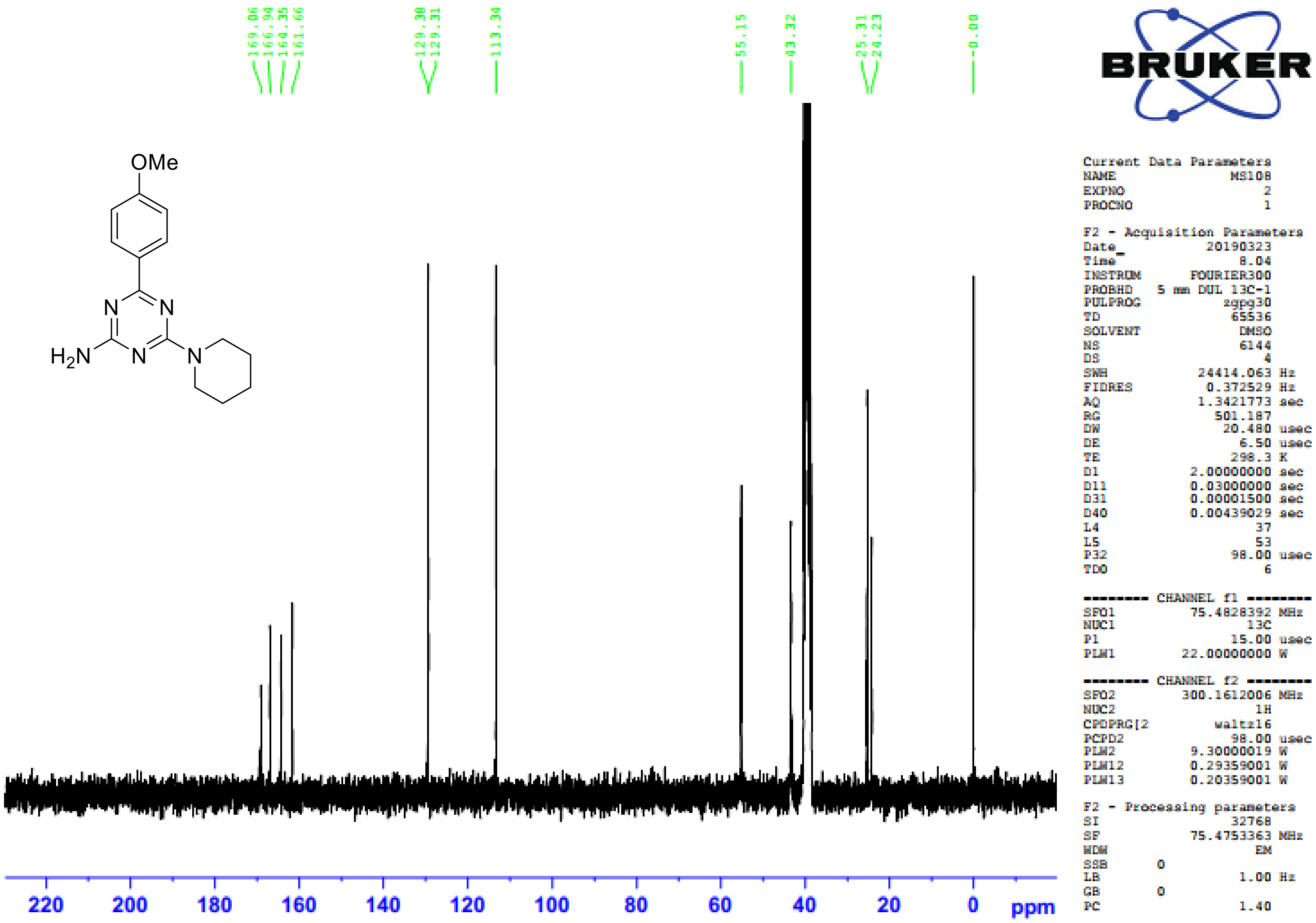

6-(3-Nitrophenyl)-4-piperidino-1,3,5-triazine-2-amine (**9**{*2*,*10*})

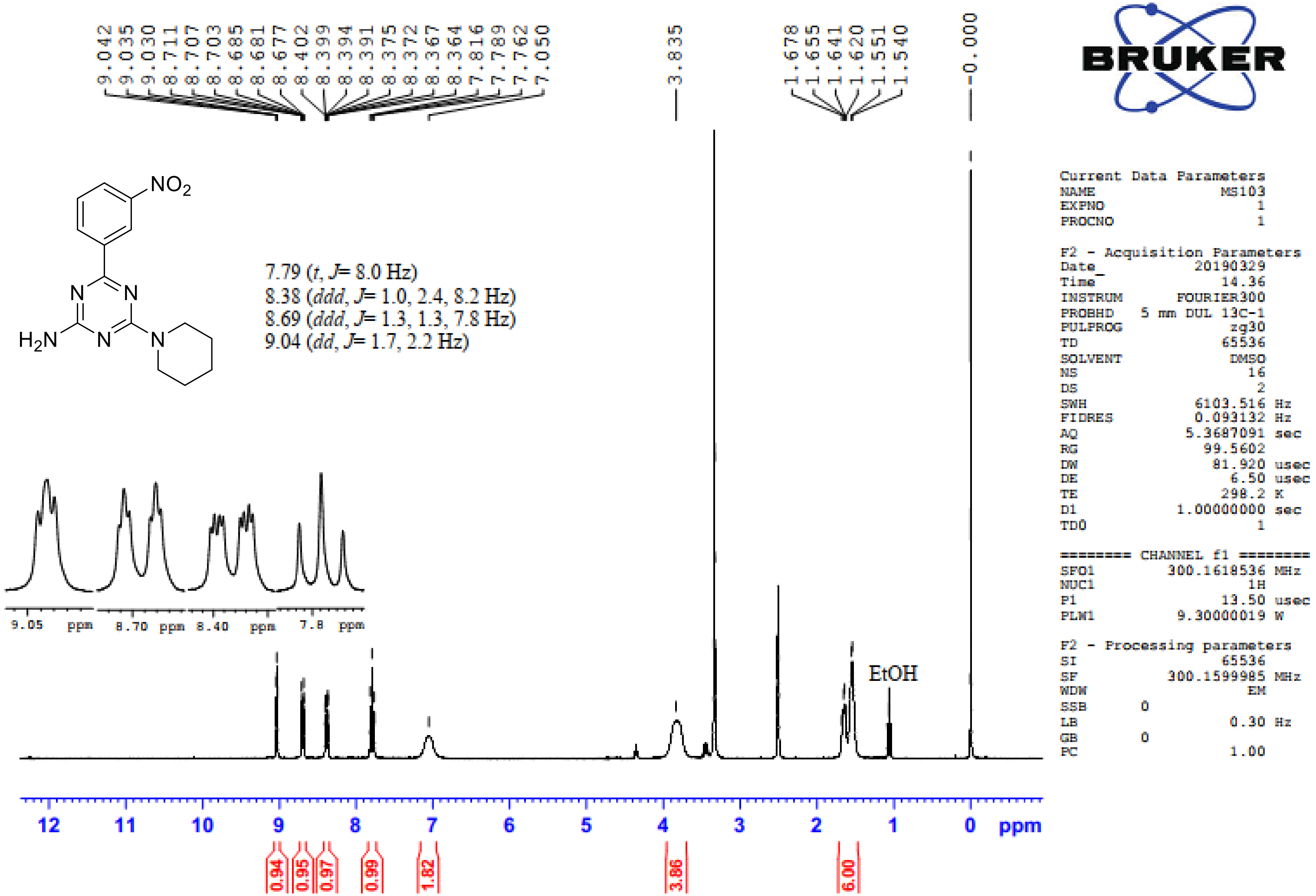

6-(3-Nitrophenyl)-4-piperidino-1,3,5-triazine-2-amine (**9**{*2*,*10*})

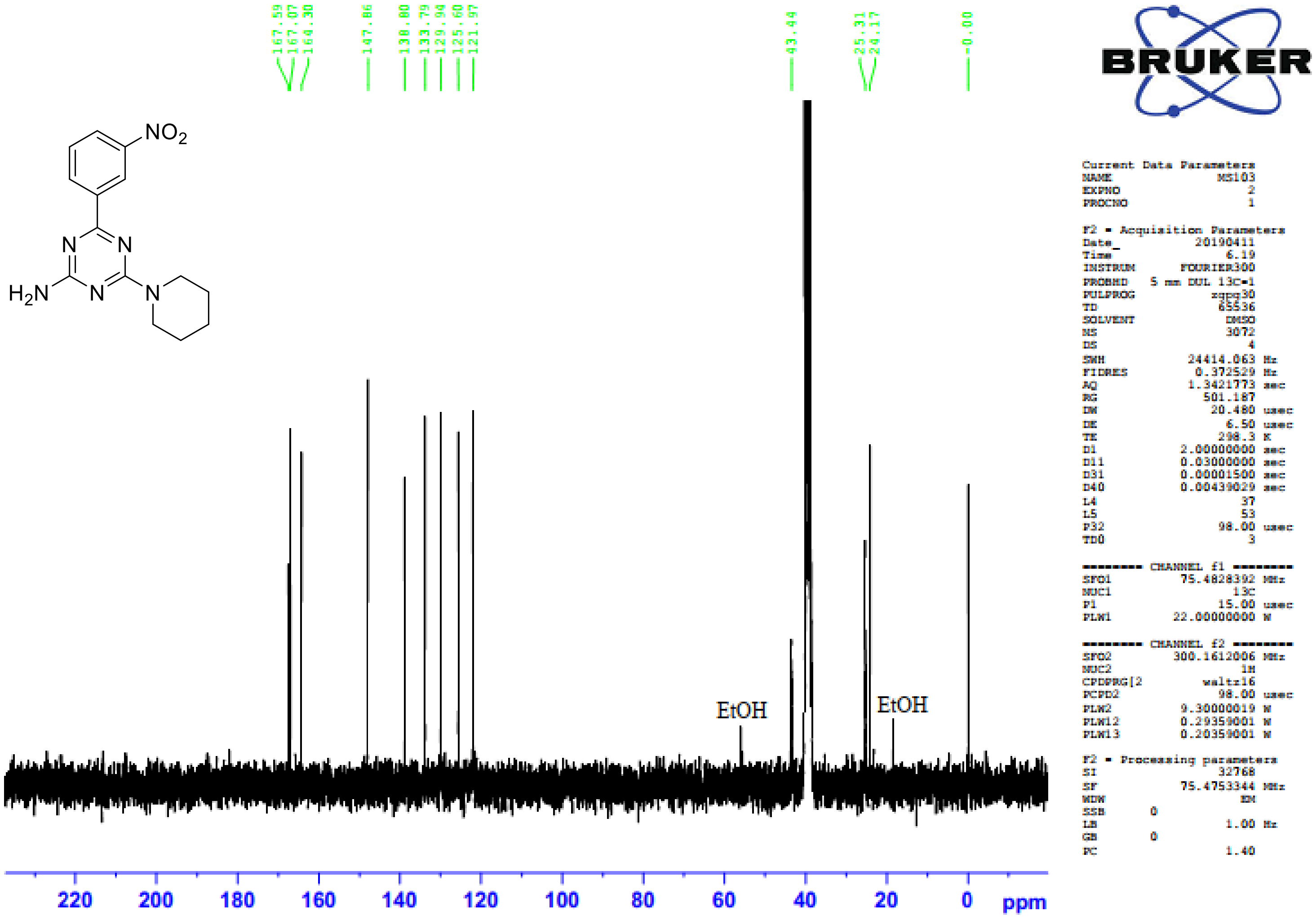

6-[4-(Trifluoromethoxy)phenyl]-4-piperidino-1,3,5-triazine-2-amine (**9**{*2*,*12*})

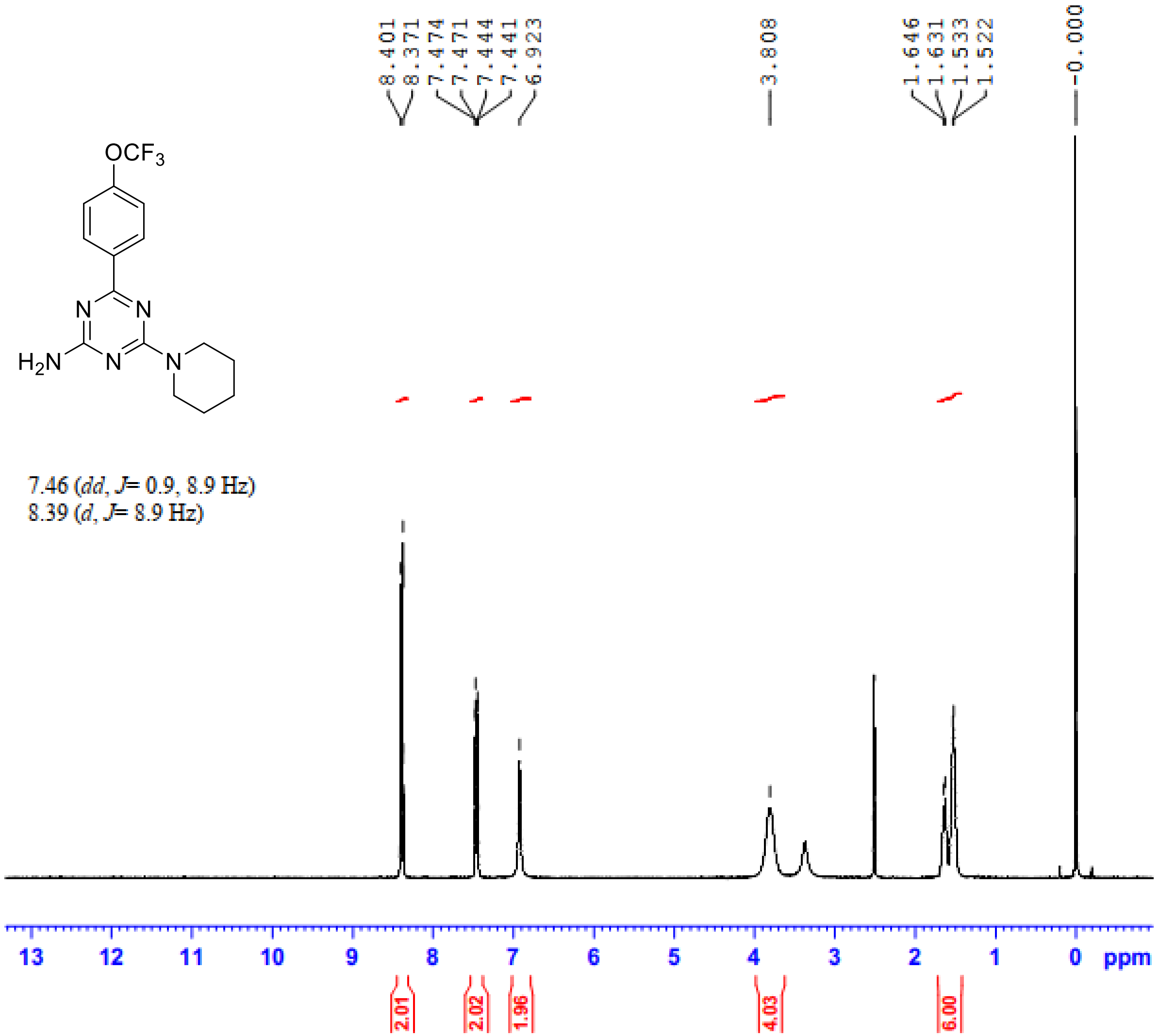


```
Current Data Parameters
NAME                MS102
EXPNO                   1
PROCNO                  1

F2 - Acquisition Parameters
Date_            20190320
Time                15.07
INSTRUM        FOURIER300
PROBHD   5 mm DUL 13C-1
PULPROG              zg30
TD                  65536
SOLVENT              DMSO
NS                     16
DS                      2
SWH              6103.516 Hz
FIDRES           0.093132 Hz
AQ              5.3687091 sec
RG                79.5951
DW                 81.920 usec
DE                   6.50 usec
TE                  298.2 K
D1            1.00000000 sec
TD0                     1

======== CHANNEL f1 ========
SFO1          300.1618536 MHz
NUC1                   1H
P1                  13.50 usec
PLW1           9.30000019 W

F2 - Processing parameters
SI                  65536
SF            300.1599981 MHz
WDW                    EM
SSB      0
LB                   0.30 Hz
GB       0
PC                   1.00
```

6-[4-(Trifluoromethoxy)phenyl]-4-piperidino-1,3,5-triazine-2-amine (**9**{*2,12*})

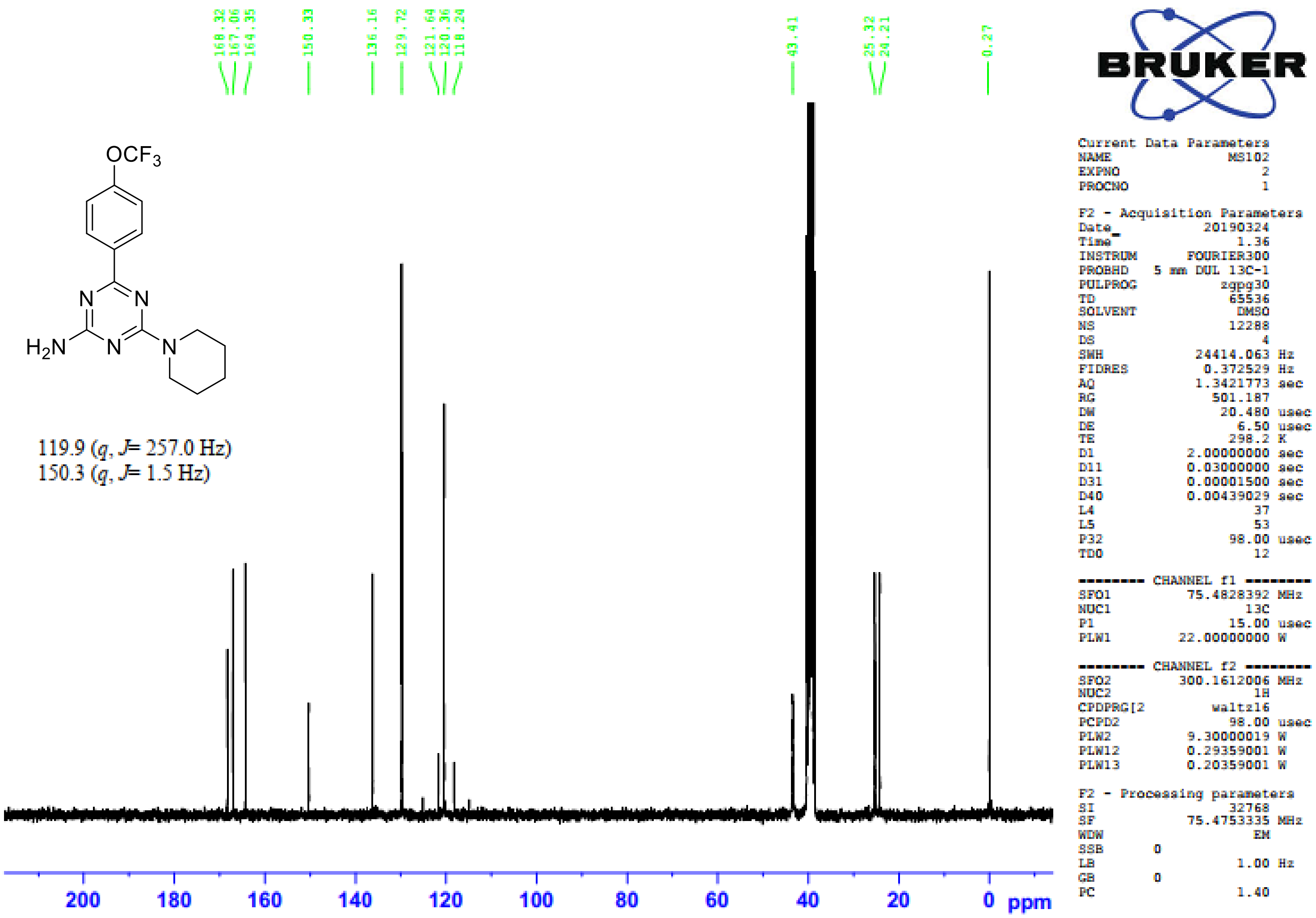

119.9 (*q*, *J*= 257.0 Hz)
150.3 (*q*, *J*= 1.5 Hz)

4-Piperidino-6-(3-phenoxyphenyl)-1,3,5-triazine-2-amine (**9**{*2*,*13*})

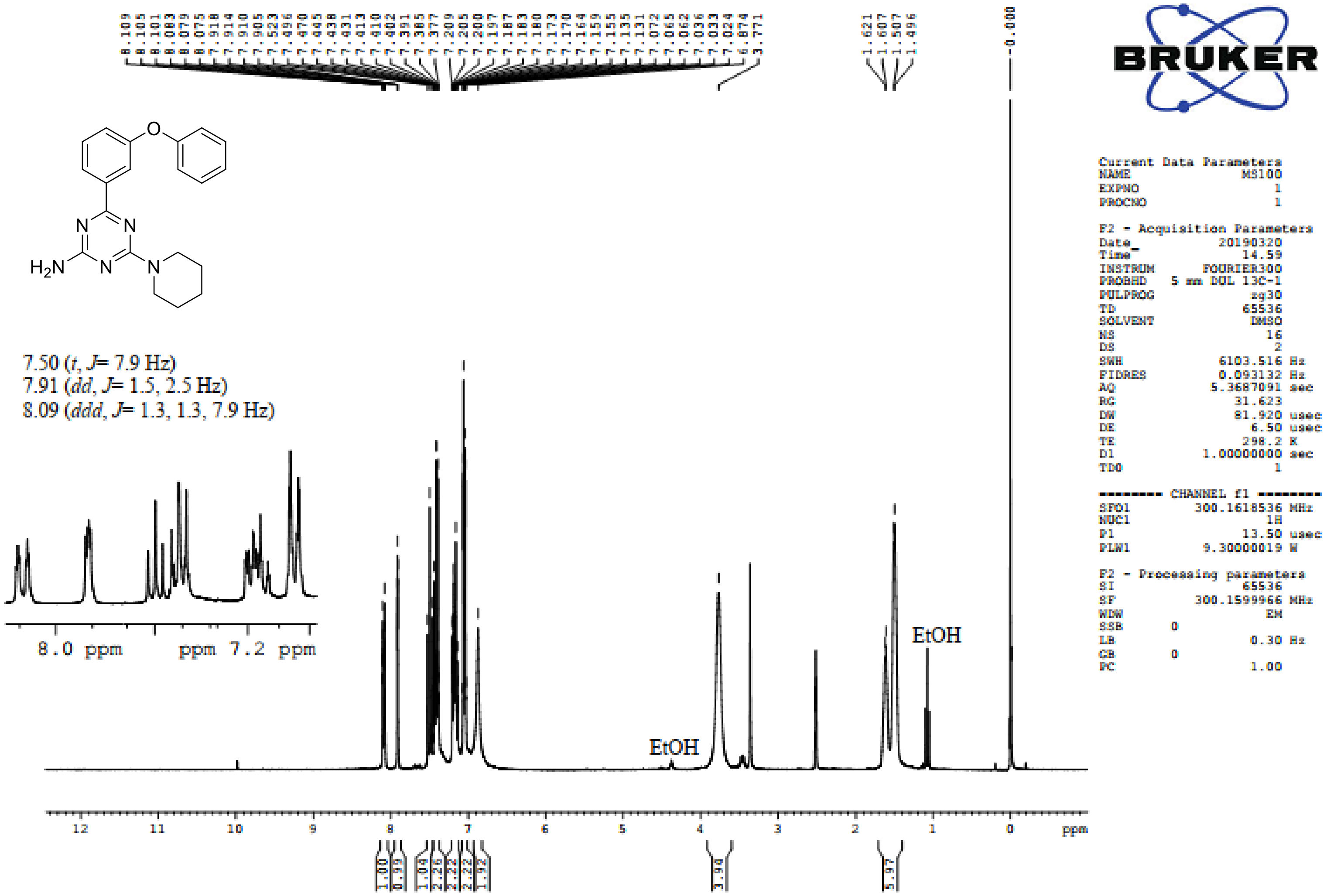

4-Piperidino-6-(3-phenoxyphenyl)-1,3,5-triazine-2-amine (**9**{*2*,*13*})

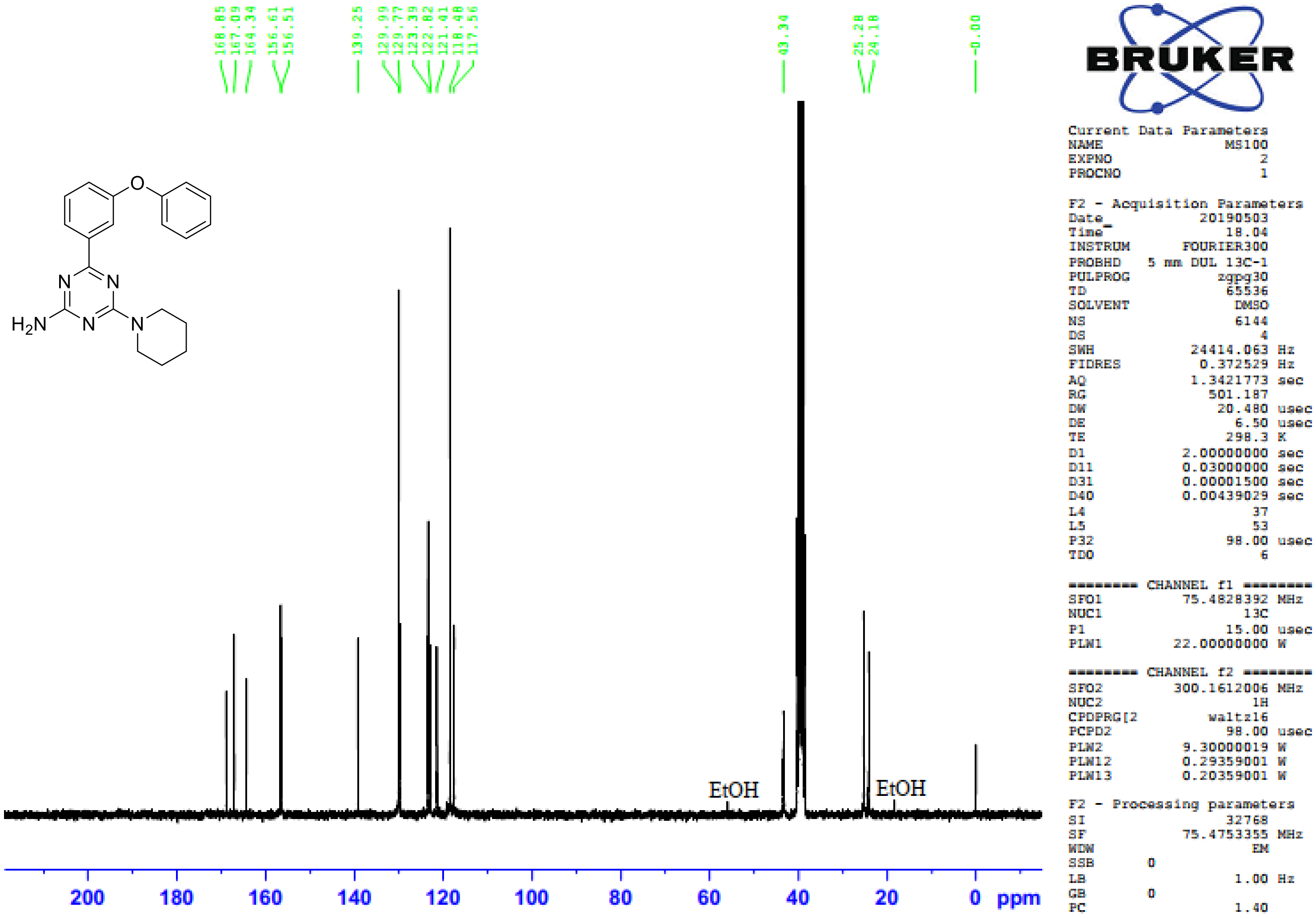

6-[3-(Benzyloxy)phenyl]-4-piperidino-1,3,5-triazine-2-amine (**9**{*2*,*14*})

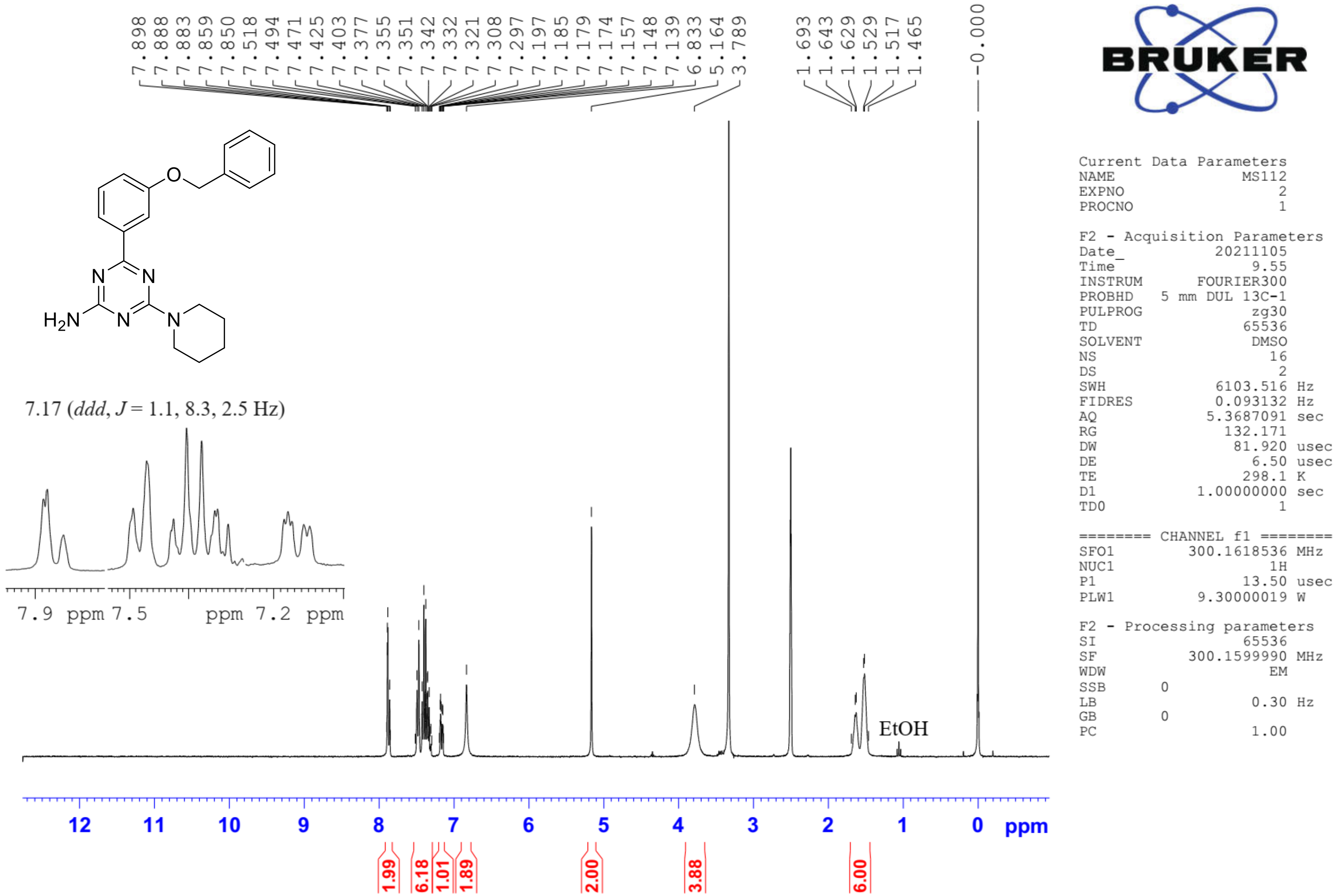

6-[3-(Benzyloxy)phenyl]-4-piperidino-1,3,5-triazine-2-amine (**9**{*2*,*14*})

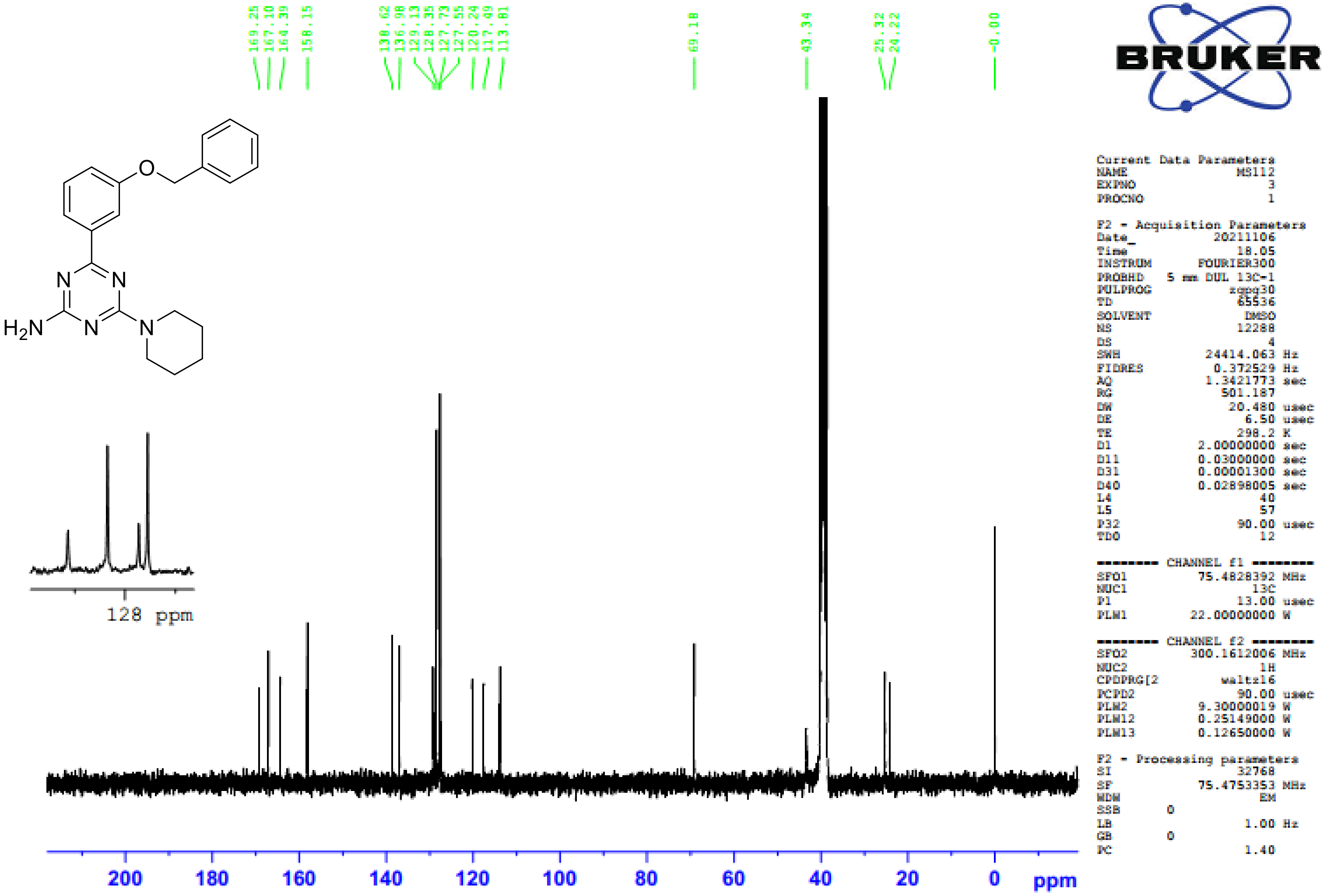

6-(3-Fluorophenyl)-4-(4-methylpiperidino)-1,3,5-triazine-2-amine (**9**{*3*,*2*})

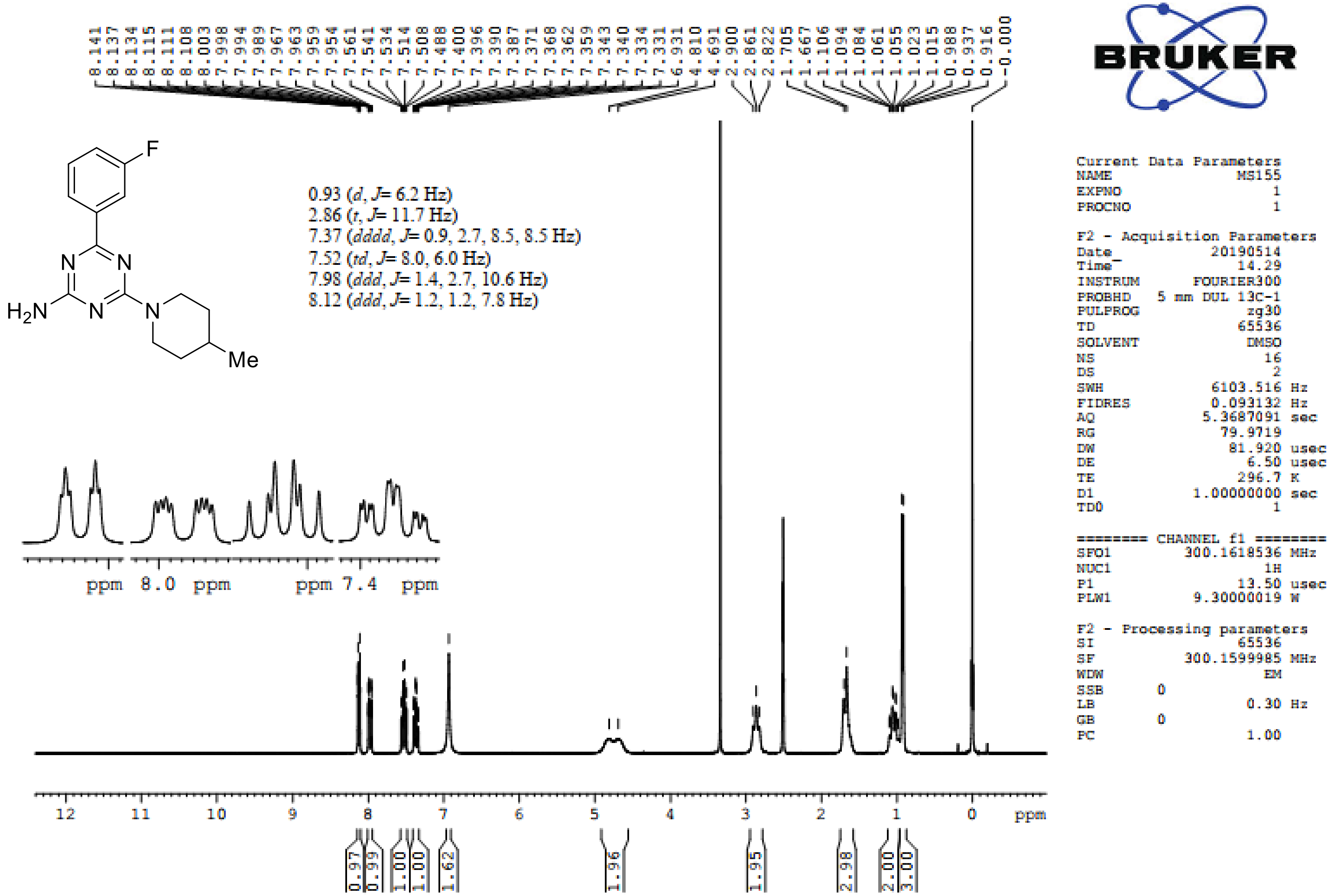

6-(3-Fluorophenyl)-4-(4-methylpiperidino)-1,3,5-triazine-2-amine (**9**{*3,2*})

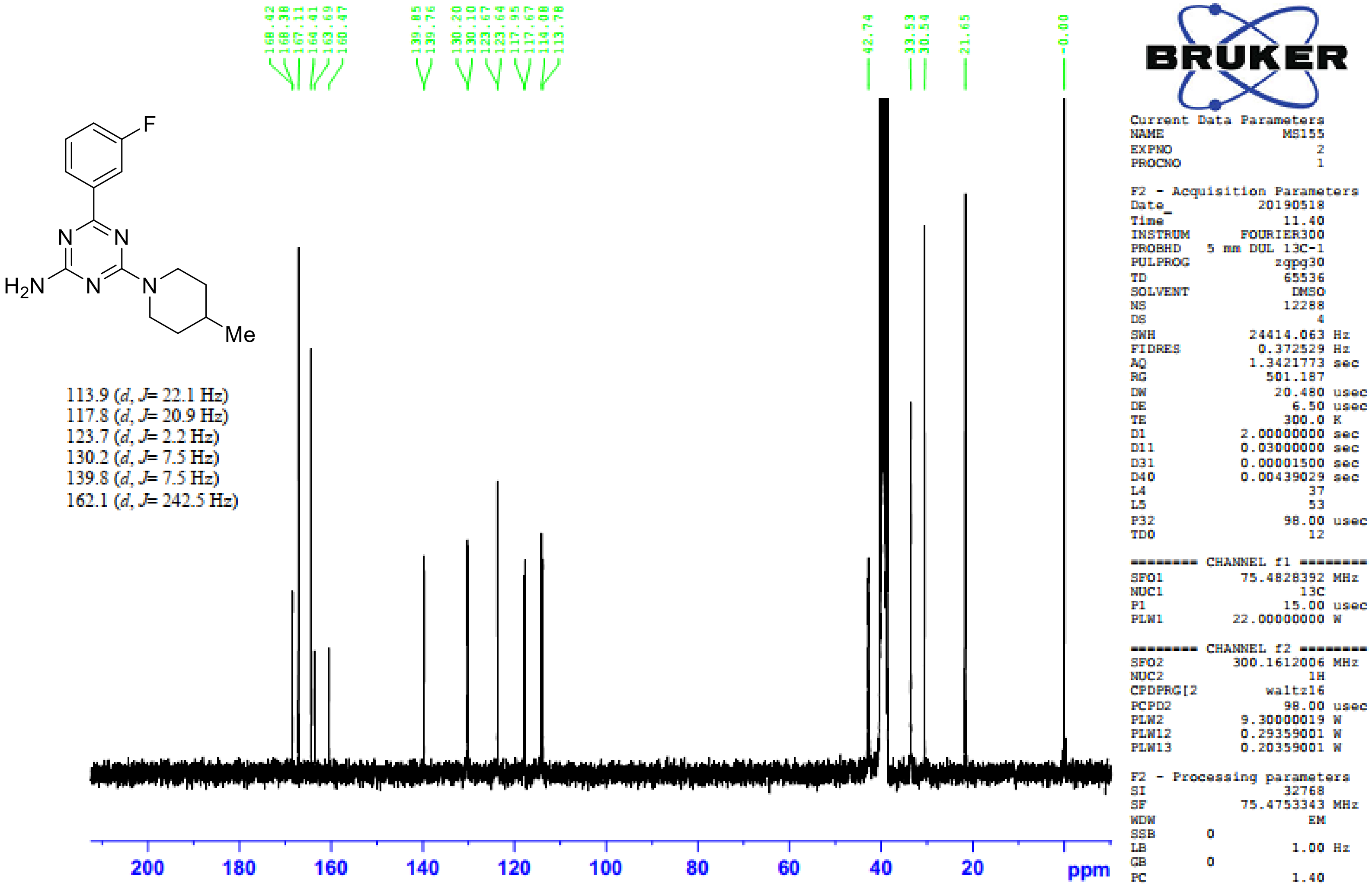

113.9 (*d*, *J*= 22.1 Hz)
117.8 (*d*, *J*= 20.9 Hz)
123.7 (*d*, *J*= 2.2 Hz)
130.2 (*d*, *J*= 7.5 Hz)
139.8 (*d*, *J*= 7.5 Hz)
162.1 (*d*, *J*= 242.5 Hz)

6-(4-Fluorophenyl)-4-(4-methylpiperidino)-1,3,5-triazine-2-amine (**9**{*3*,*3*})

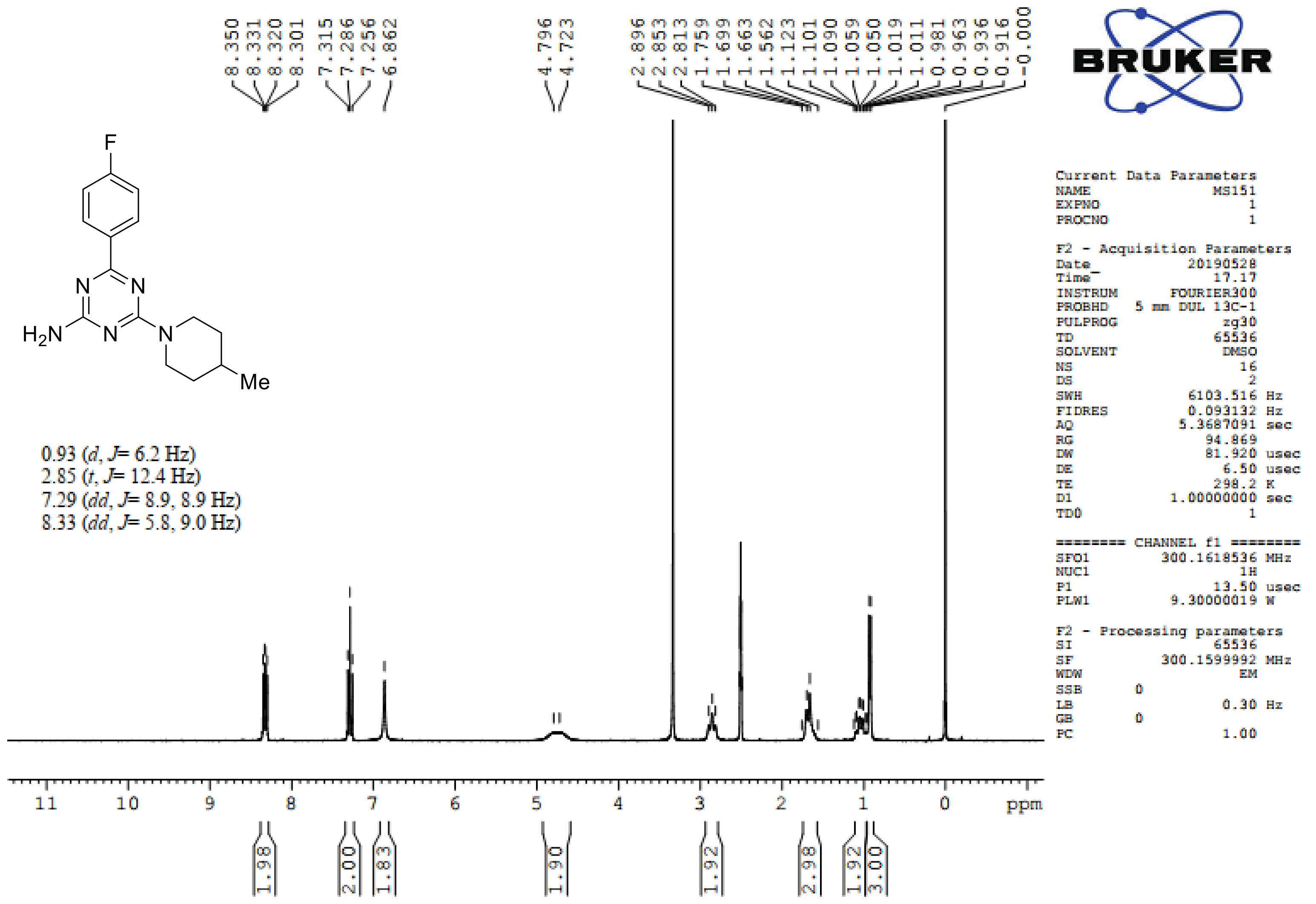

6-(4-Fluorophenyl)-4-(4-methylpiperidino)-1,3,5-triazine-2-amine (**9**{*3*,*3*})

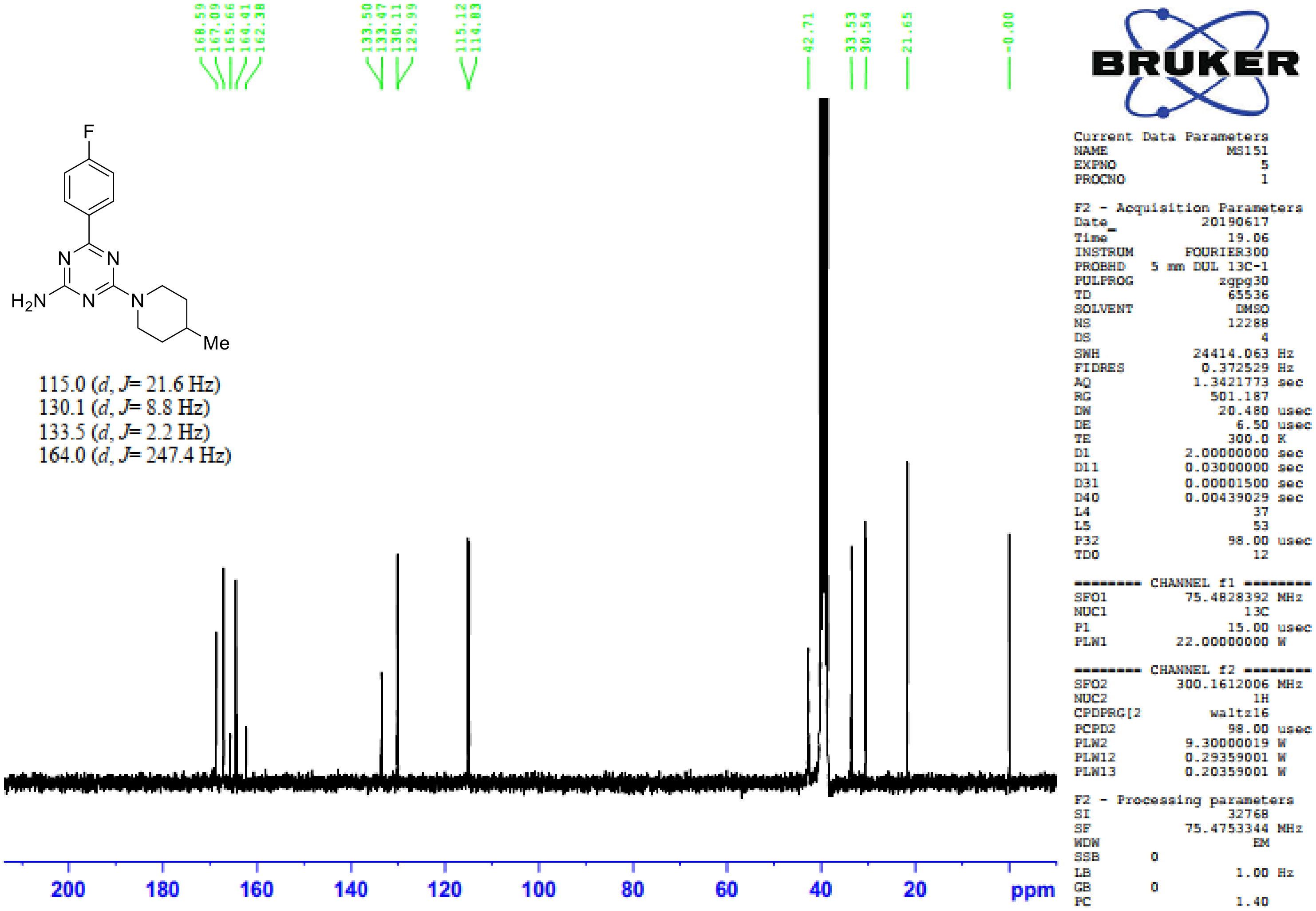

6-(4-Chlorophenyl)-4-(4-methylpiperidino)-1,3,5-triazine-2-amine (**9**{*3*,*4*})

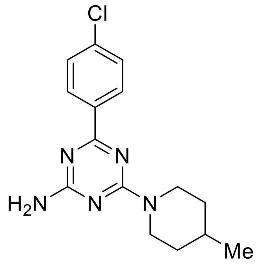

0.92 (*d*, *J*= 6.2 Hz)
2.86 (*t*, *J*= 12.3 Hz)
7.53 (*d*, *J*= 8.8 Hz)
8.28 (*d*, *J*= 8.8 Hz)

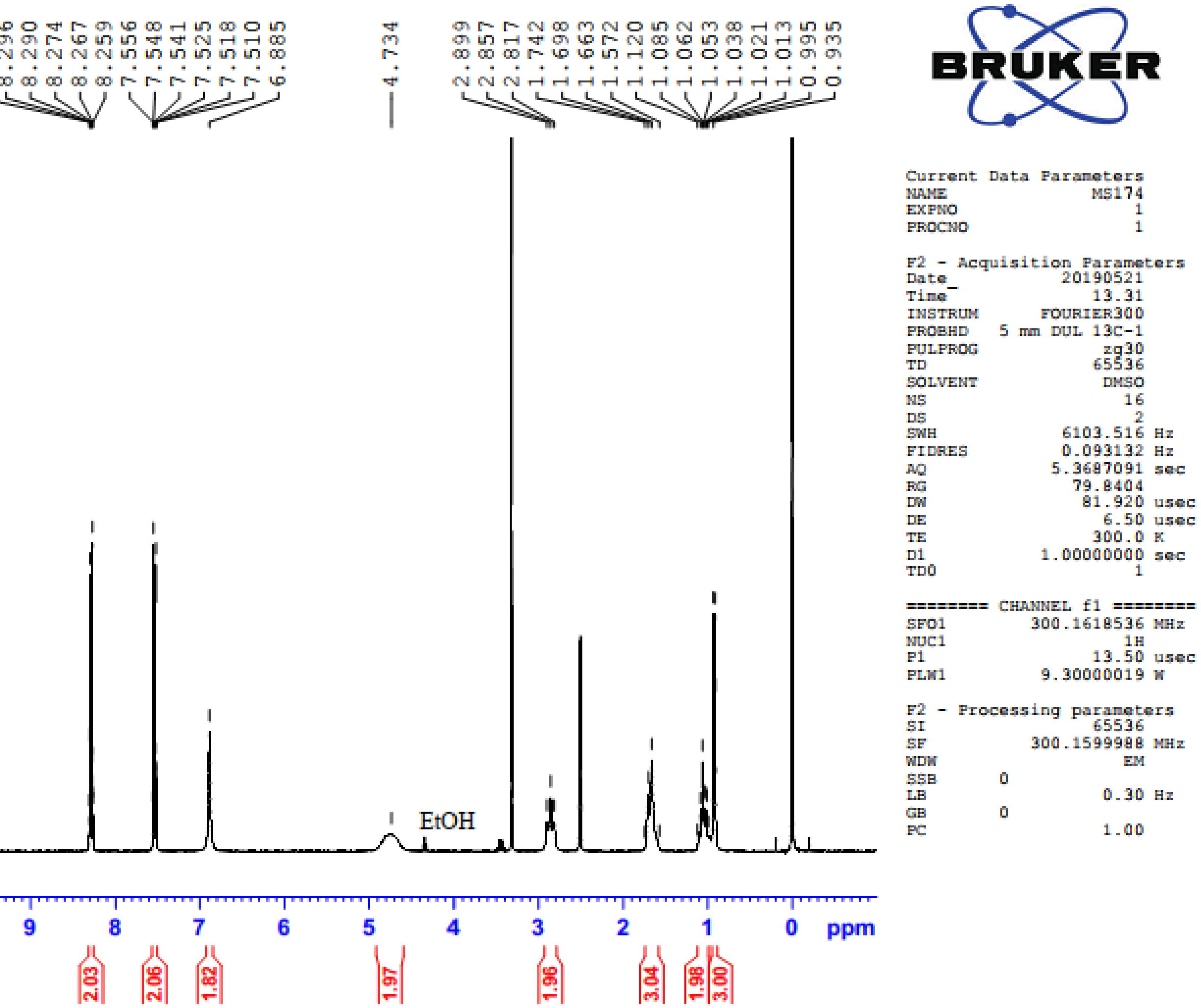

6-(4-Chlorophenyl)-4-(4-methylpiperidino)-1,3,5-triazine-2-amine (**9**{*3,4*})

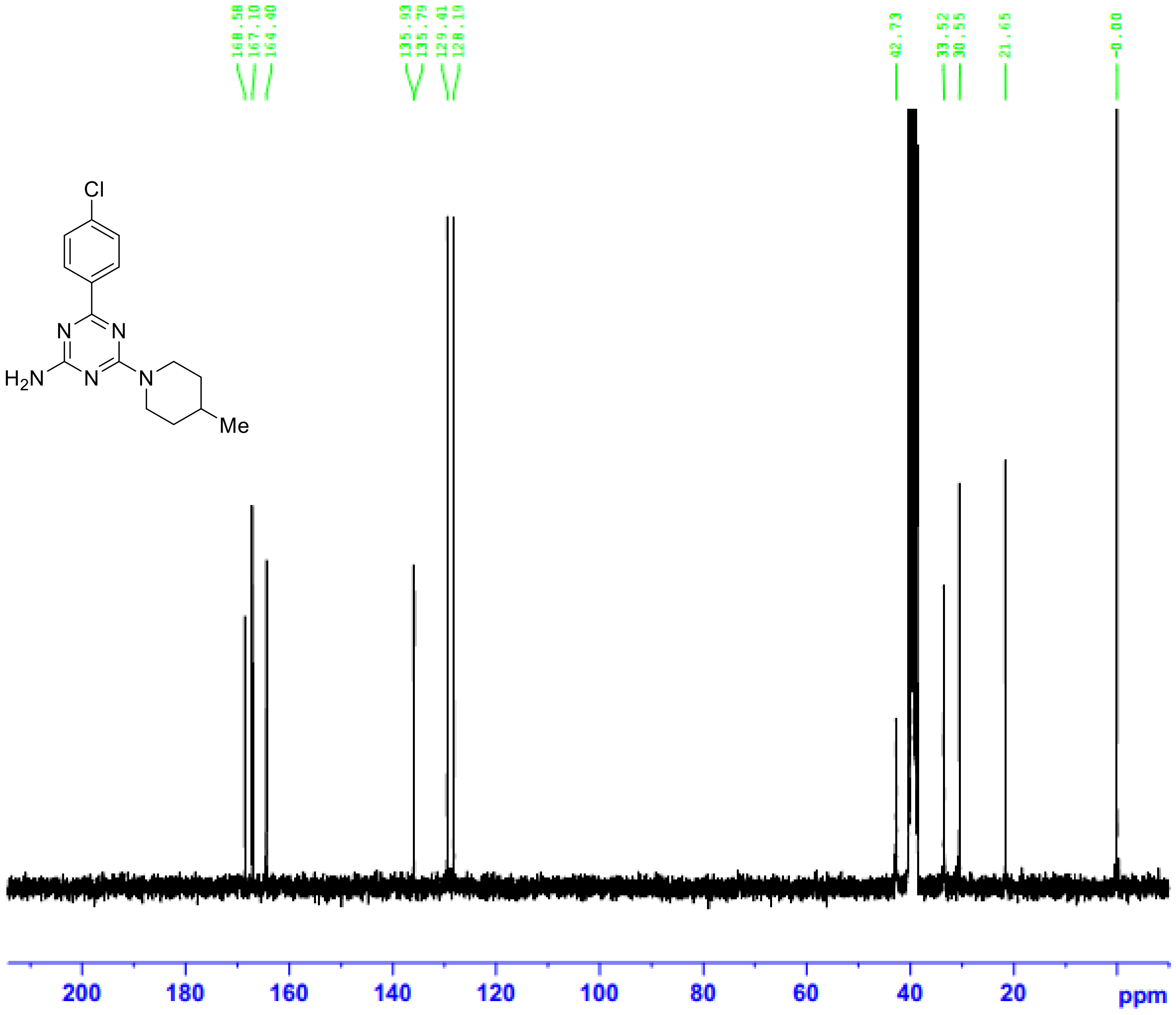

BRUKER

Current Data Parameters
NAME MS174
EXPNO 2
PROCNO 1

F2 - Acquisition Parameters
Date_ 20190525
Time 17.22
INSTRUM FOURIER300
PROBHD 5 mm DUL 13C-1
PULPROG zgpg30
TD 65536
SOLVENT DMSO
NS 6144
DS 4
SWH 24414.063 Hz
FIDRES 0.372529 Hz
AQ 1.3421773 sec
RG 501.187
DW 20.480 usec
DE 6.50 usec
TE 300.1 K
D1 2.00000000 sec
D11 0.03000000 sec
D31 0.00001500 sec
D40 0.00439029 sec
L4 37
L5 53
P32 98.00 usec
TD0 6

======== CHANNEL f1 ========
SFO1 75.4828392 MHz
NUC1 13C
P1 15.00 usec
PLW1 22.00000000 W

======== CHANNEL f2 ========
SFO2 300.1612006 MHz
NUC2 1H
CPDPRG[2 waltz16
PCPD2 98.00 usec
PLW2 9.30000019 W
PLW12 0.29359001 W
PLW13 0.20359001 W

F2 - Processing parameters
SI 32768
SF 75.4753343 MHz
WDW EM
SSB 0
LB 1.00 Hz
GB 0
PC 1.40

6-(4-Methylphenyl)-4-(4-methylpiperidino)-1,3,5-triazine-2-amine (**9**{*3*,*6*})

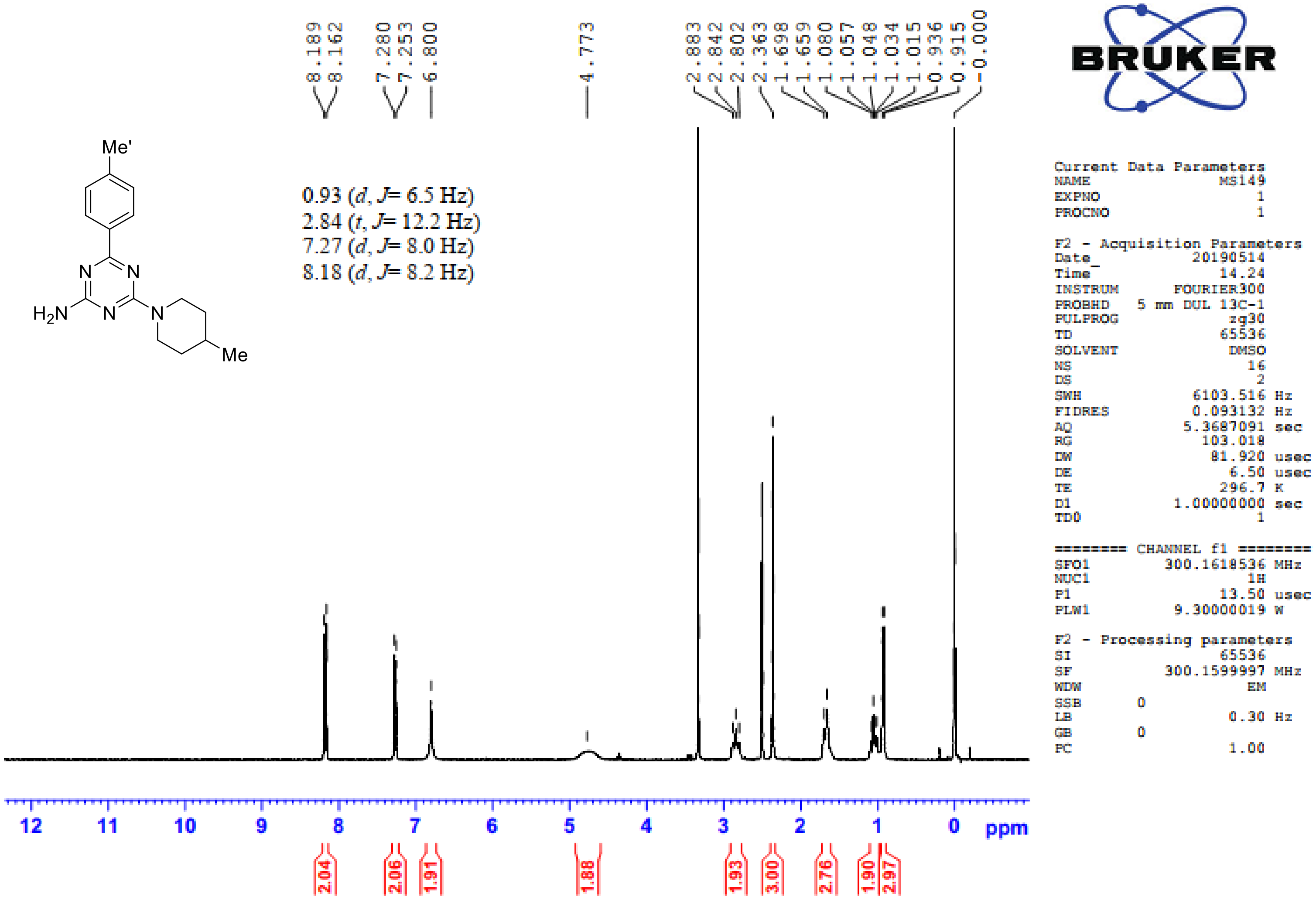

6-(4-Methylphenyl)-4-(4-methylpiperidino)-1,3,5-triazine-2-amine (**9**{*3*,*6*})

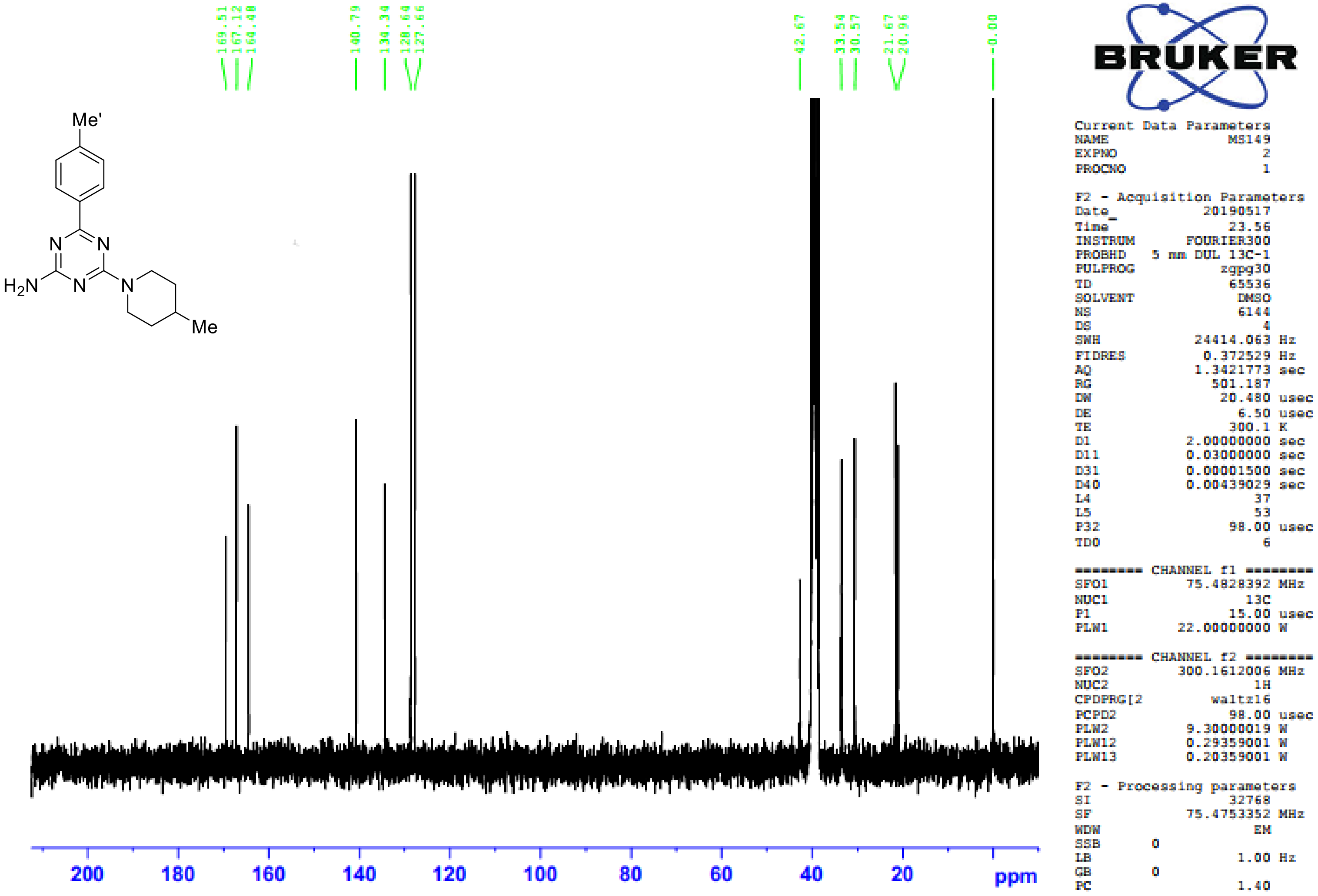

6-[4-(*N*,*N*-Dimethylamino)phenyl]-4-(4-methylpiperidino)-1,3,5-triazine-2-amine (**9**{*3*,*8*})

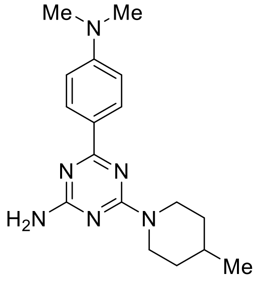

0.92 (*d*, *J*= 6.2 Hz)
2.82 (*t*, *J*= 12.1 Hz)
6.72 (*d*, *J*= 9.1 Hz)
8.13 (*d*, *J*= 9.0 Hz)

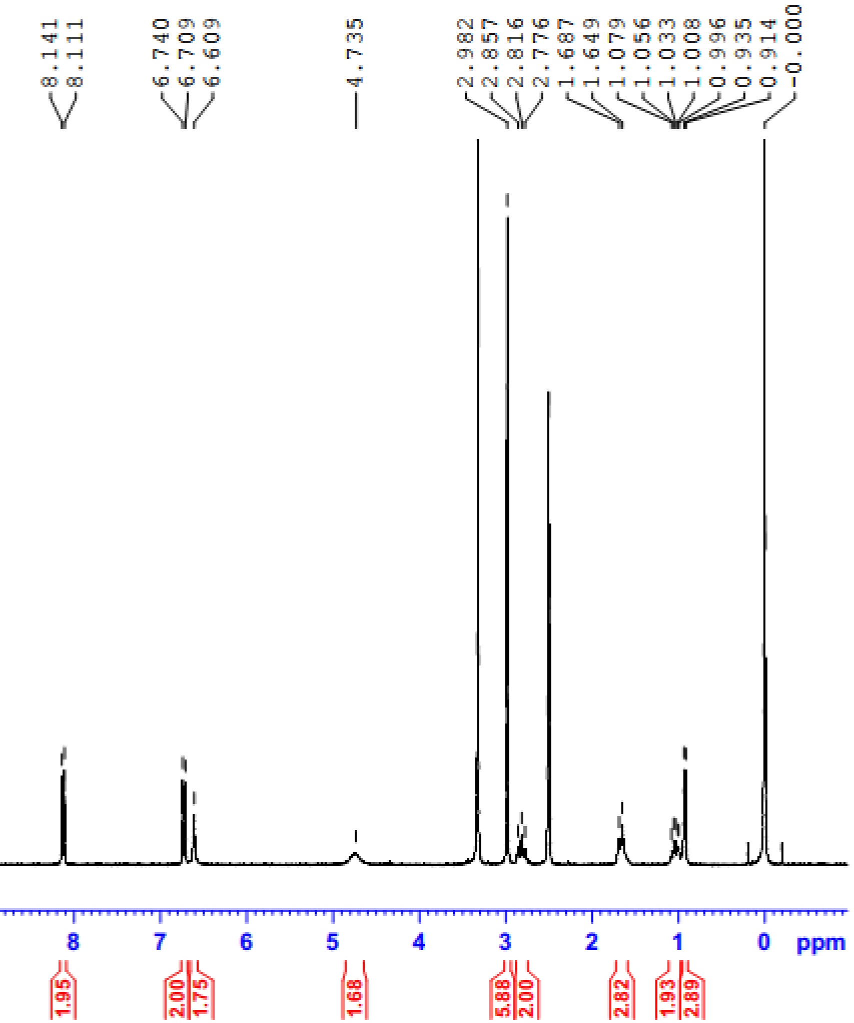

Current Data Parameters
NAME MS248
EXPNO 1
PROCNO 1

F2 - Acquisition Parameters
Date_ 20200113
Time 14.26
INSTRUM FOURIER300
PROBHD 5 mm DUL 13C-1
PULPROG zg30
TD 65536
SOLVENT DMSO
NS 16
DS 2
SWH 6103.516 Hz
FIDRES 0.093132 Hz
AQ 5.3687091 sec
RG 92.4318
DW 81.920 usec
DE 6.50 usec
TE 298.4 K
D1 1.00000000 sec
TD0 1

======== CHANNEL f1 ========
SFO1 300.1618536 MHz
NUC1 1H
P1 13.50 usec
PLW1 9.30000019 W

F2 - Processing parameters
SI 65536
SF 300.1599998 MHz
WDW EM
SSB 0
LB 0.30 Hz
GB 0
PC 1.00

6-[4-(*N*,*N*-Dimethylamino)phenyl]-4-(4-methylpiperidino)-1,3,5-triazine-2-amine (**9**{*3,8*})

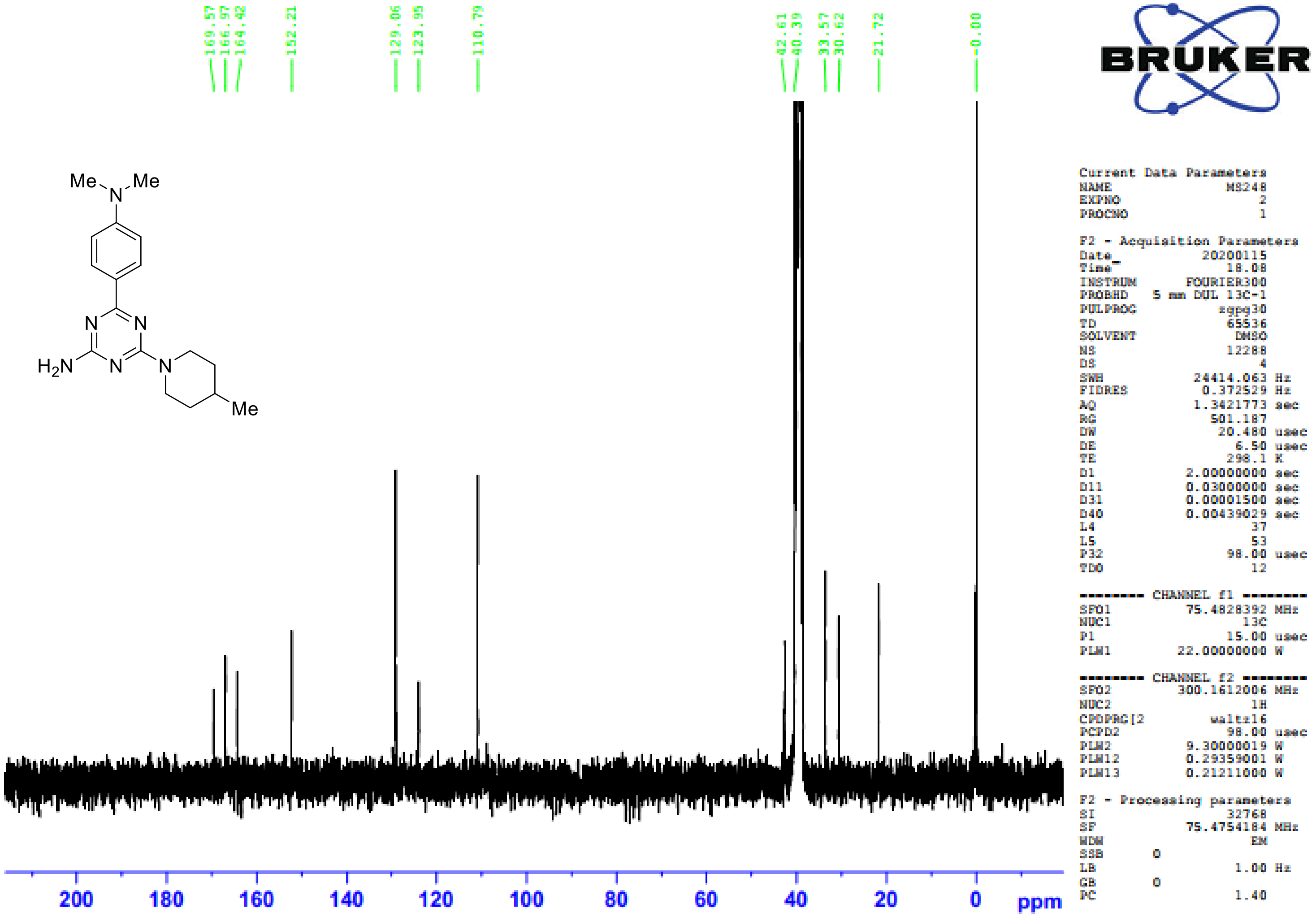

6-(4-Methoxyphenyl)-4-(4-methylpiperidino)-1,3,5-triazine-2-amine (**9**{*3*,*9*})

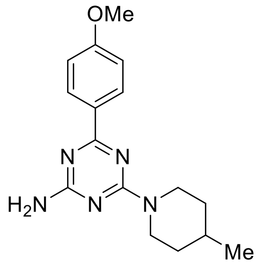

0.92 (*d*, *J*= 6.1 Hz)
2.84 (*t*, *J*= 12.4 Hz)
7.00 (*d*, *J*= 9.0 Hz)
8.24 (*d*, *J*= 8.8 Hz)

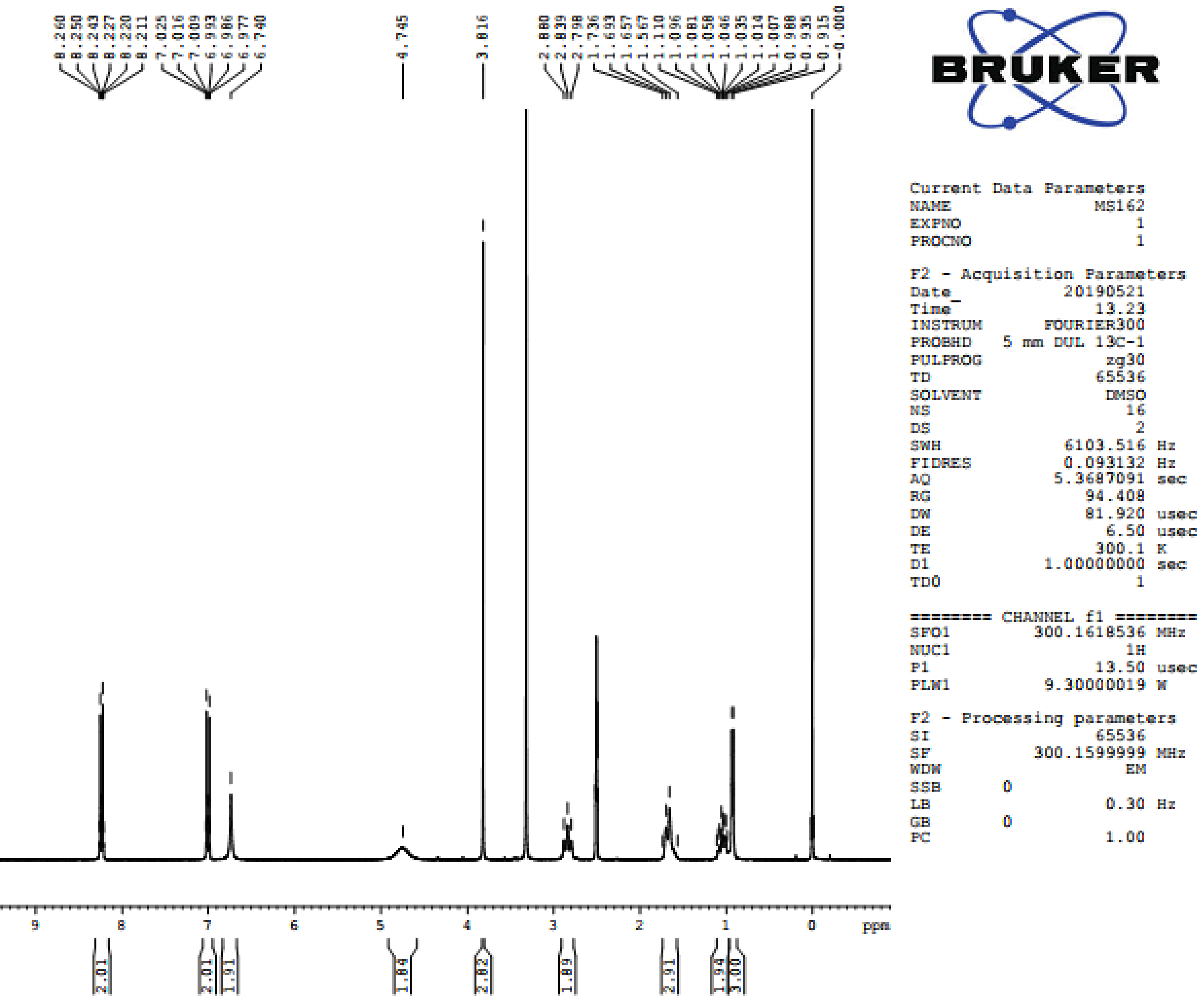

6-(4-Methoxyphenyl)-4-(4-methylpiperidino)-1,3,5-triazine-2-amine (**9**{*3,9*})

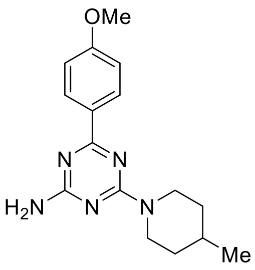

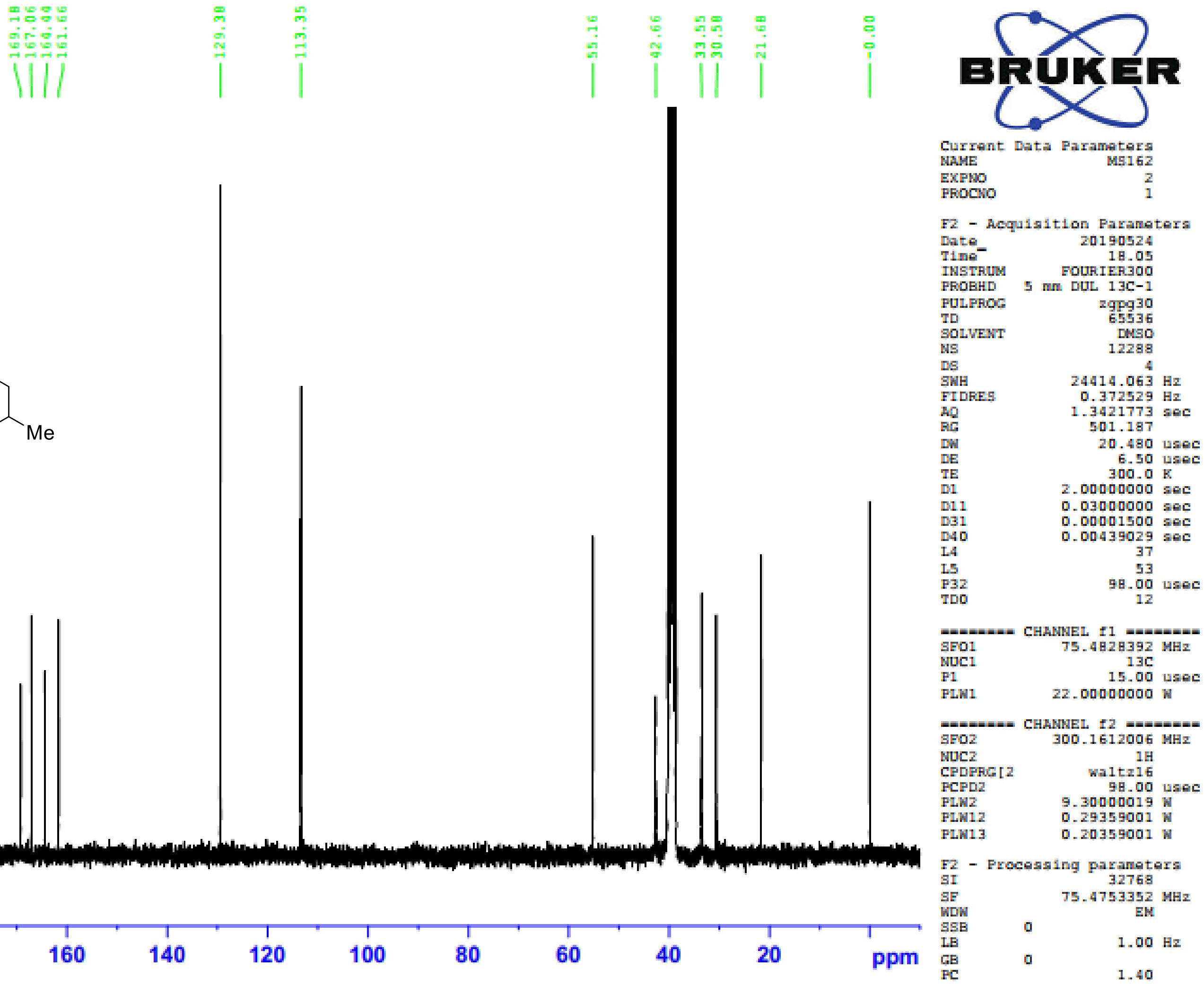

4-(4-Methylpiperidino)-6-(3-nitrophenyl)-1,3,5-triazine-2-amine (**9**{*3*,*10*})

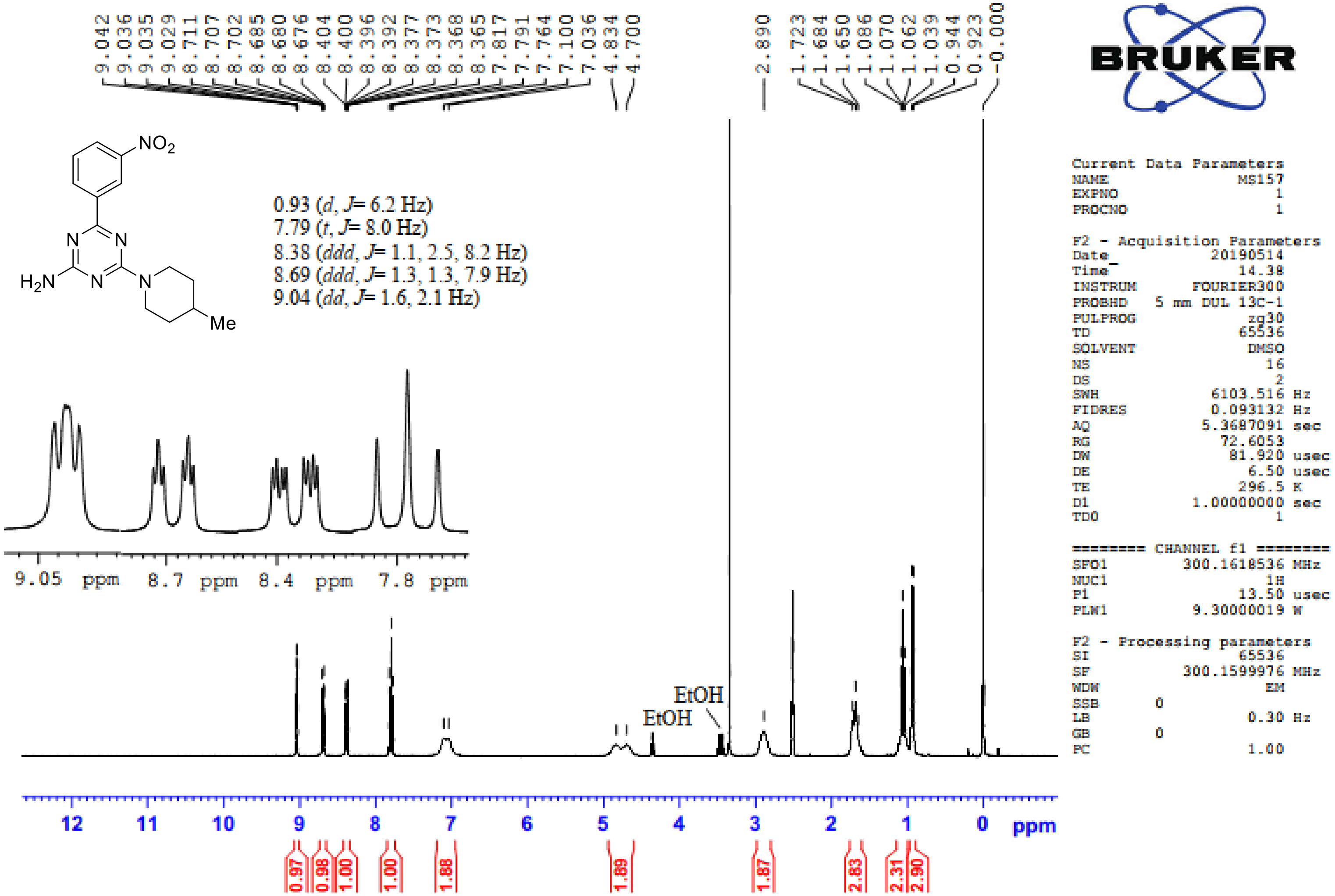

4-(4-Methylpiperidino)-6-(3-nitrophenyl)-1,3,5-triazine-2-amine (**9**{*3*,*10*})

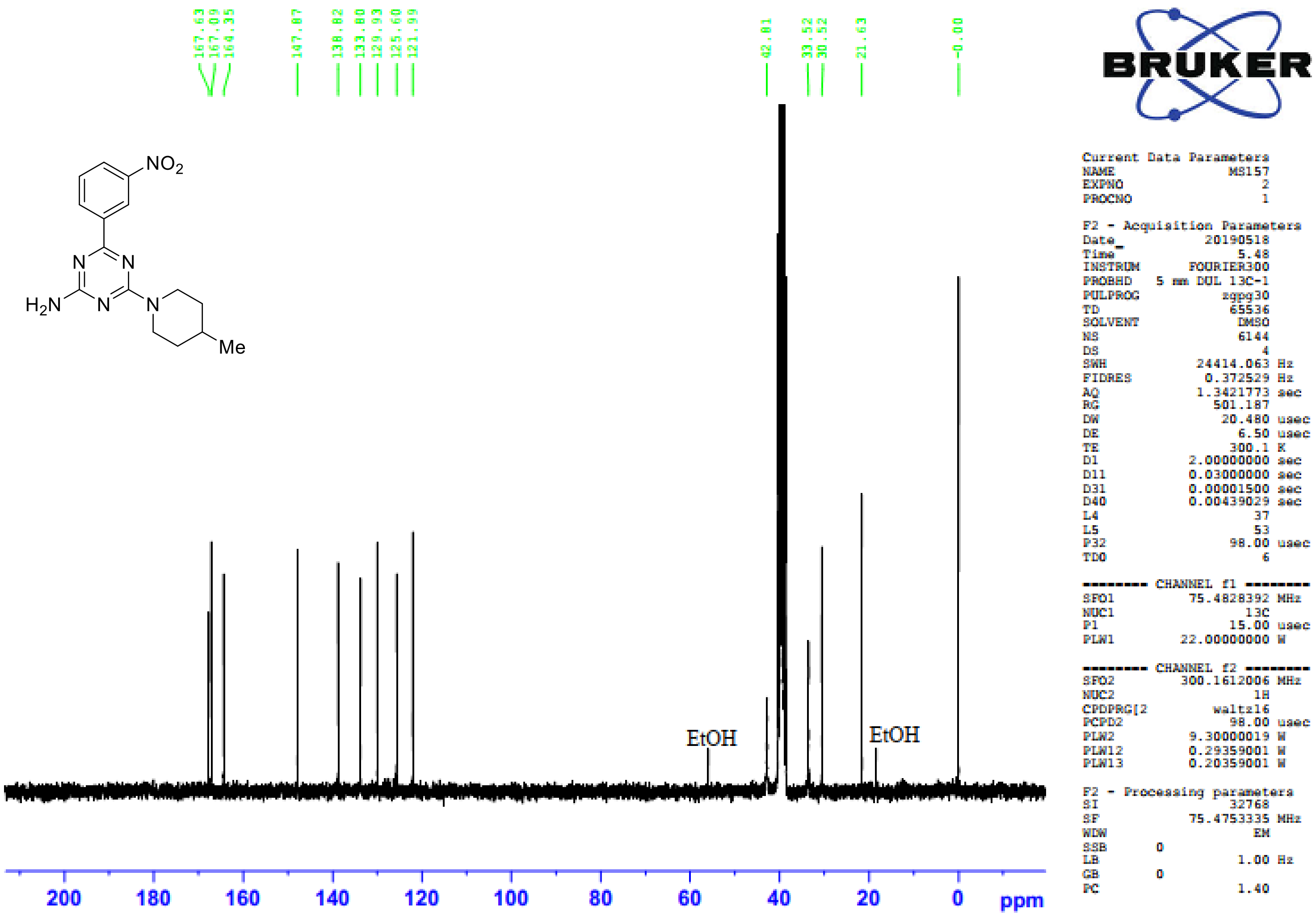

6-[4-(Trifluoromethyl)phenyl]-4-(4-methylpiperidino)-1,3,5-triazine-2-amine (**9**{*3,11*})

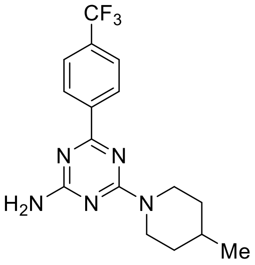

0.93 (*d*, *J*= 6.2 Hz)
2.88 (*t*, *J*= 11.8 Hz)
7.85 (*d*, *J*= 8.6 Hz)
8.46 (*d*, *J*= 7.8 Hz)

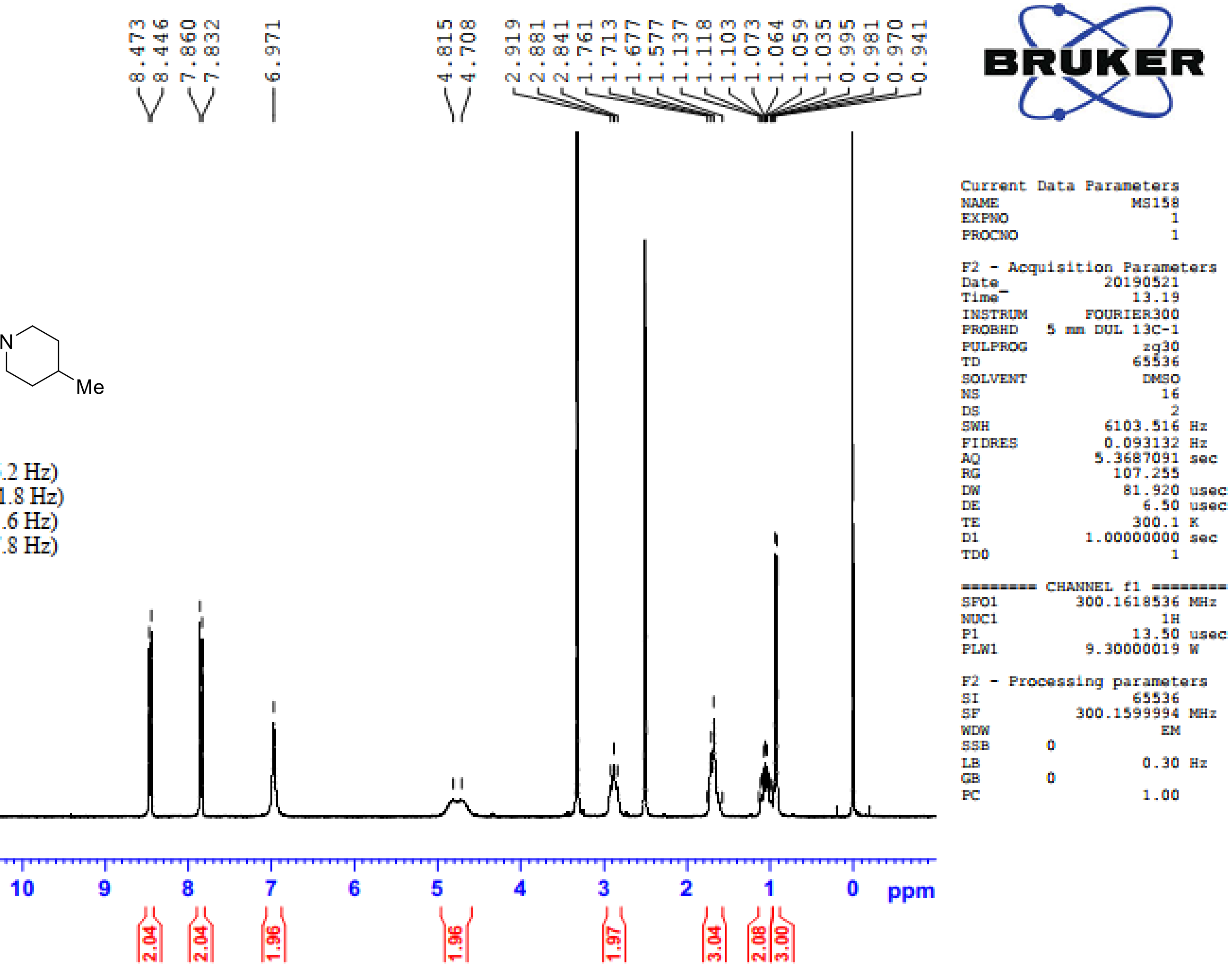

6-[4-(Trifluoromethyl)phenyl]-4-(4-methylpiperidino)-1,3,5-triazine-2-amine (**9**{*3,11*})

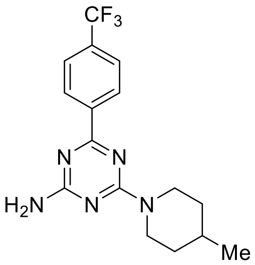

124.1 (*q*, *J*= 272.2 Hz)
125.1 (*q*, *J*= 4.0 Hz)
130.8 (*q*, *J*= 31.8 Hz)
141.0 (*q*, *J*= 1.5 Hz)

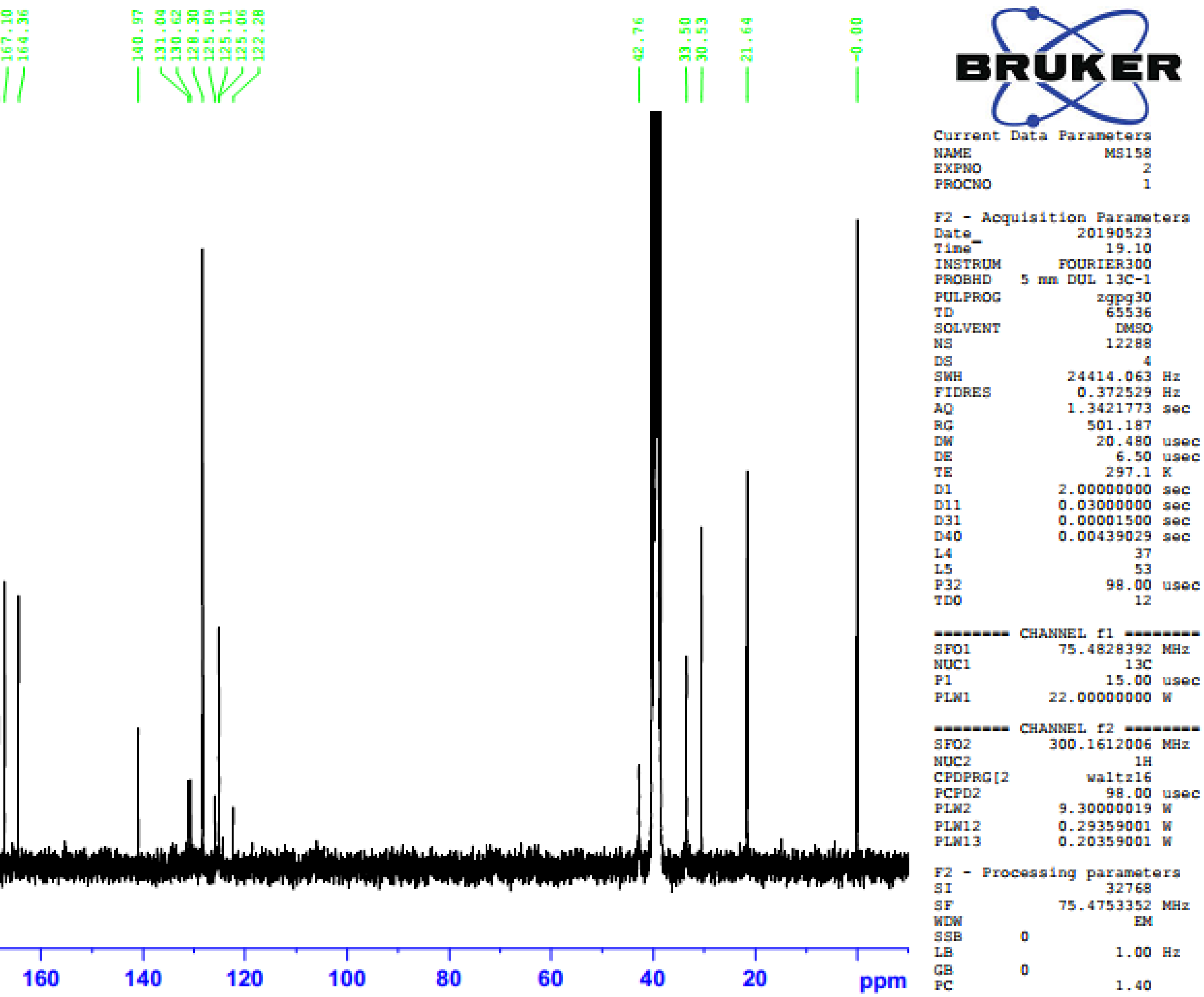

6-[4-(Trifluoromethoxy)phenyl]-4-(4-methylpiperidino)-1,3,5-triazine-2-amine (**9**{*3,12*})

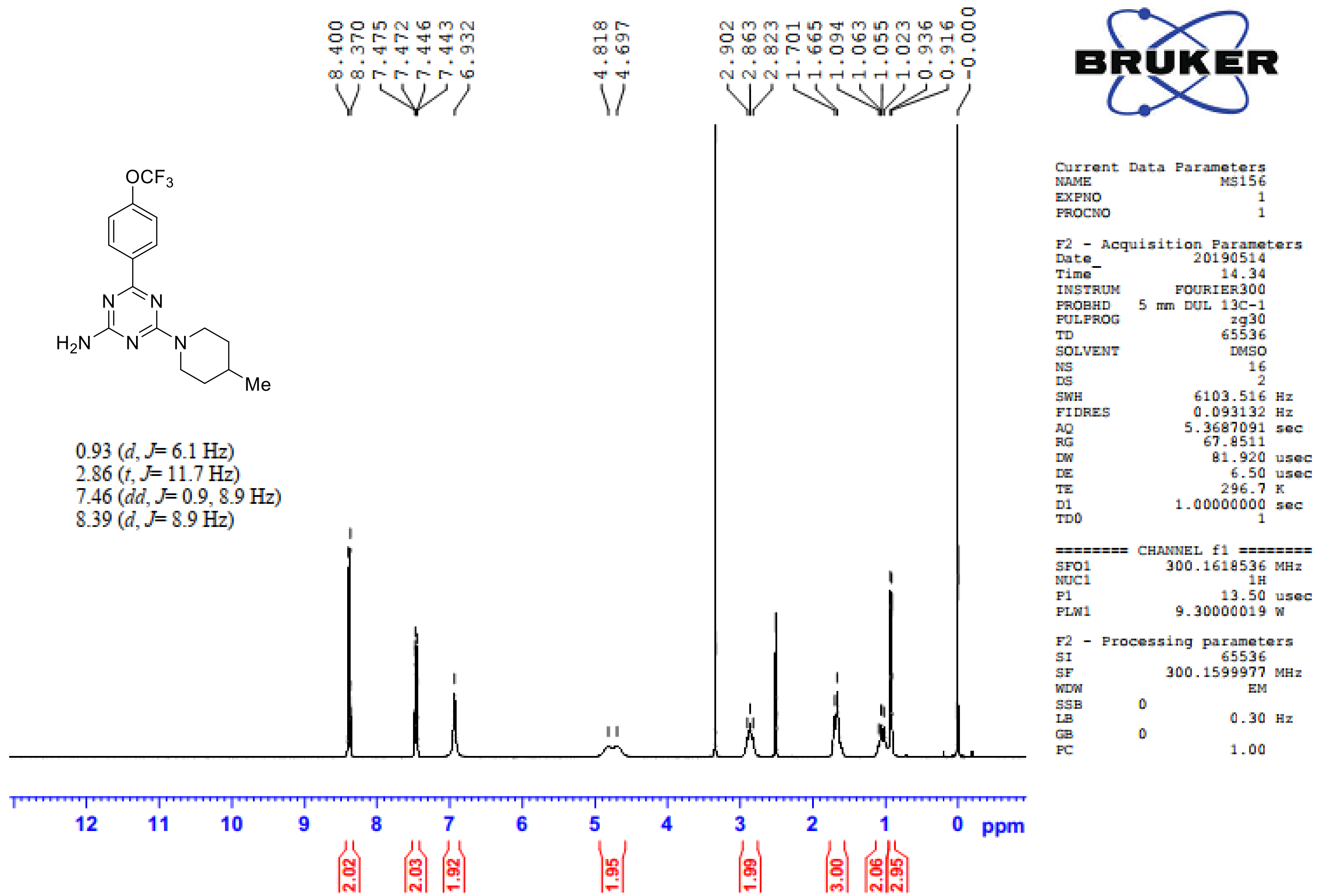

6-[4-(Trifluoromethoxy)phenyl]-4-(4-methylpiperidino)-1,3,5-triazine-2-amine (**9**{*3*,*12*})

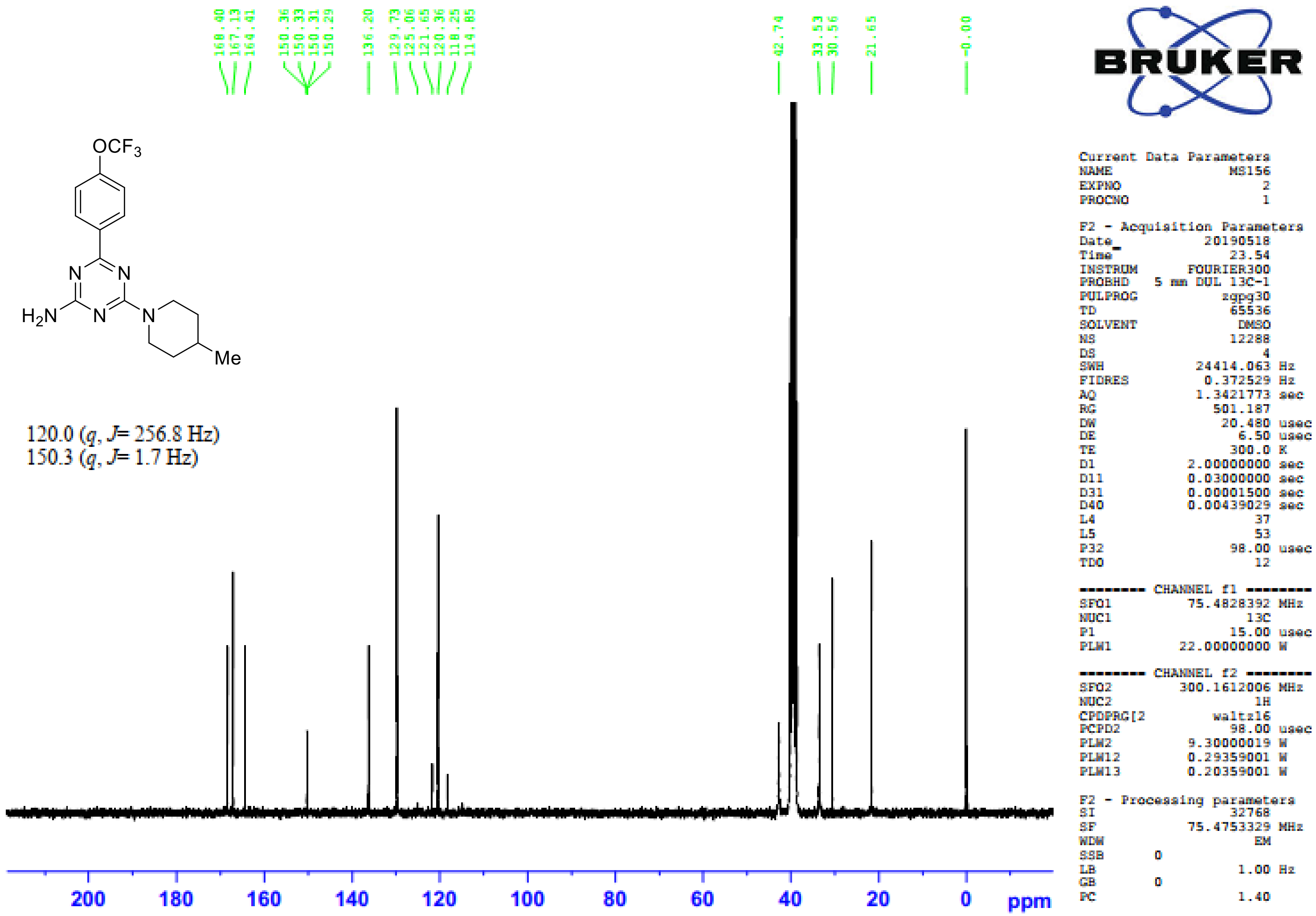

6-(3,4,5-Trimethoxyphenyl)-4-(4-methylpiperidino)-1,3,5-triazine-2-amine (**9**{*3,17*})

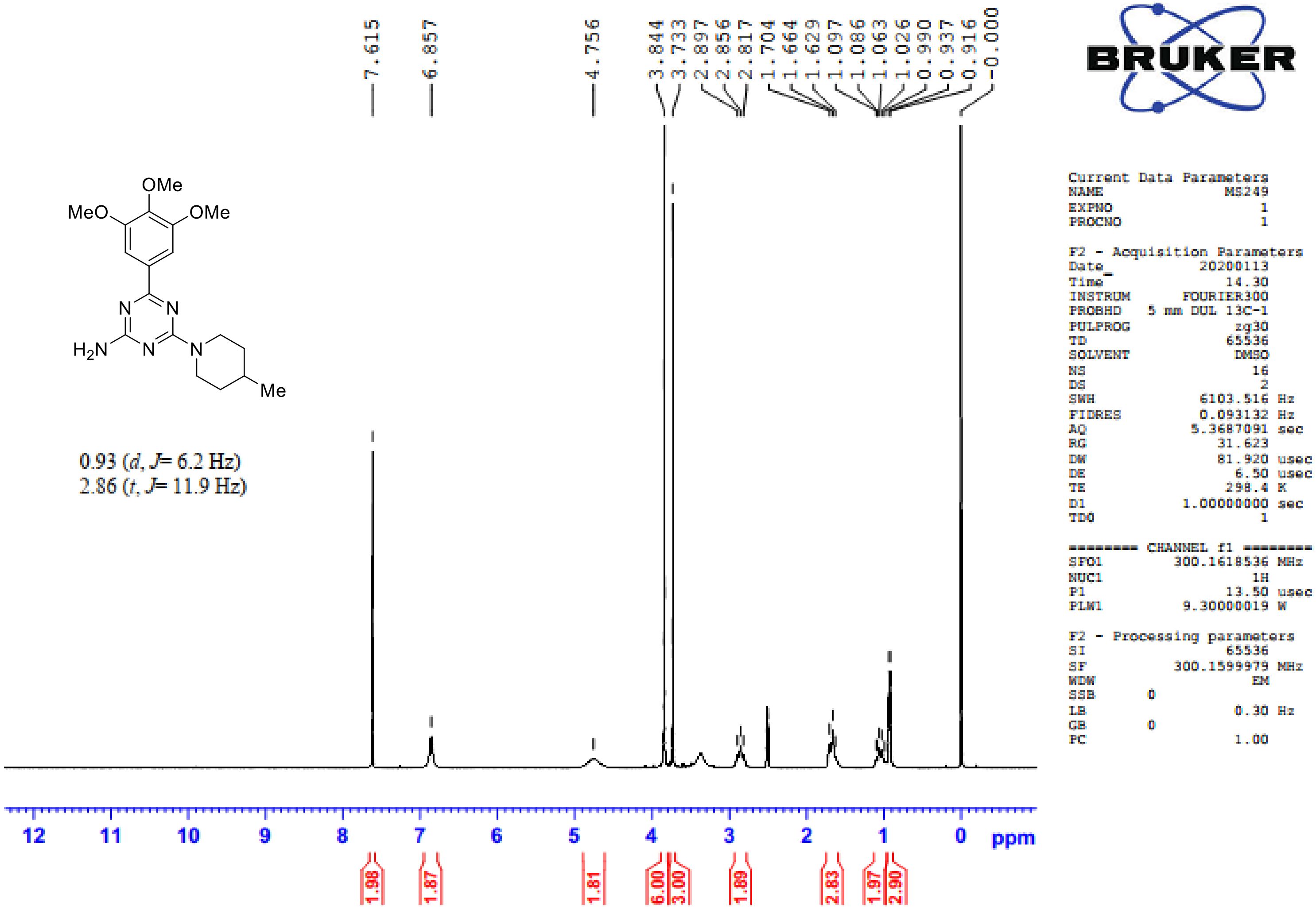

6-(3,4,5-Trimethoxyphenyl)-4-(4-methylpiperidino)-1,3,5-triazine-2-amine (**9**{*3*,*17*})

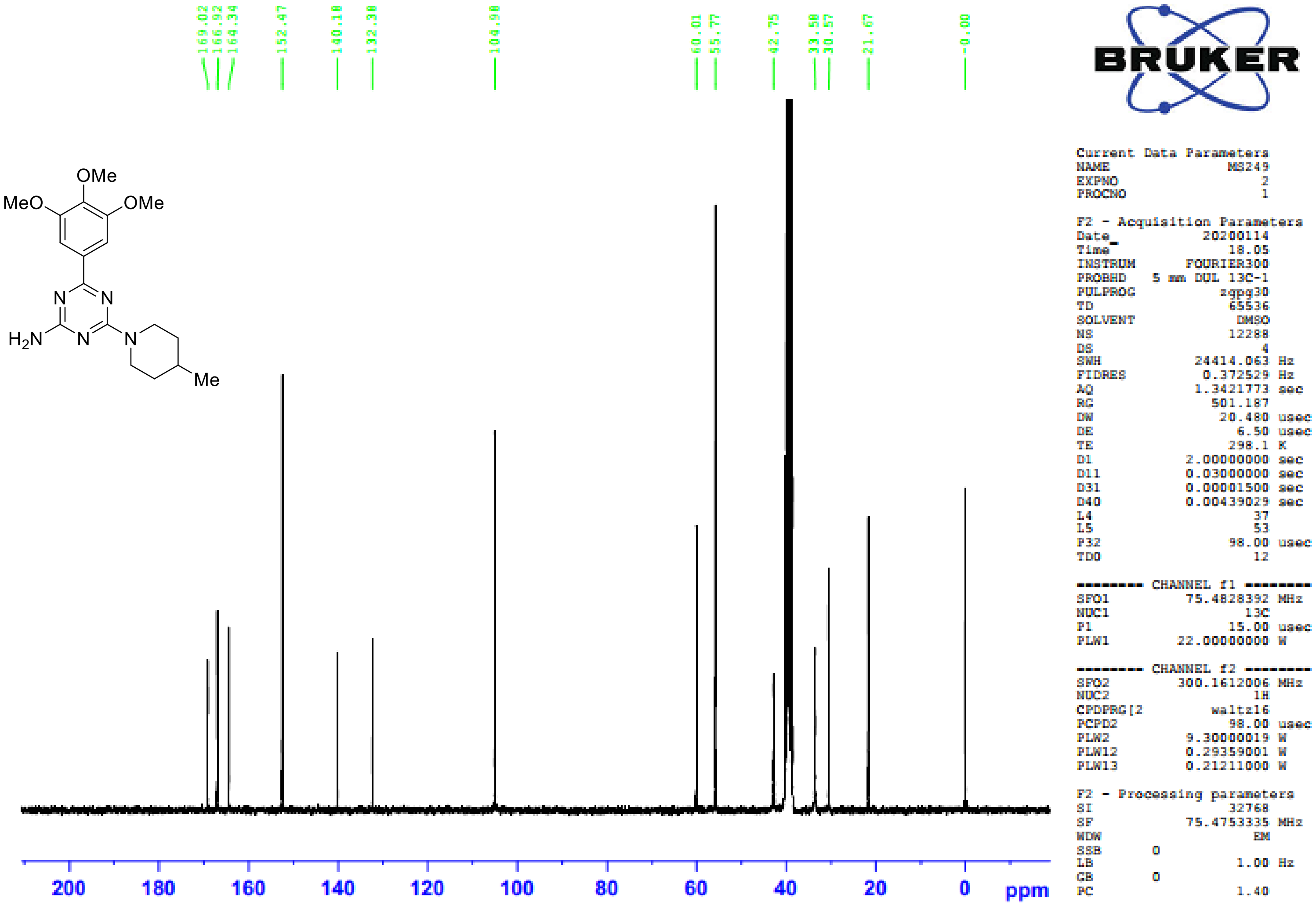

4-(4-Methylpiperidino)-6-(2-thienyl)-1,3,5-triazine-2-amine (**9**{*3*,*18*})

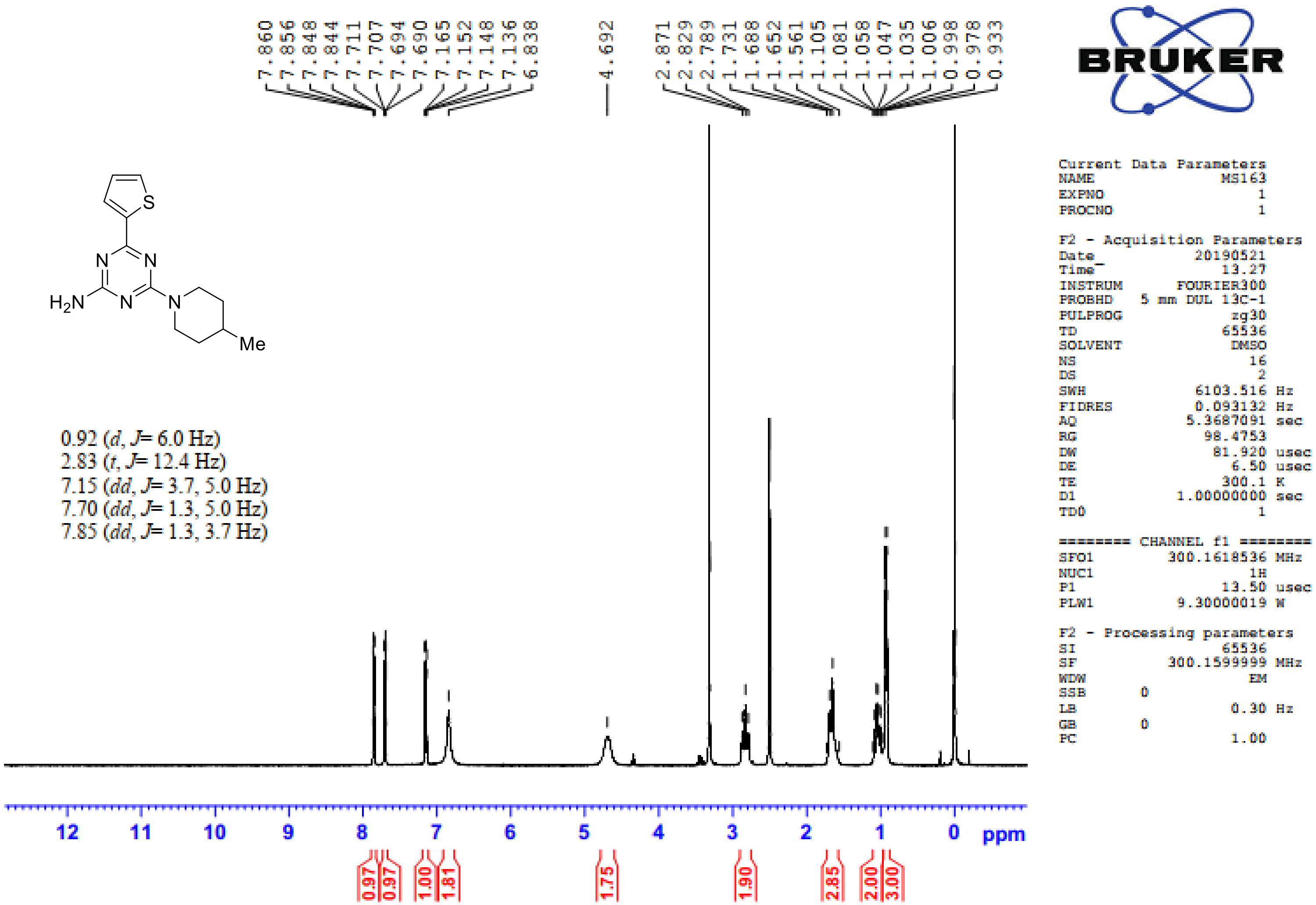

0.92 (*d*, *J*= 6.0 Hz)
2.83 (*t*, *J*= 12.4 Hz)
7.15 (*dd*, *J*= 3.7, 5.0 Hz)
7.70 (*dd*, *J*= 1.3, 5.0 Hz)
7.85 (*dd*, *J*= 1.3, 3.7 Hz)

4-(4-Methylpiperidino)-6-(2-thienyl)-1,3,5-triazine-2-amine (**9**{*3,18*})

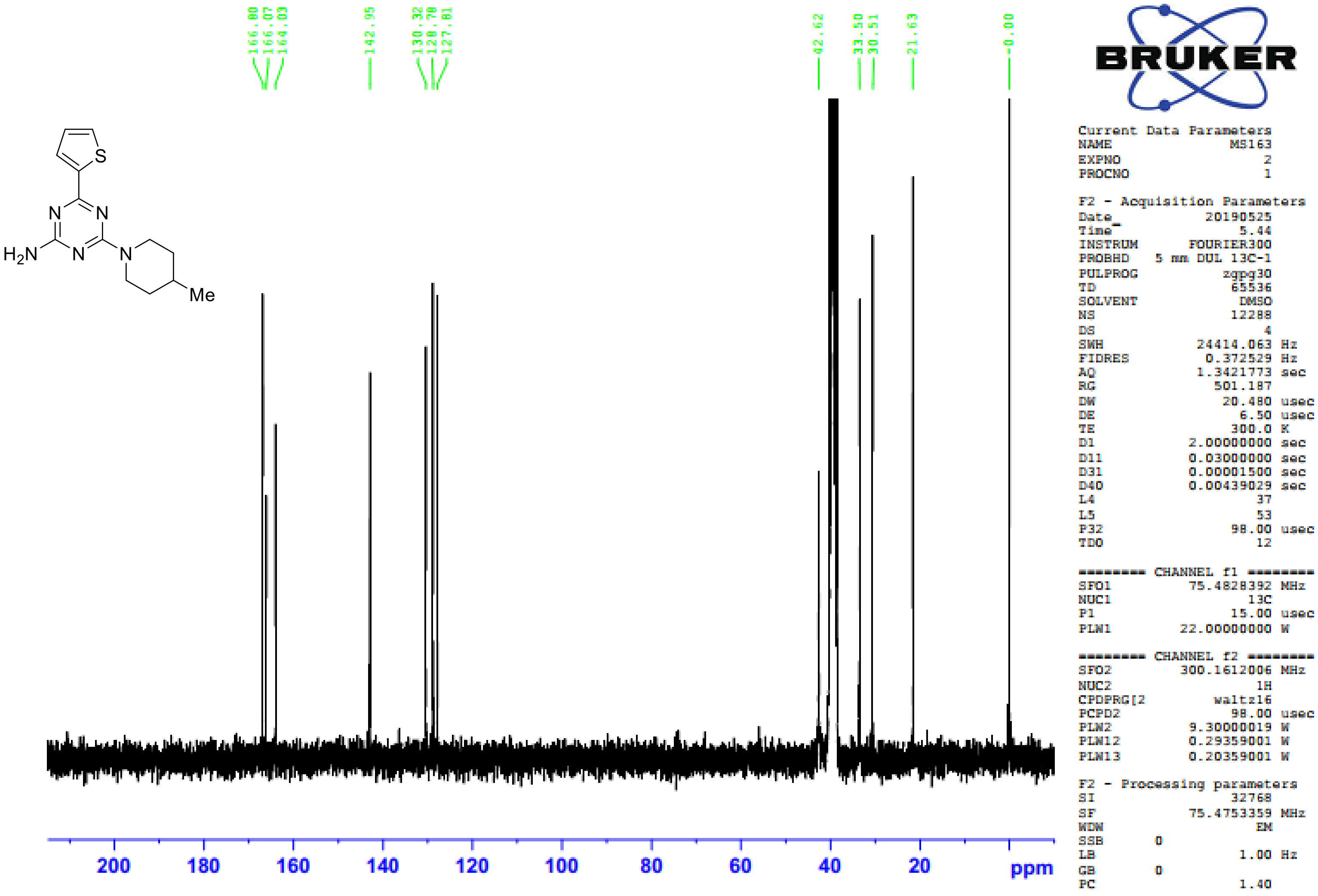

6-(3-Fluorophenyl)-4-(4-methylpiperazino)-1,3,5-triazine-2-amine (**9**{*4,2*})

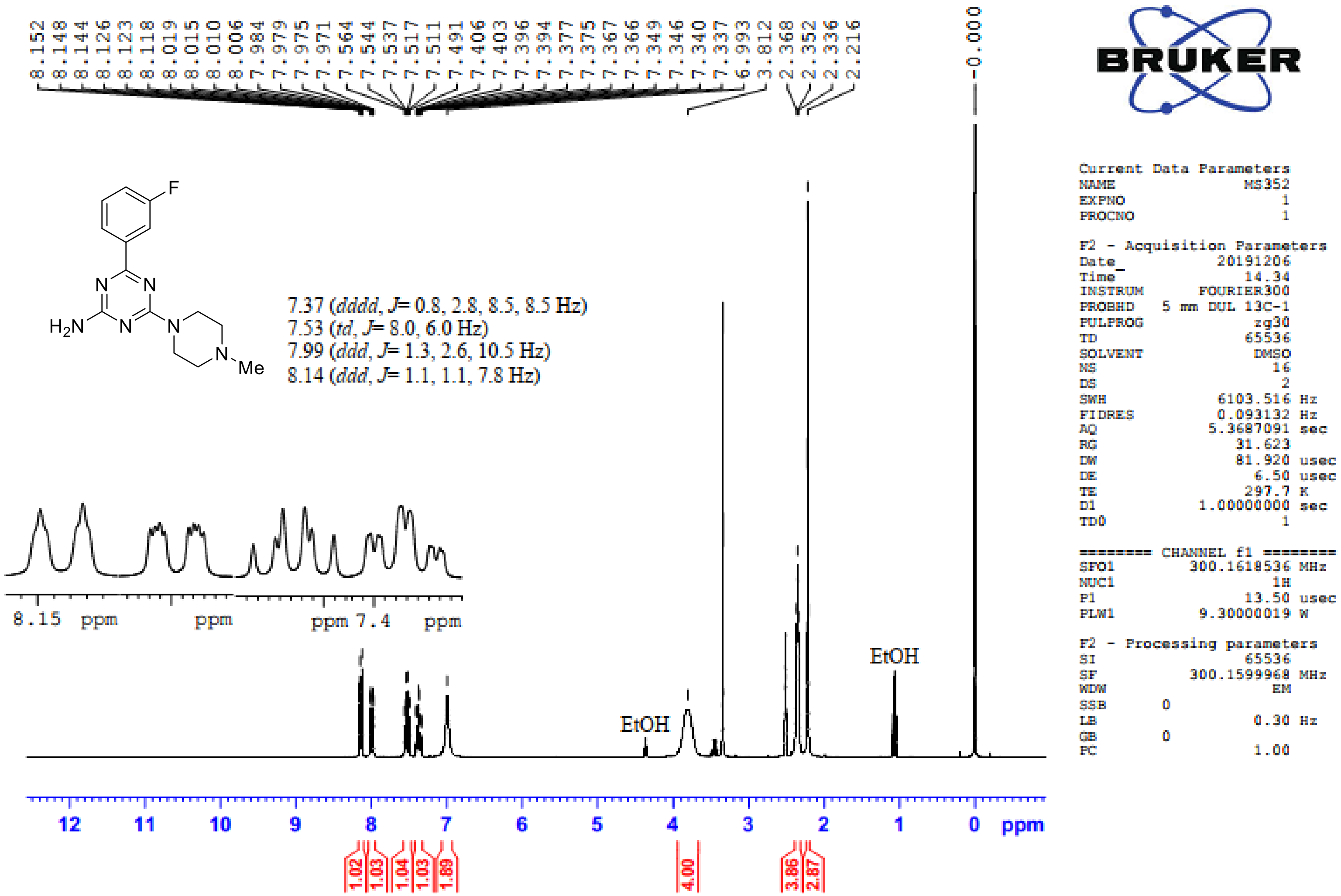

6-(3-Fluorophenyl)-4-(4-methylpiperazino)-1,3,5-triazine-2-amine (**9**{*4*,*2*})

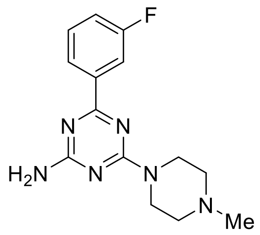

114.0 (*d*, *J*= 22.9 Hz)
117.9 (*d*, *J*= 20.9 Hz)
123.7 (*d*, *J*= 2.2 Hz)
130.2 (*d*, *J*= 8.2 Hz)
139.6 (*d*, *J*= 7.5 Hz)
162.1 (*d*, *J*= 242.9 Hz)

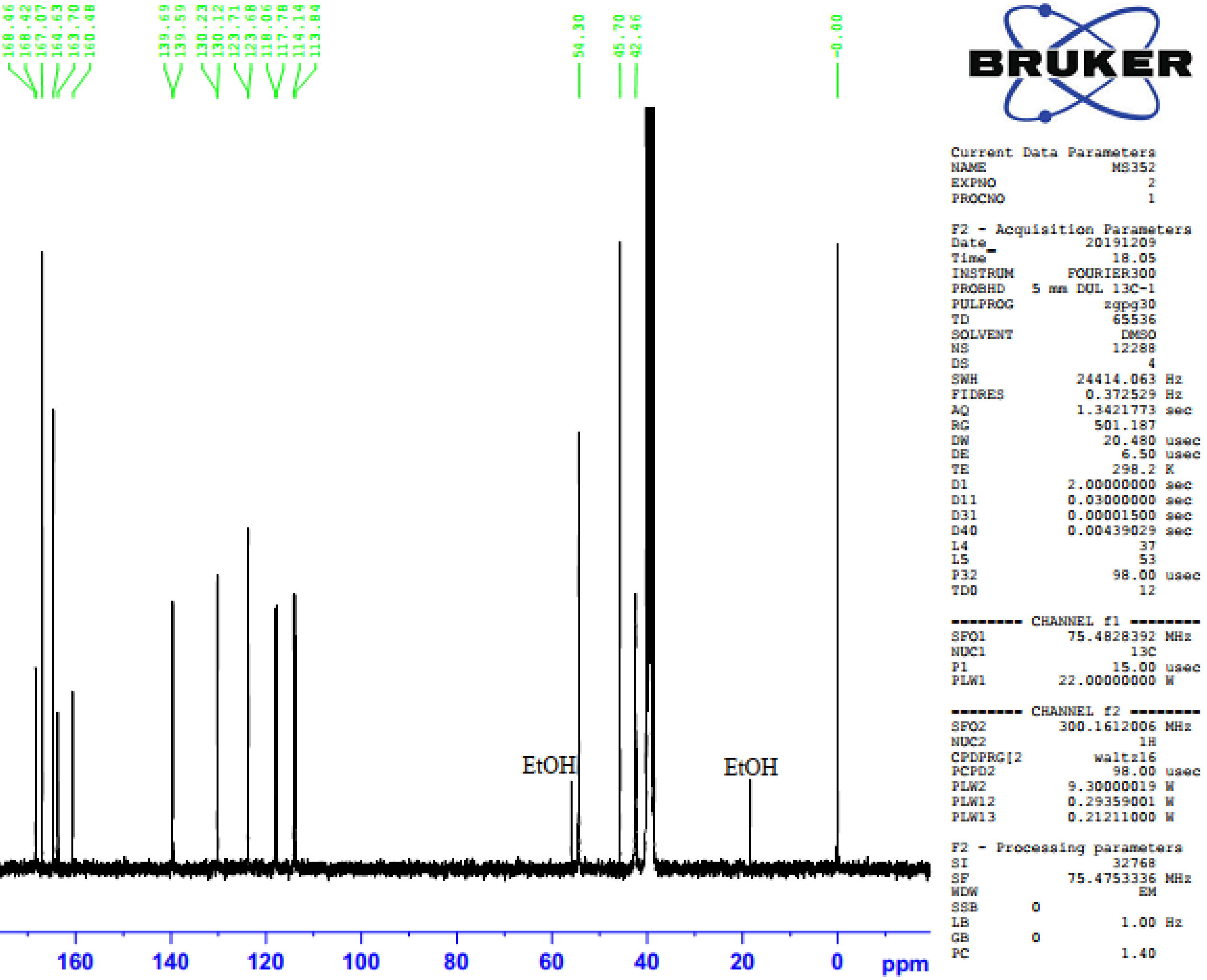

6-(4-Fluorophenyl)-4-(4-methylpiperazino)-1,3,5-triazine-2-amine (**9**{*4,3*})

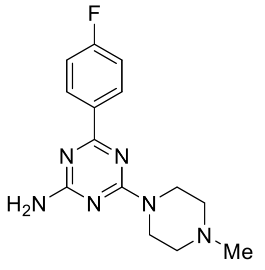

7.29 (*dd*, *J*= 8.9, 8.9 Hz)
8.34 (*dd*, *J*= 5.8, 9.0 Hz)

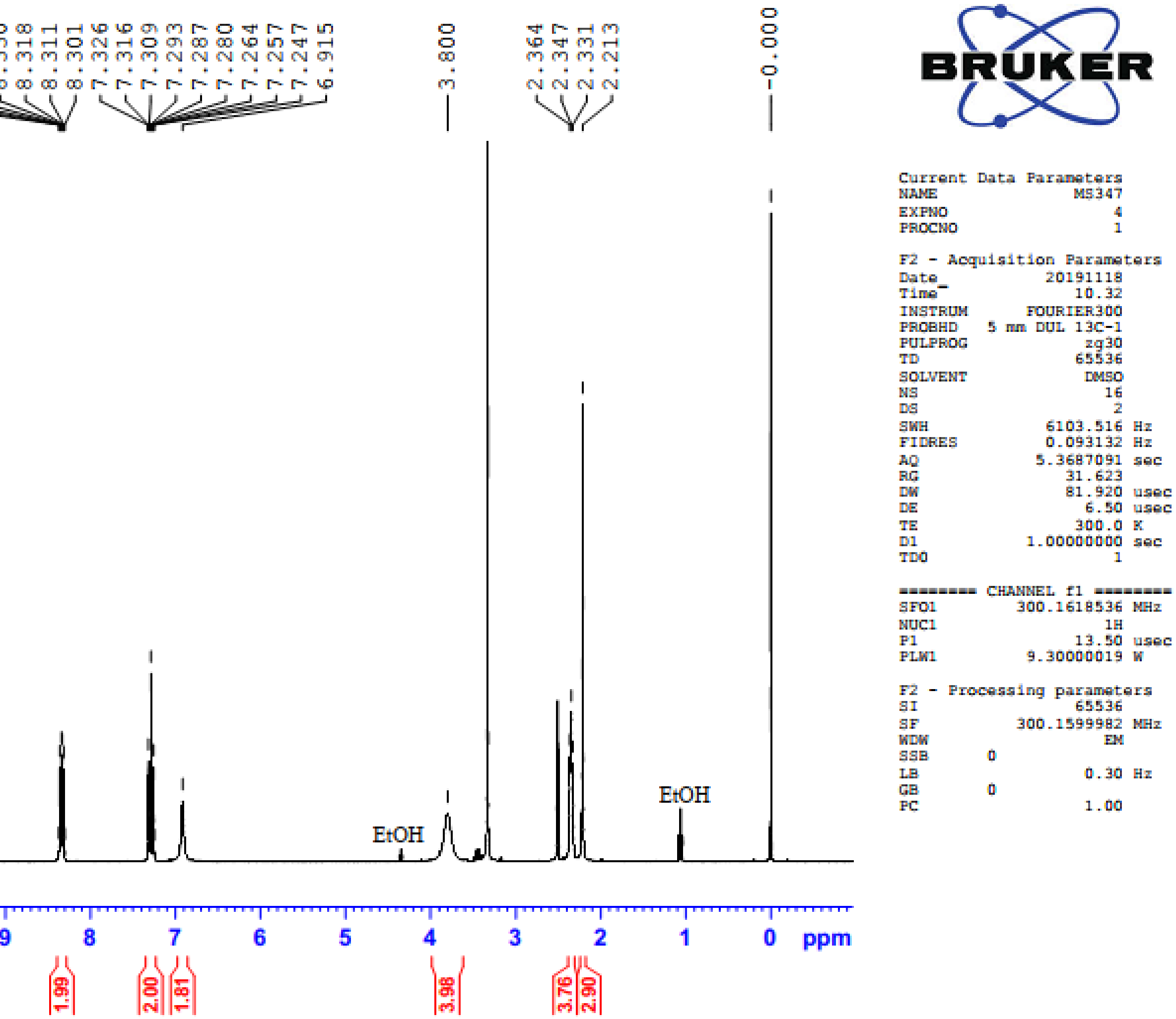

6-(4-Fluorophenyl)-4-(4-methylpiperazino)-1,3,5-triazine-2-amine (**9**{*4*,*3*})

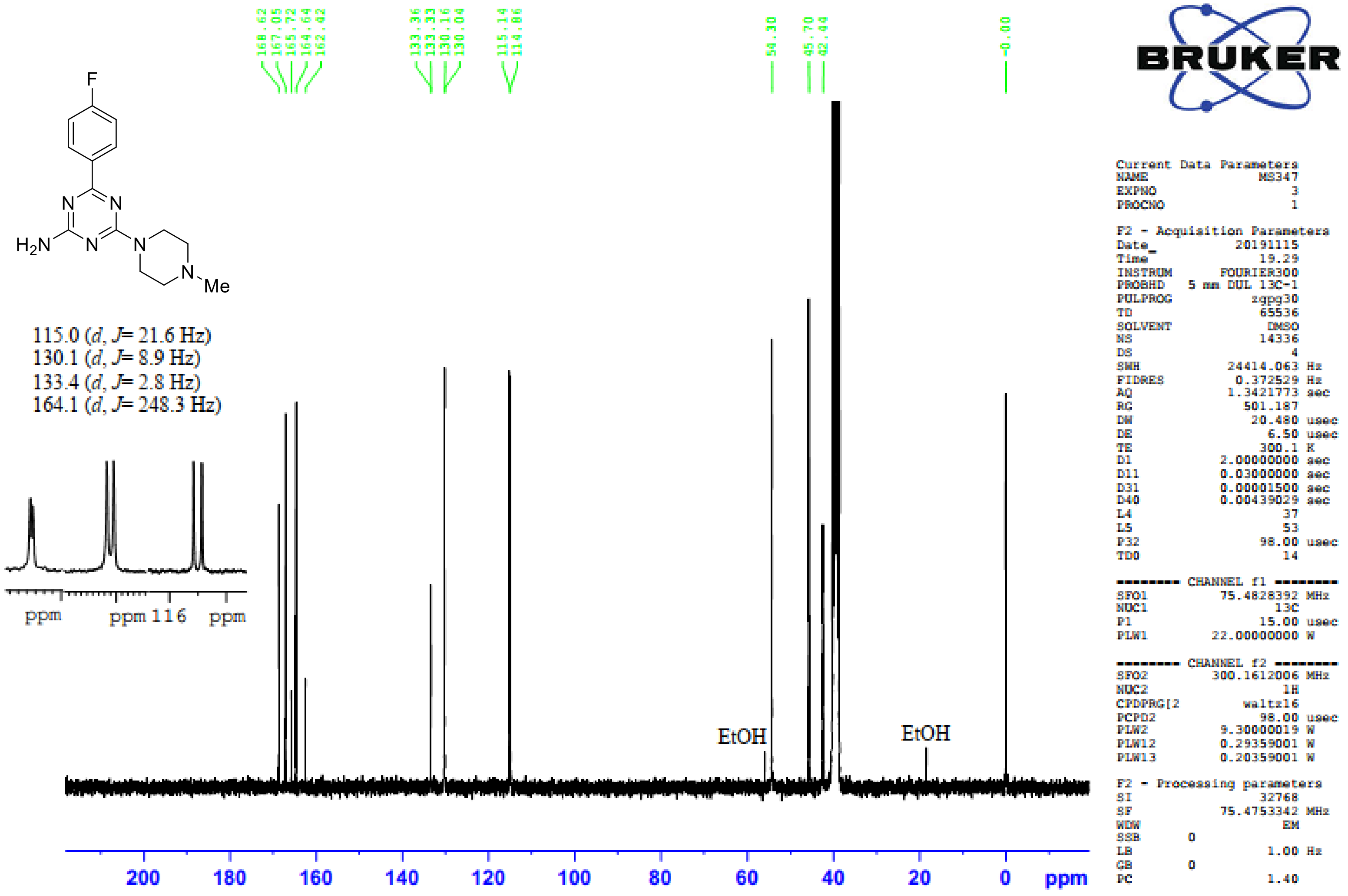

6-(4-Chlorophenyl)-4-(4-methylpiperazino)-1,3,5-triazine-2-amine (**9**{*4*,*4*})

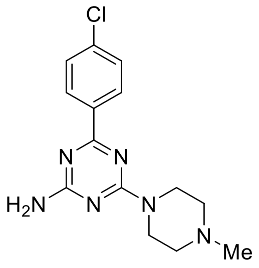

7.53 (*d*, *J*= 8.6 Hz)
8.29 (*d*, *J*= 8.7 Hz)

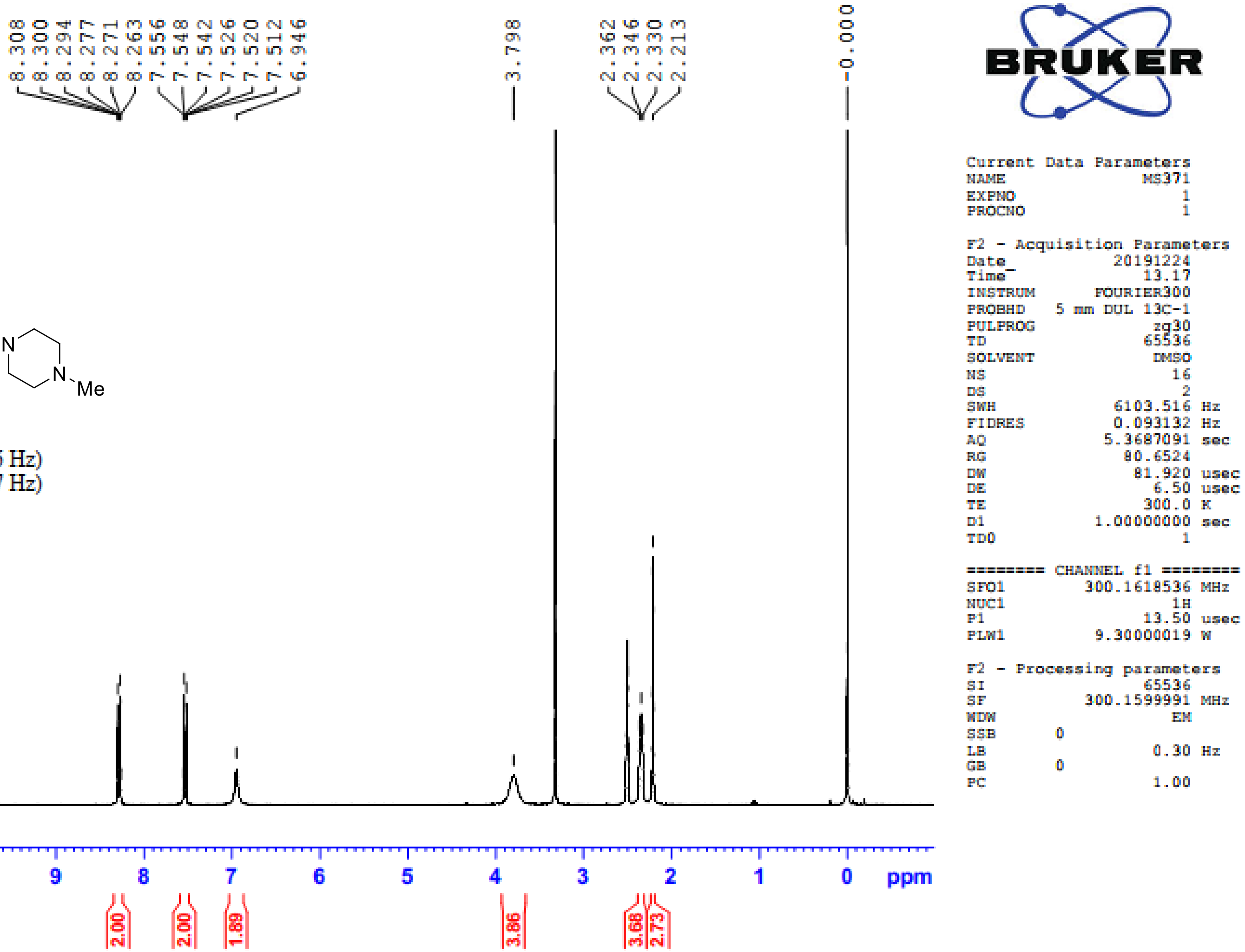

6-(4-Chlorophenyl)-4-(4-methylpiperazino)-1,3,5-triazine-2-amine (**9**{*4,4*})

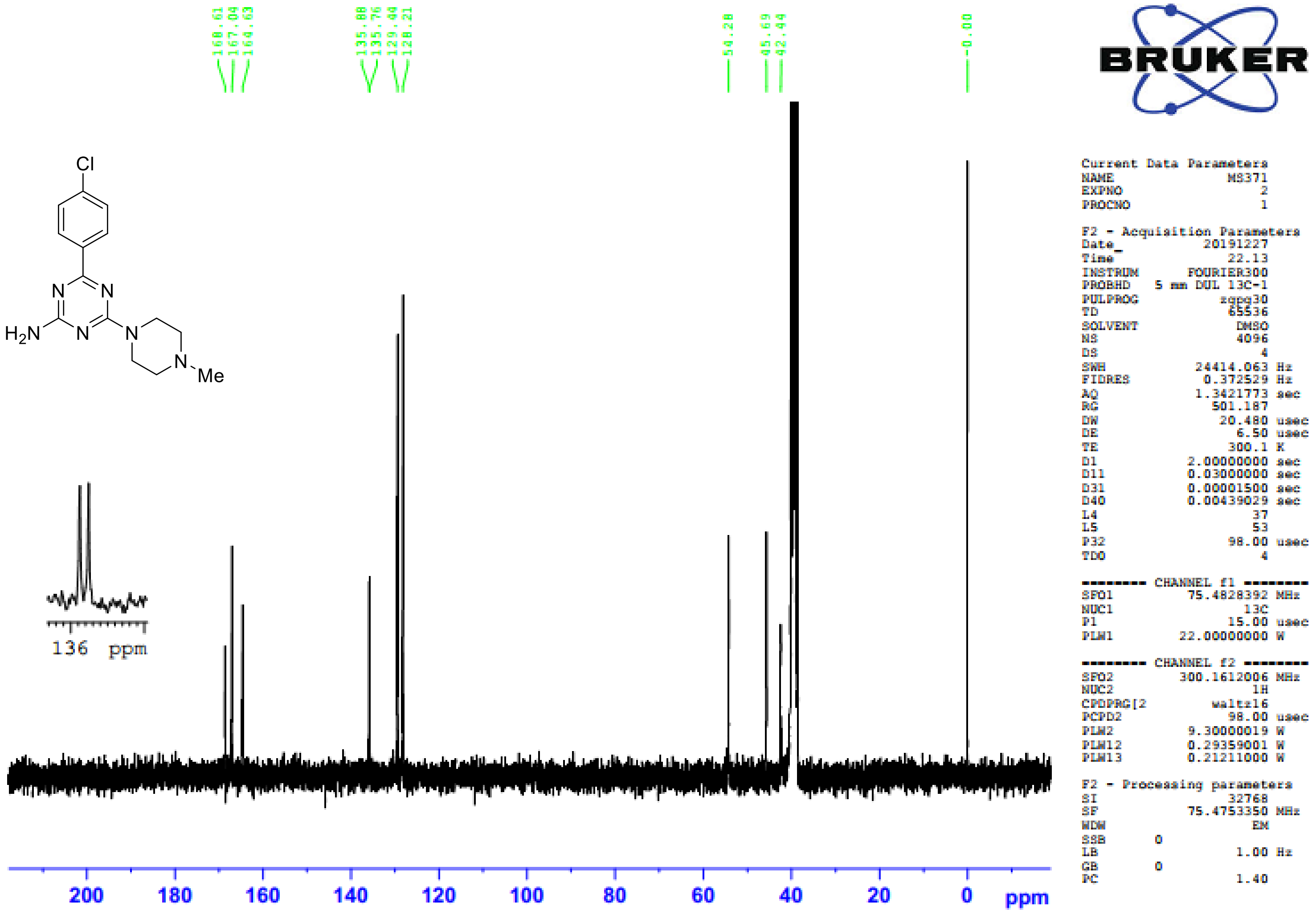

6-(3-Methylphenyl)-4-(4-methylpiperazino)-1,3,5-triazine-2-amine (**9**{*4*,*5*})

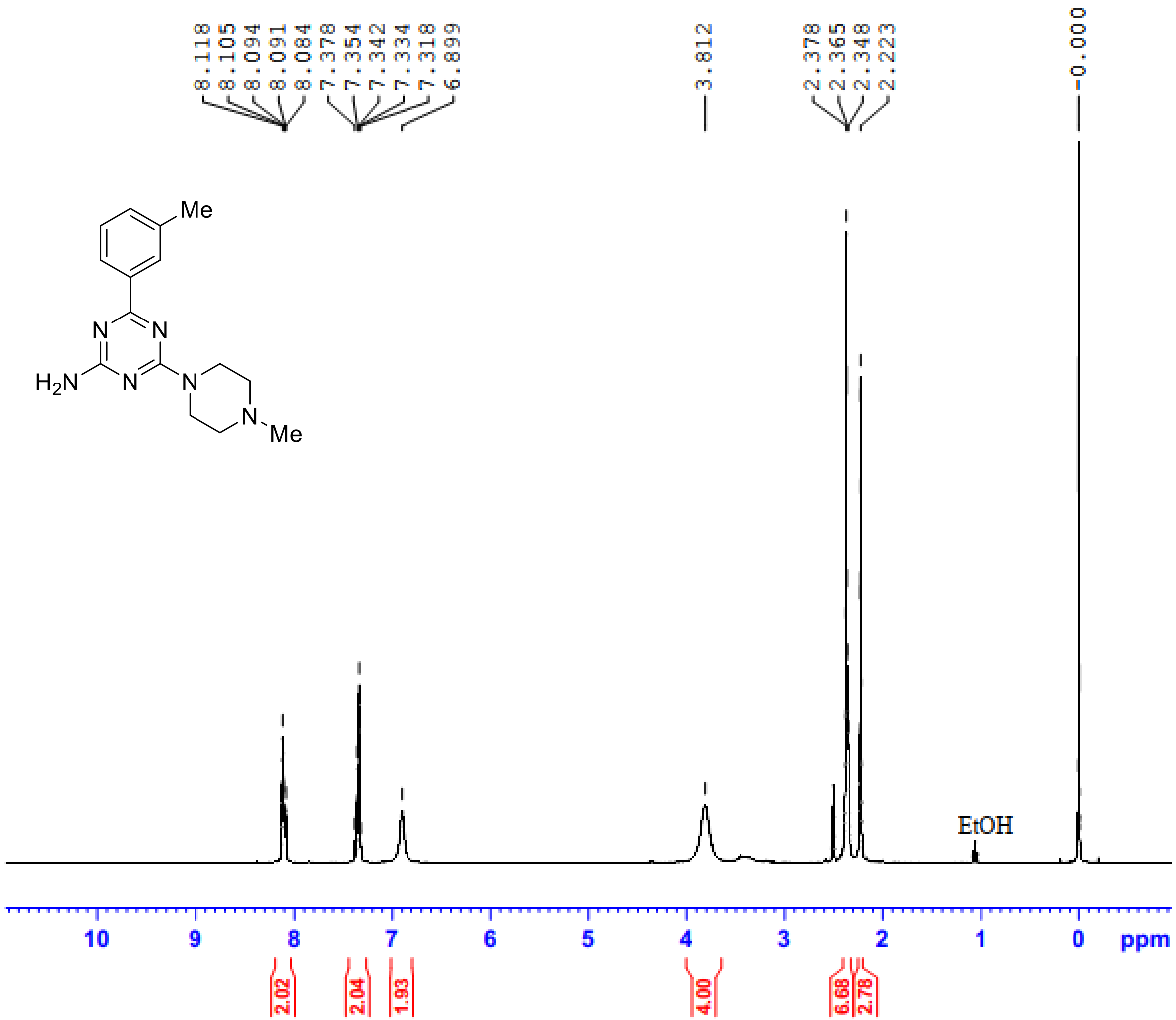


```
Current Data Parameters
NAME              MS349
EXPNO                 1
PROCNO                1

F2 - Acquisition Parameters
Date_          20191217
Time              15.41
INSTRUM     FOURIER300
PROBHD   5 mm DUL 13C-1
PULPROG            zg30
TD                65536
SOLVENT            DMSO
NS                   16
DS                    2
SWH            6103.516 Hz
FIDRES         0.093132 Hz
AQ            5.3687091 sec
RG              20.1113
DW               81.920 usec
DE                 6.50 usec
TE                298.2 K
D1           1.00000000 sec
TD0                   1

======== CHANNEL f1 ========
SFO1       300.1618536 MHz
NUC1                1H
P1               13.50 usec
PLW1        9.30000019 W

F2 - Processing parameters
SI                65536
SF          300.1599974 MHz
WDW                  EM
SSB      0
LB                 0.30 Hz
GB       0
PC                 1.00
```

6-(3-Methylphenyl)-4-(4-methylpiperazino)-1,3,5-triazine-2-amine (**9**{*4*,*5*})

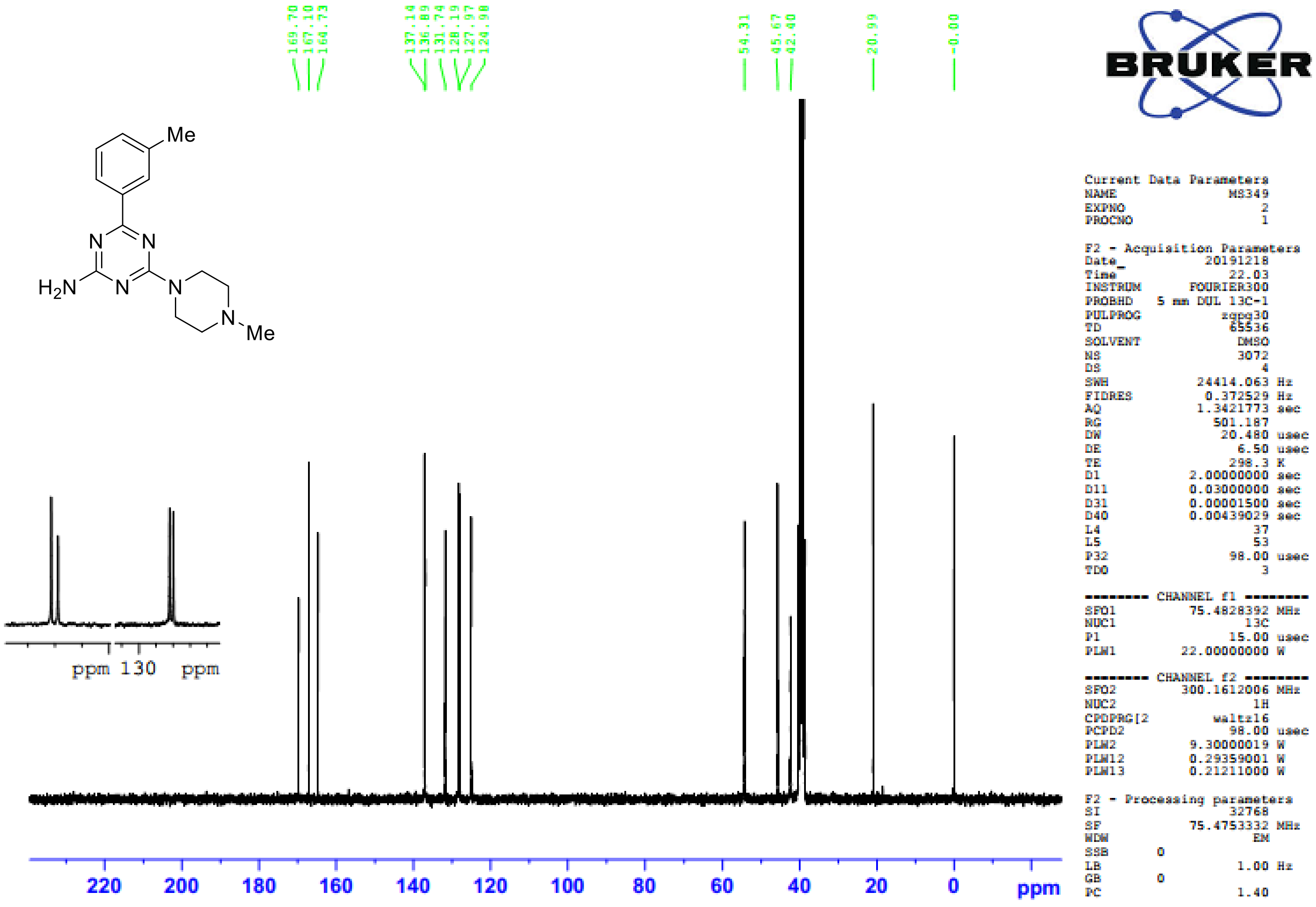

6-(4-Methylphenyl)-4-(4-methylpiperazino)-1,3,5-triazine-2-amine (**9**{*4*,*6*})

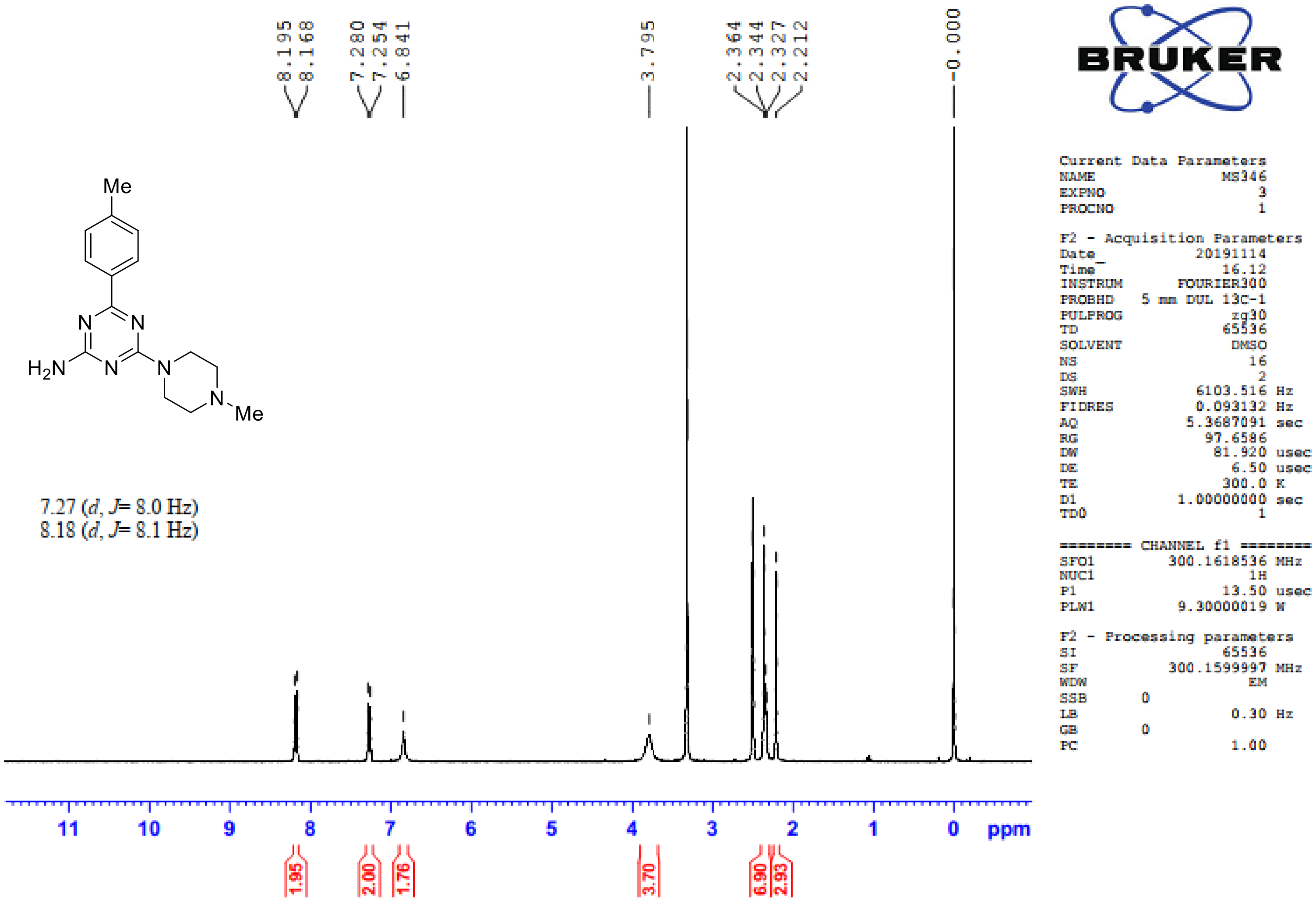

6-(4-Methylphenyl)-4-(4-methylpiperazino)-1,3,5-triazine-2-amine (**9**{*4*,*6*})

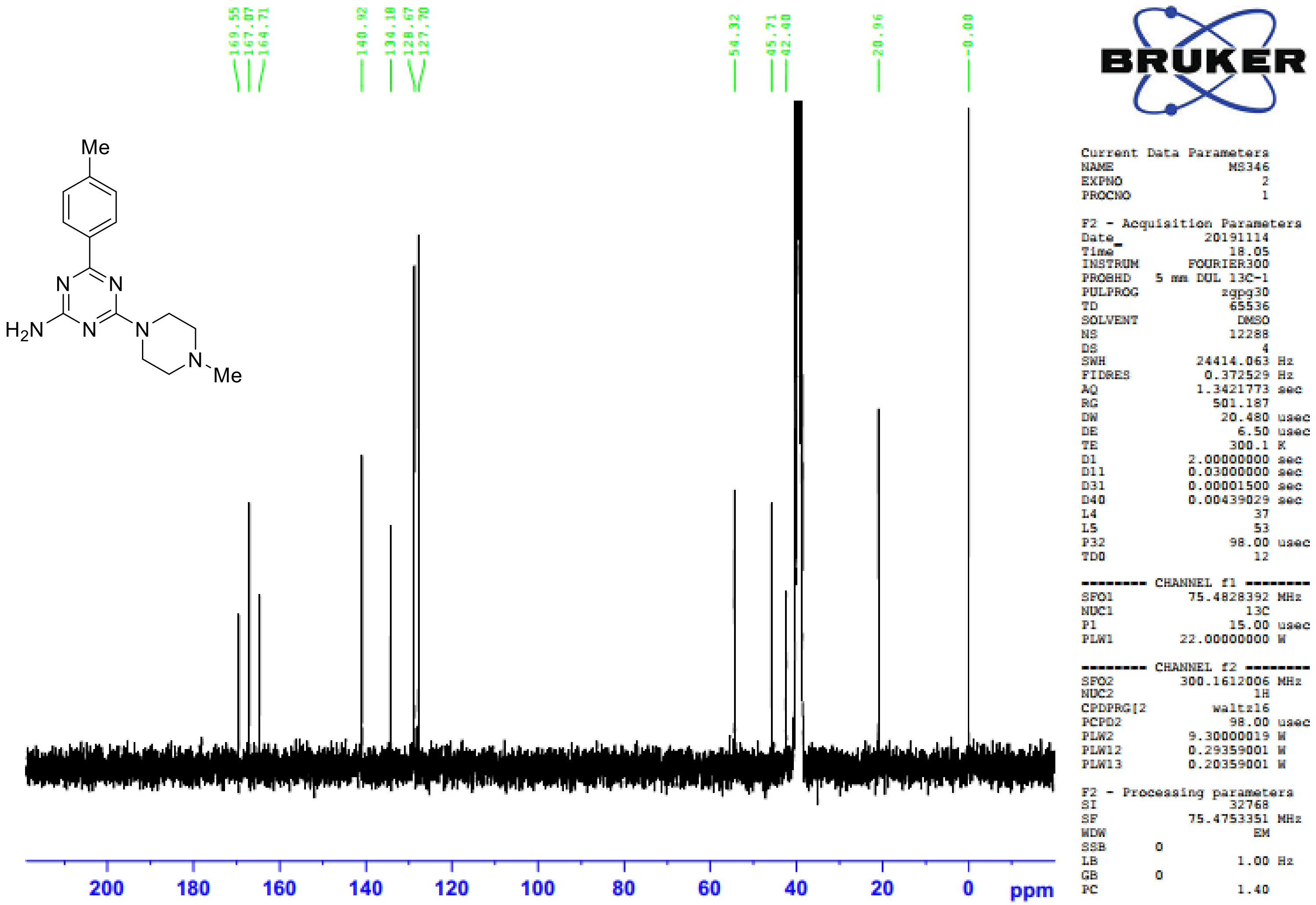

6-[4-(*tert*-Butyl)phenyl]-4-(4-methylpiperazino)-1,3,5-triazine-2-amine (**9**{*4,7*})

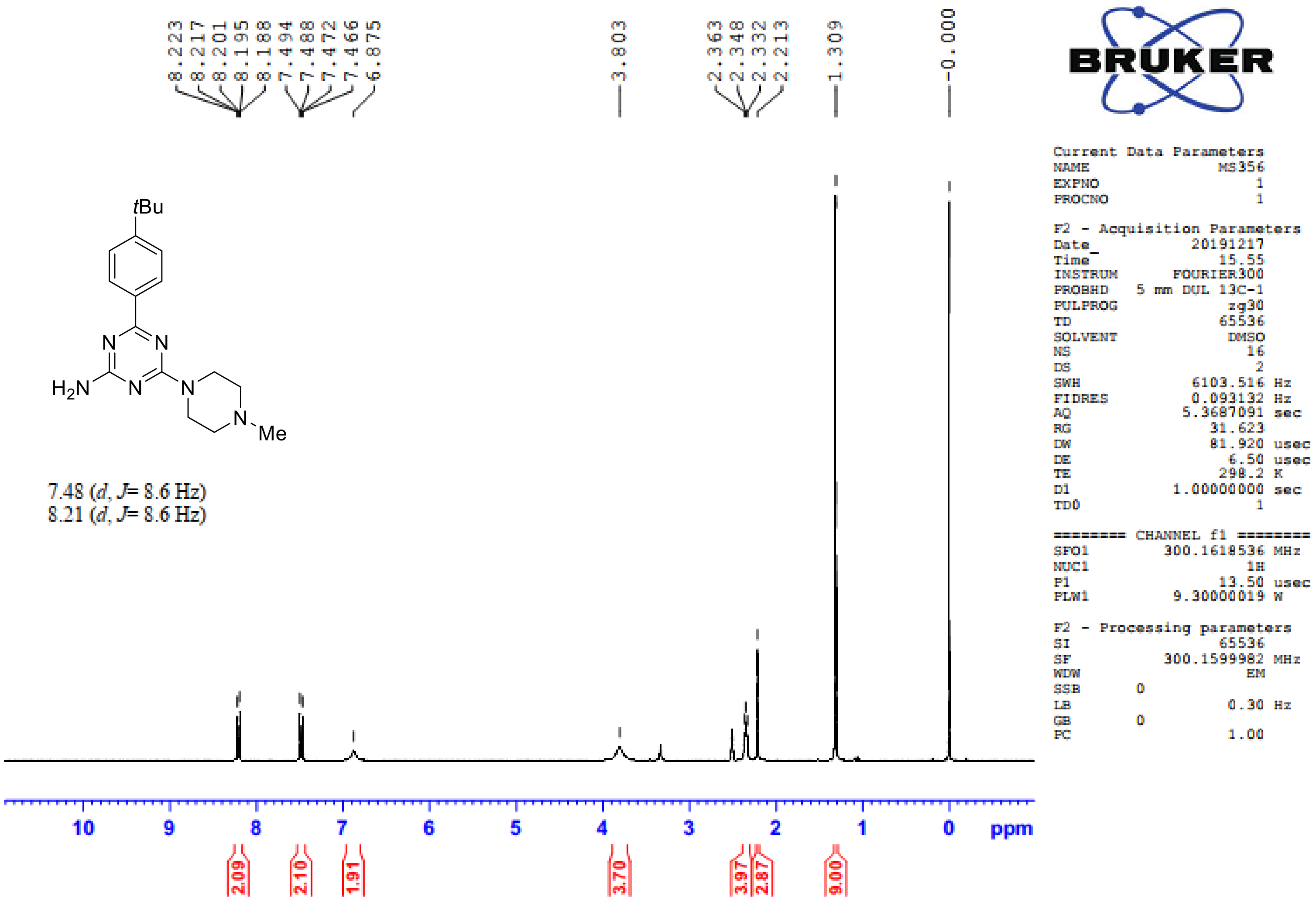

6-[4-(*tert*-Butyl)phenyl]-4-(4-methylpiperazino)-1,3,5-triazine-2-amine (**9**{*4,7*})

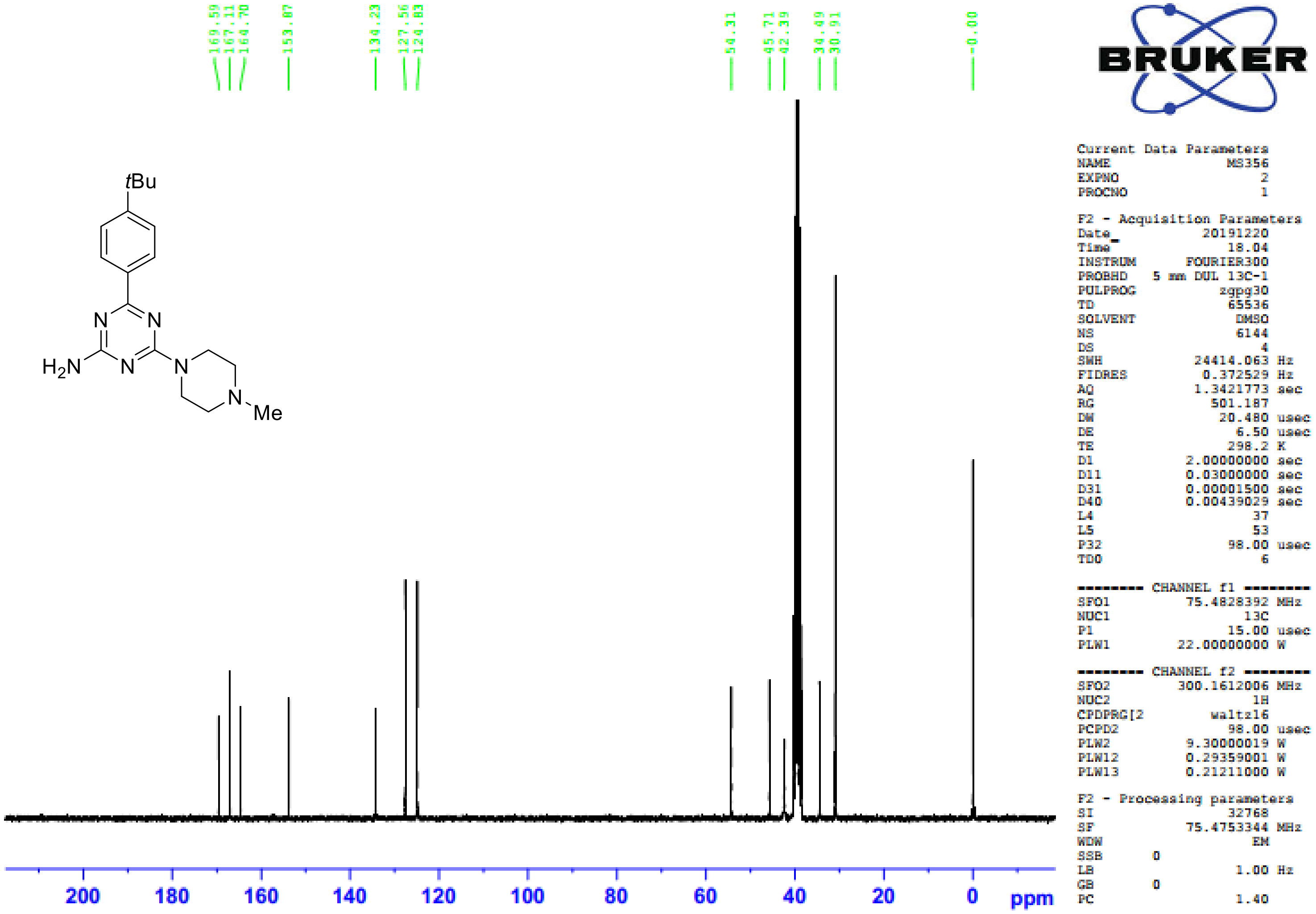

6-[4-(*N*,*N*-Dimethylamino)phenyl]-4-(4-methylpiperazino)-1,3,5-triazine-2-amine (**9**{*4,8*})

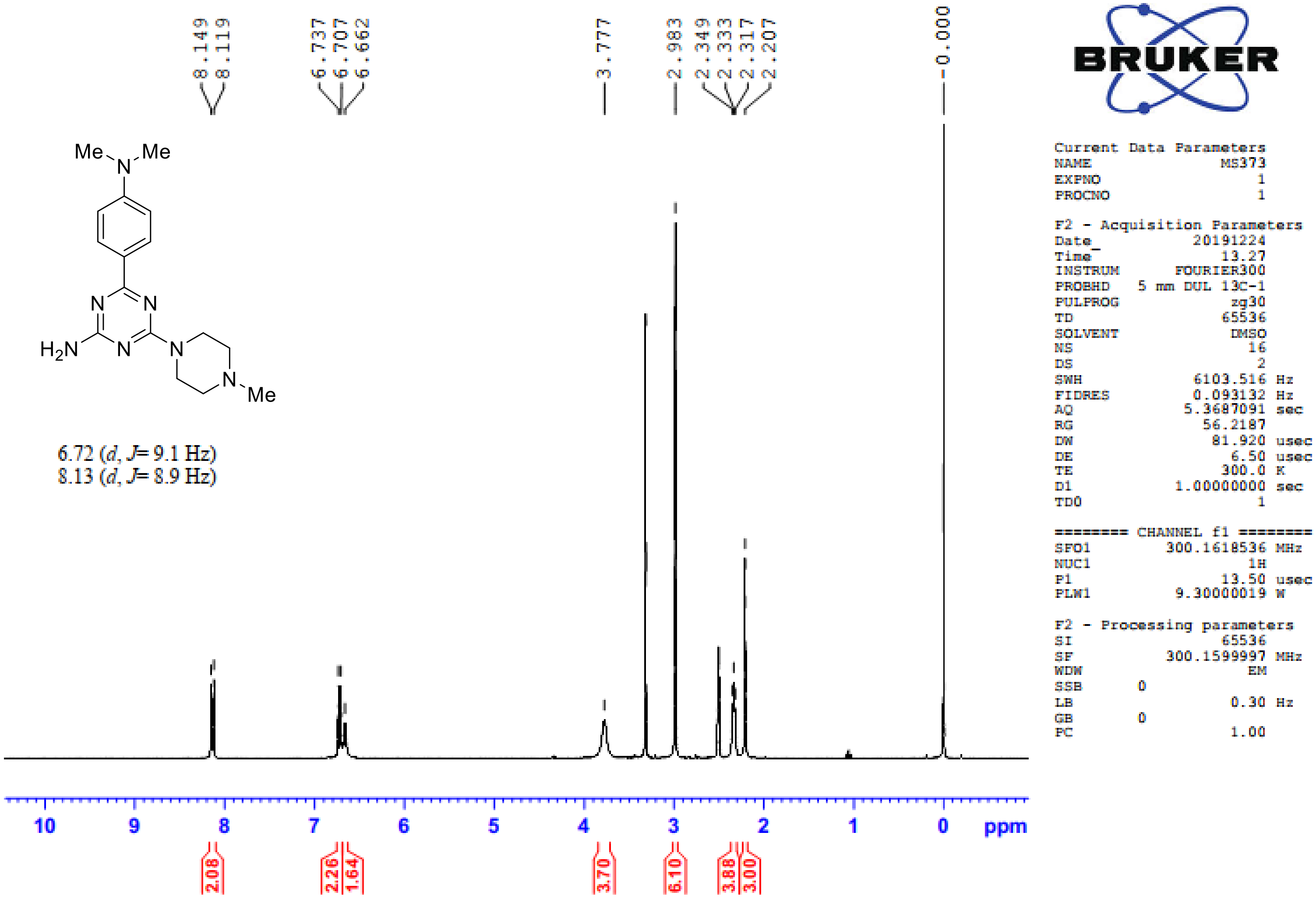

6-[4-(*N*,*N*-Dimethylamino)phenyl]-4-(4-methylpiperazino)-1,3,5-triazine-2-amine (**9**{*4*,*8*})

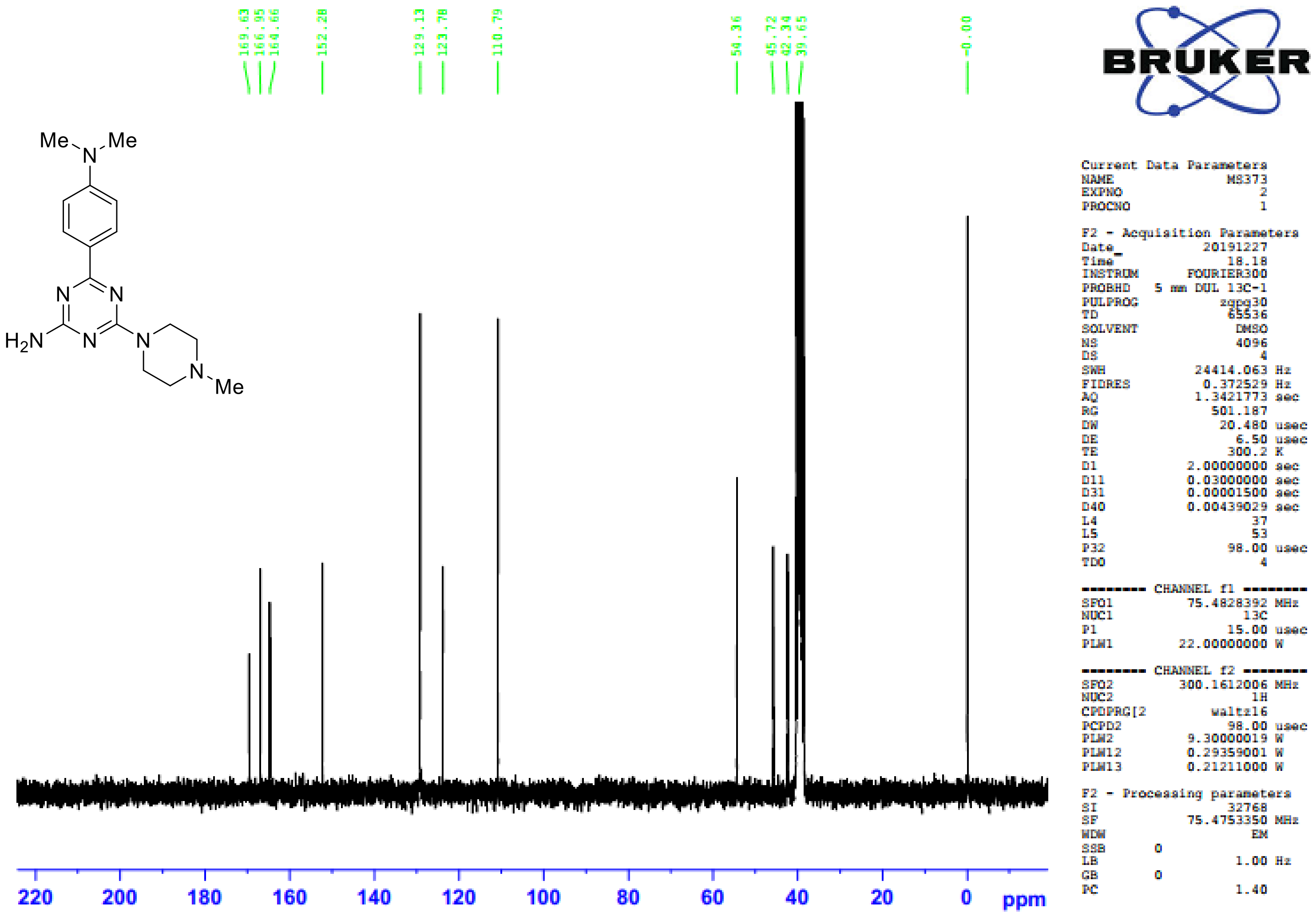

6-(4-Methoxyphenyl)-4-(4-methylpiperazino)-1,3,5-triazine-2-amine (**9**{*4,9*})

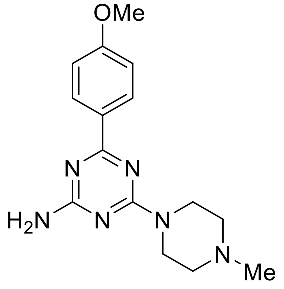

7.00 (*d*, *J*= 8.9 Hz)
8.25 (*d*, *J*= 8.9 Hz)

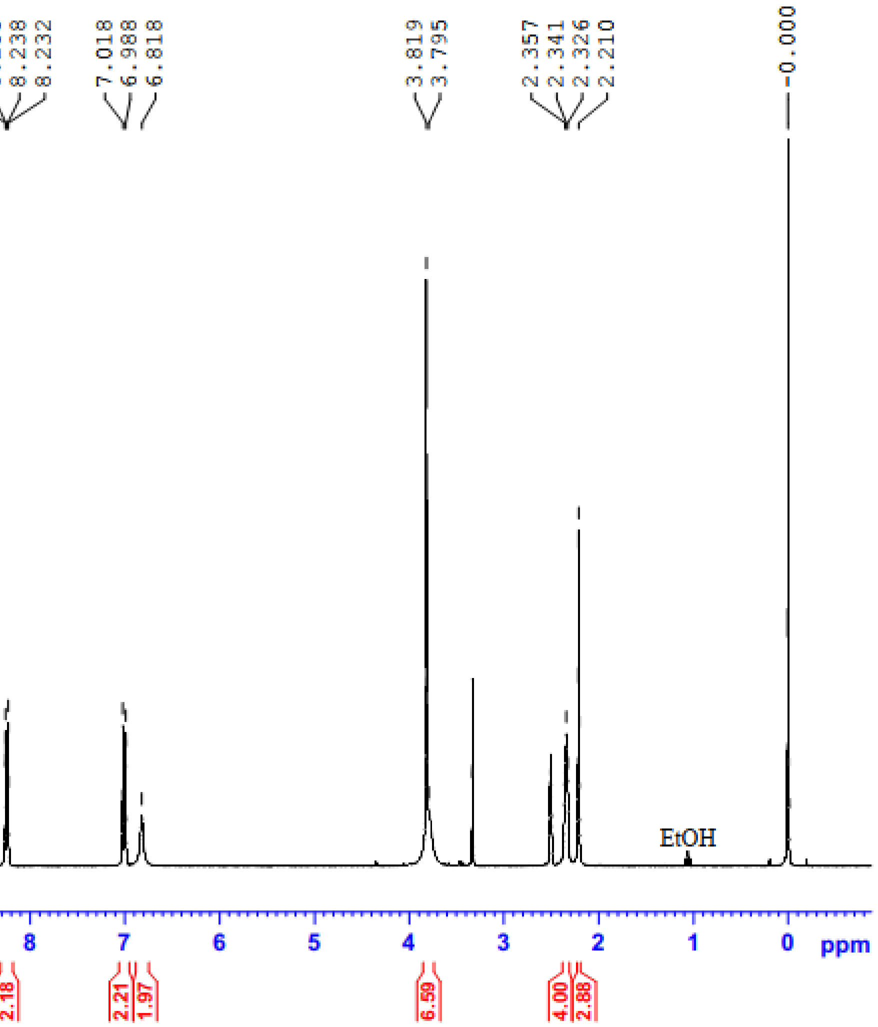


```
Current Data Parameters
NAME                  MS359
EXPNO                     1
PROCNO                    1

F2 - Acquisition Parameters
Date_              20191217
Time                  16.00
INSTRUM         FOURIER300
PROBHD   5 mm DUL 13C-1
PULPROG                zg30
TD                    65536
SOLVENT                DMSO
NS                       16
DS                        2
SWH                6103.516 Hz
FIDRES             0.093132 Hz
AQ                5.3687091 sec
RG                   31.623
DW                   81.920 usec
DE                     6.50 usec
TE                    298.1 K
D1               1.00000000 sec
TD0                       1

======== CHANNEL f1 ========
SFO1          300.1618536 MHz
NUC1                     1H
P1                    13.50 usec
PLW1             9.30000019 W

F2 - Processing parameters
SI                    65536
SF            300.1599985 MHz
WDW                      EM
SSB      0
LB                     0.30 Hz
GB       0
PC                     1.00
```

6-(4-Methoxyphenyl)-4-(4-methylpiperazino)-1,3,5-triazine-2-amine (**9**{*4*,*9*})

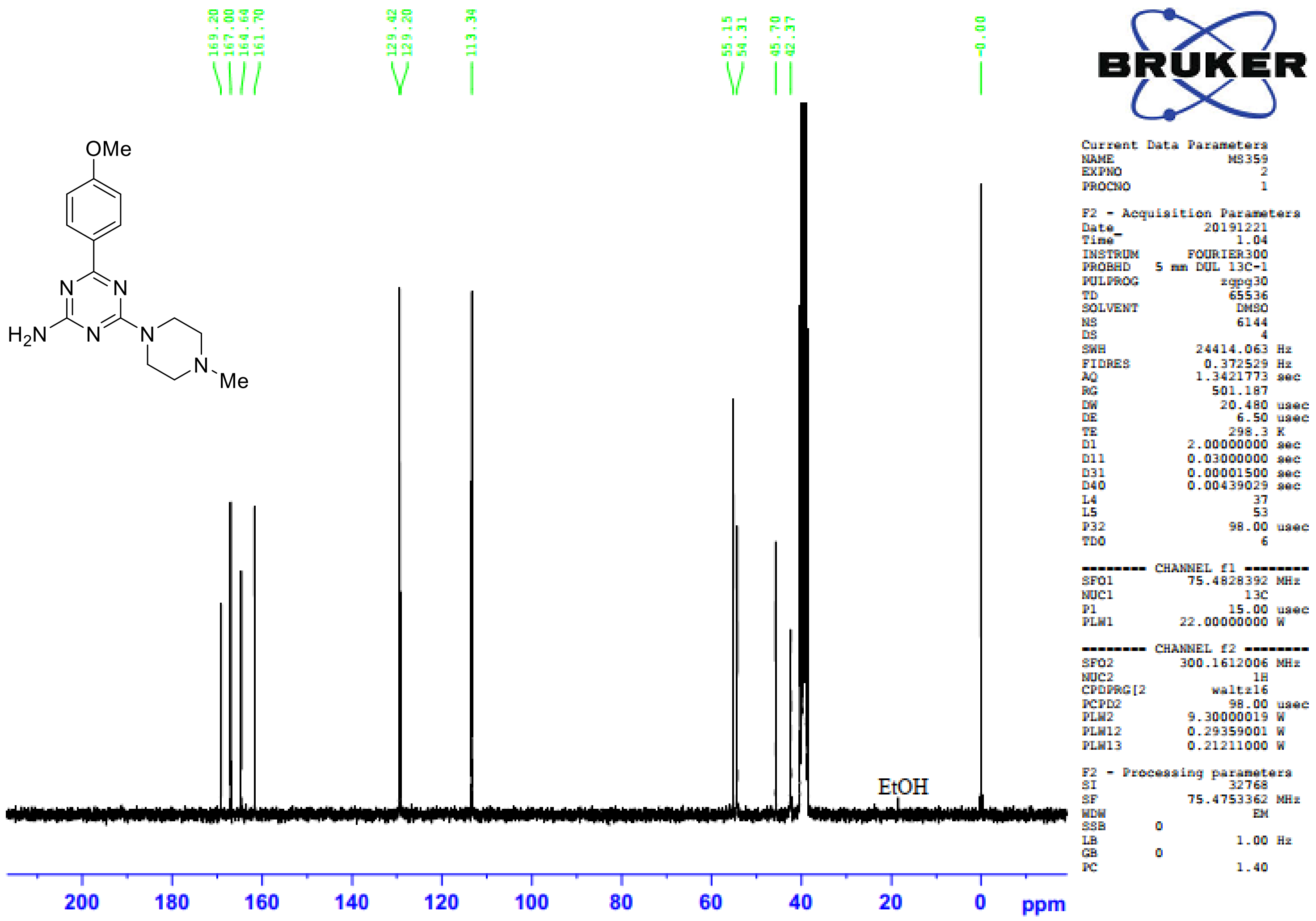

4-(4-Methylpiperazino)-6-(3-nitrophenyl)-1,3,5-triazine-2-amine (**9**{*4,10*})

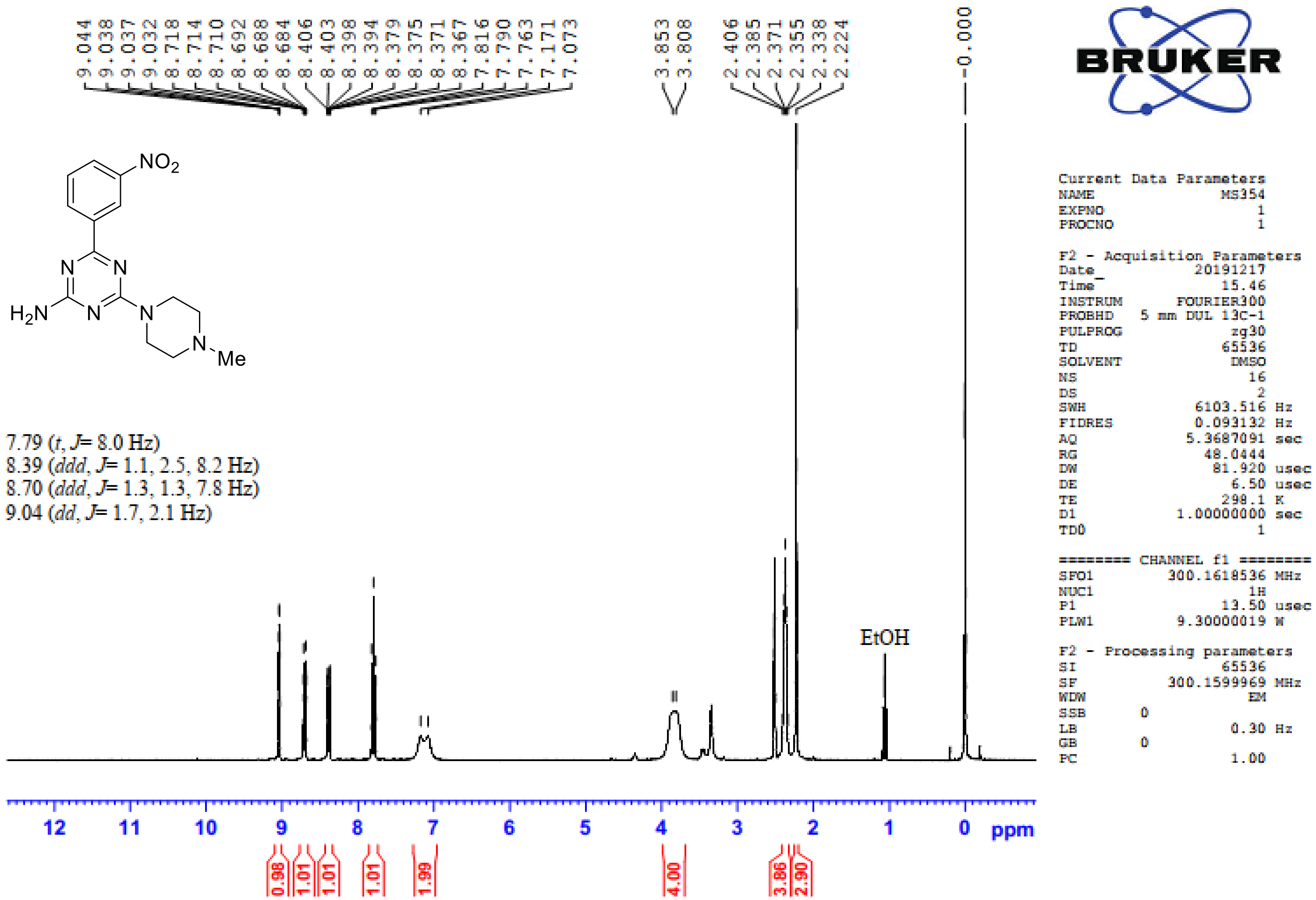

4-(4-Methylpiperazino)-6-(3-nitrophenyl)-1,3,5-triazine-2-amine (**9**{*4,10*})

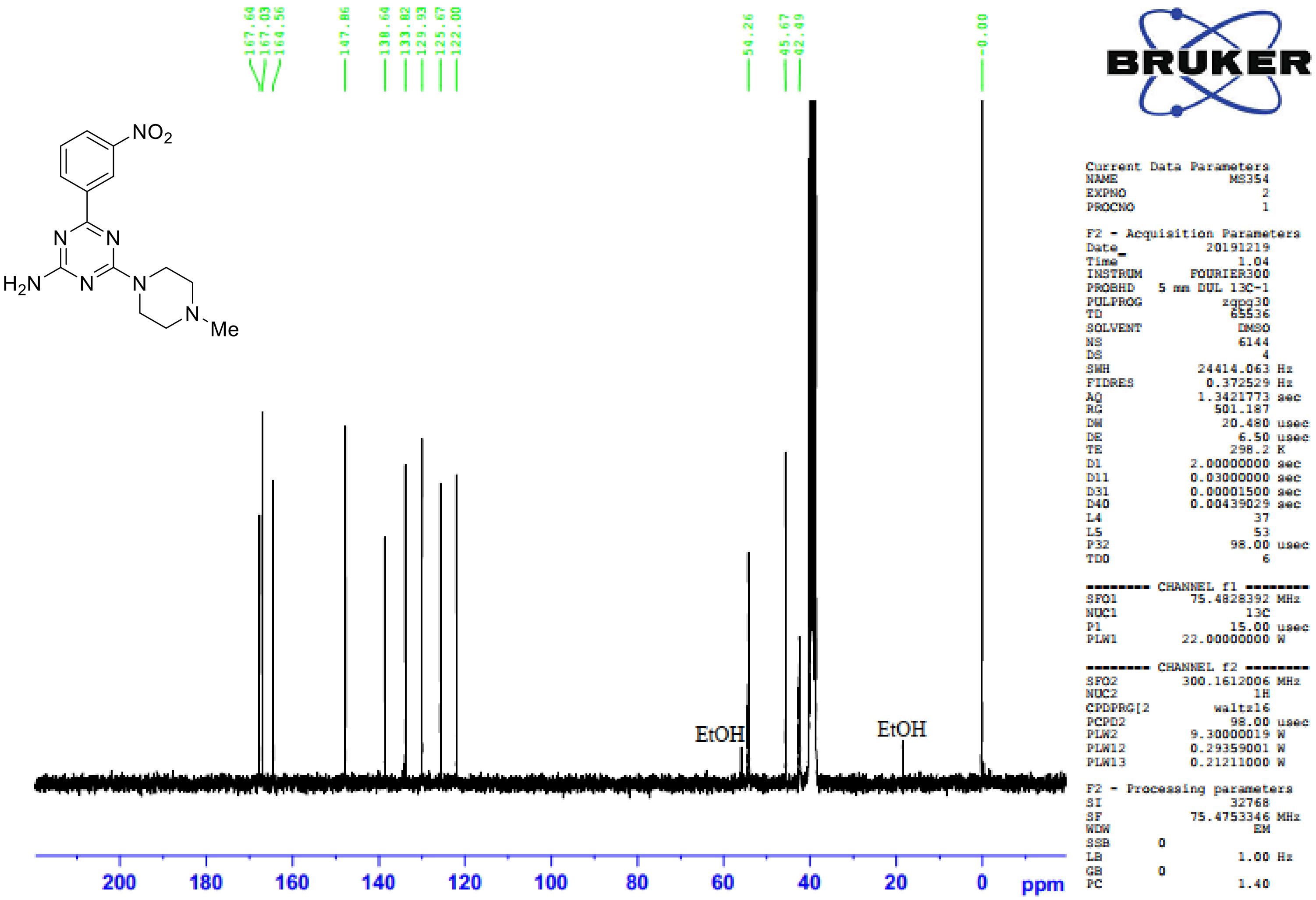

6-[4-(Trifluoromethyl)phenyl]-4-(4-methylpiperazino)-1,3,5-triazine-2-amine (**9**{*4,11*})

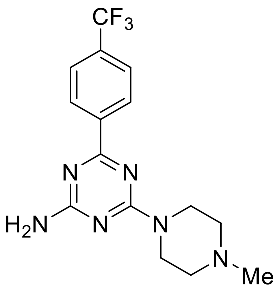

7.85 (*d*, *J*= 8.3 Hz)
8.48 (*d*, *J*= 8.3 Hz)

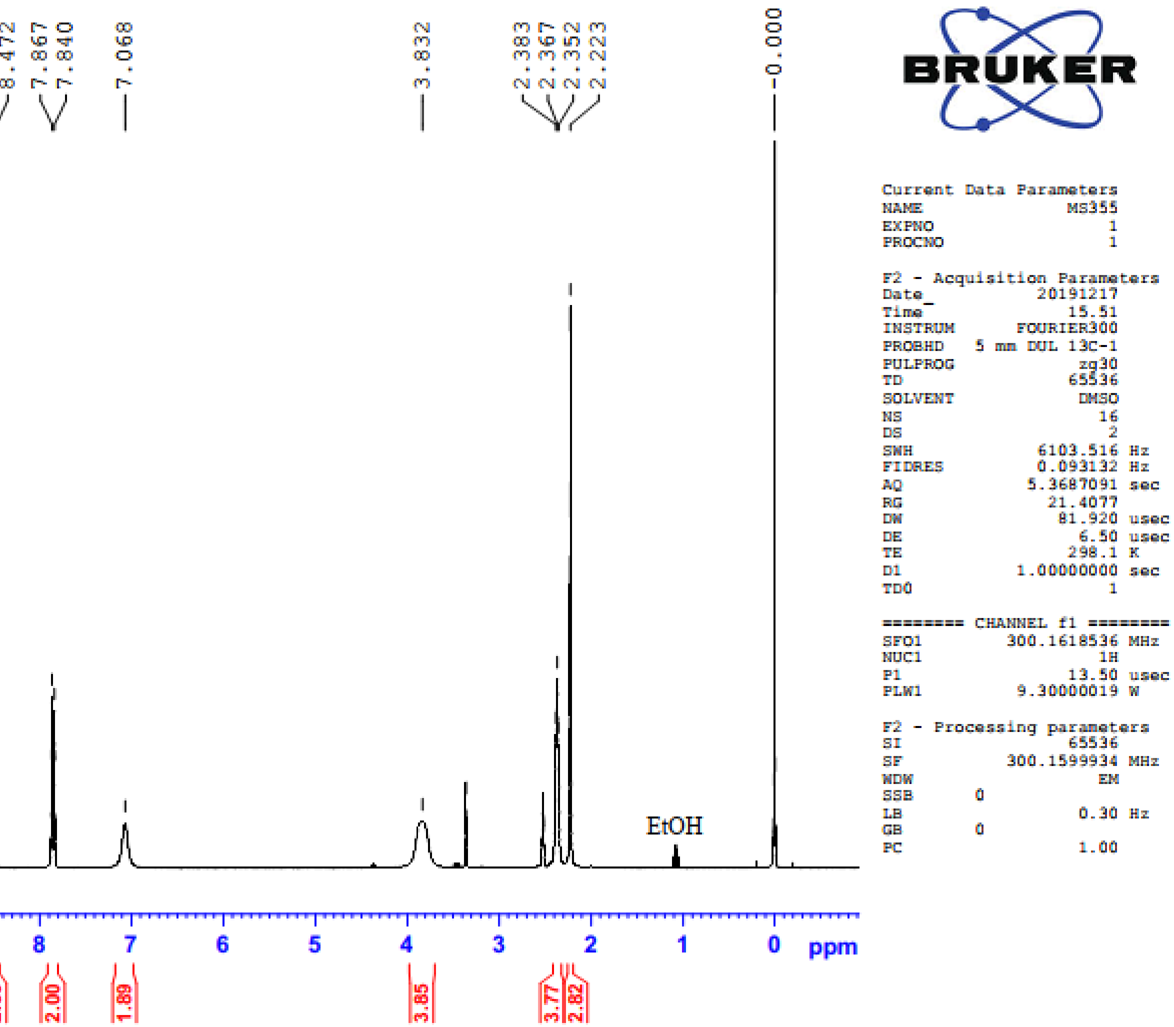

6-[4-(Trifluoromethyl)phenyl]-4-(4-methylpiperazino)-1,3,5-triazine-2-amine (**9**{*4*,*11*})

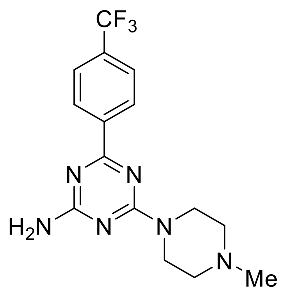

124.1 (*q*, *J*= 272.4 Hz)
125.1 (*q*, *J*= 3.5 Hz)
131.0 (*q*, *J*= 31.7 Hz)
140.9 (*q*, *J*= 1.5 Hz)

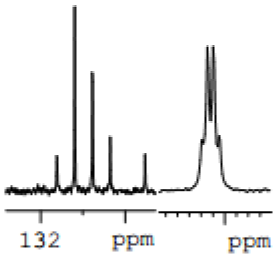

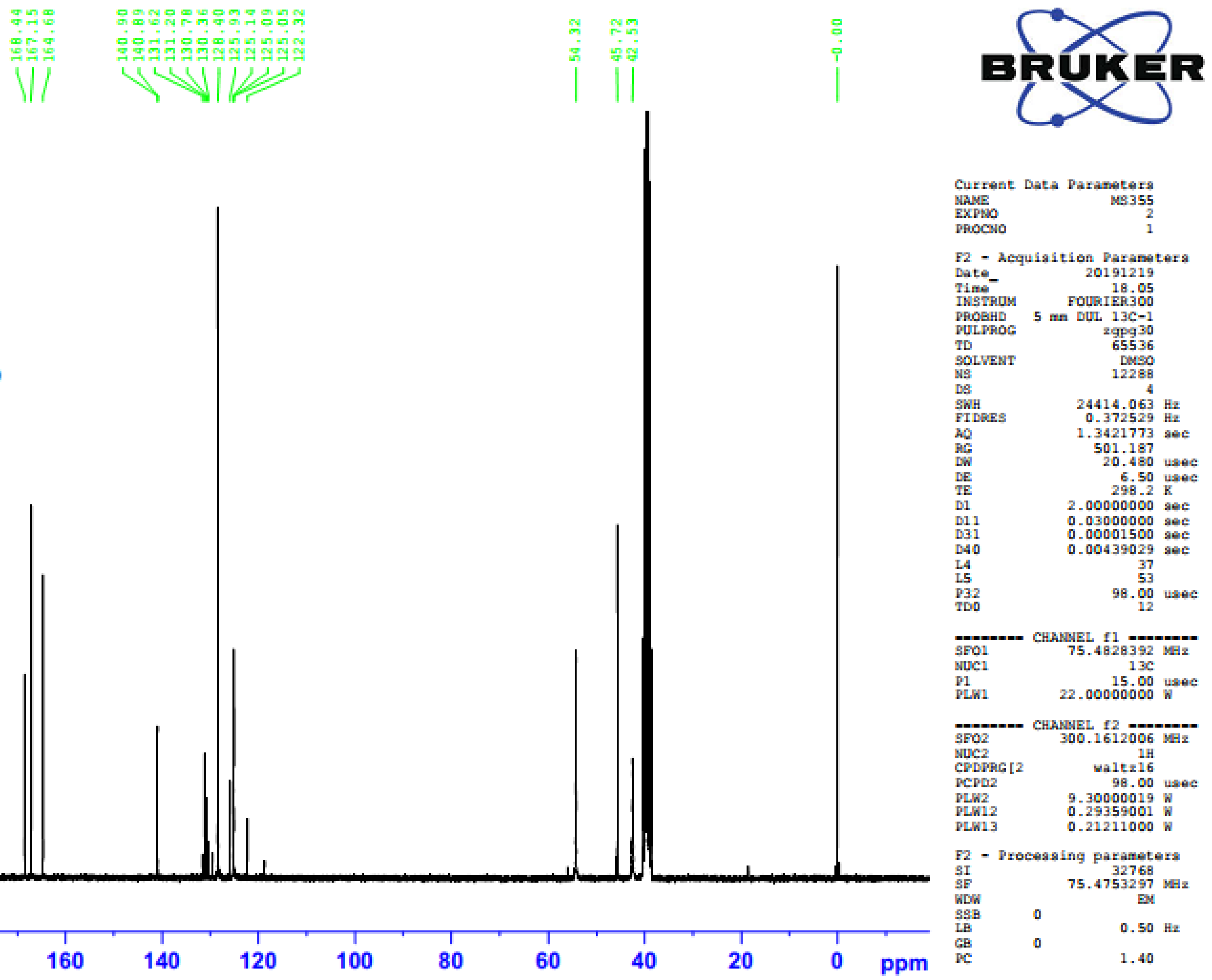

6-[4-(Trifluoromethoxy)phenyl]-4-(4-methylpiperazino)-1,3,5-triazine-2-amine (**9**{*4*,*12*})

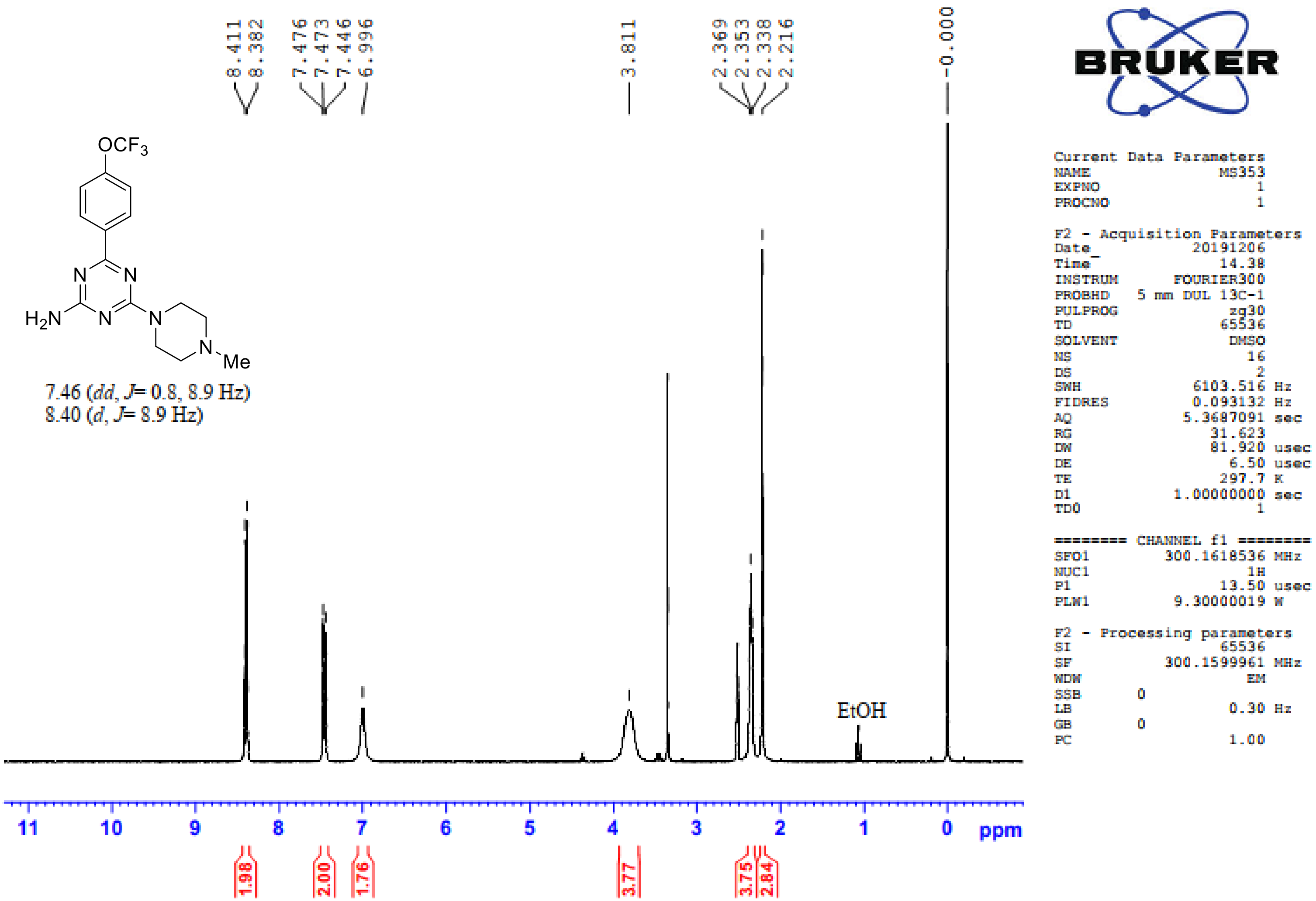

6-[4-(Trifluoromethoxy)phenyl]-4-(4-methylpiperazino)-1,3,5-triazine-2-amine (**9**{*4*,*12*})

119.9 (*q*, *J*= 257.1 Hz)
150.4 (*d*, *J*= 2.0 Hz)

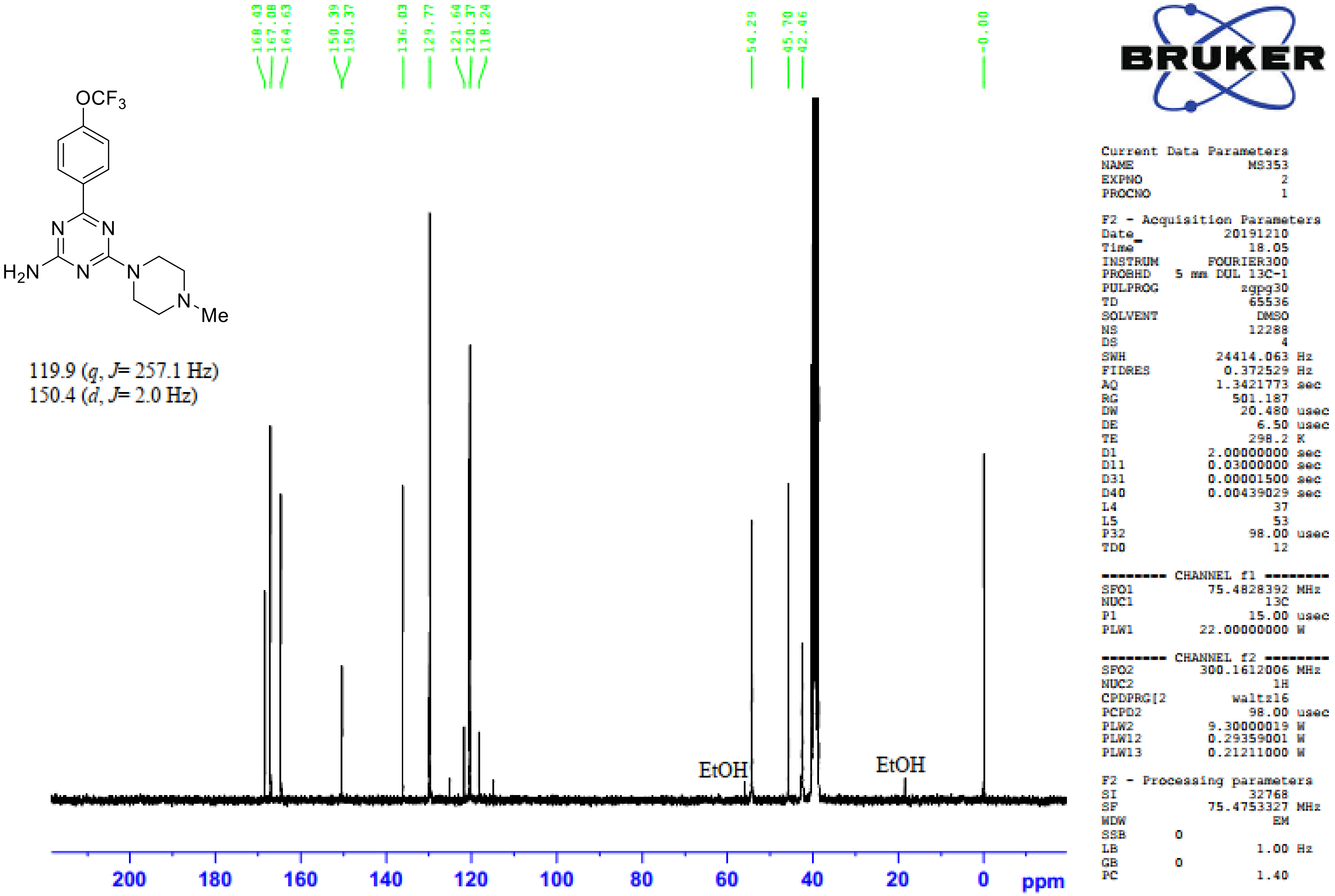

6-[3-(Benzyloxy)phenyl]-4-(4-methylpiperazino)-1,3,5-triazine-2-amine (**9**{*4*,*14*})

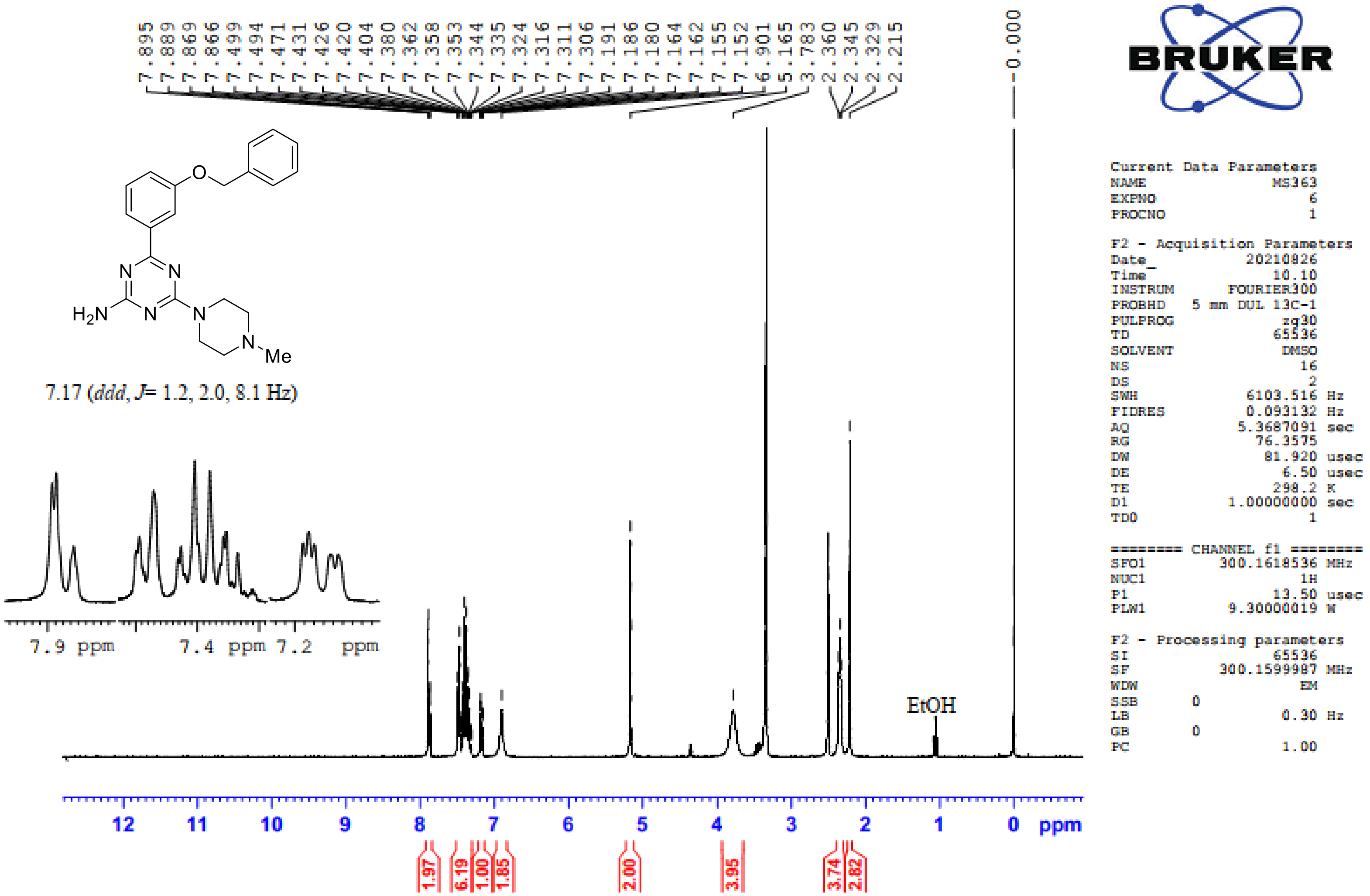

6-[3-(Benzyloxy)phenyl]-4-(4-methylpiperazino)-1,3,5-triazine-2-amine (**9**{*4,14*})

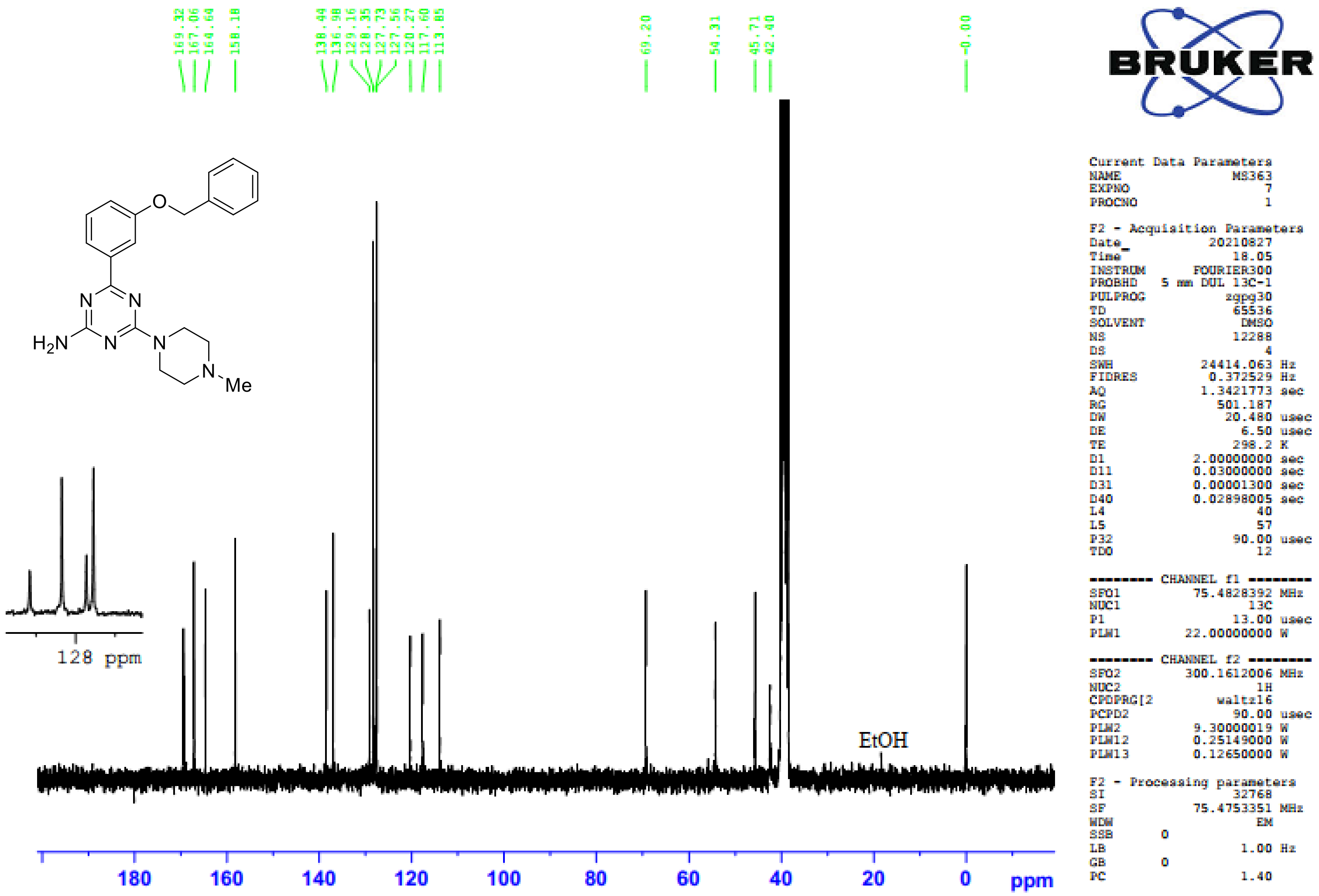

6-[4-(Benzyloxy)phenyl]-4-(4-methylpiperazino)-1,3,5-triazine-2-amine (**9**{*4*,*15*})

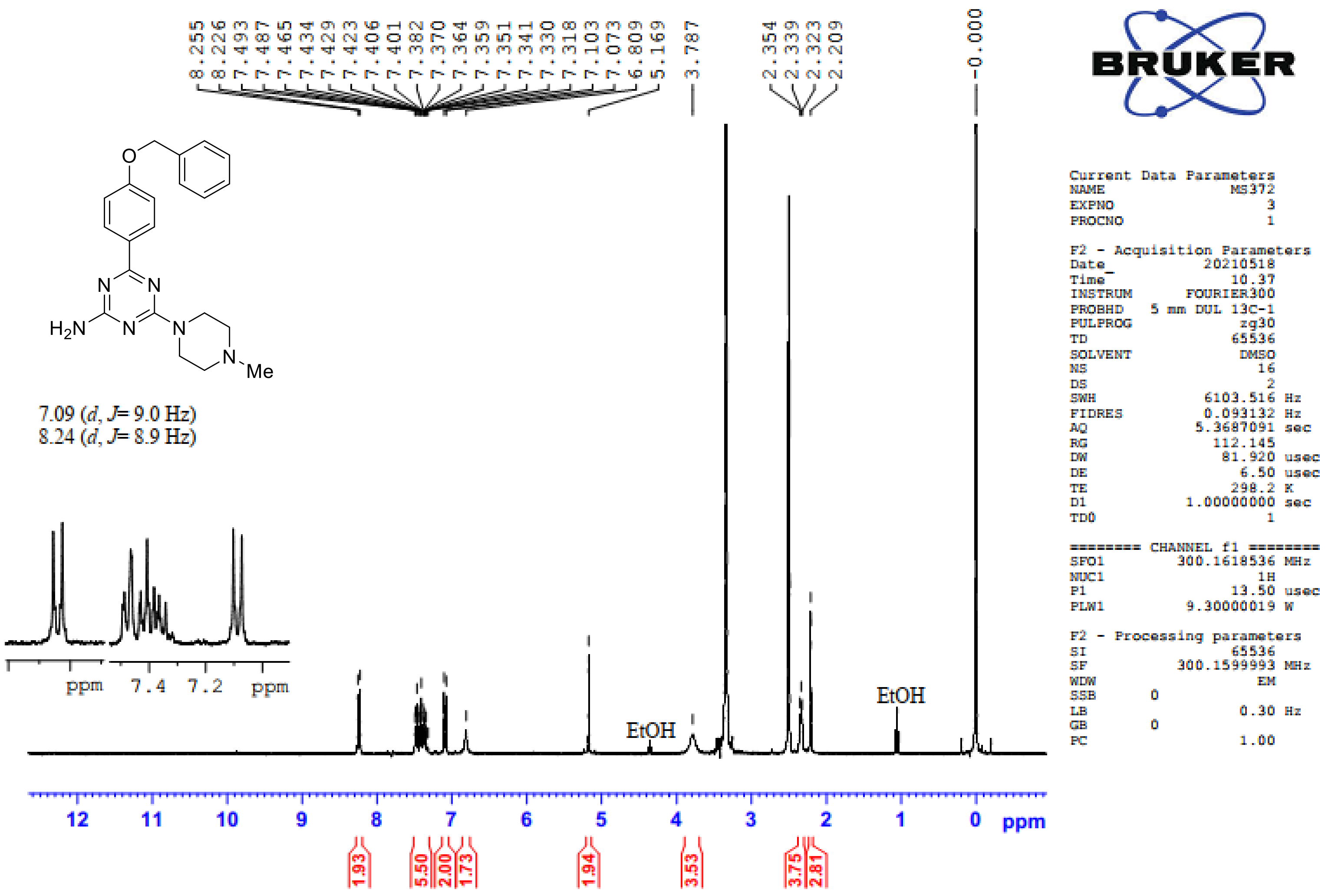

6-[4-(Benzyloxy)phenyl]-4-(4-methylpiperazino)-1,3,5-triazine-2-amine (**9**{*4*,*15*})

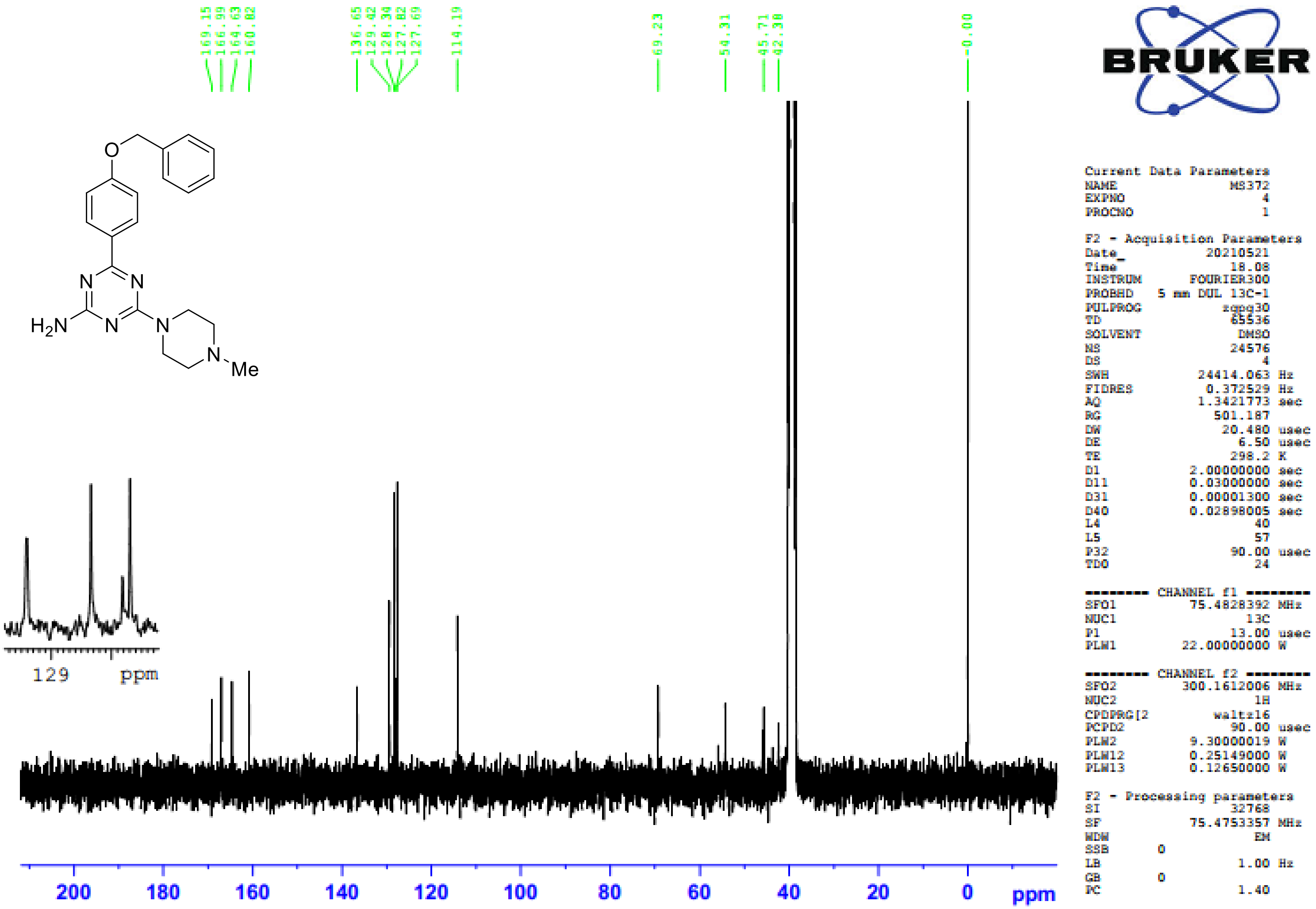

6-(3,4-Dimethoxyphenyl)-4-(4-methylpiperazino)-1,3,5-triazine-2-amine (**9**{*4,16*})

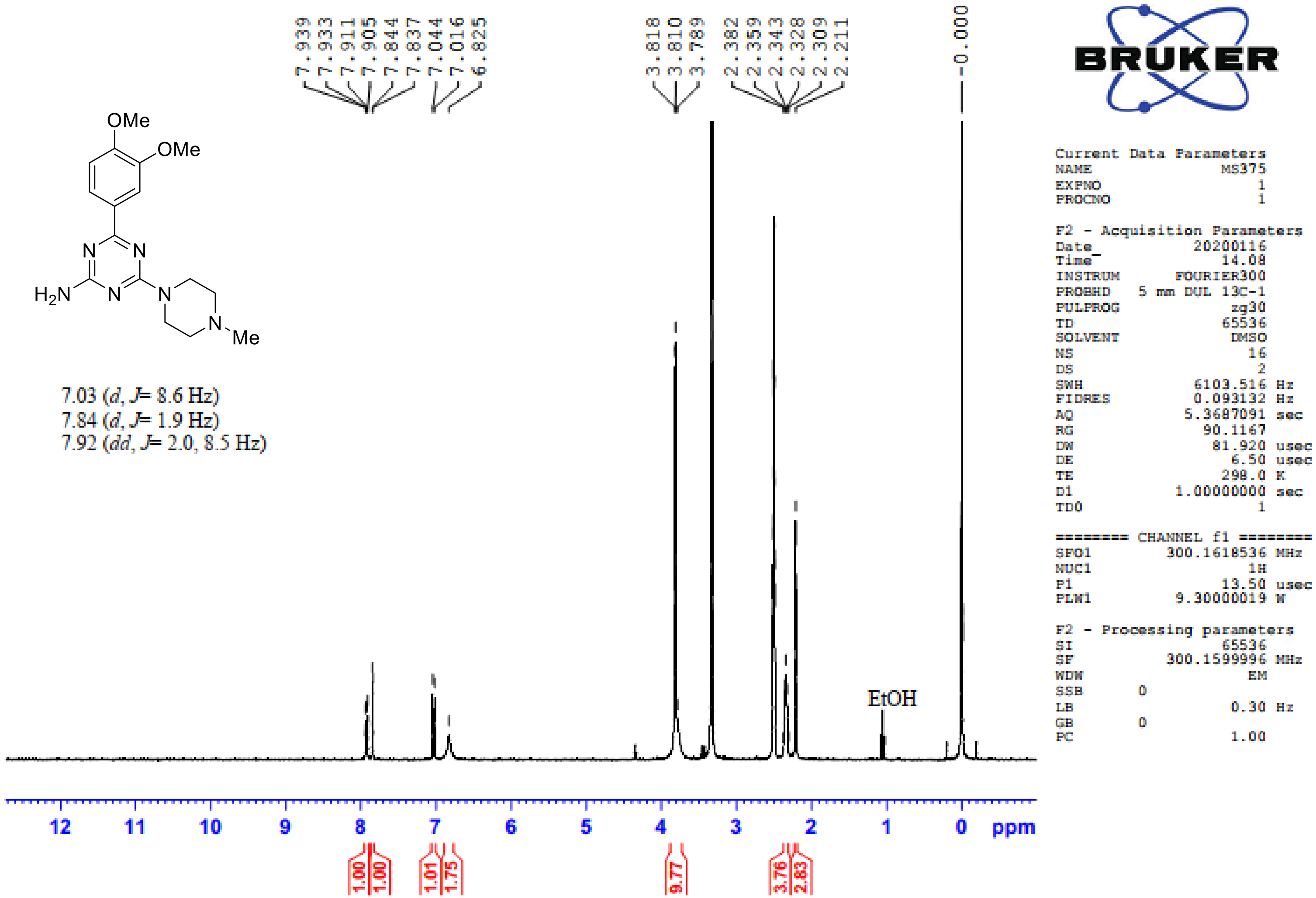

6-(3,4-Dimethoxyphenyl)-4-(4-methylpiperazino)-1,3,5-triazine-2-amine (**9**{*4,16*})

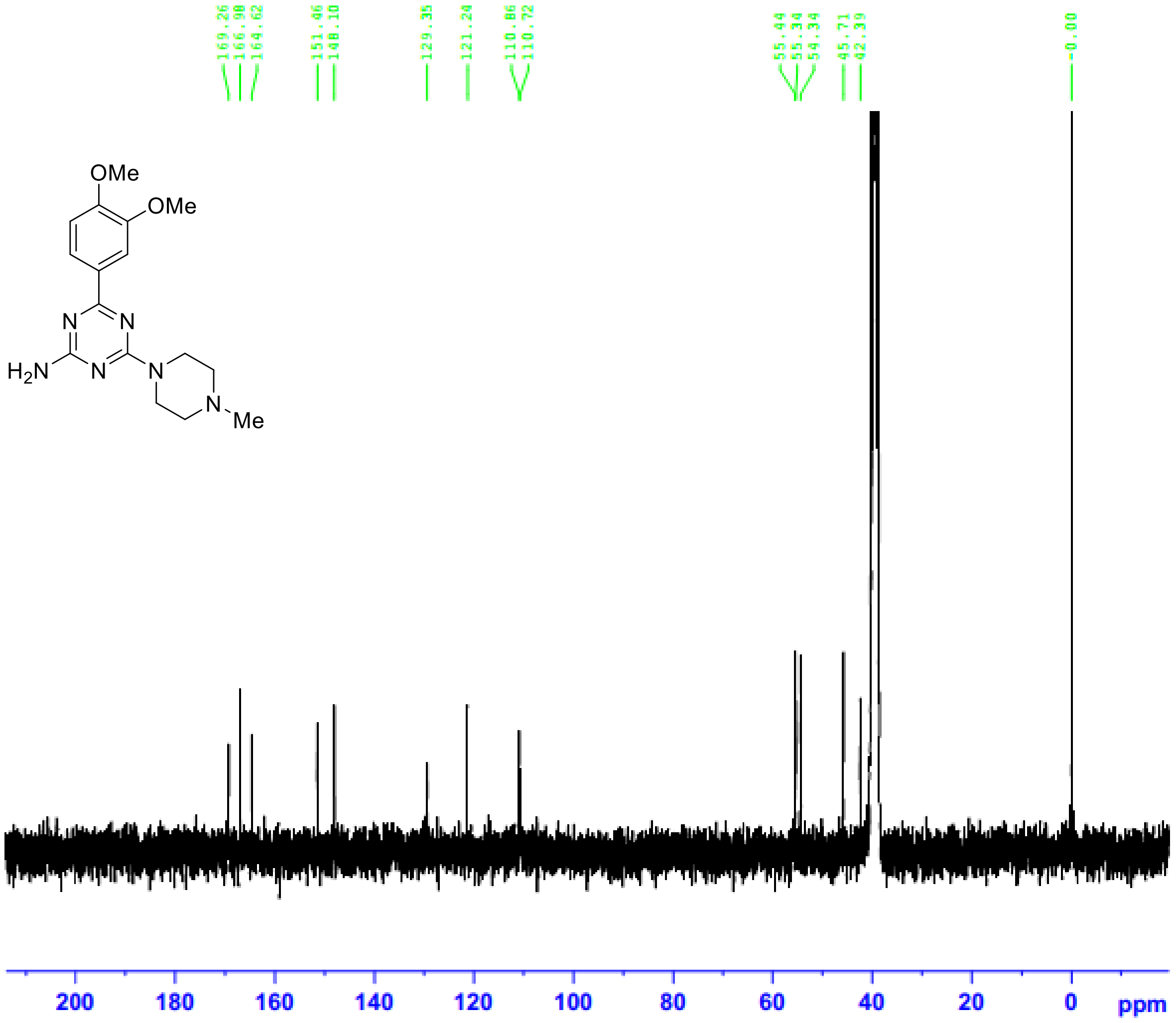

Current Data Parameters
NAME MS375
EXPNO 2
PROCNO 1

F2 - Acquisition Parameters
Date_ 20200117
Time 18.06
INSTRUM FOURIER300
PROBHD 5 mm DUL 13C-1
PULPROG zgpg30
TD 65536
SOLVENT DMSO
NS 12288
DS 4
SWH 24414.063 Hz
FIDRES 0.372529 Hz
AQ 1.3421773 sec
RG 501.187
DW 20.480 usec
DE 6.50 usec
TE 298.1 K
D1 2.00000000 sec
D11 0.03000000 sec
D31 0.00001500 sec
D40 0.00439029 sec
L4 37
L5 53
P32 98.00 usec
TD0 12

======== CHANNEL f1 ========
SFO1 75.4828392 MHz
NUC1 13C
P1 15.00 usec
PLW1 22.00000000 W

======== CHANNEL f2 ========
SFO2 300.1612006 MHz
NUC2 1H
CPDPRG[2 waltz16
PCPD2 98.00 usec
PLW2 9.30000019 W
PLW12 0.29359001 W
PLW13 0.21211000 W

F2 - Processing parameters
SI 32768
SF 75.4753357 MHz
WDW EM
SSB 0
LB 1.00 Hz
GB 0
PC 1.40

6-(3,4,5-Trimethoxyphenyl)-4-(4-methylpiperazino)-1,3,5-triazine-2-amine (**9**{*4*,*17*})

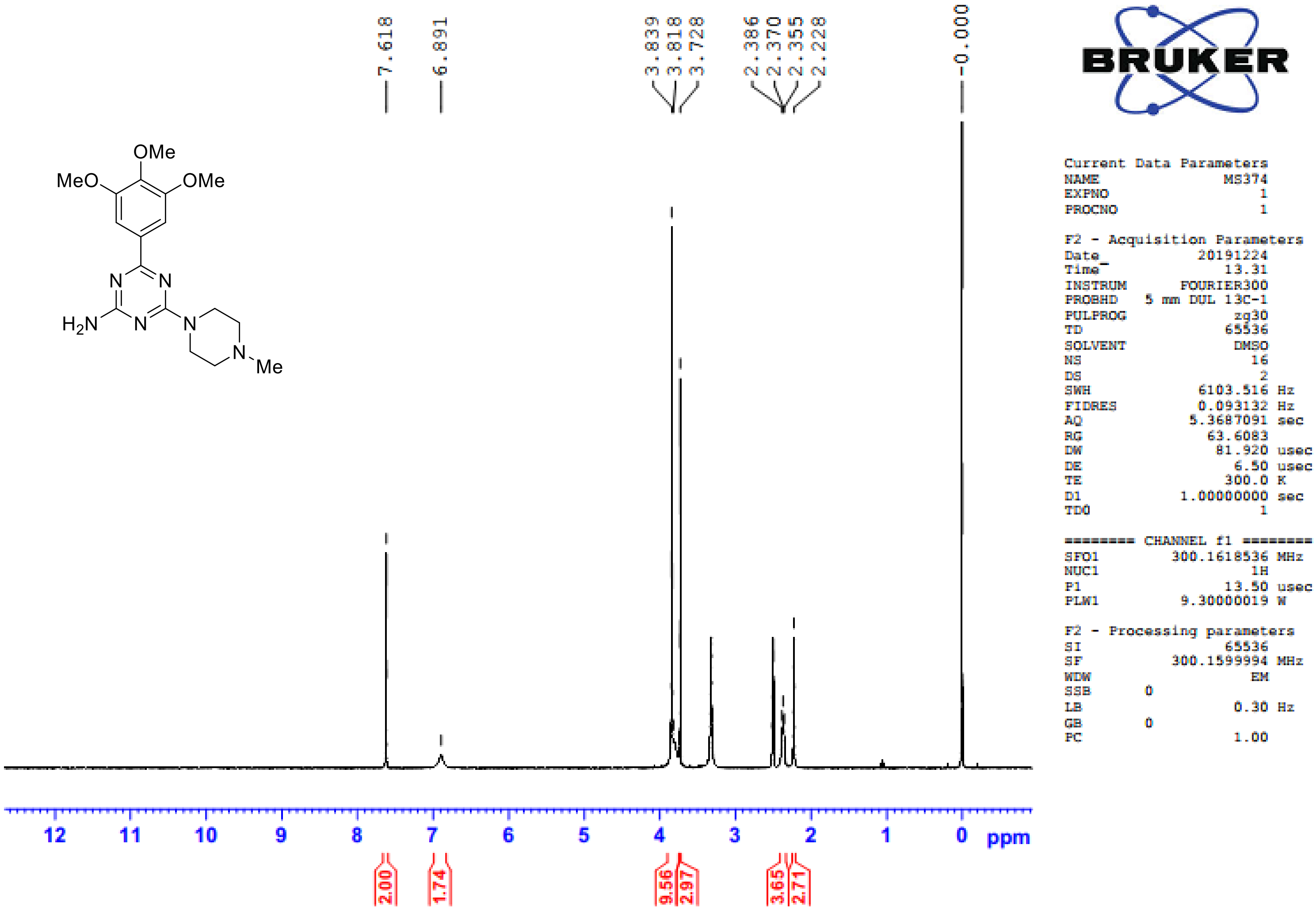

6-(3,4,5-Trimethoxyphenyl)-4-(4-methylpiperazino)-1,3,5-triazine-2-amine (**9**{*4,17*})

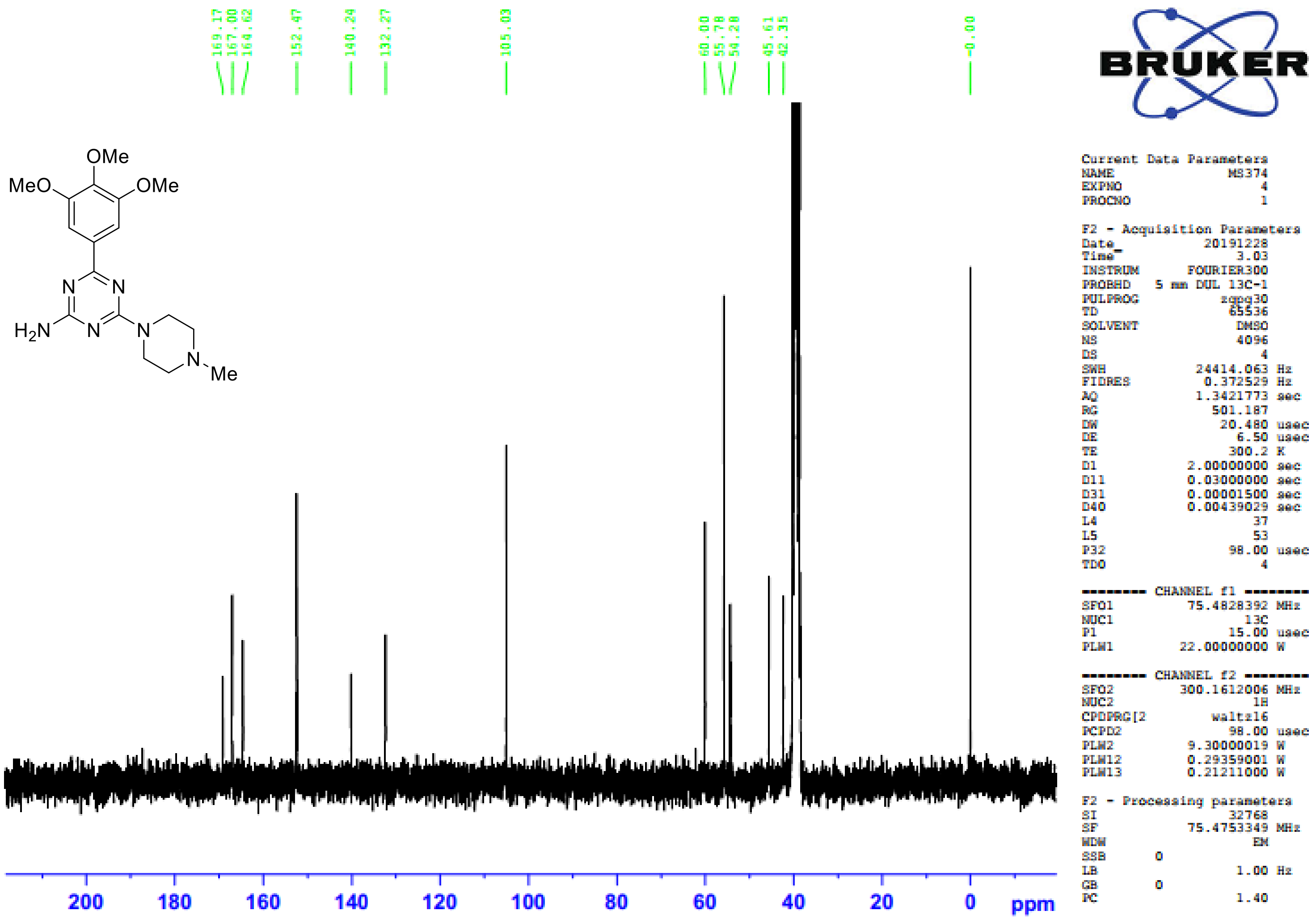

6-(3-Fluorophenyl)-4-(3,4-dihydroisoquinolin-2(1*H*)-yl)-1,3,5-triazine-2-amine (**9**{*6,2*})

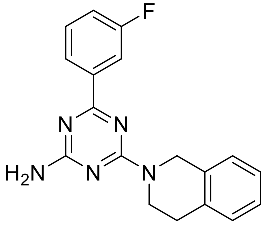

2.89 (*br t*, *J*= 5.5 Hz)
7.38 (*dddd*, *J*= 0.9, 2.7, 8.5, 8.5 Hz)
7.55 (*td*, *J*= 8.0, 6.0 Hz)
8.06 (*d*, *J*= 10.0 Hz)
8.19 (*d*, *J*= 7.8 Hz)

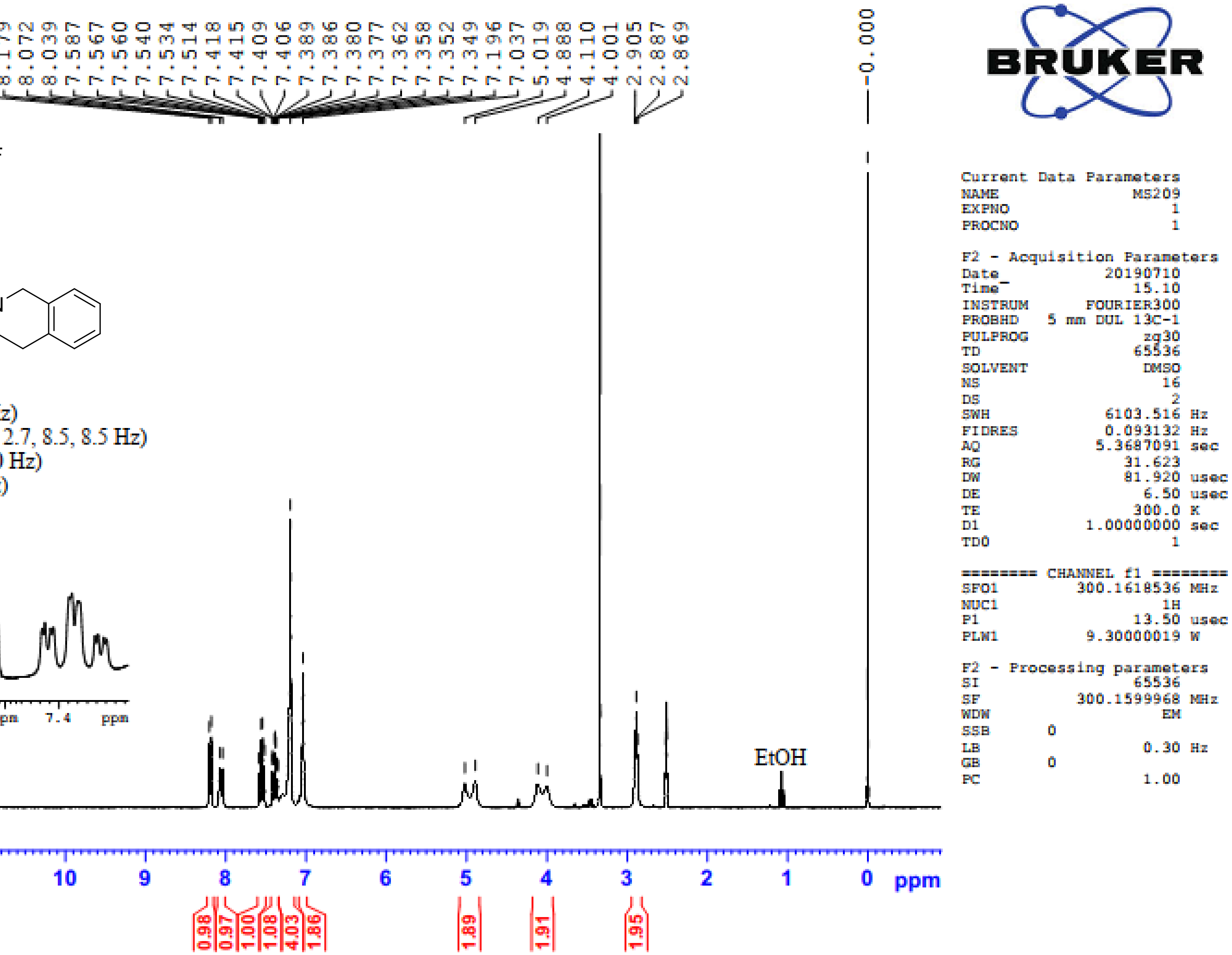

6-(3-Fluorophenyl)-4-(3,4-dihydroisoquinolin-2(1*H*)-yl)-1,3,5-triazine-2-amine (**9**{*6*,*2*})

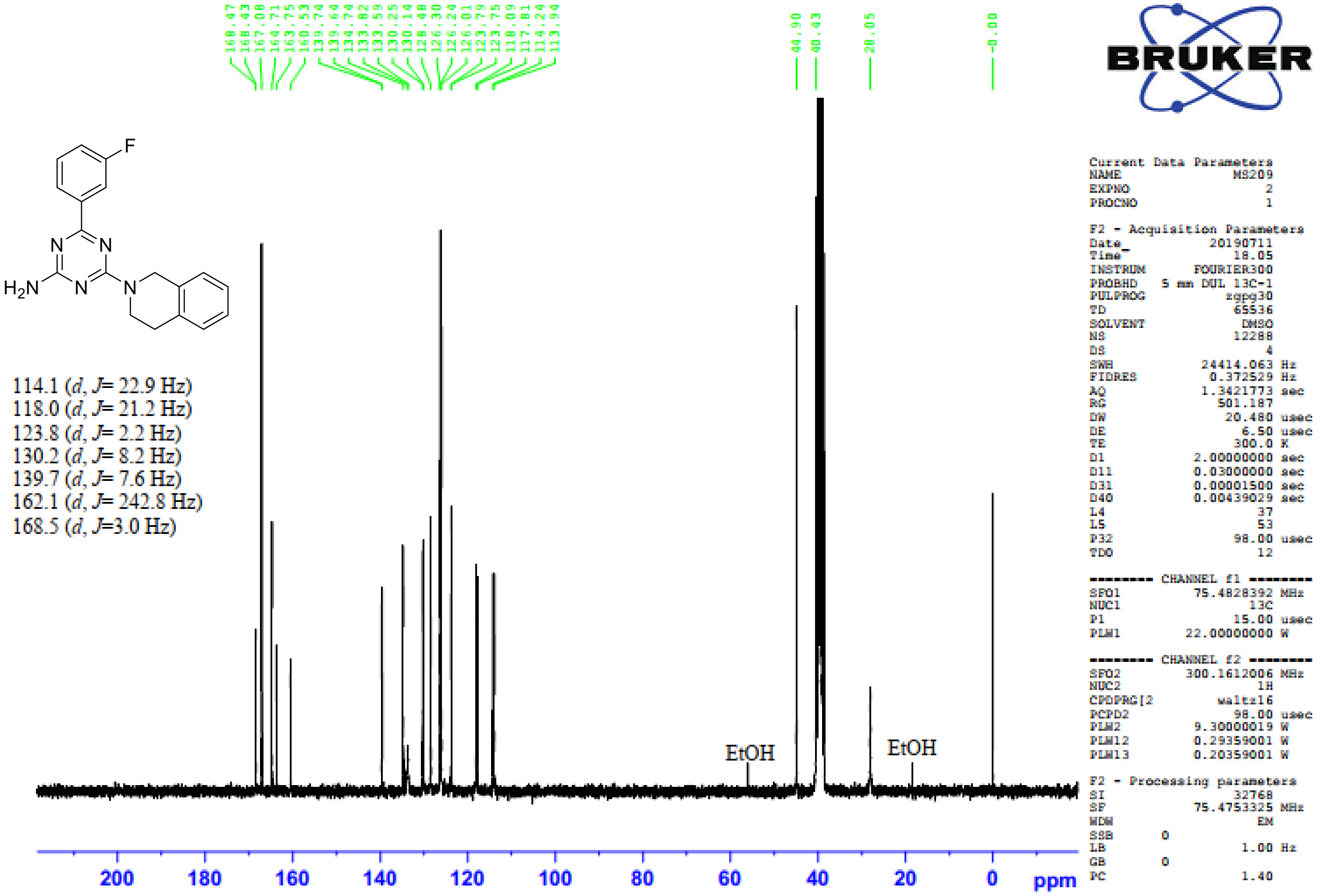

114.1 (*d*, *J*= 22.9 Hz)
118.0 (*d*, *J*= 21.2 Hz)
123.8 (*d*, *J*= 2.2 Hz)
130.2 (*d*, *J*= 8.2 Hz)
139.7 (*d*, *J*= 7.6 Hz)
162.1 (*d*, *J*= 242.8 Hz)
168.5 (*d*, *J*=3.0 Hz)

6-(4-Chlorophenyl)-4-(3,4-dihydroisoquinolin-2(1*H*)-yl)-1,3,5-triazine-2-amine (**9**{*6*,*4*})

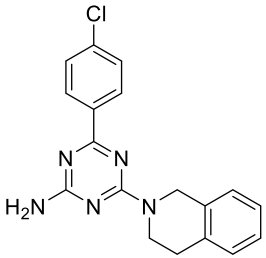

2.88 (*br t*, *J*= 5.3 Hz)
7.56 (*d*, *J*= 8.6 Hz)
8.34 (*d*, *J*= 8.6 Hz)

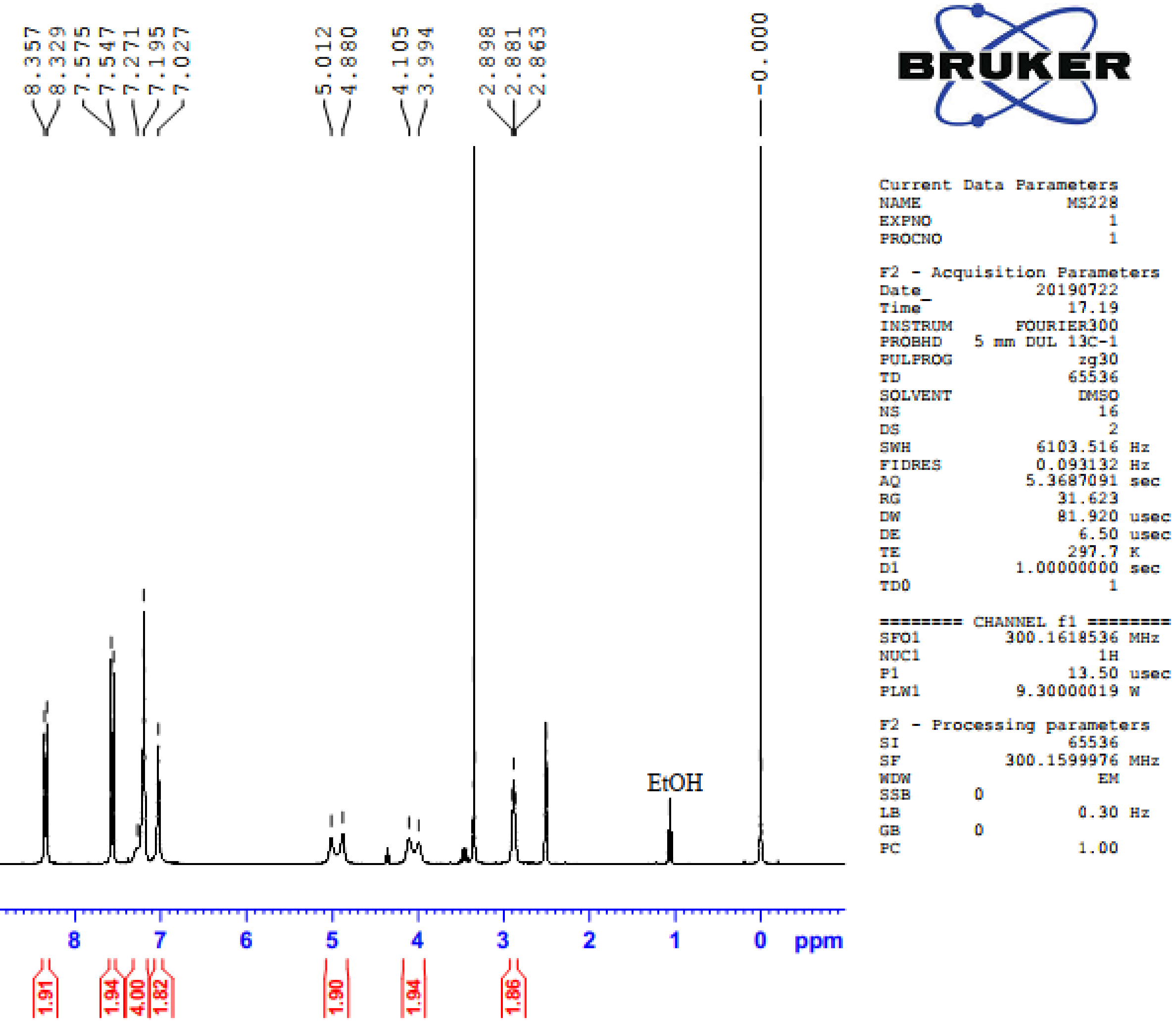

6-(4-Chlorophenyl)-4-(3,4-dihydroisoquinolin-2(1*H*)-yl)-1,3,5-triazine-2-amine (**9**{*6,4*})

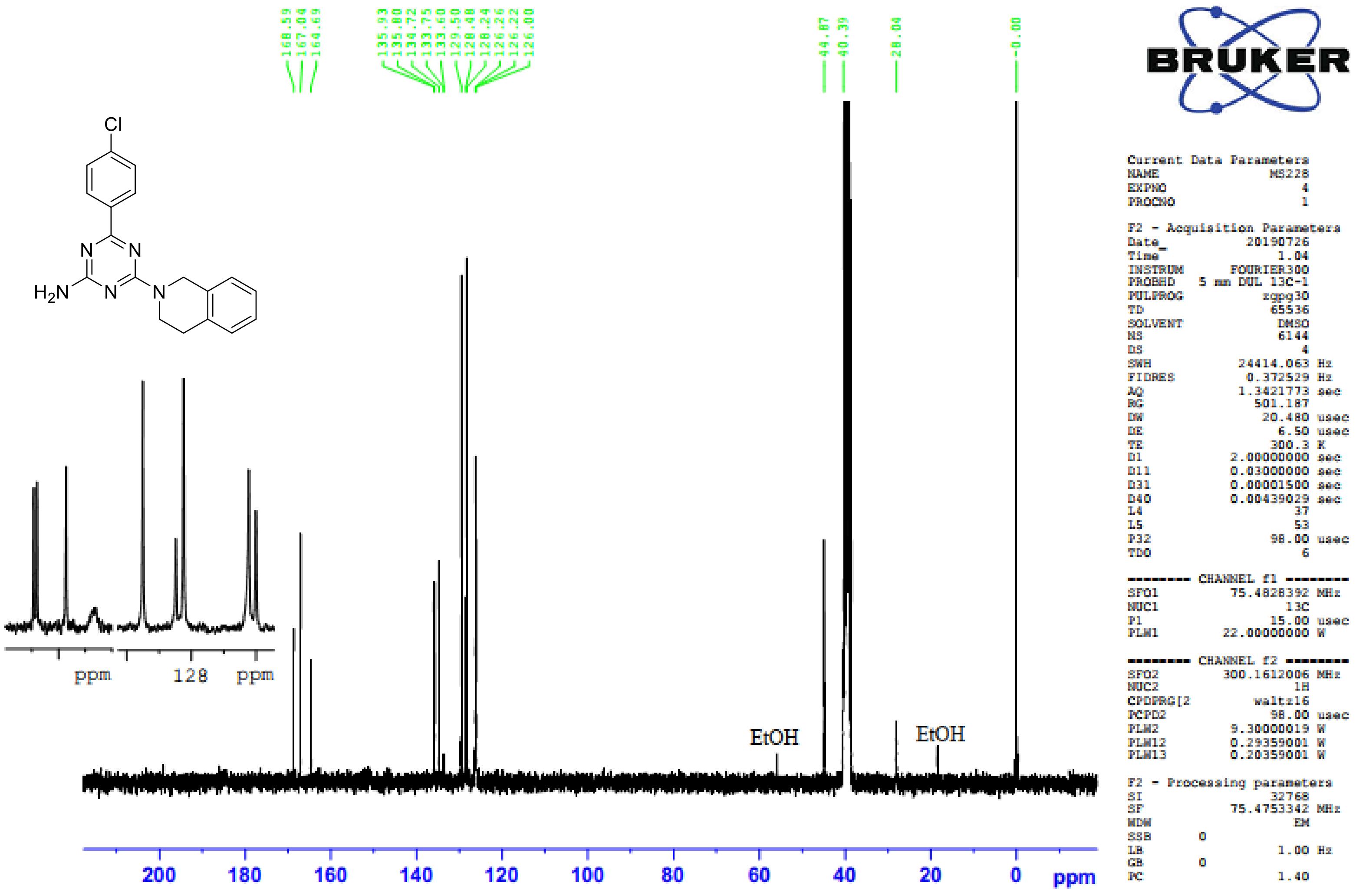

6-(3-Methylphenyl)-4-(3,4-dihydroisoquinolin-2(1*H*)-yl)-1,3,5-triazine-2-amine (**9**{*6*,*5*})

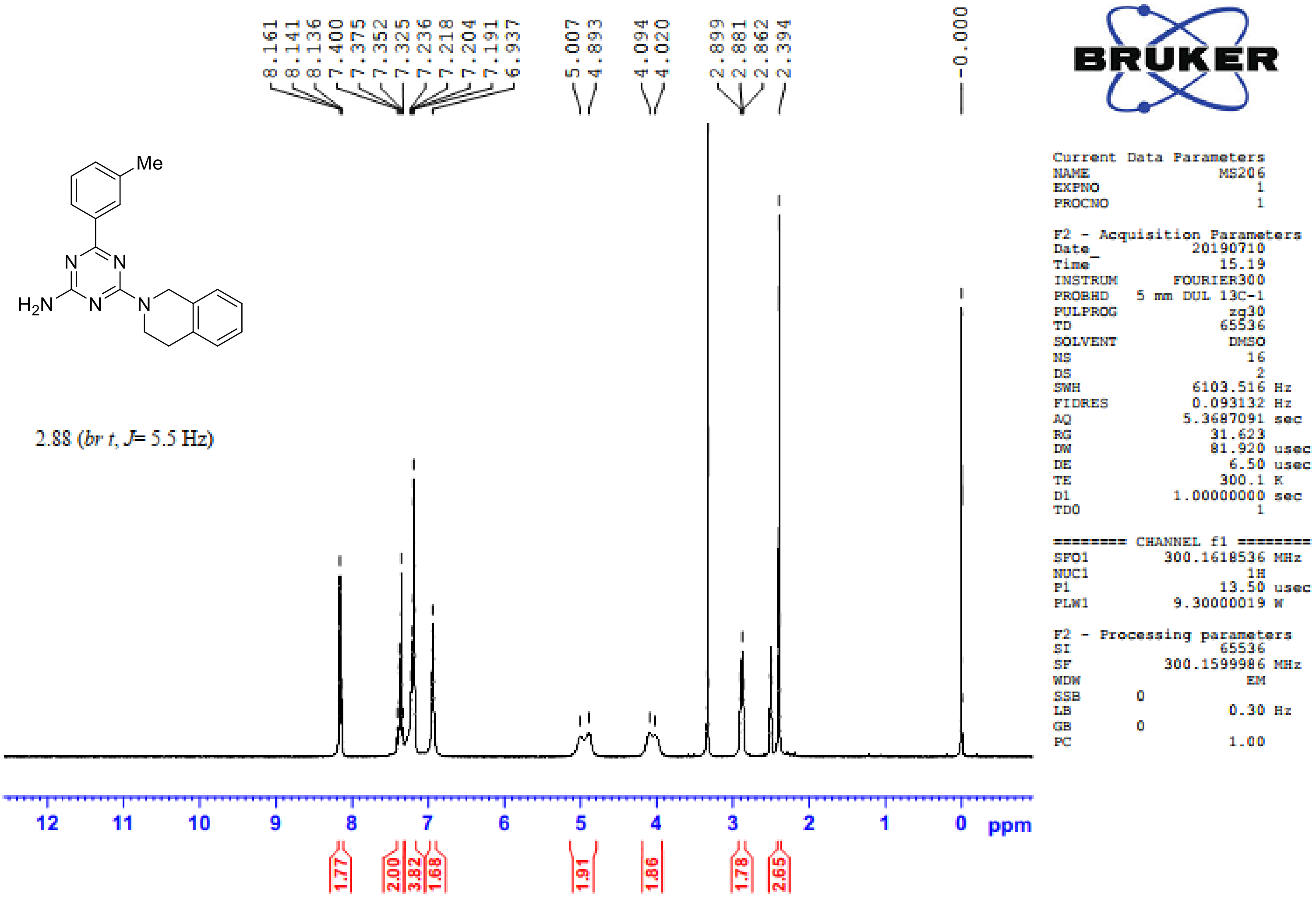

6-(3-Methylphenyl)-4-(3,4-dihydroisoquinolin-2(1*H*)-yl)-1,3,5-triazine-2-amine (**9**{*6*,*5*})

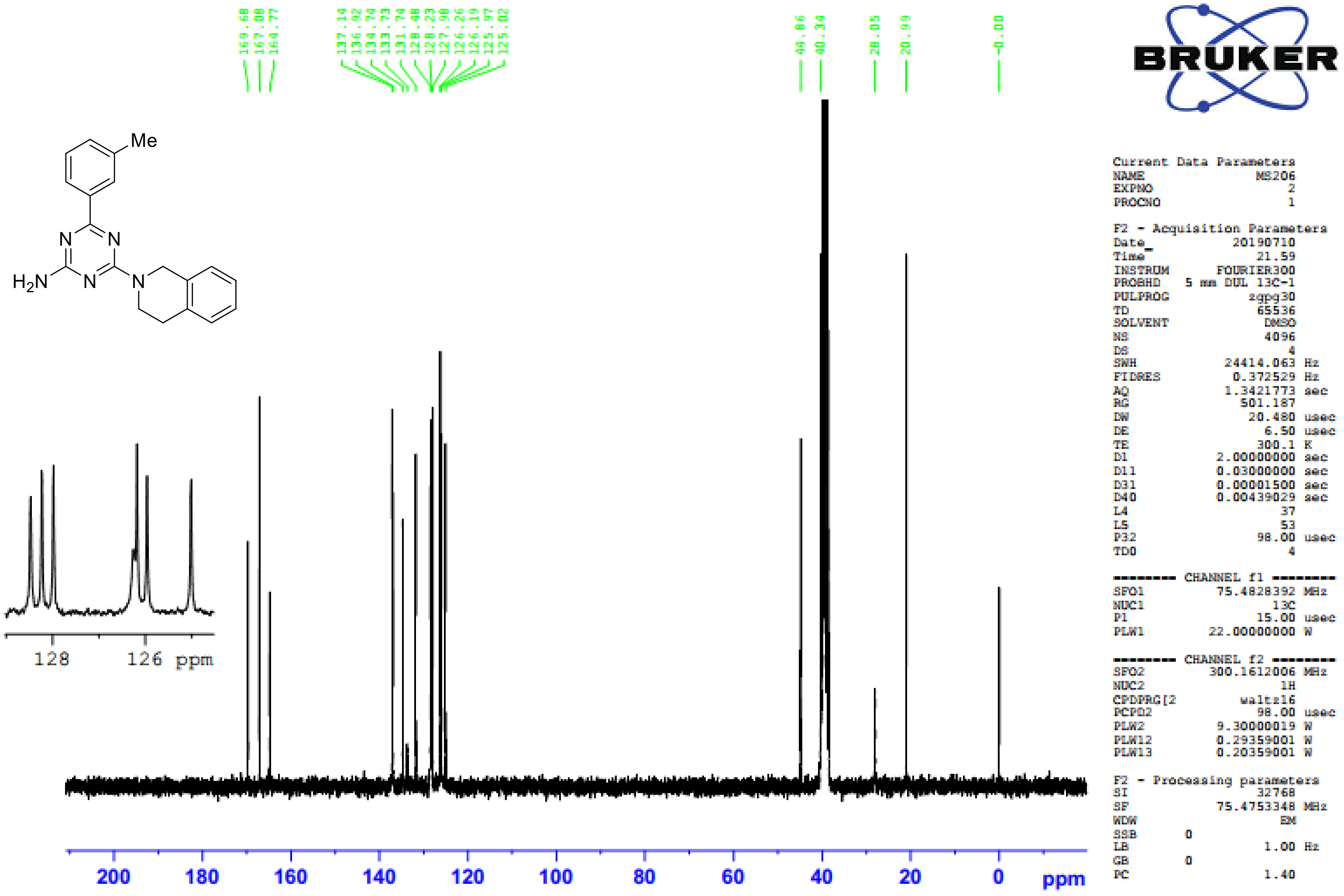

6-(4-Methylphenyl)-4-(3,4-dihydroisoquinolin-2(1*H*)-yl)-1,3,5-triazine-2-amine (**9**{*6*,*6*})

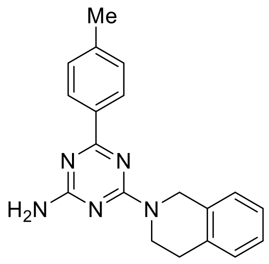

2.87 (*br t*, *J*= 5.7 Hz)
7.29 (*d*, *J*= 8.0 Hz)
8.26 (*d*, *J*= 8.1 Hz)

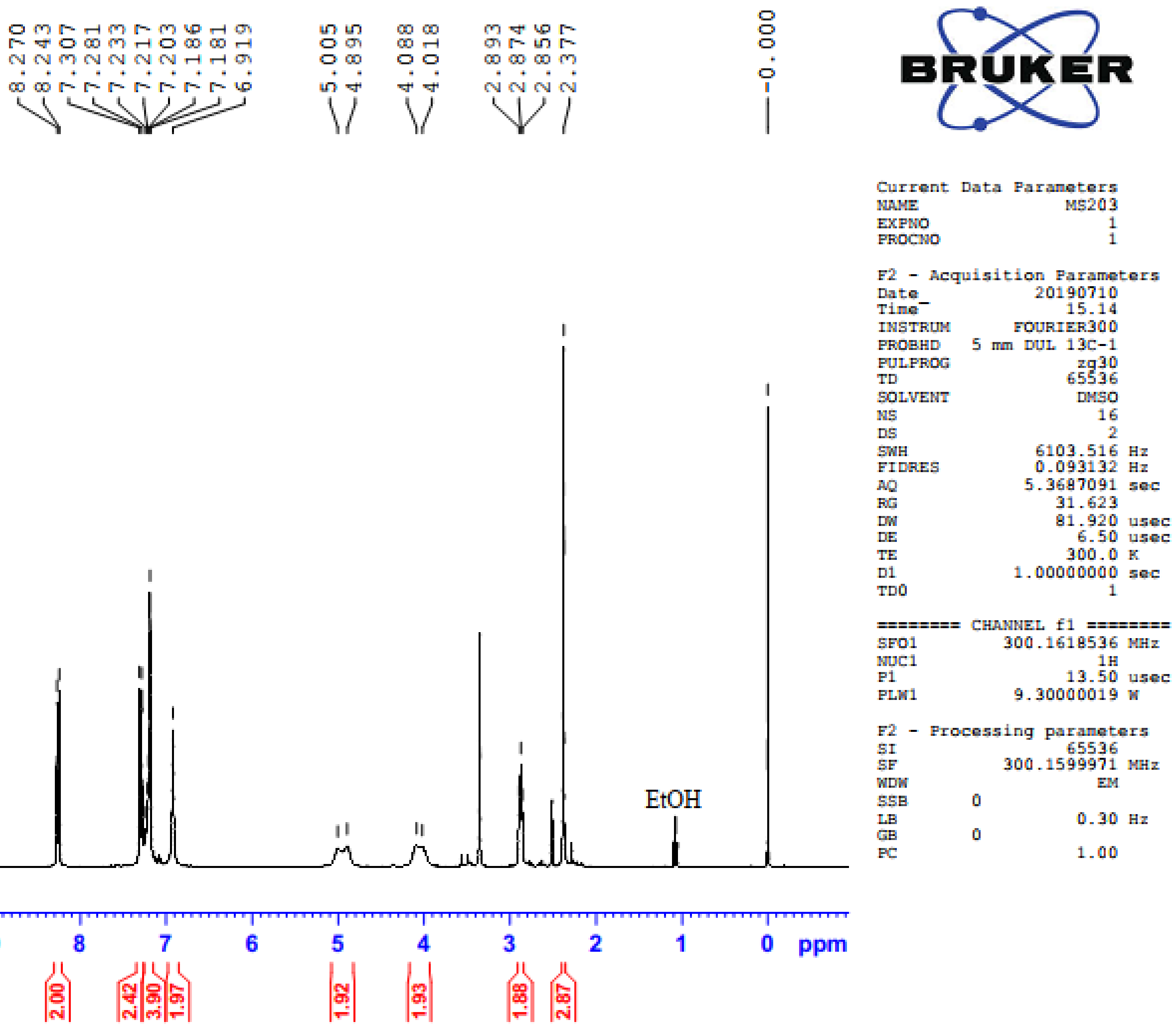

6-(4-Methylphenyl)-4-(3,4-dihydroisoquinolin-2(1*H*)-yl)-1,3,5-triazine-2-amine (**9**{*6,6*})

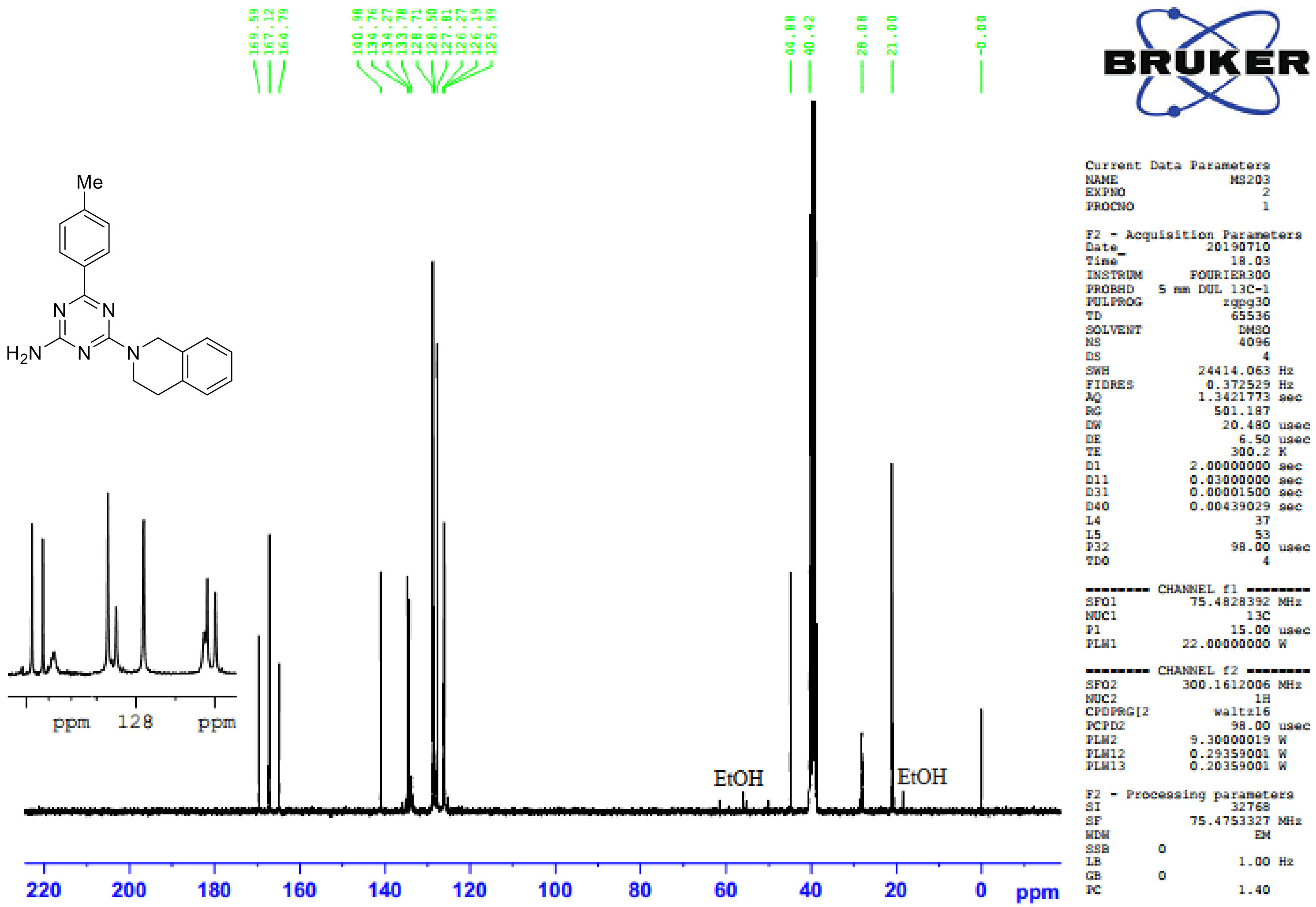

6-[4-(*tert*-Butyl)phenyl]-4-(3,4-dihydroisoquinolin-2(1*H*)-yl)-1,3,5-triazine-2-amine (**9**{*6,7*})

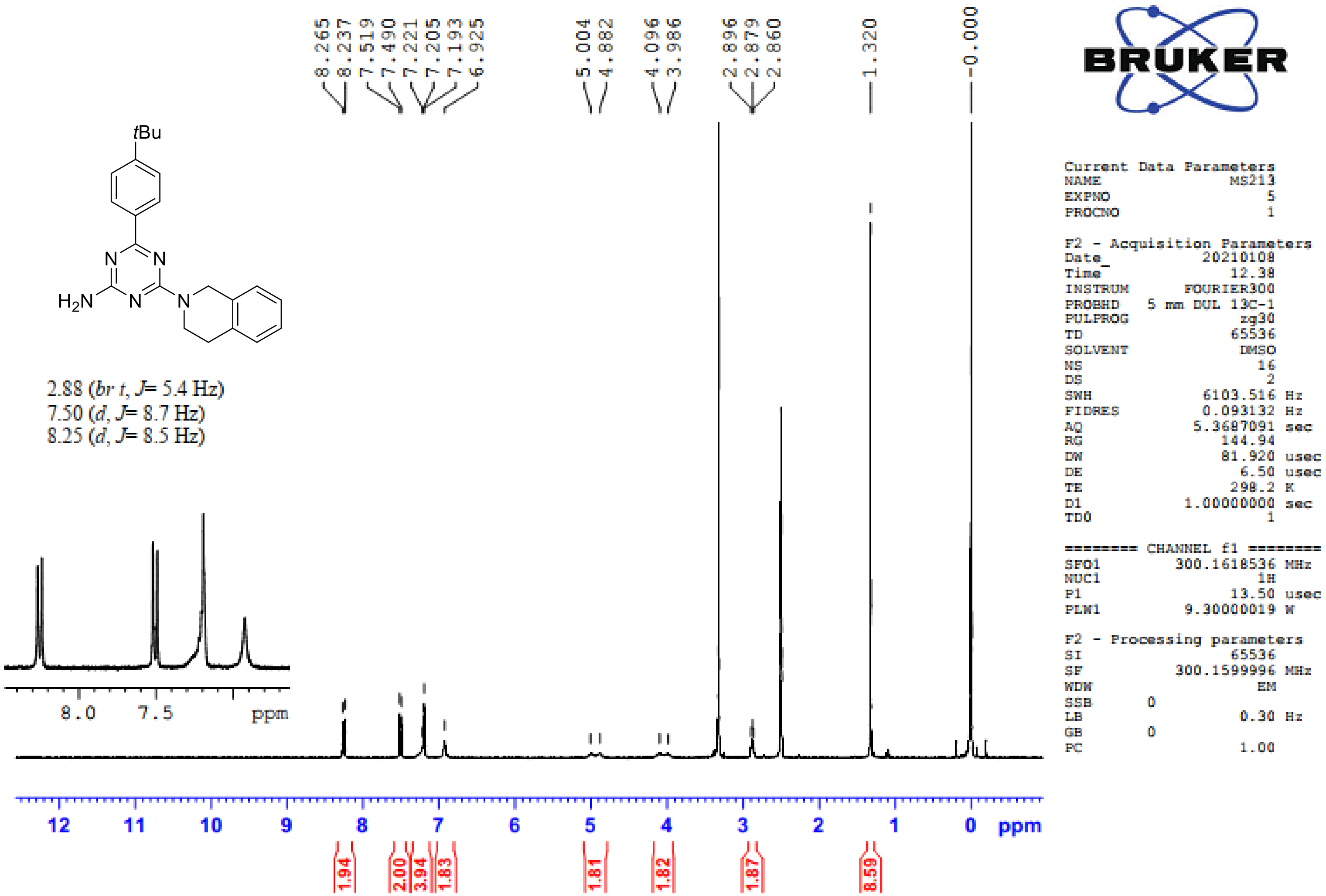

6-[4-(*tert*-Butyl)phenyl]-4-(3,4-dihydroisoquinolin-2(1*H*)-yl)-1,3,5-triazine-2-amine (**9**{*6*,*7*})

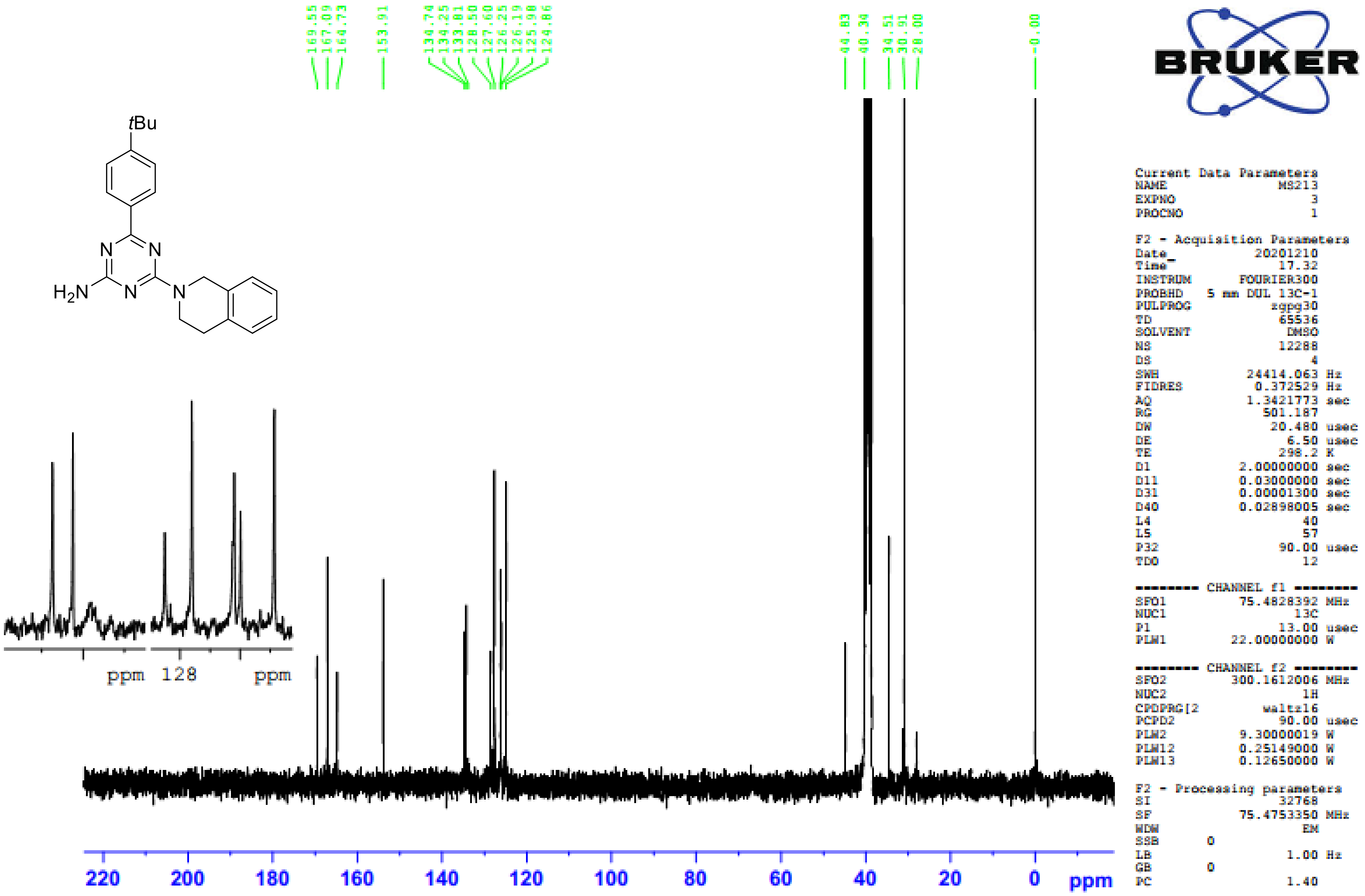

6-[4-(*N*,*N*-Dimethylamino)phenyl]-4-(3,4-dihydroisoquinolin-2(1*H*)-yl)-1,3,5-triazine-2-amine (**9**{*6*,*8*})

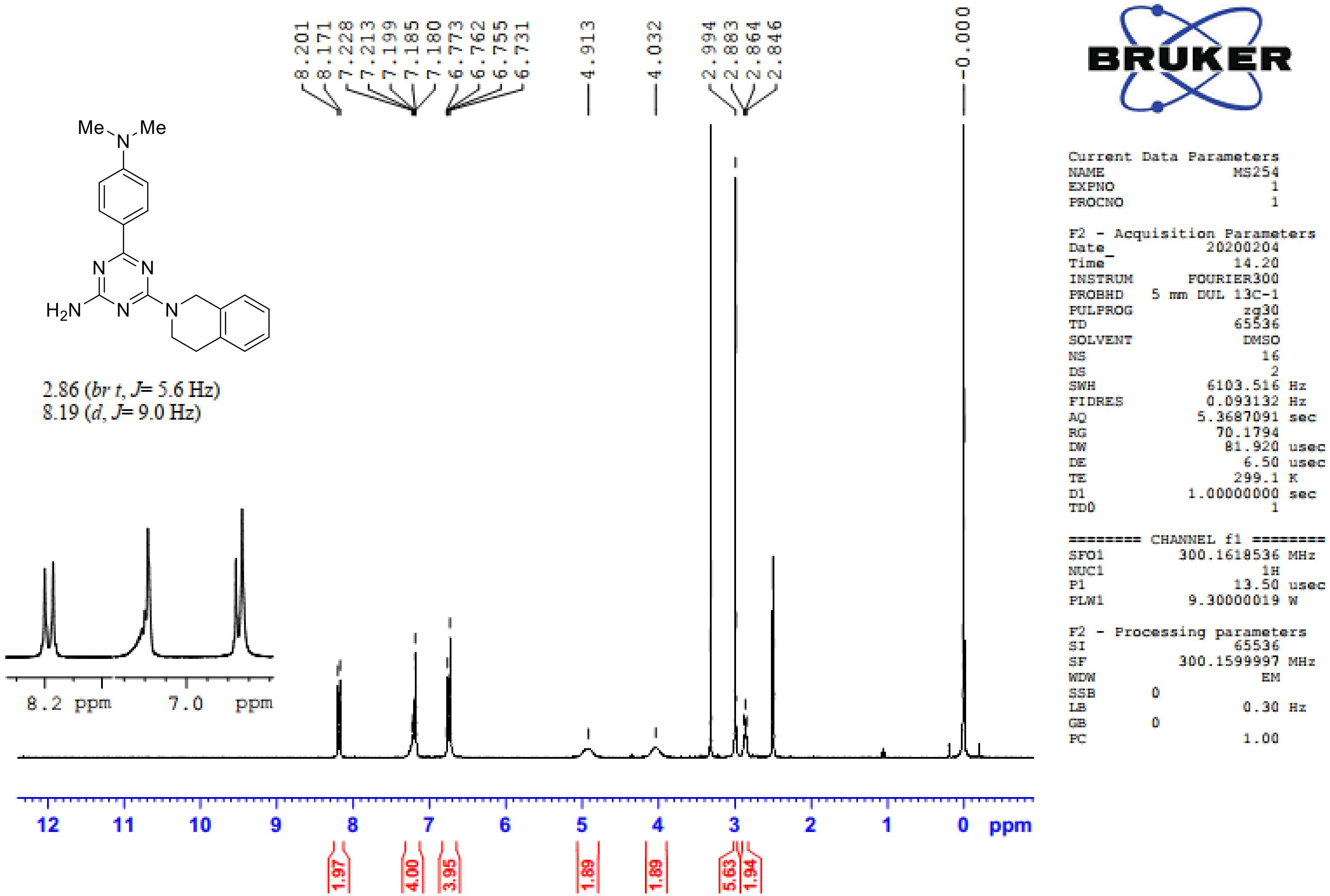

6-[4-(*N*,*N*-Dimethylamino)phenyl]-4-(3,4-dihydroisoquinolin-2(1*H*)-yl)-1,3,5-triazine-2-amine (**9**{*6*,*8*})

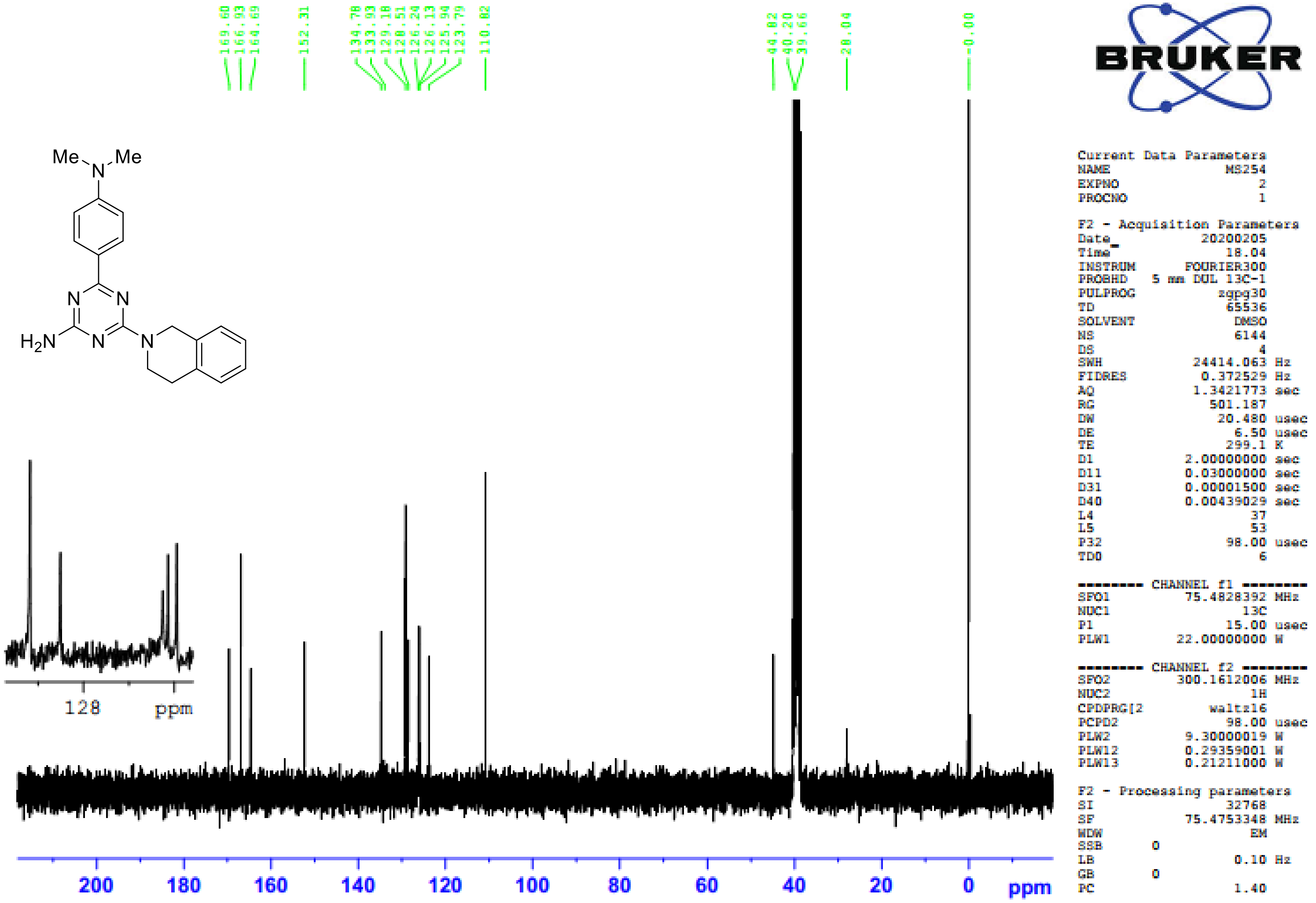

6-(4-Methoxyphenyl)-4-(3,4-dihydroisoquinolin-2(1*H*)-yl)-1,3,5-triazine-2-amine (**9**{*6*,*9*})

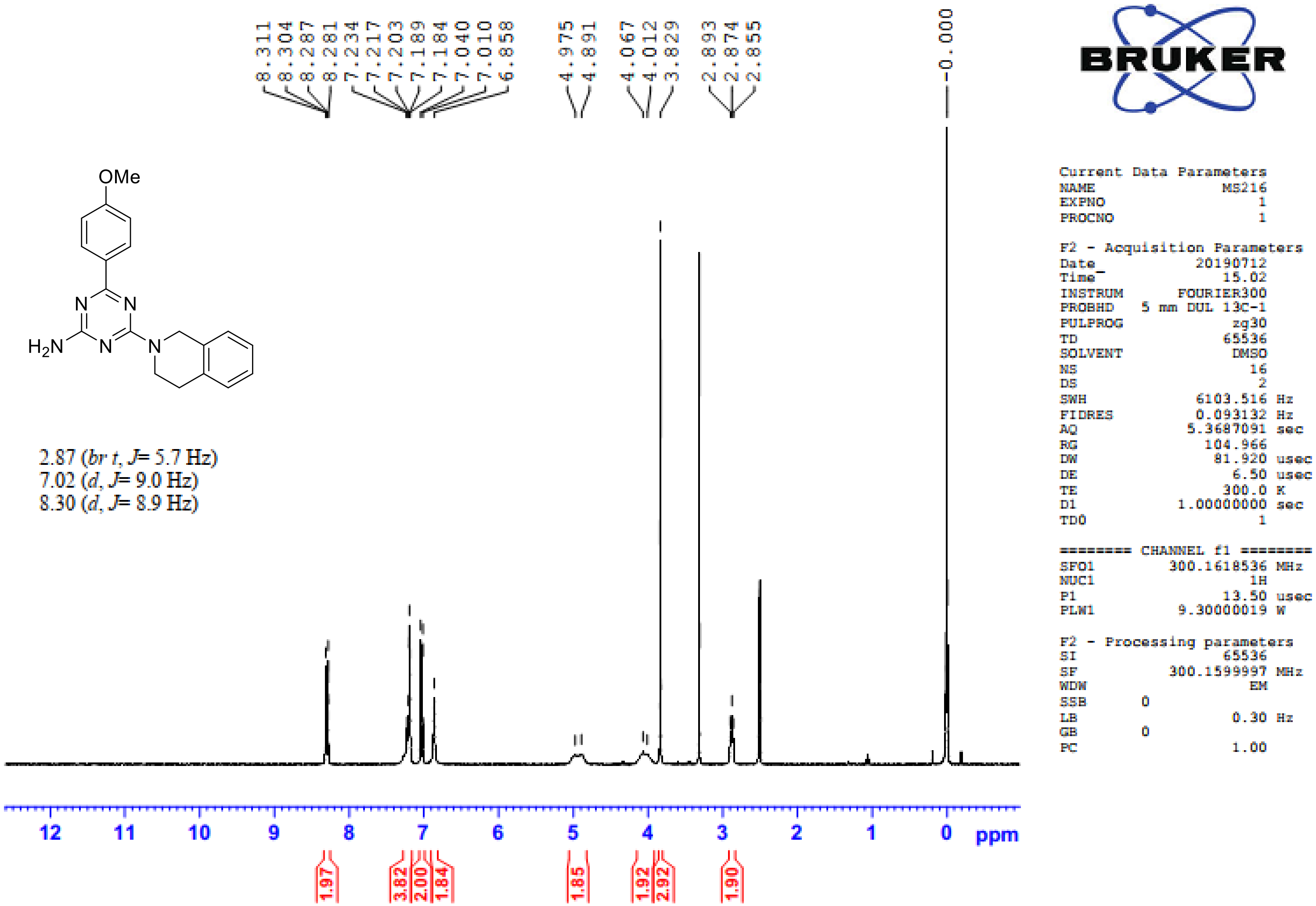

2.87 (*br t*, *J*= 5.7 Hz)
7.02 (*d*, *J*= 9.0 Hz)
8.30 (*d*, *J*= 8.9 Hz)

6-(4-Methoxyphenyl)-4-(3,4-dihydroisoquinolin-2(1*H*)-yl)-1,3,5-triazine-2-amine (**9**{*6,9*})

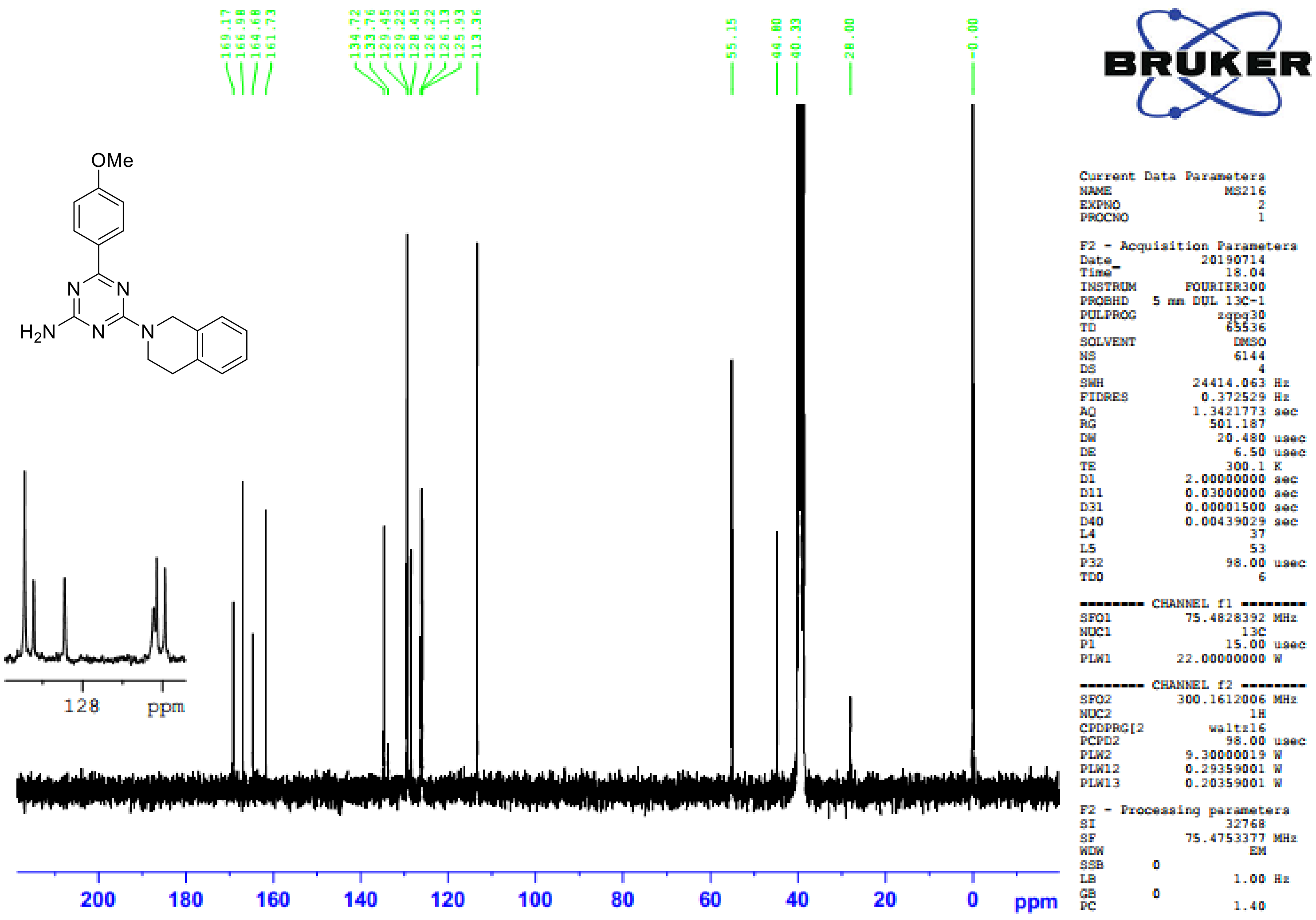

6-[4-(Trifluoromethyl)phenyl]-4-(3,4-dihydroisoquinolin-2(1*H*)-yl)-1,3,5-triazine-2-amine (**9**{*6,11*})

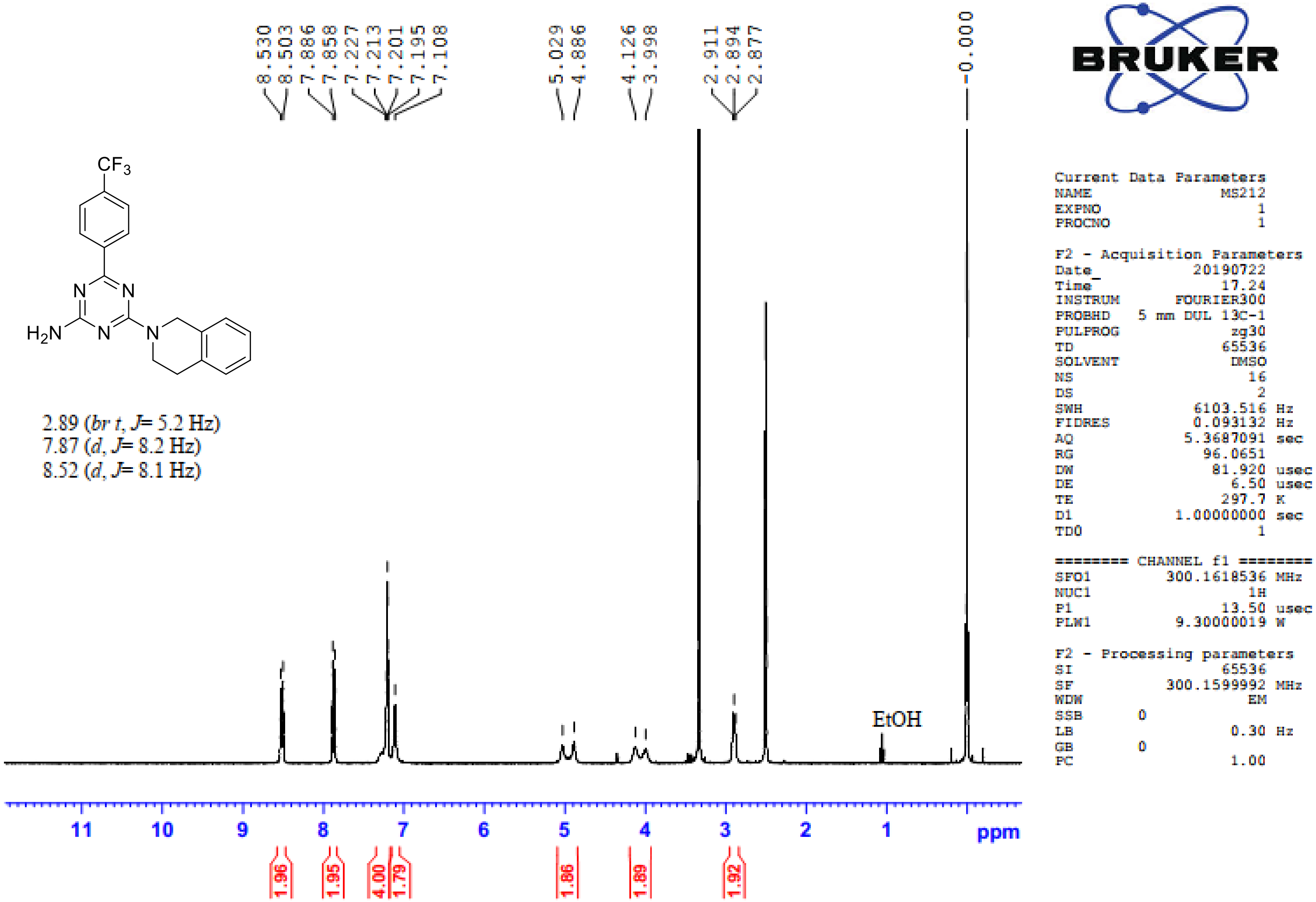

6-[4-(Trifluoromethyl)phenyl]-4-(3,4-dihydroisoquinolin-2(1*H*)-yl)-1,3,5-triazine-2-amine (**9**{*6,11*})

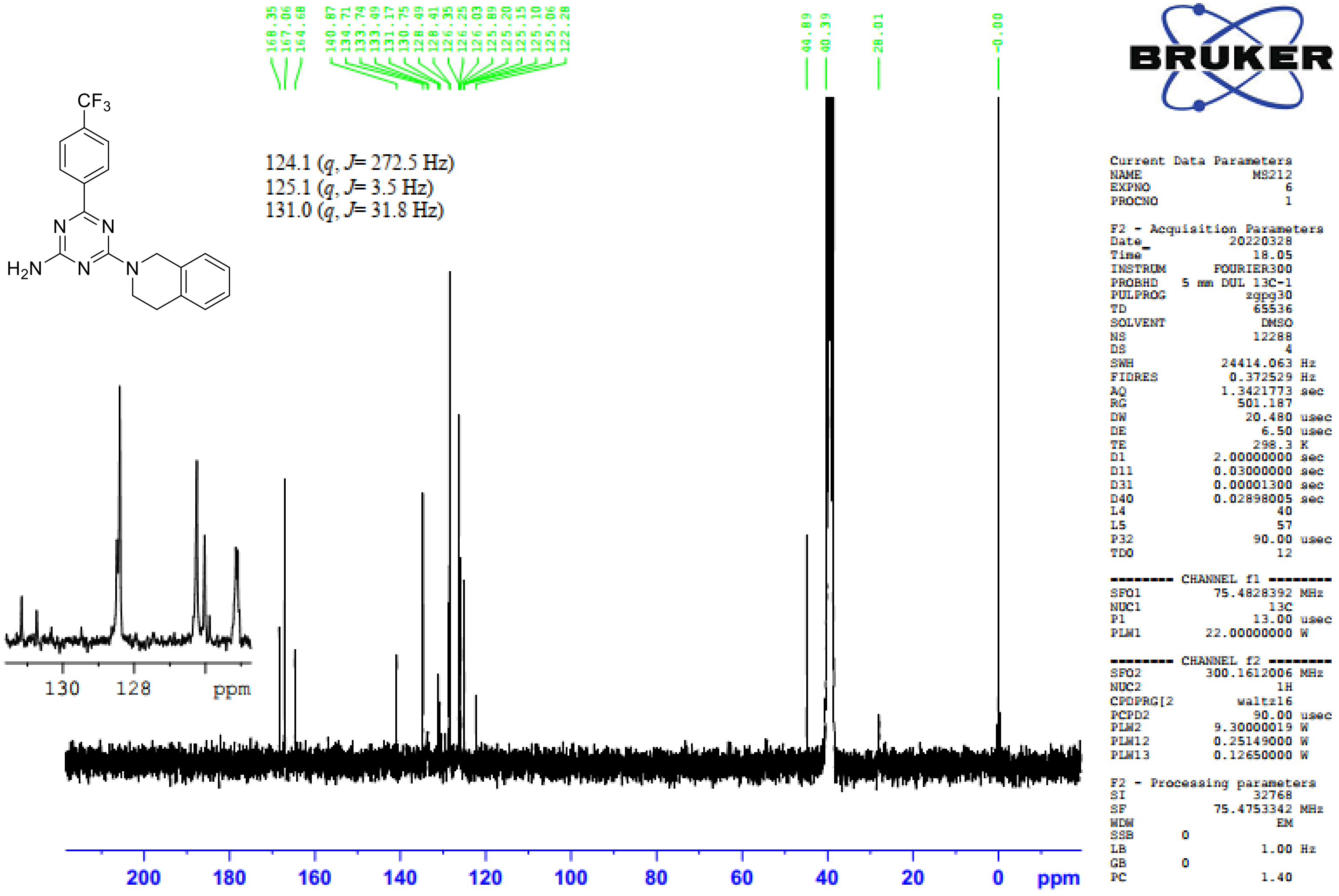

6-[4-(Trifluoromethoxy)phenyl]-4-(3,4-dihydroisoquinolin-2(1*H*)-yl)-1,3,5-triazine-2-amine (**9**{*6,12*})

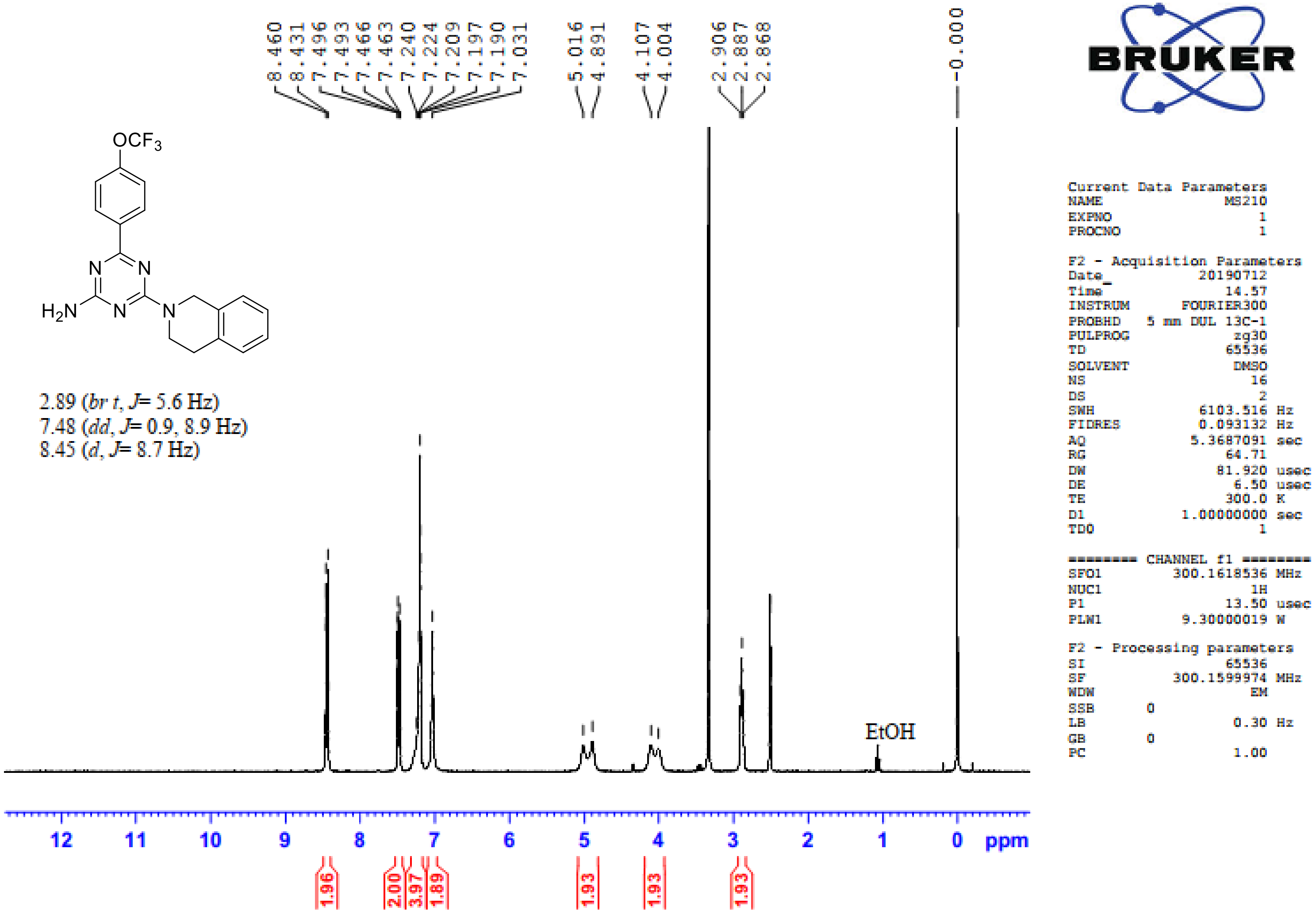

6-[4-(Trifluoromethoxy)phenyl]-4-(3,4-dihydroisoquinolin-2(1*H*)-yl)-1,3,5-triazine-2-amine (**9**{*6*,*12*})

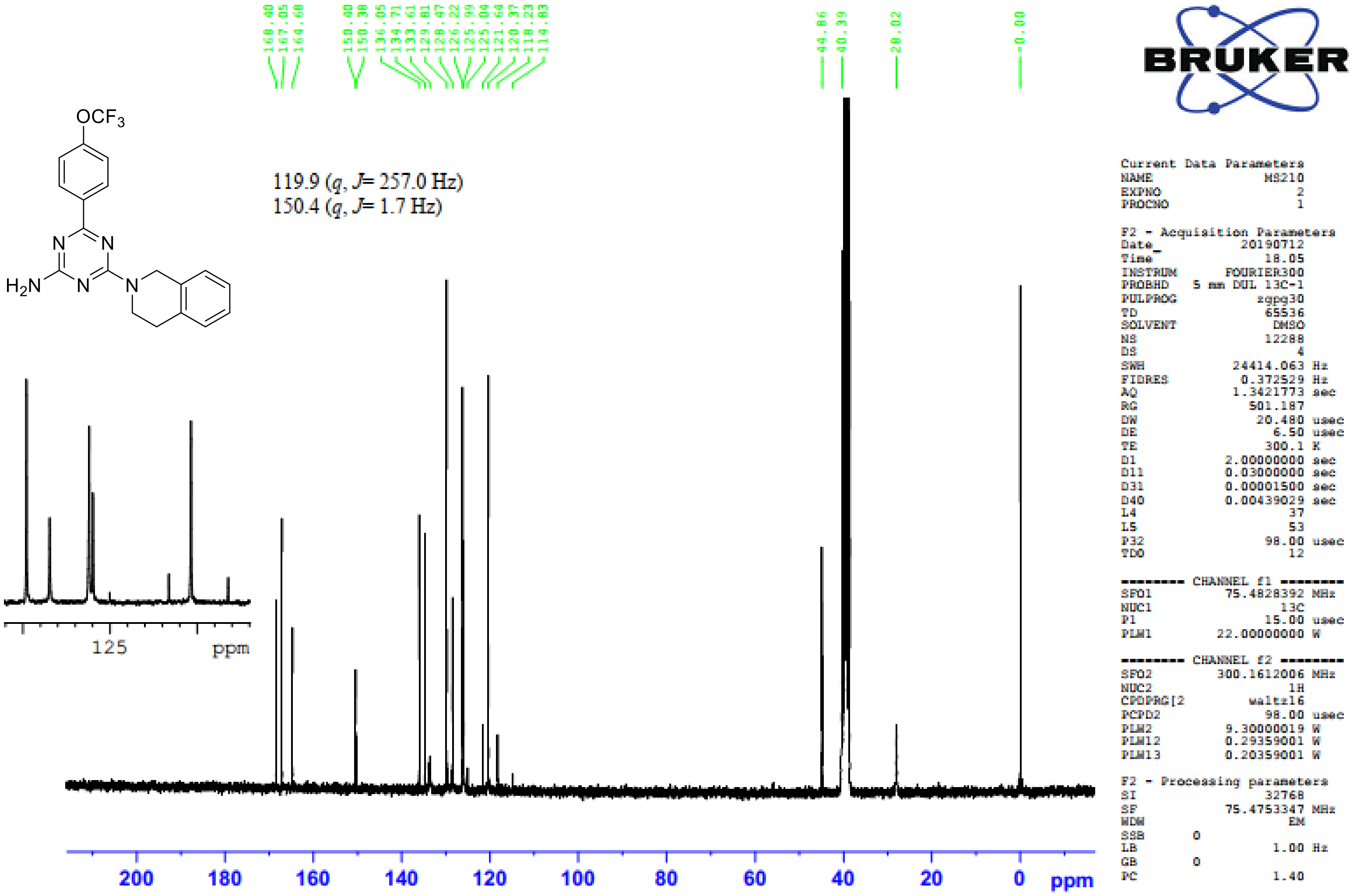

4-(3,4-Dihydroisoquinolin-2(1*H*)-yl)-6-(3-phenoxyphenyl)-1,3,5-triazine-2-amine (**9**{*6,13*})

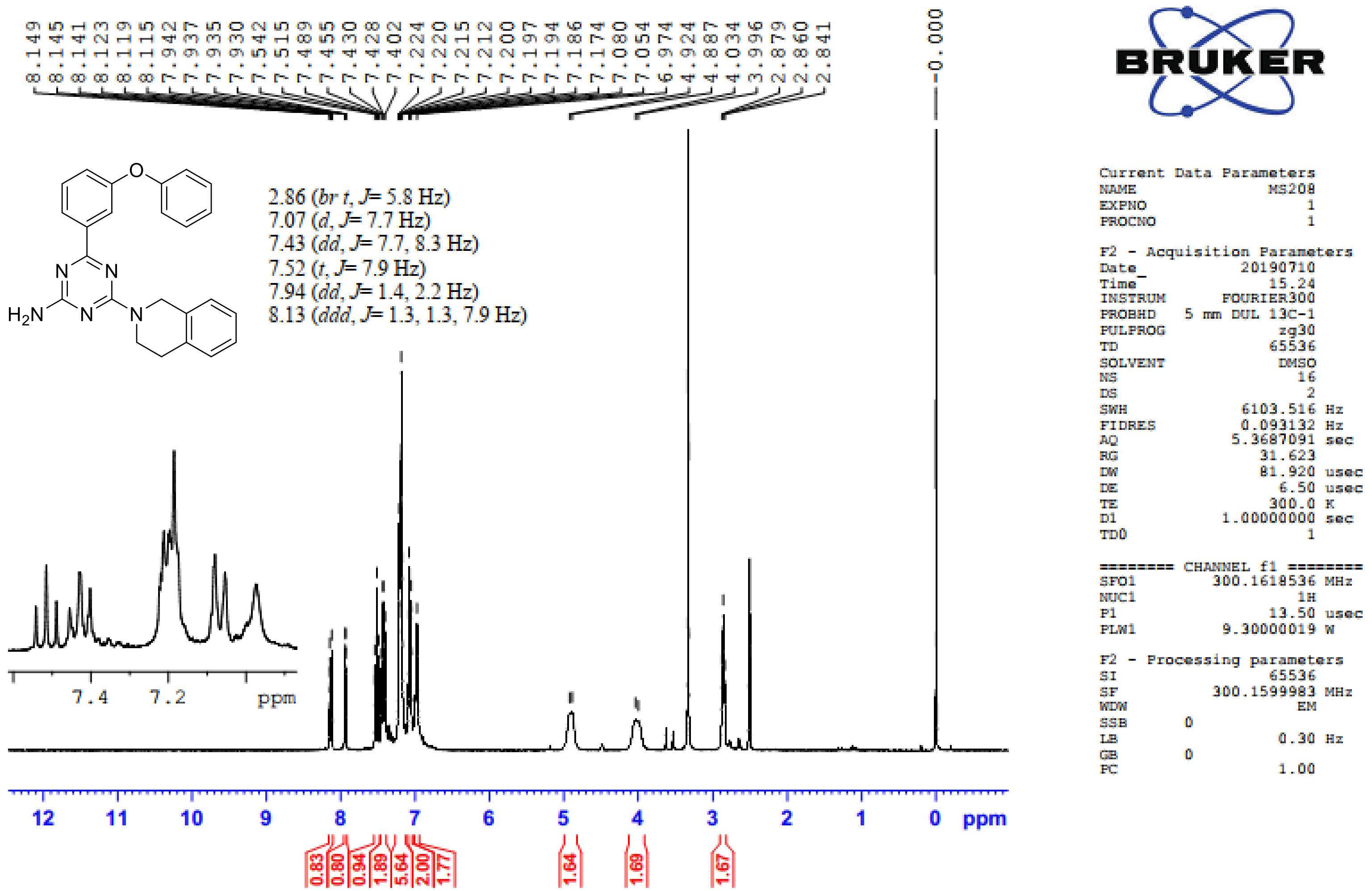

4-(3,4-Dihydroisoquinolin-2(1*H*)-yl)-6-(3-phenoxyphenyl)-1,3,5-triazine-2-amine (**9**{*6*,*13*})

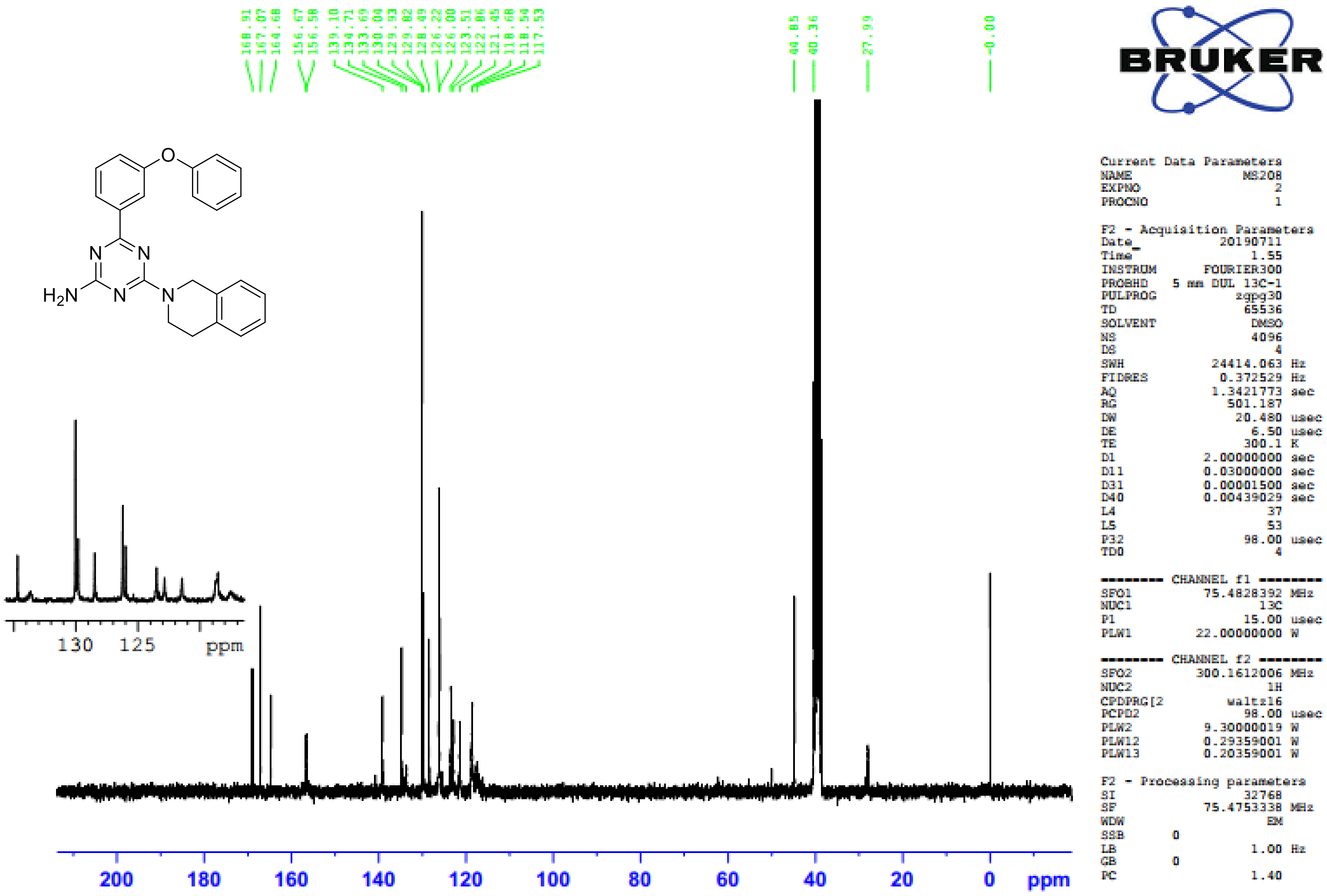

6-(3-(Benzyloxy)phenyl)-4-(3,4-dihydroisoquinolin-2(1*H*)-yl)-1,3,5-triazine-2-amine (**9**{*6*,*14*})

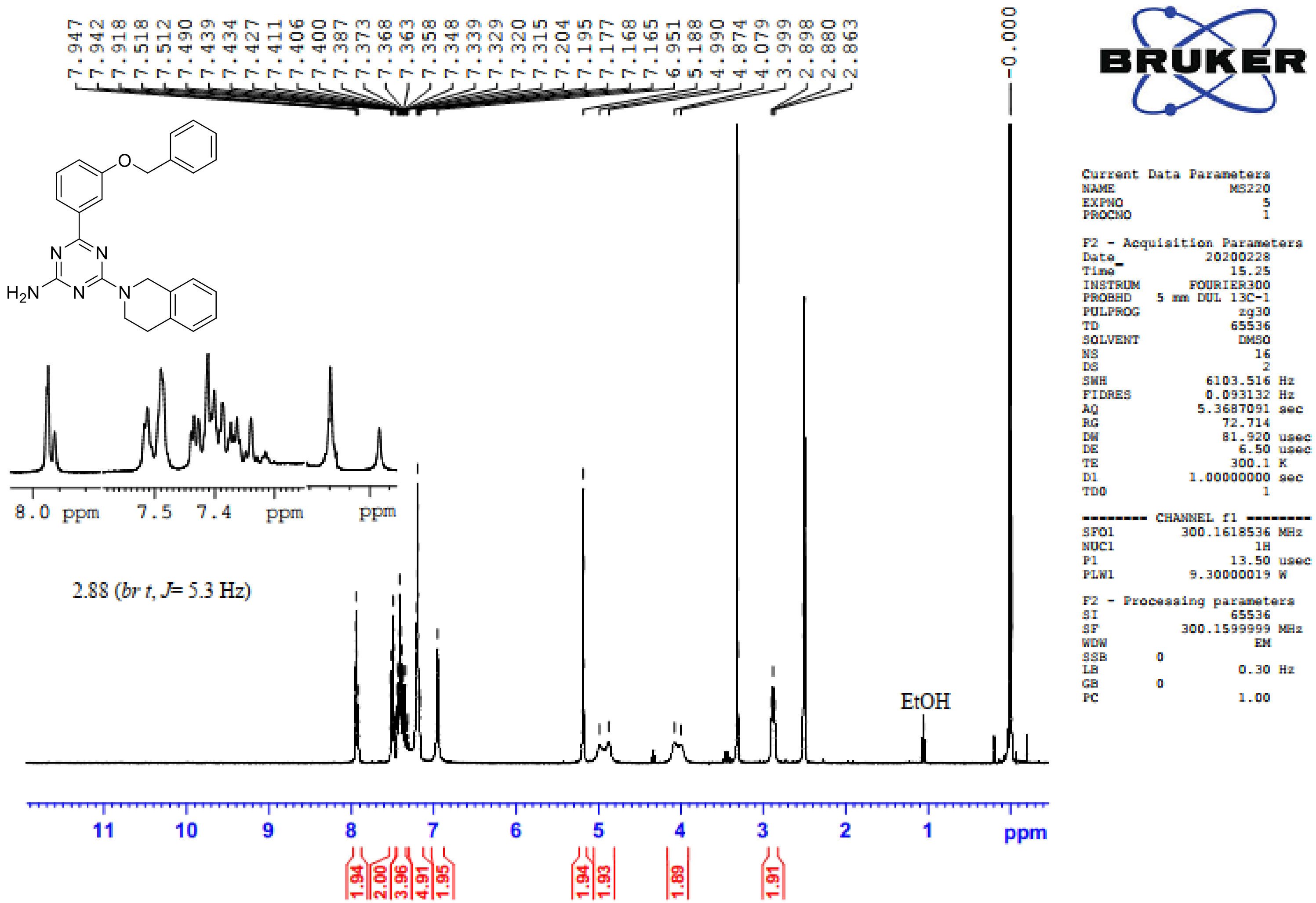

6-(3-(Benzyloxy)phenyl)-4-(3,4-dihydroisoquinolin-2(1*H*)-yl)-1,3,5-triazine-2-amine (**9**{*6,14*})

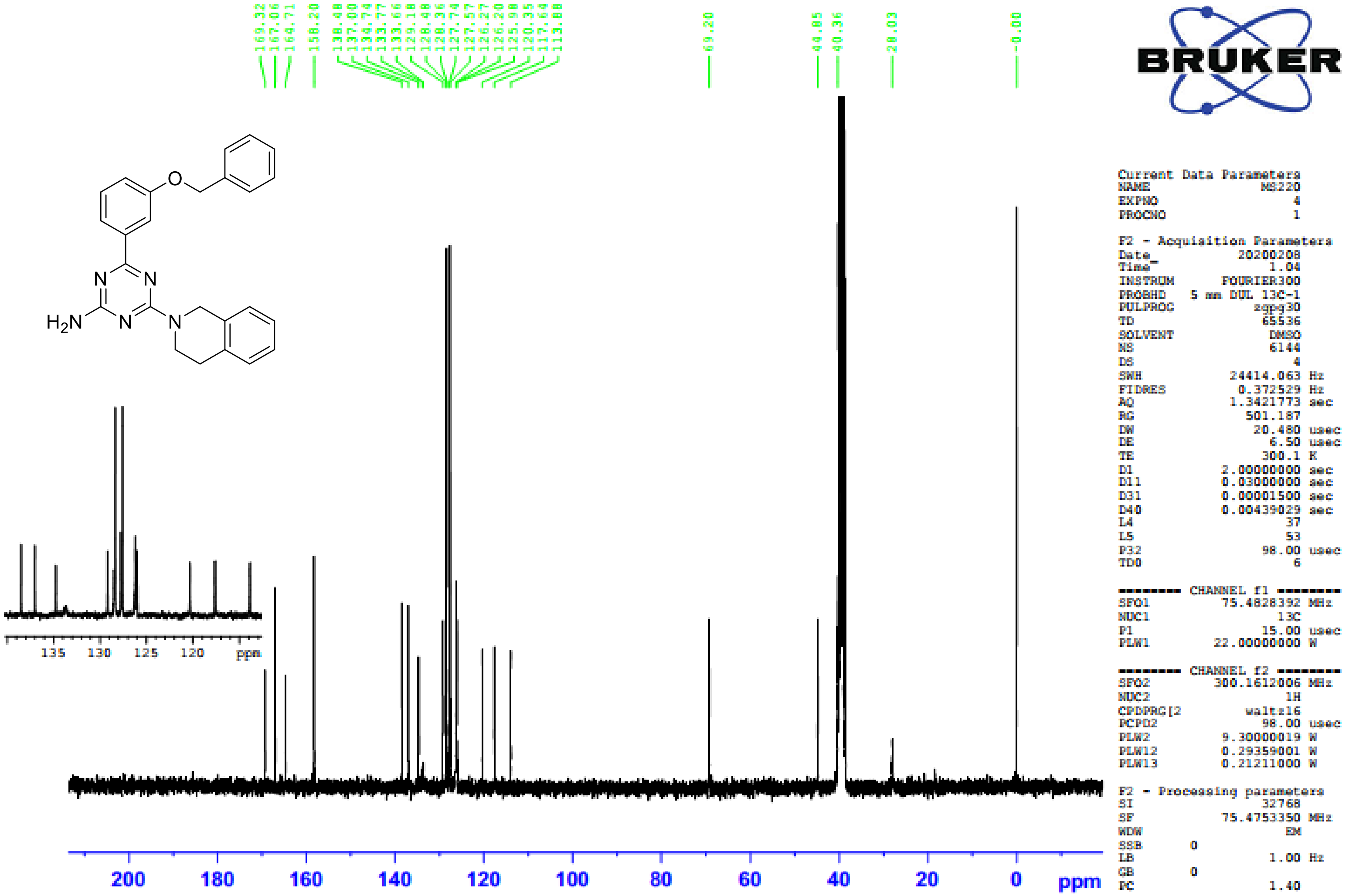

6-(4-(Benzyloxy)phenyl)-4-(3,4-dihydroisoquinolin-2(1*H*)-yl)-1,3,5-triazine-2-amine (**9**{*6*,*15*})

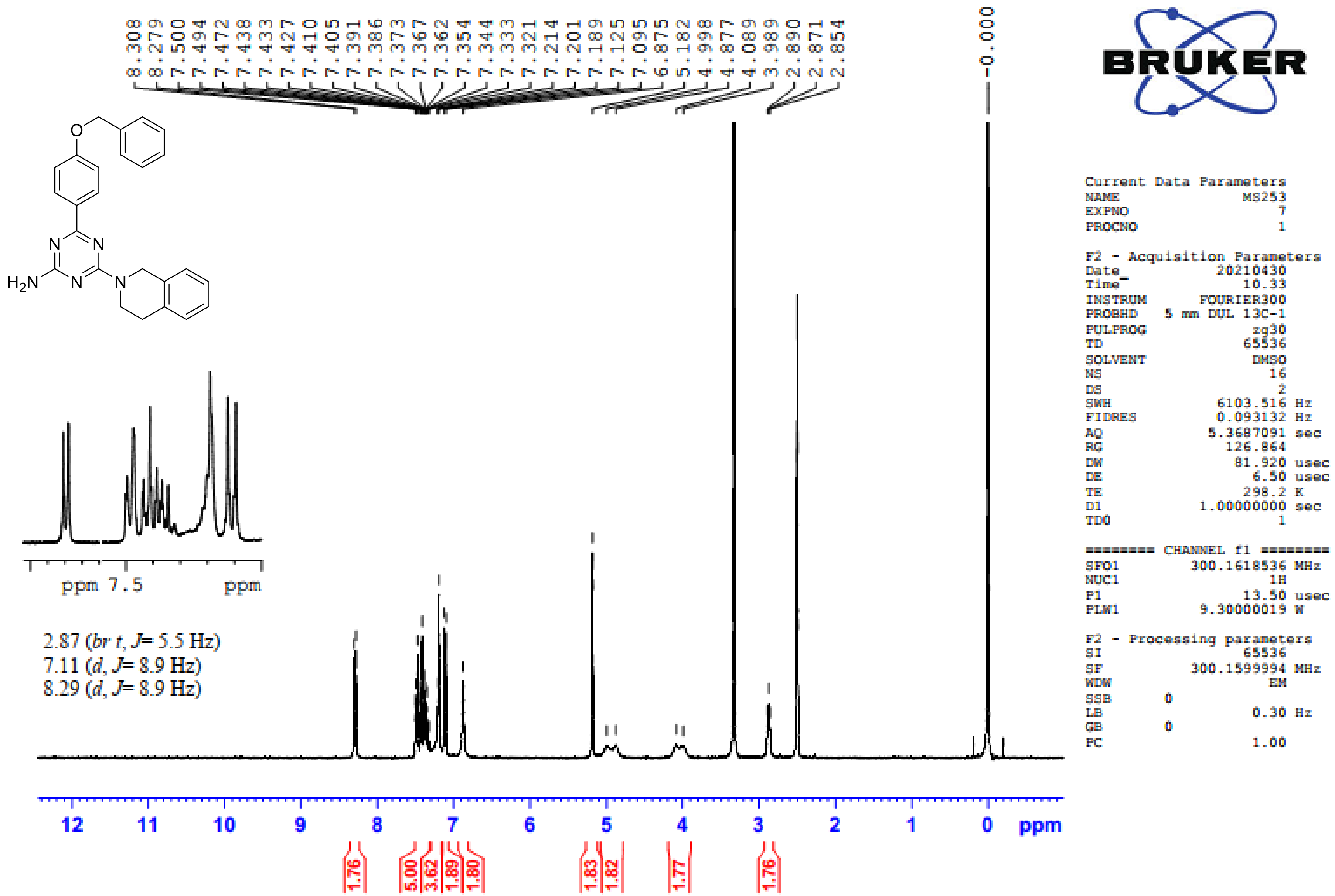

6-(4-(Benzyloxy)phenyl)-4-(3,4-dihydroisoquinolin-2(1*H*)-yl)-1,3,5-triazine-2-amine (**9**{*6,15*})

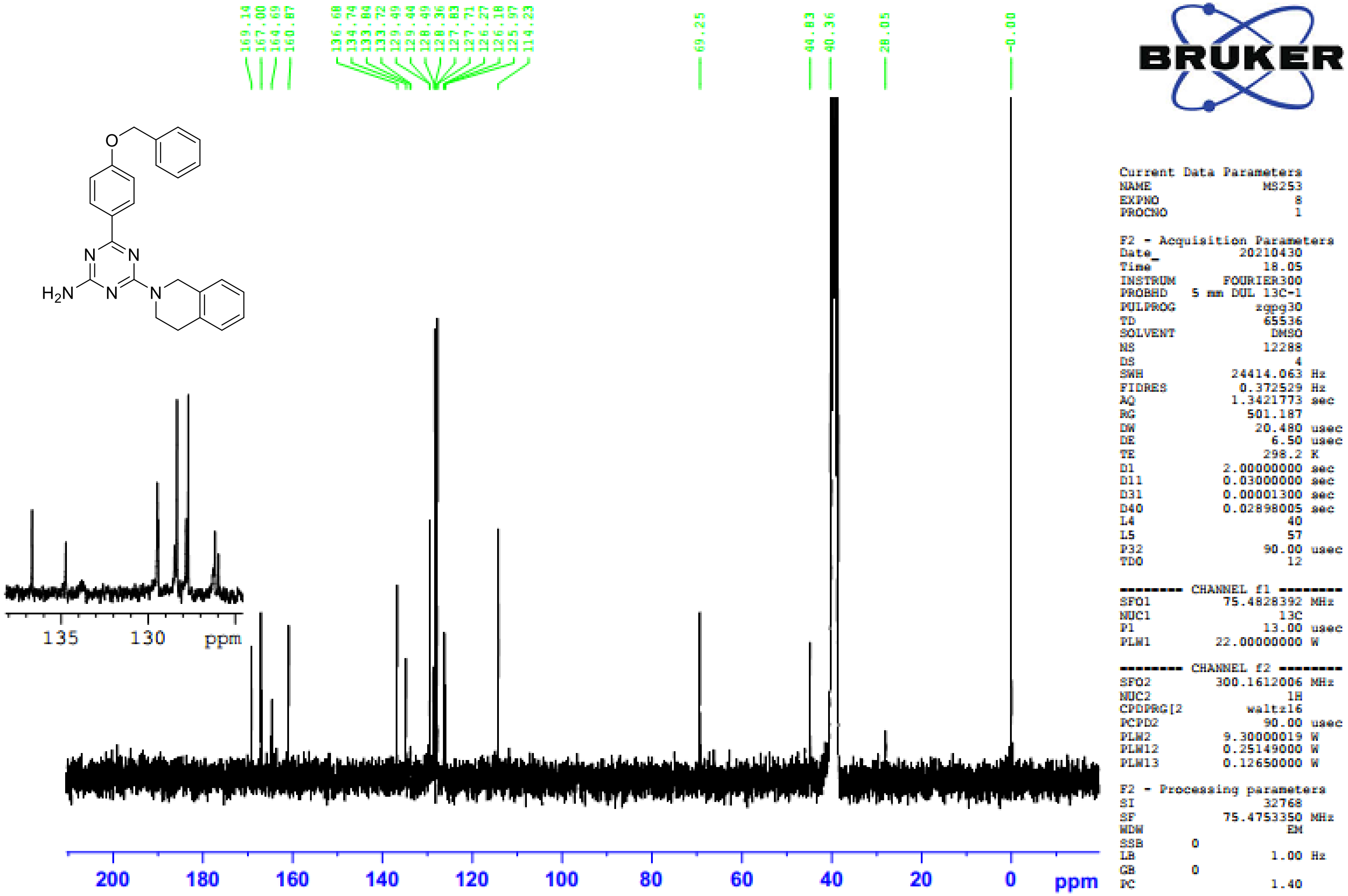

6-(3,4-Dimethoxyphenyl)-4-(3,4-dihydroisoquinolin-2(1*H*)-yl)-1,3,5-triazin-2-amine (**9**{*6*,*16*})

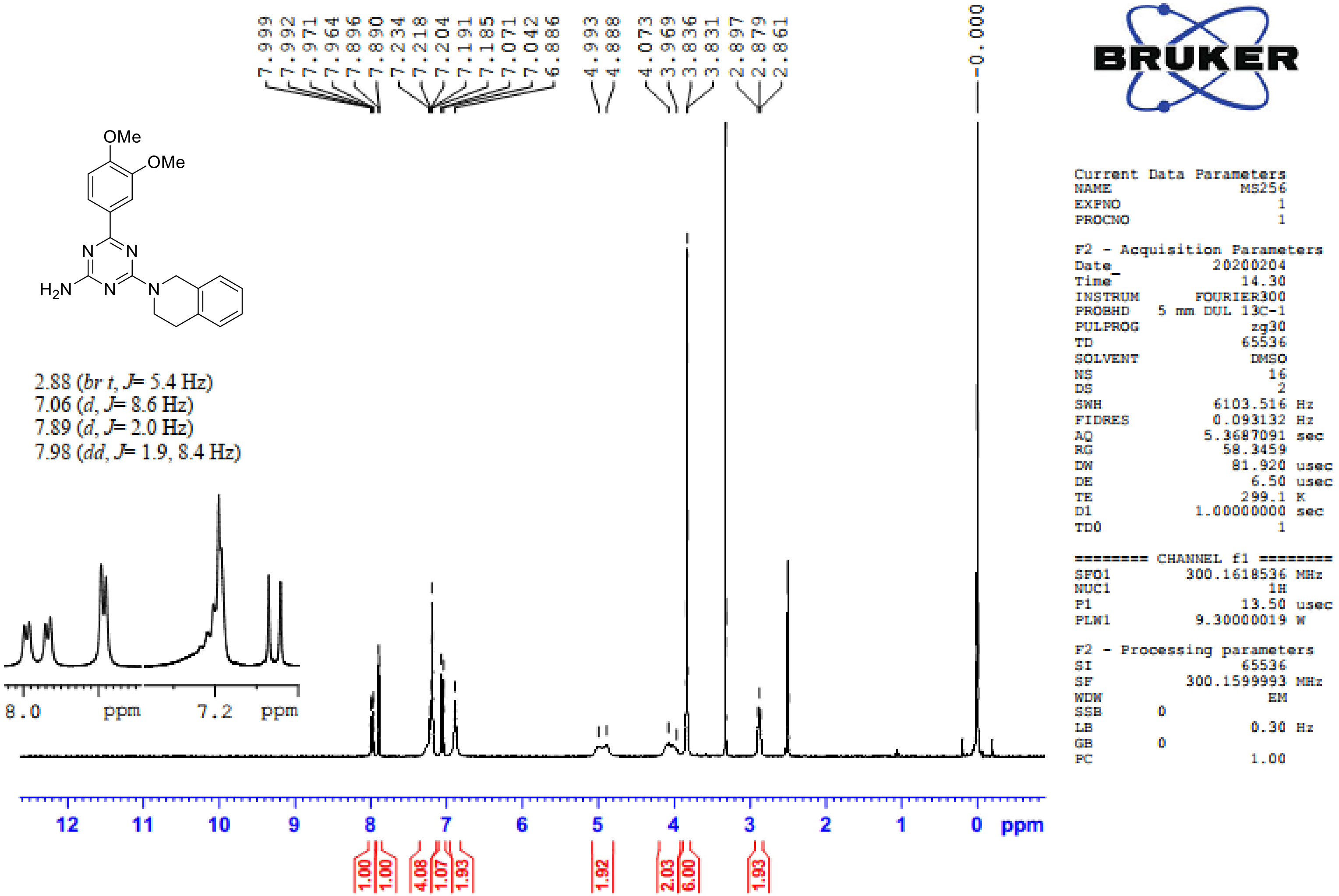

2.88 (*br t*, *J*= 5.4 Hz)
7.06 (*d*, *J*= 8.6 Hz)
7.89 (*d*, *J*= 2.0 Hz)
7.98 (*dd*, *J*= 1.9, 8.4 Hz)

6-(3,4-Dimethoxyphenyl)-4-(3,4-dihydroisoquinolin-2(1*H*)-yl)-1,3,5-triazin-2-amine (**9**{*6*,*16*})

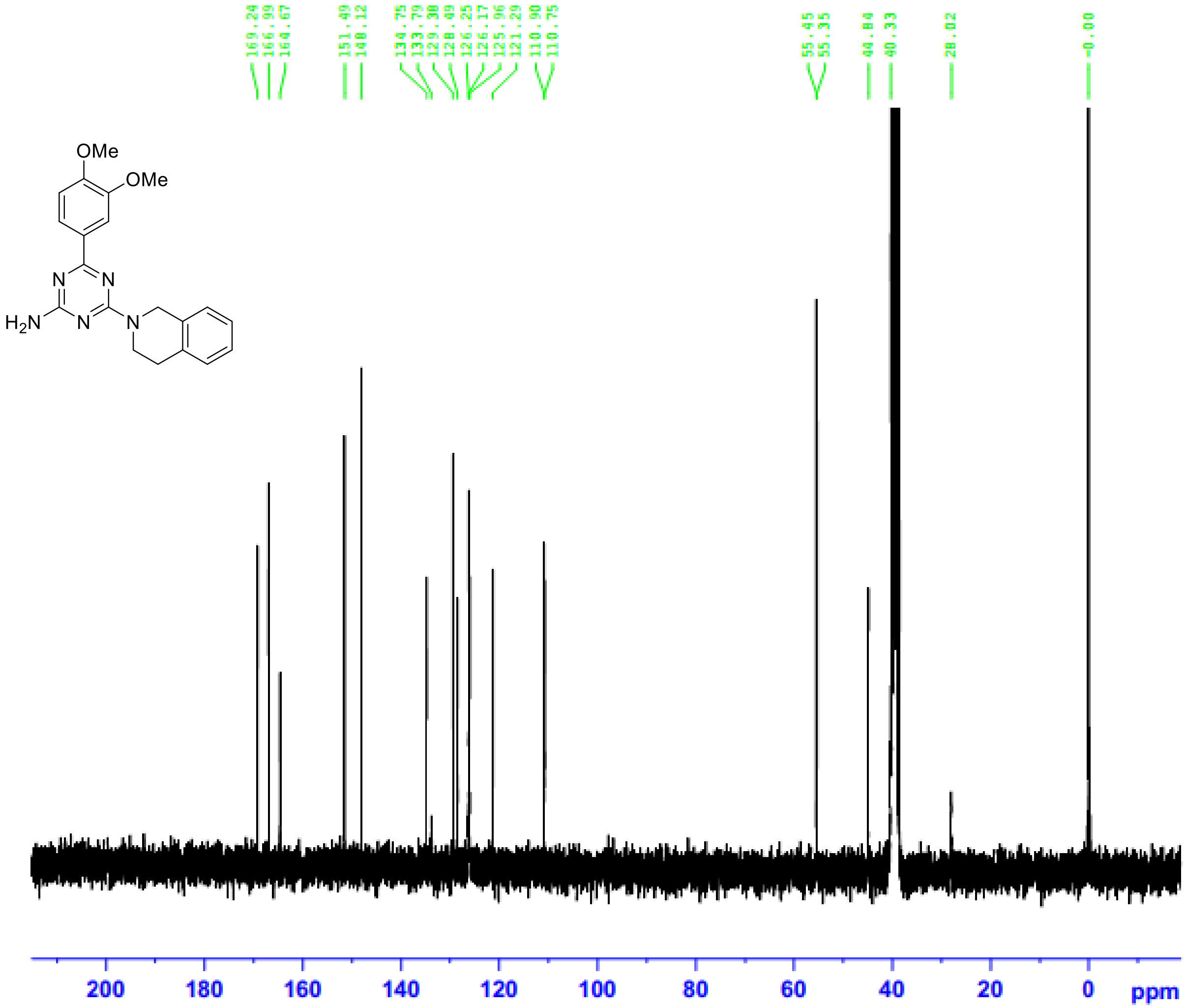


```
Current Data Parameters
NAME                MS256
EXPNO                   2
PROCNO                  1

F2 - Acquisition Parameters
Date_            20200206
Time                 1.19
INSTRUM        FOURIER300
PROBHD   5 mm DUL 13C-1
PULPROG           zgpg30
TD                  65536
SOLVENT              DMSO
NS                   6144
DS                      4
SWH             24414.063 Hz
FIDRES           0.372529 Hz
AQ              1.3421773 sec
RG                501.187
DW                 20.480 usec
DE                   6.50 usec
TE                  299.2 K
D1             2.00000000 sec
D11            0.03000000 sec
D31            0.00001500 sec
D40            0.00439029 sec
L4                     37
L5                     53
P32                 98.00 usec
TD0                     6

======== CHANNEL f1 ========
SFO1          75.4828392 MHz
NUC1                 13C
P1                 15.00 usec
PLW1         22.00000000 W

======== CHANNEL f2 ========
SFO2         300.1612006 MHz
NUC2                  1H
CPDPRG[2         waltz16
PCPD2              98.00 usec
PLW2          9.30000019 W
PLW12         0.29359001 W
PLW13         0.21211000 W

F2 - Processing parameters
SI                 32768
SF            75.4753353 MHz
WDW                   EM
SSB      0
LB                  0.50 Hz
GB       0
PC                  1.40
```

6-(3,4,5-Trimethoxyphenyl)-4-(3,4-dihydroisoquinolin-2(1*H*)-yl)-1,3,5-triazine-2-amine (**9**{*6,17*})

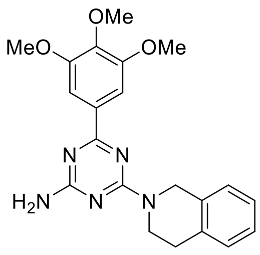

2.89 (*br t*, *J*= 5.4 Hz)

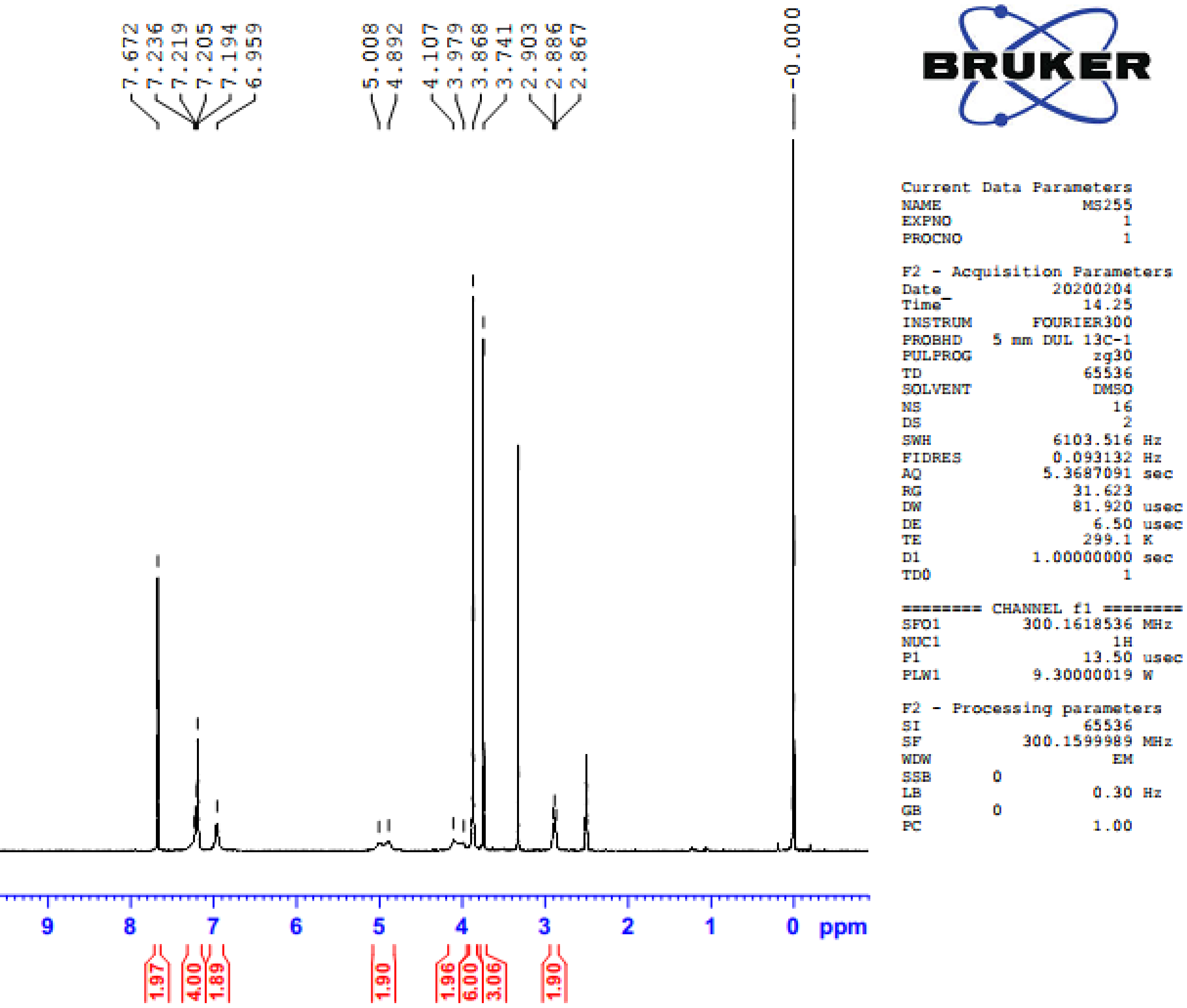

6-(3,4,5-Trimethoxyphenyl)-4-(3,4-dihydroisoquinolin-2(1*H*)-yl)-1,3,5-triazine-2-amine (**9**{*6*,*17*})

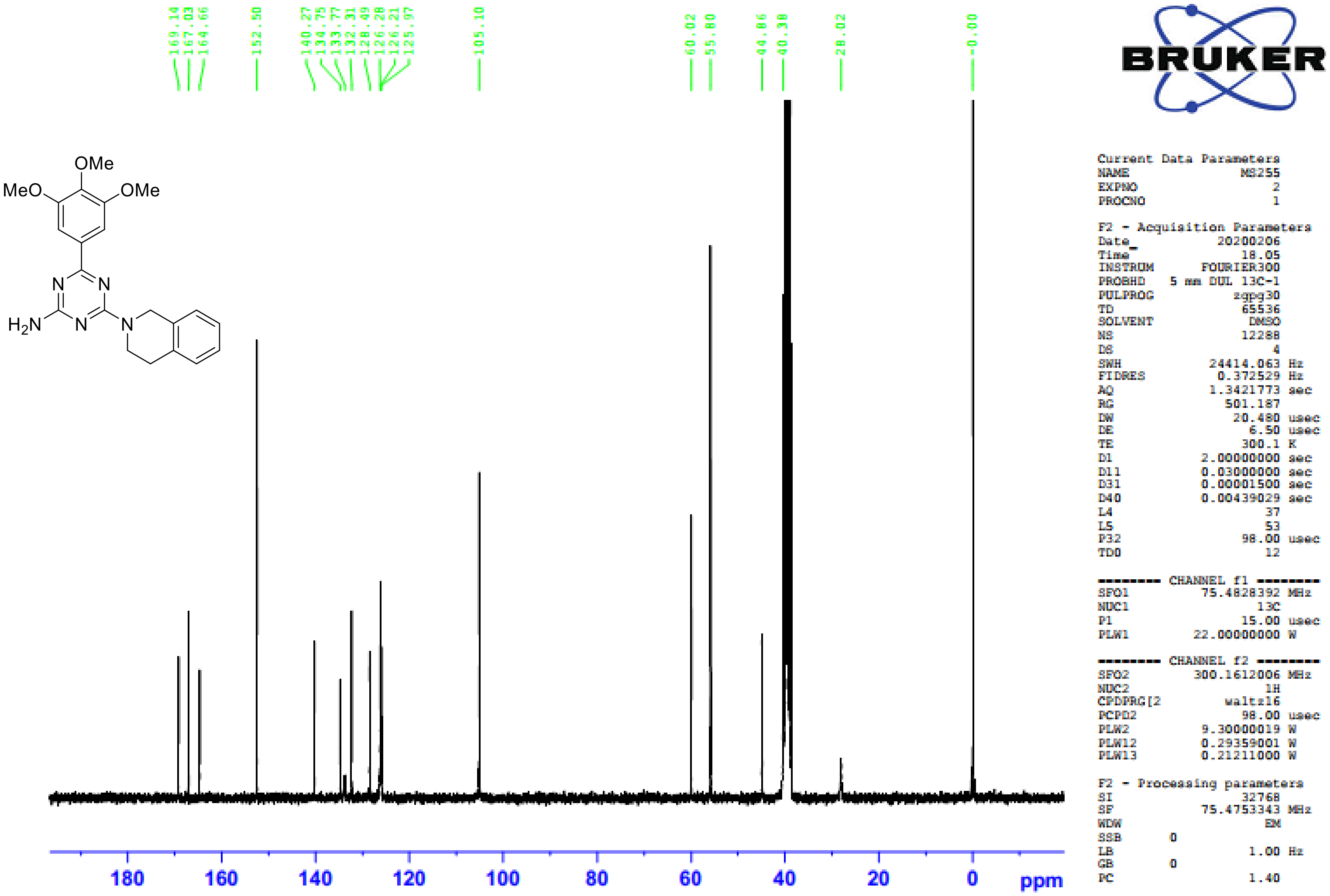

4-(3,4-Dihydroisoquinolin-2(1*H*)-yl)-6-(2-thienyl)-1,3,5-triazine-2-amine (**9**{*6*,*18*})

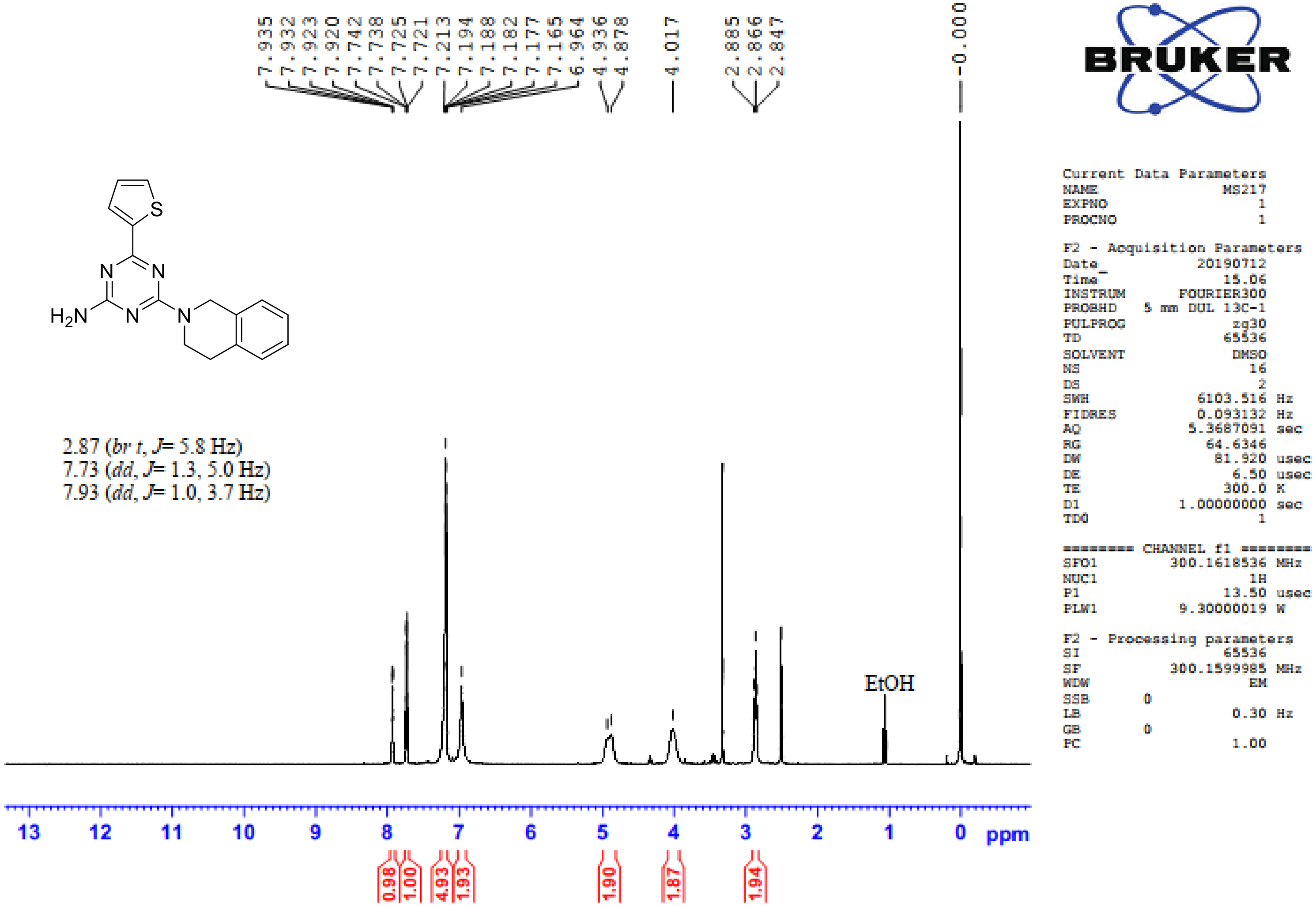

2.87 (*br t*, *J*= 5.8 Hz)
7.73 (*dd*, *J*= 1.3, 5.0 Hz)
7.93 (*dd*, *J*= 1.0, 3.7 Hz)

4-(3,4-Dihydroisoquinolin-2(1*H*)-yl)-6-(2-thienyl)-1,3,5-triazine-2-amine (**9**{*6*,*18*})

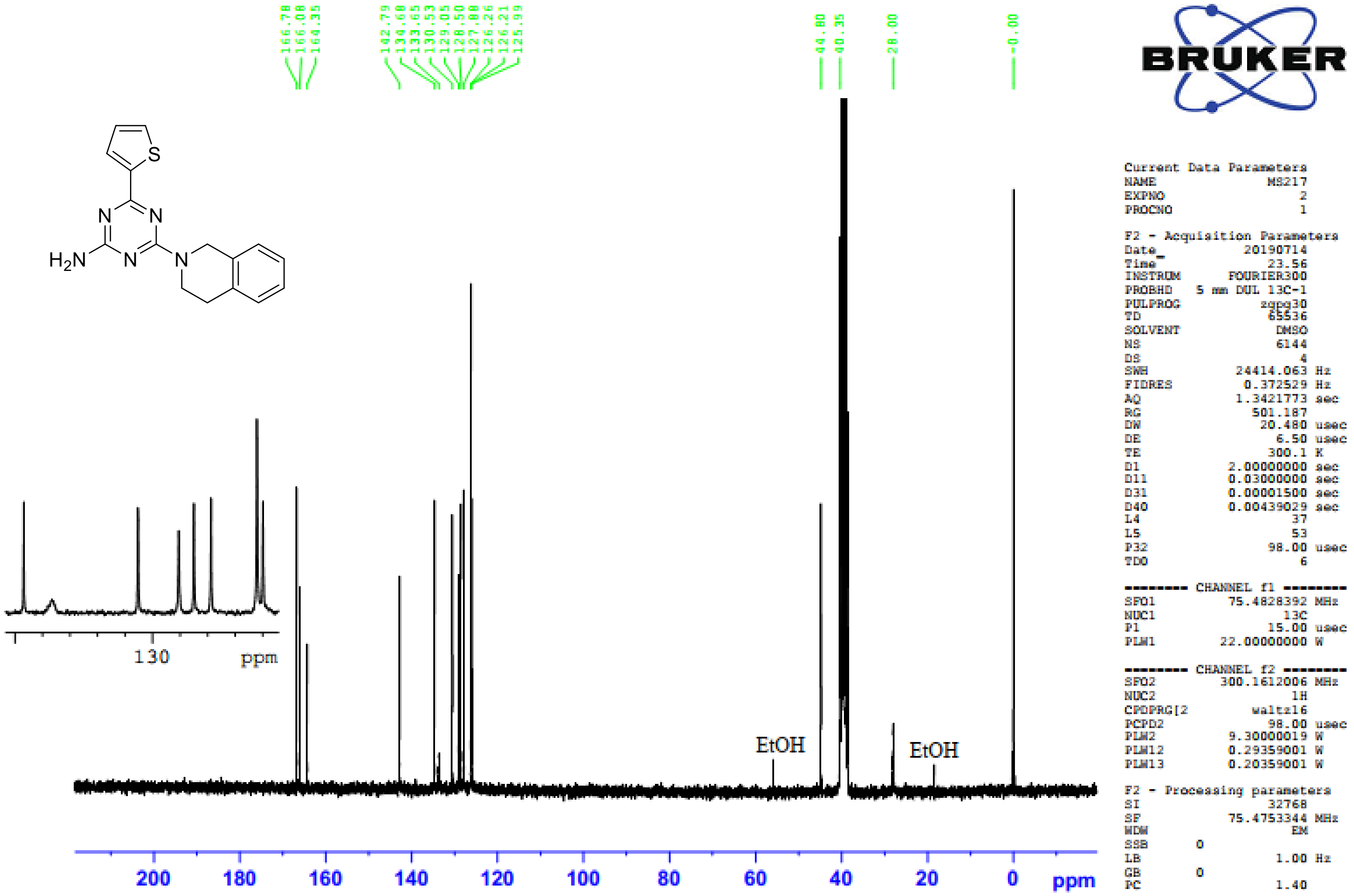

6-(4-Fluorophenyl)-4-(indolin-1-yl)-1,3,5-triazine-2-amine (**9**{*7*,*3*})

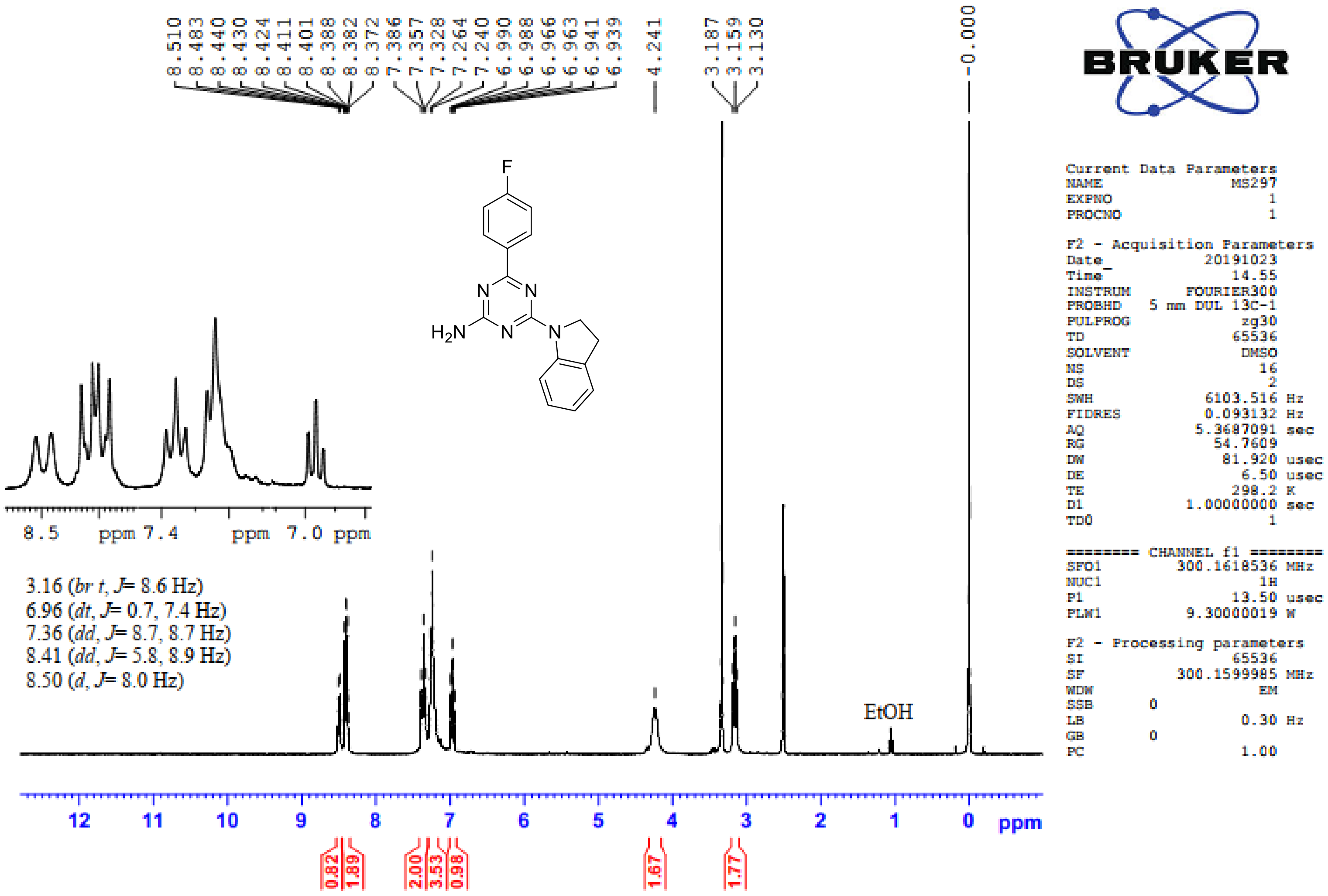

6-(4-Fluorophenyl)-4-(indolin-1-yl)-1,3,5-triazine-2-amine (**9**{*7*,*3*})

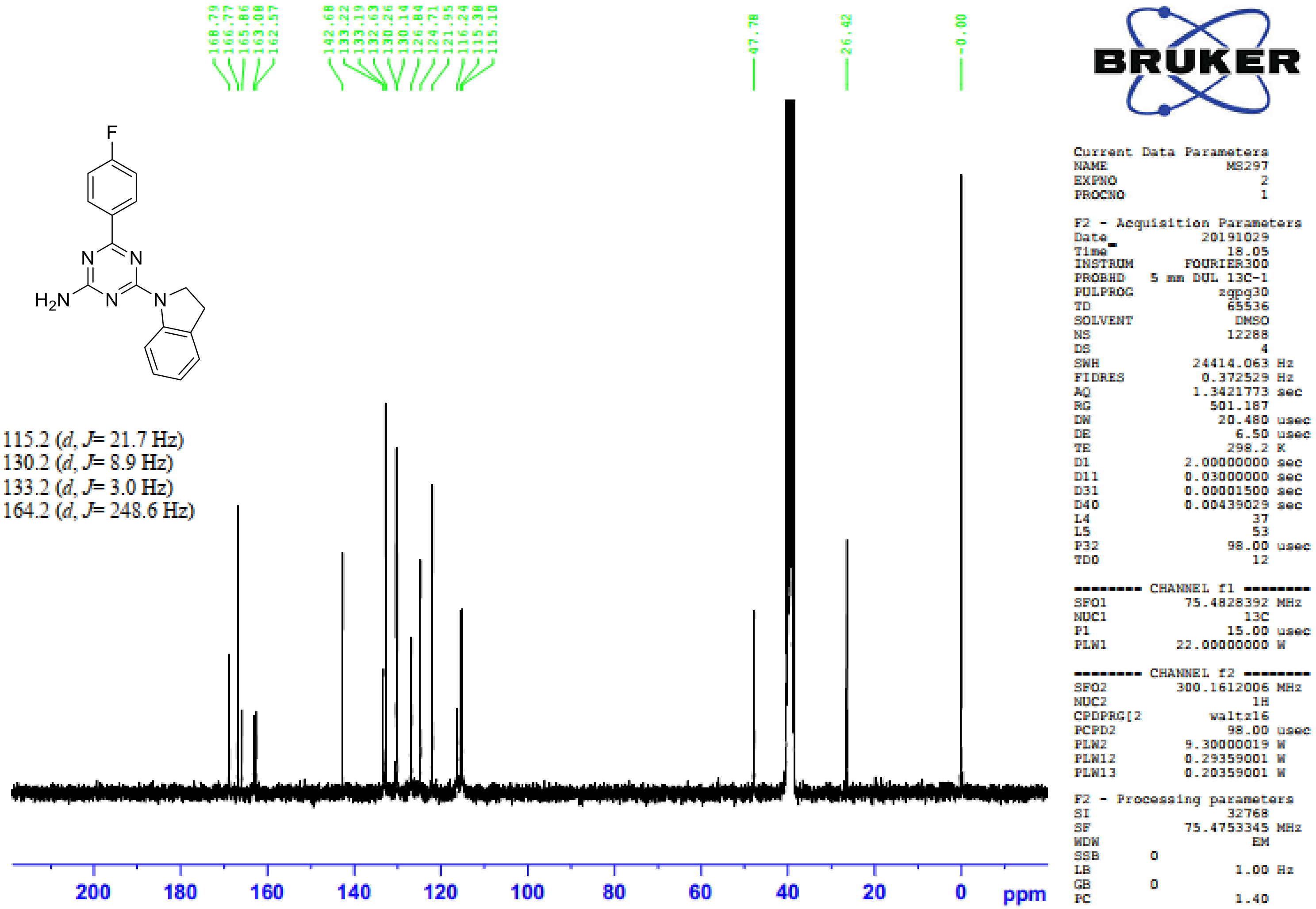

6-[4-(*N*,*N*-Dimethylamino)phenyl]-4-(indolin-1-yl)-1,3,5-triazine-2-amine (**9**{*7*,*8*})

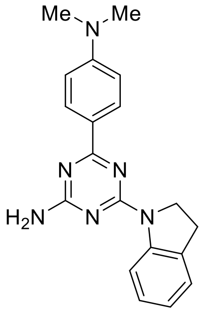

3.14 (*br t*, *J*= 8.2 Hz)
4.21 (*br t*, *J*= 8.6 Hz)
6.79 (*d*, *J*= 8.9 Hz)
8.21 (*d*, *J*= 9.1 Hz)
8.51 (*d*, *J*= 7.9 Hz)

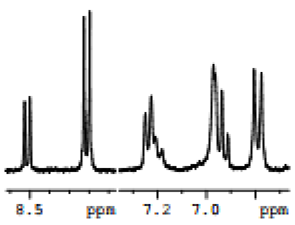

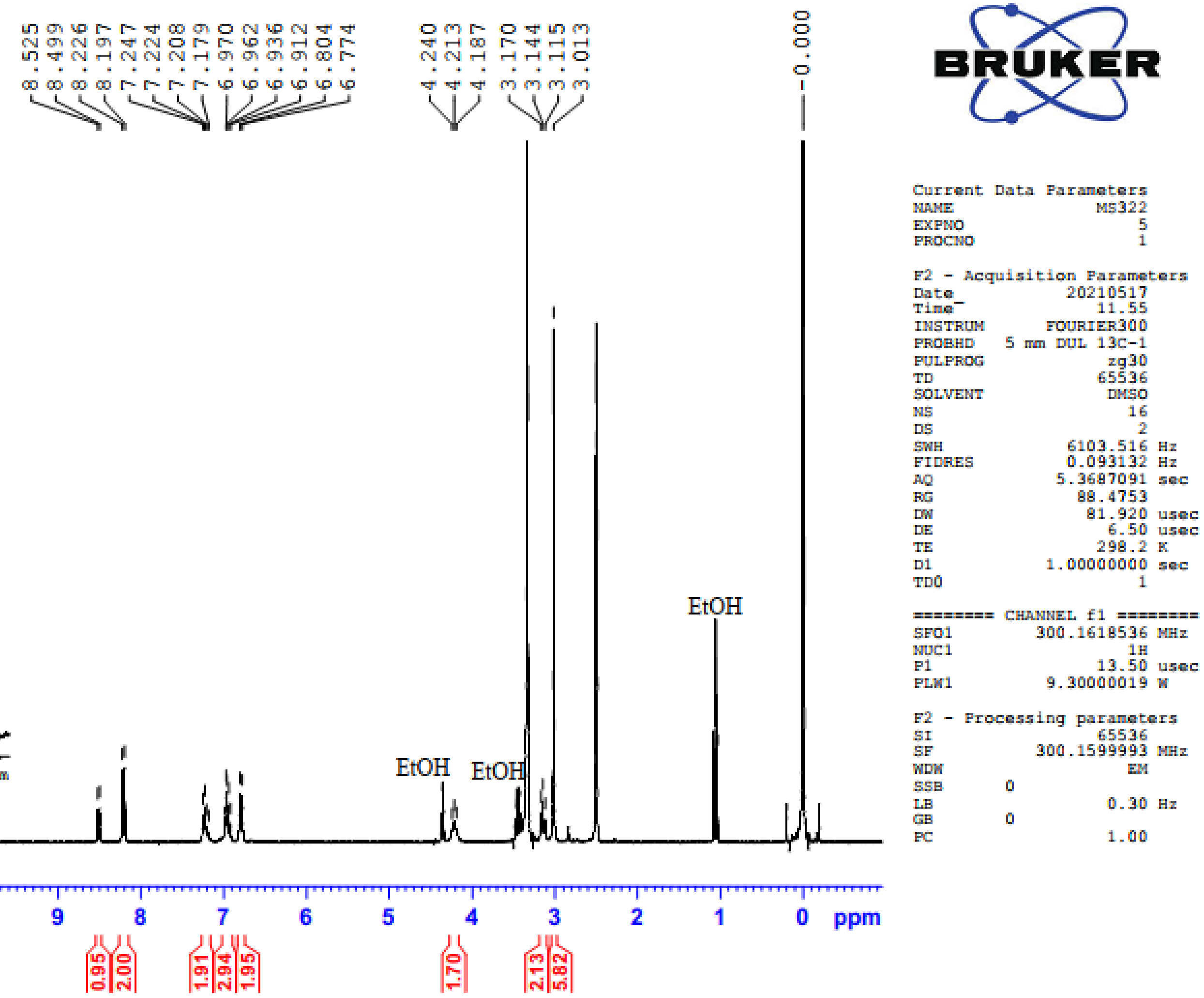

6-[4-(*N*,*N*-Dimethylamino)phenyl]-4-(indolin-1-yl)-1,3,5-triazine-2-amine (**9**{*7*,*8*})

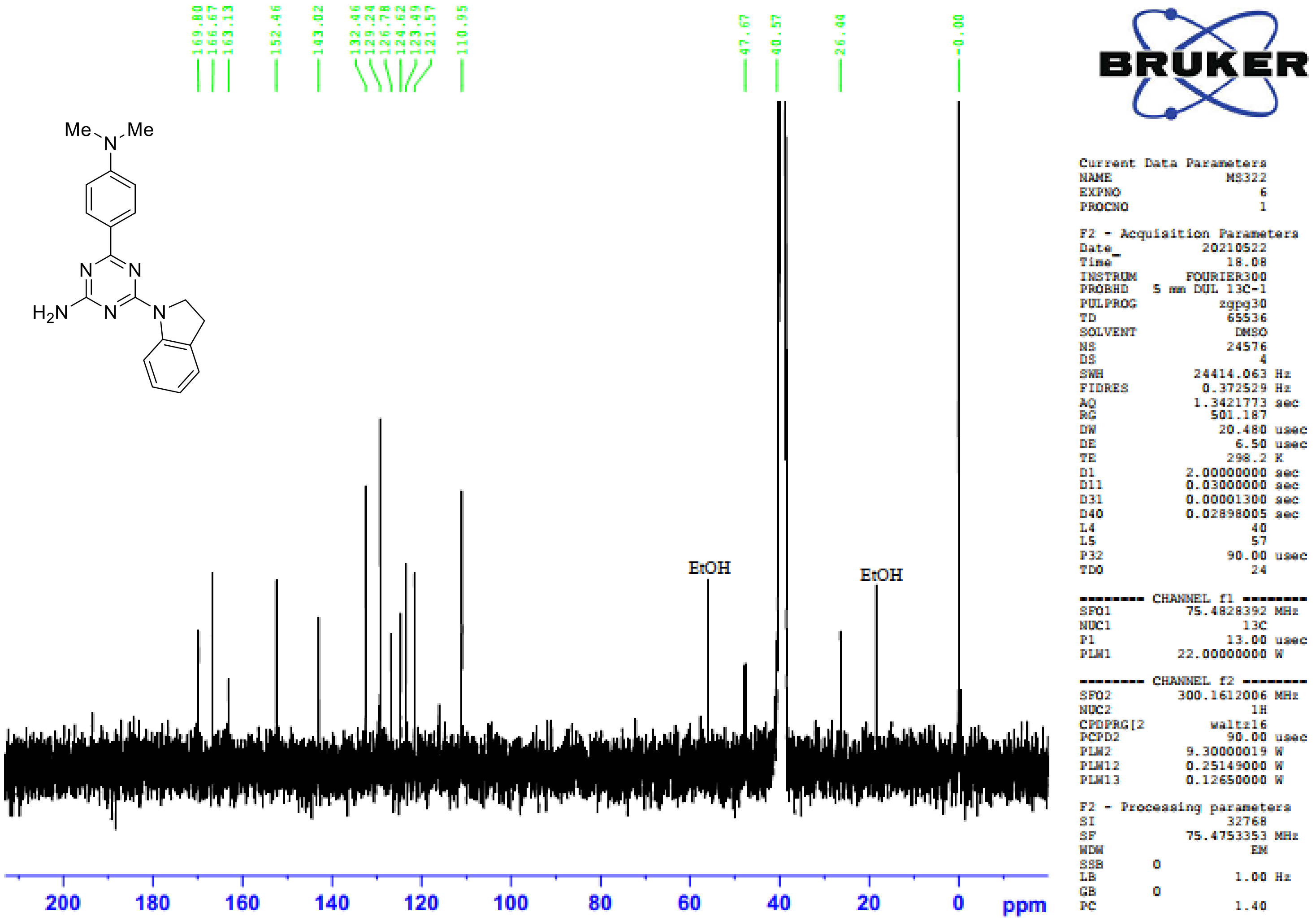

6-(4-Methoxyphenyl)-4-(indolin-1-yl)-1,3,5-triazine-2-amine (**9**{*7*,*9*})

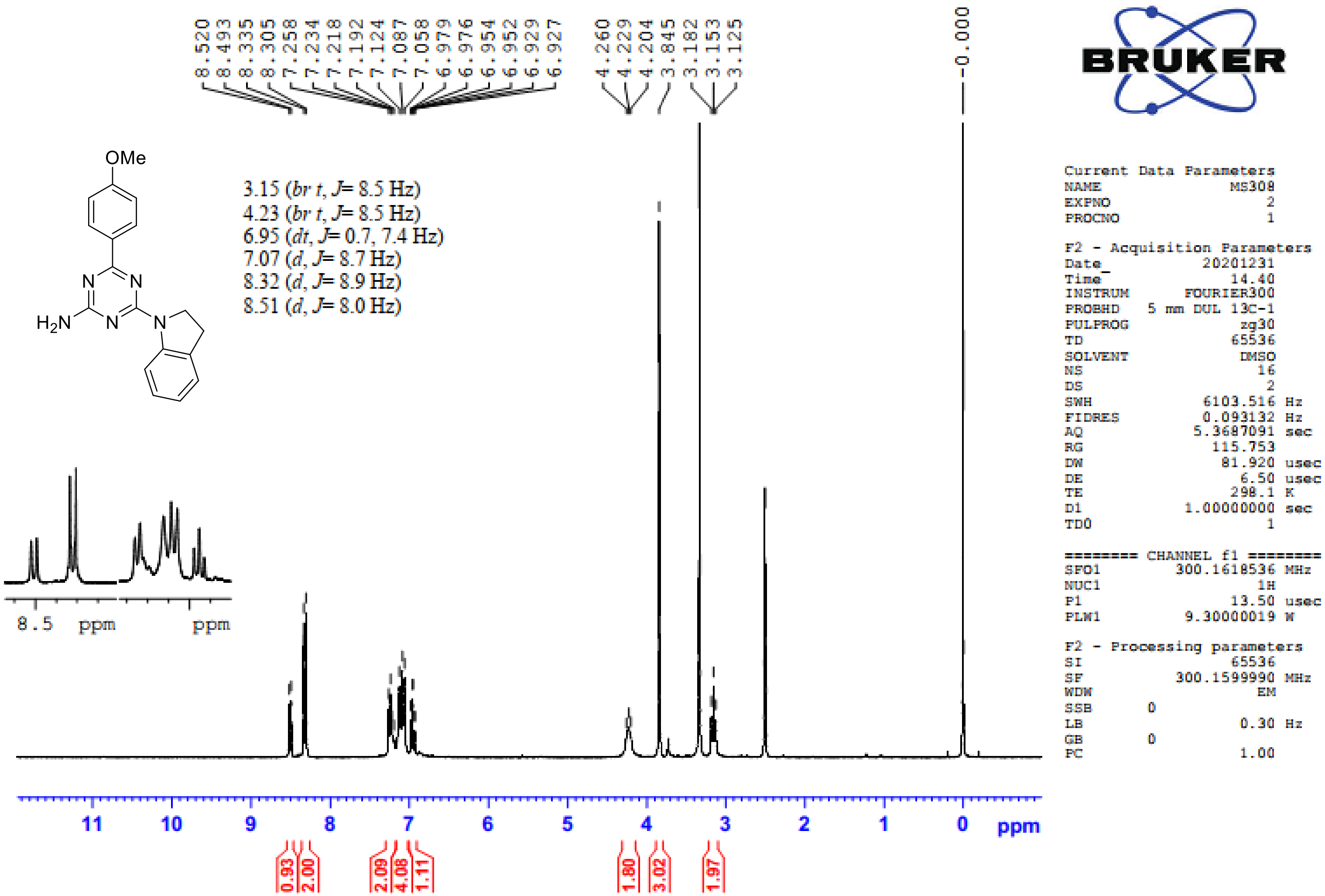

6-(4-Methoxyphenyl)-4-(indolin-1-yl)-1,3,5-triazine-2-amine (**9**{*7*,*9*})

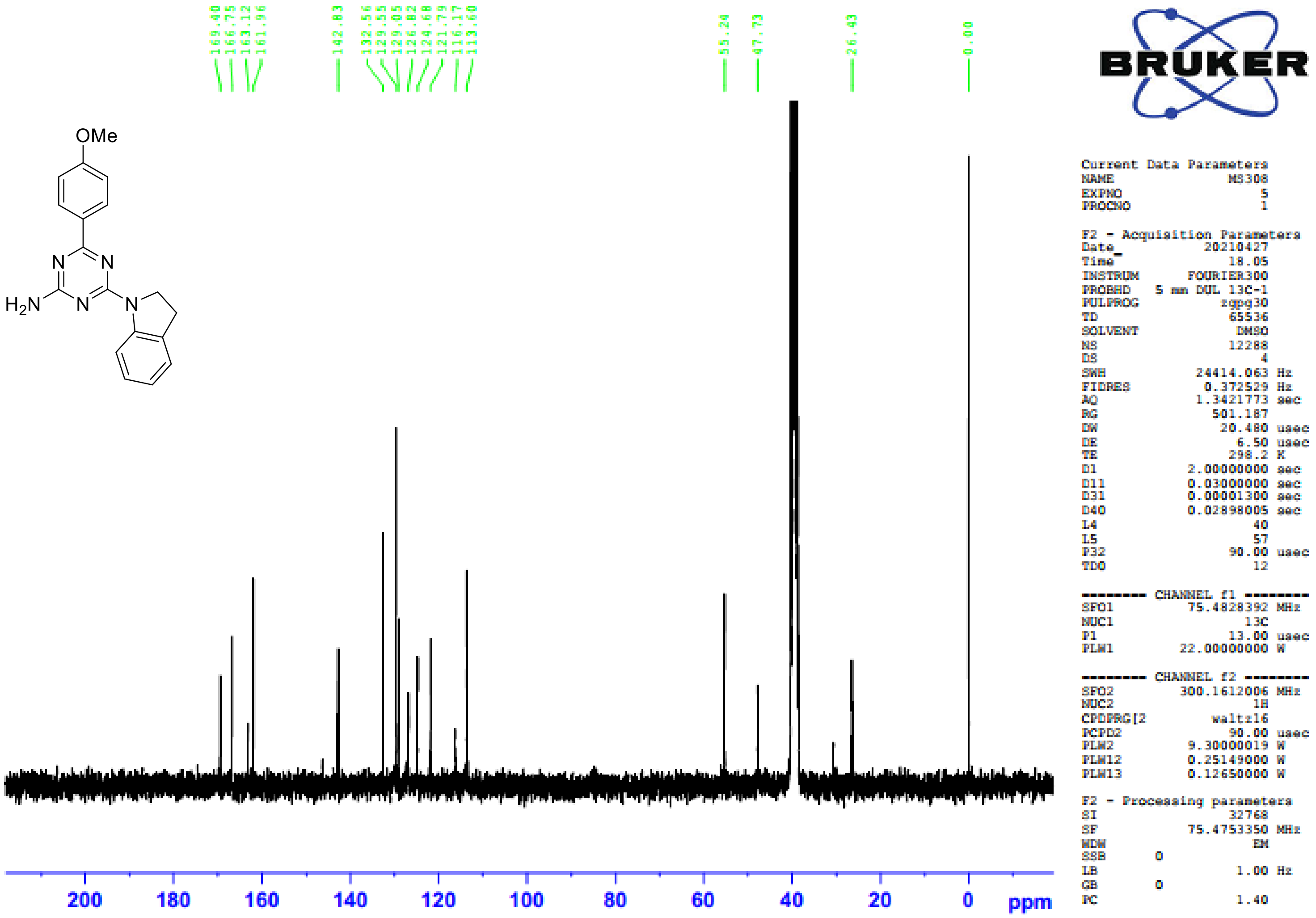

6-[4-(Trifluoromethyl)phenyl]-4-(indolin-1-yl)-1,3,5-triazine-2-amine (**9**{*7,11*})

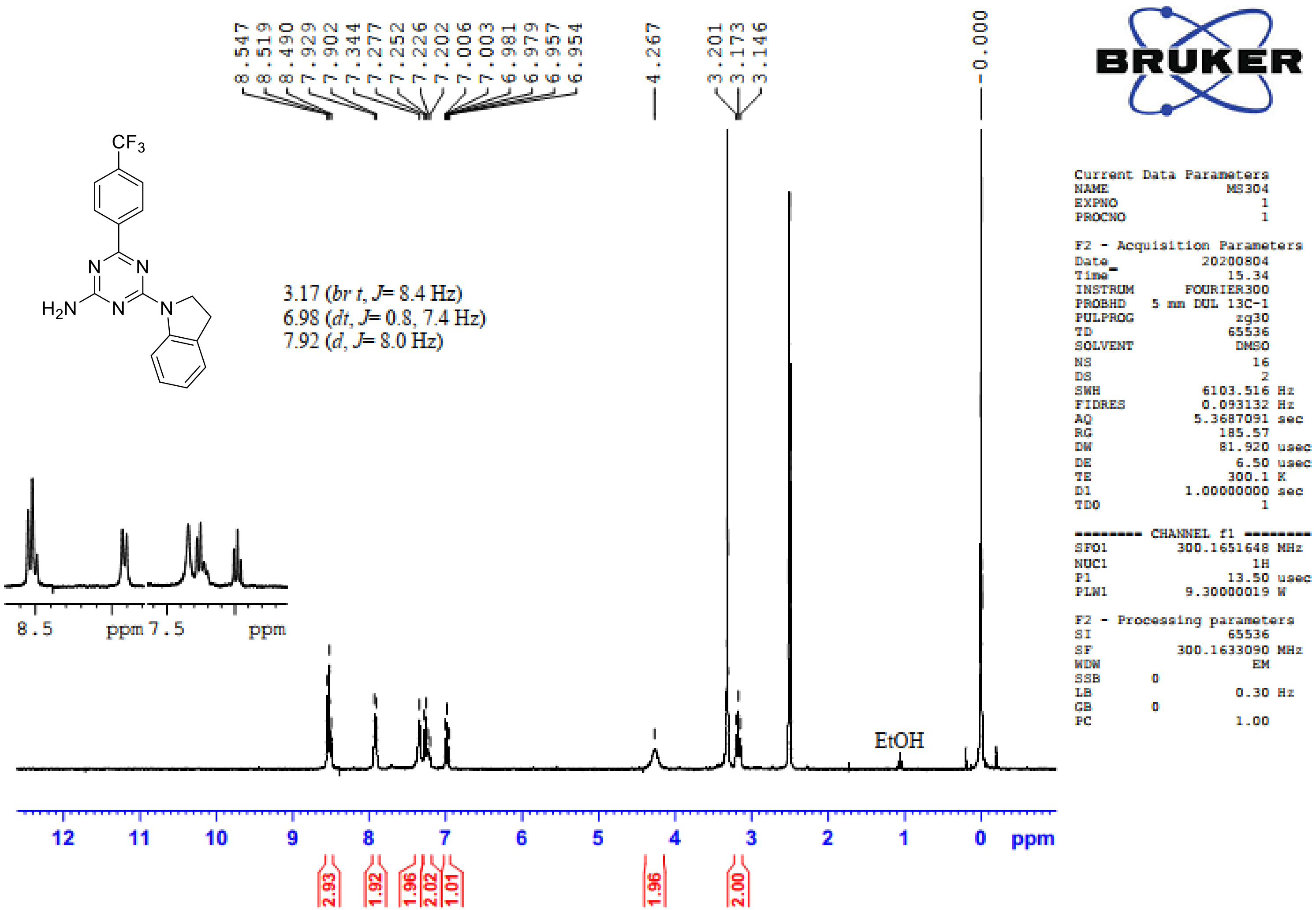

6-[4-(Trifluoromethoxy)phenyl]-4-(indolin-1-yl)-1,3,5-triazine-2-amine (**9**{*7*,*12*})

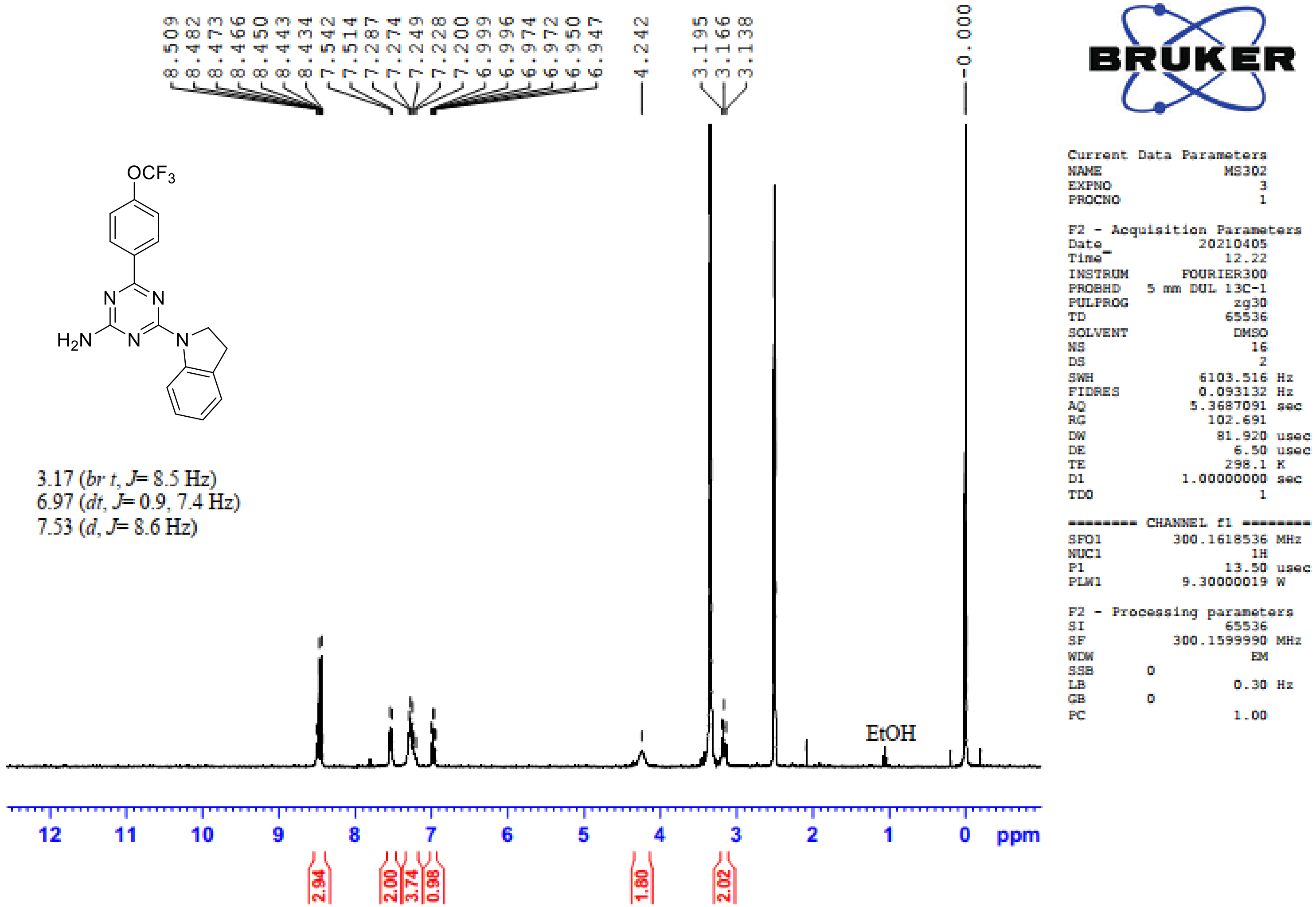

6-(4-(Benzyloxy)phenyl)-4-(indolin-1-yl)-1,3,5-triazine-2-amine (**9**{*7,15*})

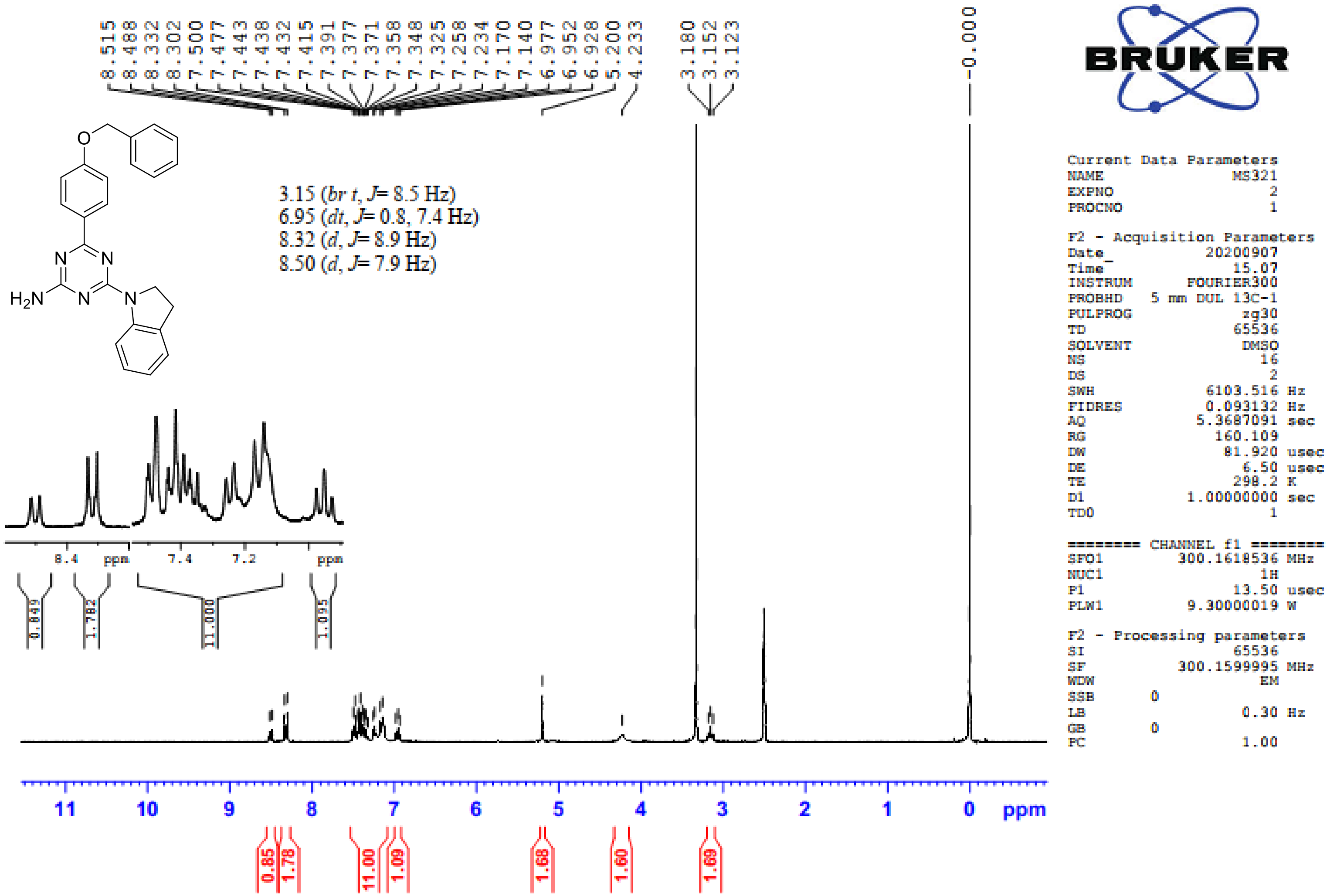

6-(4-(Benzyloxy)phenyl)-4-(indolin-1-yl)-1,3,5-triazine-2-amine (**9**{*7*,*15*})

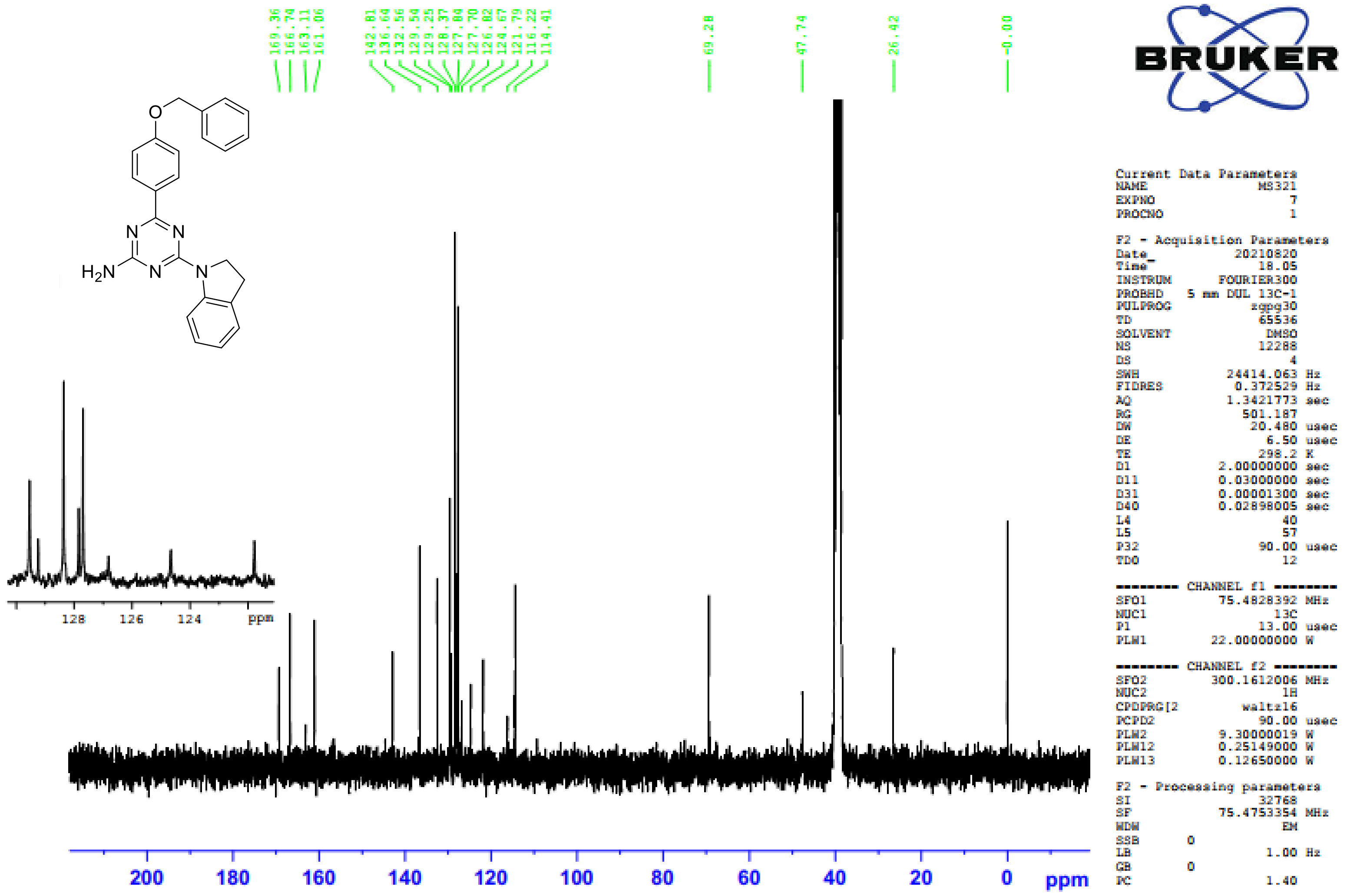

6-(3,4-Dimethoxyphenyl)-4-(indolin-1-yl)-1,3,5-triazine-2-amine (**9**{*7*,*16*})

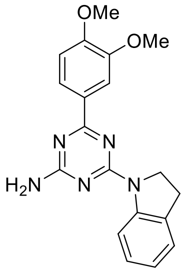

3.16 (*br t*, *J*= 8.6 Hz)
6.95 (*dt*, *J*= 0.9, 7.4 Hz)
7.94 (*d*, *J*= 1.9 Hz)
8.00 (*dd*, *J*= 1.9, 8.4 Hz)
8.52 (*d*, *J*= 7.9 Hz)

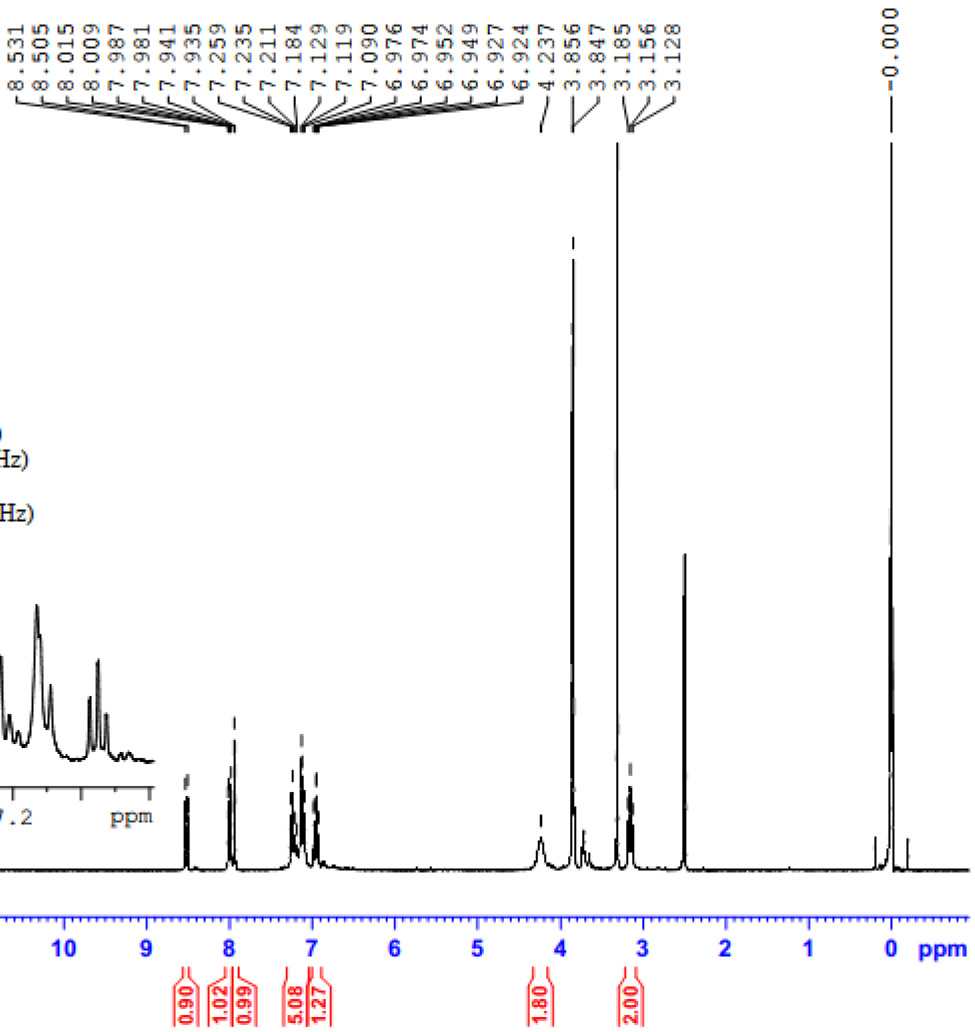

Current Data Parameters
NAME MS324
EXPNO 1
PROCNO 1

F2 - Acquisition Parameters
Date_ 20191104
Time 15.11
INSTRUM FOURIER300
PROBHD 5 mm DUL 13C-1
PULPROG zg30
TD 65536
SOLVENT DMSO
NS 16
DS 2
SWH 6103.516 Hz
FIDRES 0.093132 Hz
AQ 5.3687091 sec
RG 50.7488
DW 81.920 usec
DE 6.50 usec
TE 300.0 K
D1 1.00000000 sec
TD0 1

======== CHANNEL f1 ========
SFO1 300.1618536 MHz
NUC1 1H
P1 13.50 usec
PLW1 9.30000019 W

F2 - Processing parameters
SI 65536
SF 300.1599996 MHz
WDW EM
SSB 0
LB 0.30 Hz
GB 0
PC 1.00

6-(3,4-Dimethoxyphenyl)-4-(indolin-1-yl)-1,3,5-triazine-2-amine (**9**{*7*,*16*})

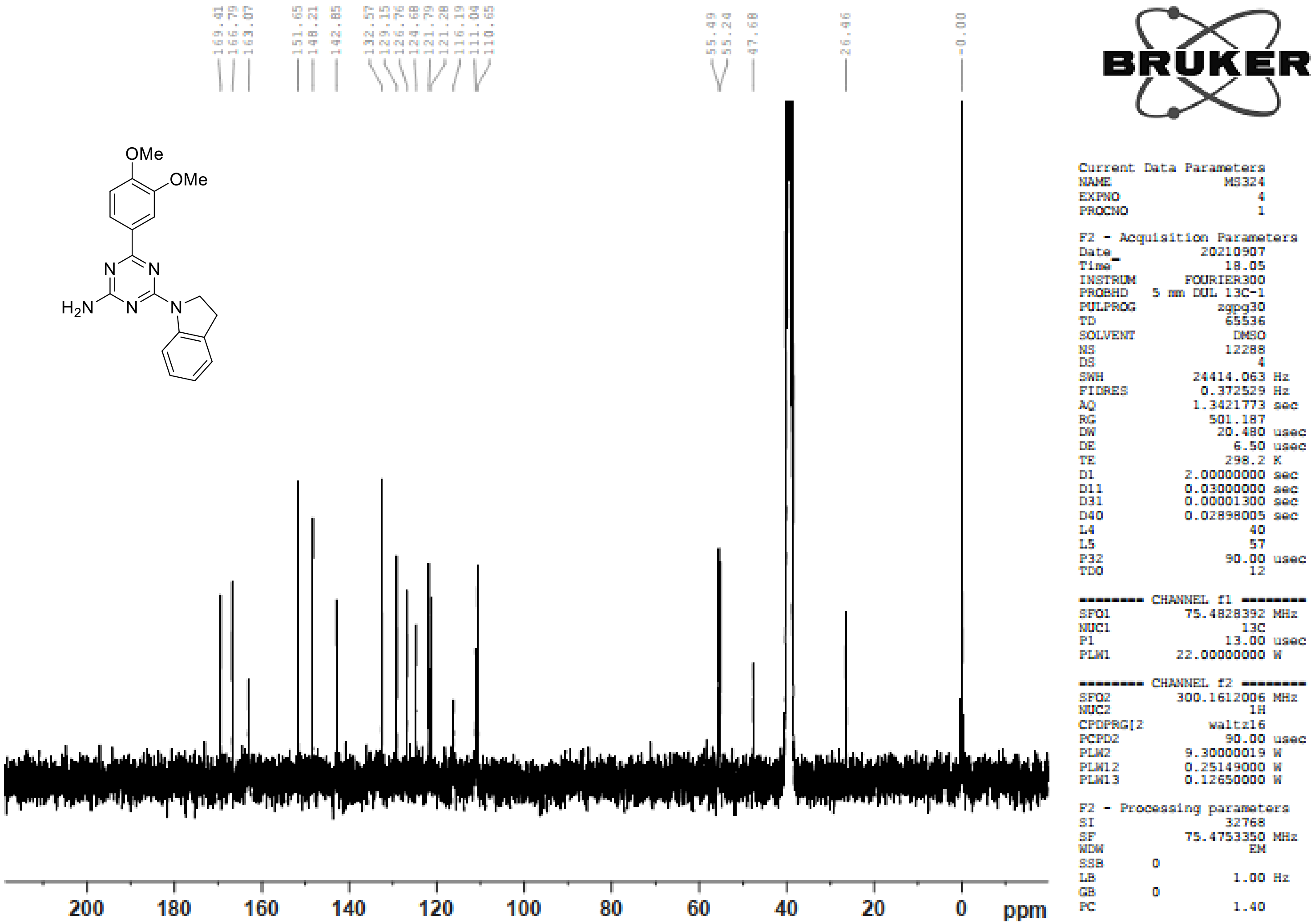

6-(3,4,5-Trimethoxyphenyl)-4-(indolin-1-yl)-1,3,5-triazine-2-amine (**9**{*7*,*17*})

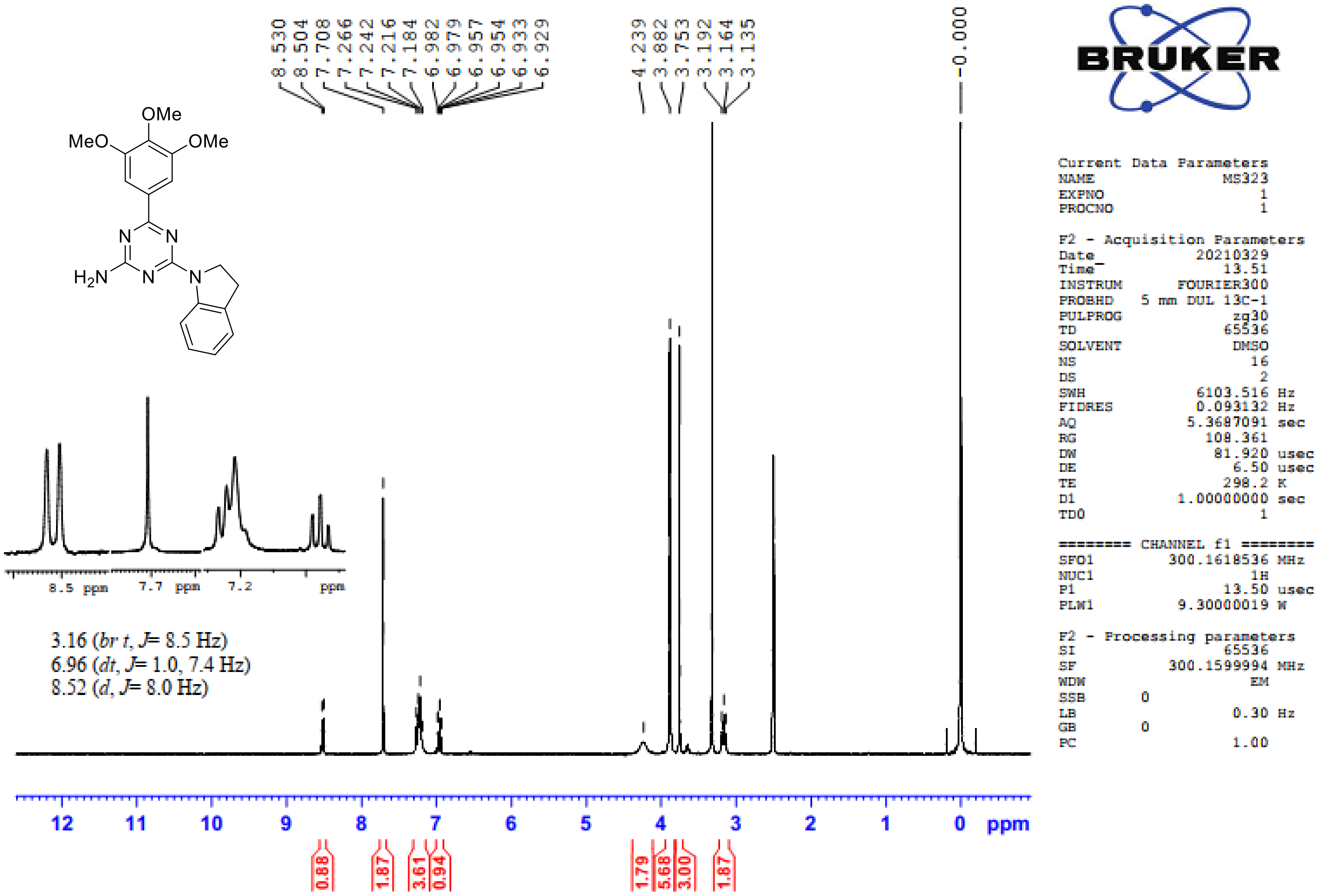

6-(3,4,5-Trimethoxyphenyl)-4-(indolin-1-yl)-1,3,5-triazine-2-amine (**9**{*7*,*17*})

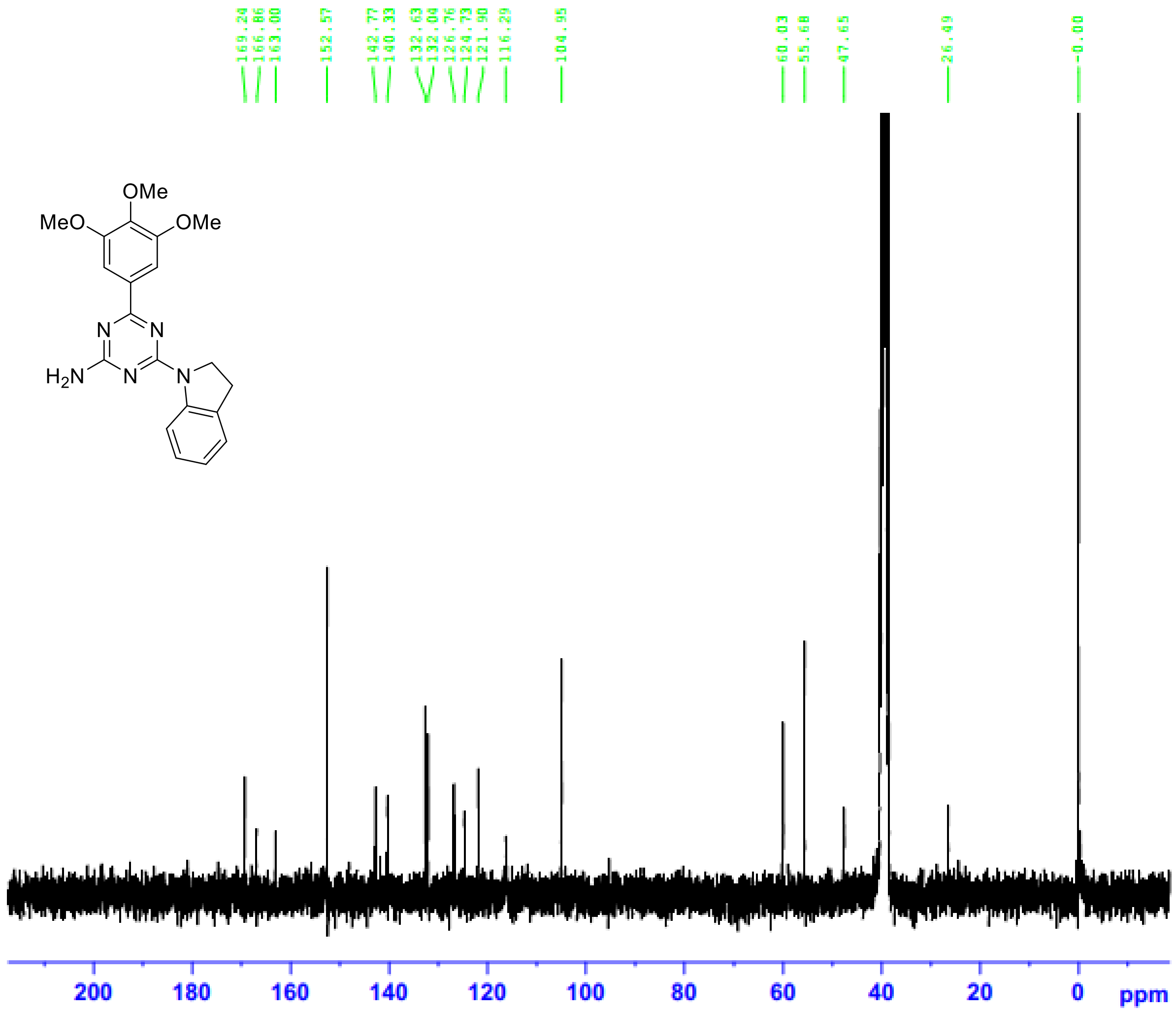

Current Data Parameters
NAME MS323
EXPNO 2
PROCNO 1

F2 - Acquisition Parameters
Date_ 20210401
Time 19.05
INSTRUM FOURIER300
PROBHD 5 mm DUL 13C-1
PULPROG zgpg30
TD 65536
SOLVENT DMSO
NS 12288
DS 4
SWH 24414.063 Hz
FIDRES 0.372529 Hz
AQ 1.3421773 sec
RG 501.187
DW 20.480 usec
DE 6.50 usec
TE 298.2 K
D1 2.00000000 sec
D11 0.03000000 sec
D31 0.00001300 sec
D40 0.02898005 sec
L4 40
L5 57
P32 90.00 usec
TD0 12

======== CHANNEL f1 ========
SFO1 75.4828392 MHz
NUC1 13C
P1 13.00 usec
PLW1 22.00000000 W

======== CHANNEL f2 ========
SFO2 300.1612006 MHz
NUC2 1H
CPDPRG[2 waltz16
PCPD2 90.00 usec
PLW2 9.30000019 W
PLW12 0.25149000 W
PLW13 0.12650000 W

F2 - Processing parameters
SI 32768
SF 75.4753350 MHz
WDW EM
SSB 0
LB 1.00 Hz
GB 0
PC 1.40

4-(Indolin-1-yl)-6-(2-thienyl)-1,3,5-triazine-2-amine (**9**{*7*,*18*})

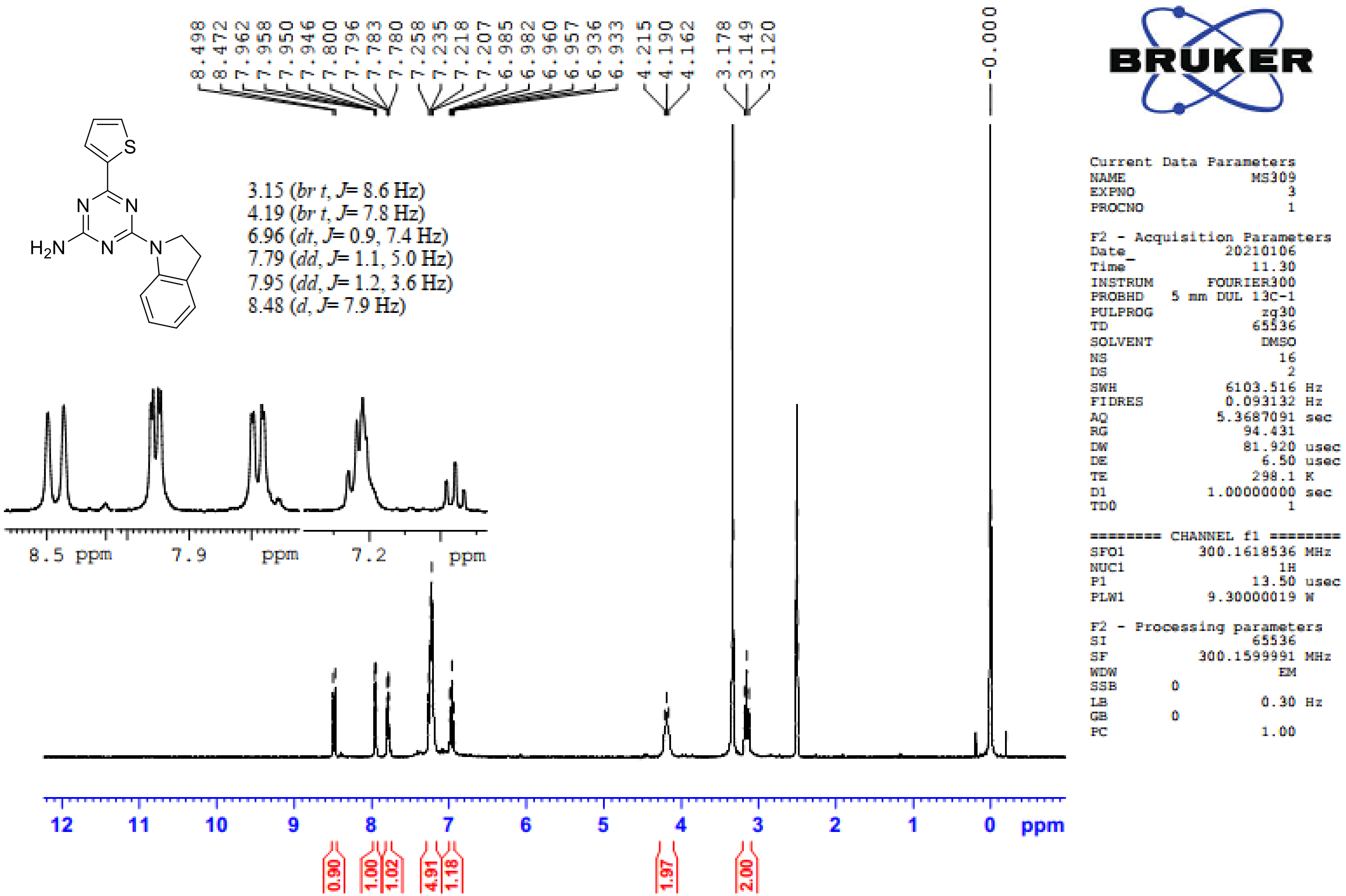

4-(Indolin-1-yl)-6-(2-thienyl)-1,3,5-triazine-2-amine (**9**{*7*,*18*})

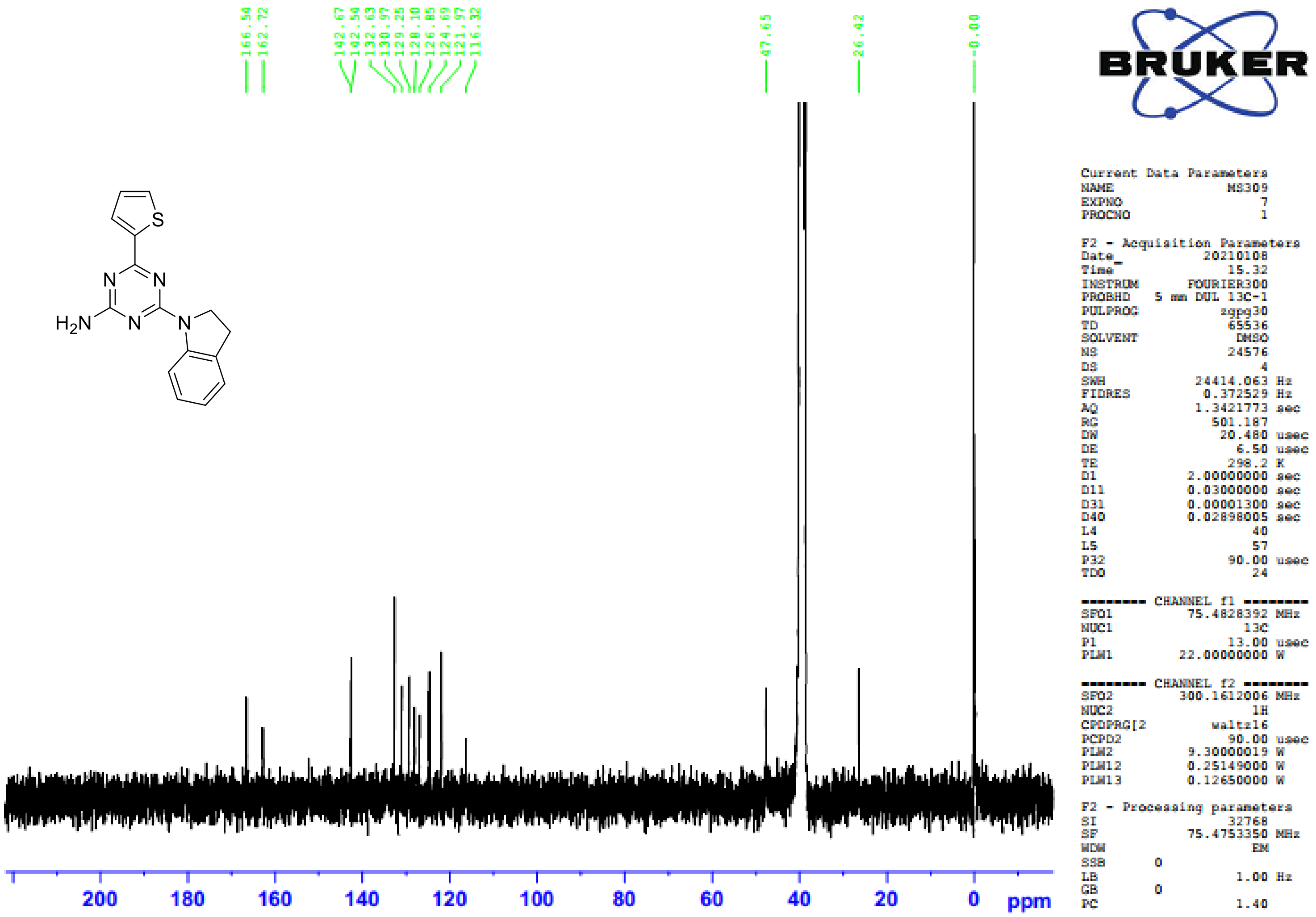

Supplement: RA-014-D3RA08091A-s001 [file RA-014-D3RA08091A-s001.pdf]
